# Genome-wide identification of A-to-I RNA editing events provides the functional implications in PDAC

**DOI:** 10.3389/fonc.2023.1092046

**Published:** 2023-02-21

**Authors:** Yue Mei, Dong Liang, Bin Ai, Tengjiao Wang, Shiwei Guo, Gang Jin, Dong Yu

**Affiliations:** ^1^ Department of Precision Medicine, Translational Medicine Research Center, Naval Medical University, Shanghai, China; ^2^ Shanghai Key Laboratory of Cell Engineering, Shanghai, China; ^3^ Department of General Surgery, Changhai Hospital, Naval Medical University, Shanghai, China

**Keywords:** RNA editing, PDAC, prognosis, RNA secondary structure, immune genes, alternative splicing

## Abstract

**Introduction:**

RNA editing, a wide-acknowledged post-transcriptional mechanism, has been reported to be involved in the occurrence and development of cancer, especially the abnormal alteration of adenosine to inosine. However, fewer studies focus on pancreaticcancer. Therefore, we aimed to explore the possible linkages between altered RNA editing events and the development of PDAC.

**Method:**

We characterized the global A-to-I RNA editing spectrum from RNA and matched whole-genome sequencing data of 41 primary PDAC and adjacent normal tissues. The following analyses were performed: different editing level and RNA expression analysis,pathway analysis, motif analysis, RNA secondary structure analysis, alternative splicing events analysis, and survival analysis.The RNA editing of single-cell RNA public sequencing data was also characterized.

**Result:**

A large number of adaptive RNA editing events with significant differences in editing levels were identified, which are mainly regulated by ADAR1. Moreover, RNA editing in tumors has a higher editing level and more abundant editing sites in general. 140genes were screened out since they were identified with significantly different RNA editing events and were significantly different in expression level between tumor and matched normal samples. Further analysis showed a preference that in the tumor-specific group, they are mainly enriched in cancer-related signal pathways, while in the normal tissue-specific group, they are mainly enriched in pancreatic secretion. At the same time, we also found positively selected differentially edited sites in a series of cancer immune genes, including EGF, IGF1R, and PIK3CD. RNA editing might participate in pathogenisis of PDAC through regulating the alternative splicing and RNA secondary structure of important genesto further regulate gene expression and protein synthesis, including RAB27B and CERS4. Furthermore, single cell sequencing results showed that type2 ductal cells contributed the most to RNA editing events in tumors.

**Conclusion:**

RNA editing is an epigenetic mechanism involved in the occurrence and development of pancreatic cancer, which has the potential to diagnose of PDAC and is closely related to the prognosis.

## Introduction

RNA editing, one of the most common and abundant forms of posttranscriptional RNA modifications, has a vital impact on many biological processes (1), including missense codon changes (2), modulation of alteration splicing, RNA secondary structure, or modification of regulatory RNAs and their binding sites. It has been declared that RNA editing is related to many cancers, and abnormal changes in RNA editing level can induce cancers (3), including hepatocellular carcinoma (4), chronic lymphocytic leukemia (5), and ovarian cancer (6). Various phenomena and evidence have suggested that RNA editing has important physiological and pathological significance. For example, Masayuki Sakurai’s team has found that abnormally regulated RNA editing events in the central nervous system played an important role in neurological development and brain function and were related to the pathogenesis of neurological and psychiatric disorders (7). Peng’s team has found that A-to-I RNA editing events in cancer is a novel source of cancer protein heterogeneity, which can promote the protein diversity of cancer cells by altering the amino acid sequences (8). Therefore, RNA editing events in cancer should be paid more attention.

In humans, the most prevalent RNA editing event is the conversion of adenosine (A) on the double-stranded RNAs to inosine (I) through oxidative deamination reaction under the catalysis of adenosine deaminase ADARs (9). There are three types of ADAR proteins in mammals, ADAR1, ADAR2 (ADARB1), and ADAR3 (ADARB2), of which ADAR1 and ADAR2 are the unique mediators of A-to-I RNA editing and exist in most human tissues. Recent research has indicated that the abnormal expression of ADARs can trigger various diseases (2, 10). ADAR1 and ADAR2 are reported to be involved in the cell proliferation activity and inflammatory response (11, 12). In Okugawa’s study, the abnormal activity of RNA editing in tumors is closely related to the overexpression of ADAR1 (13).

Various studies show that RNA editing participates in the pathogenesis of tumors and has the potential as a clinical indicator. Chigaev’s study has systematically characterized the RNA editing genomic landscape of various cancers conducted through The Cancer Genome Atlas (TCGA) project or Genotype-Tissue Expression (GTEx) Project (14), which presents a difference in RNA editing level between tumor and normal samples. Higher levels of RNA editing were identified in most tumor samples (for example, head and neck cancer, breast cancer, and thyroid cancer). However, only a few coding RNA editing sites have been characterized (4), such as AZIN1, GABRA3, FLNB, SLC22A3, and COPA, which can affect tumor progression. Stably edited AZIN1 acts as an analog of ornithine decarboxylase (ODC) to prevent the degradation of ODC and cyclin D1, resulting in increased cell proliferation, metastasis potential and tumor initiation in colorectal cancer (15), esophageal squamous cell carcinoma (16), and hepatocellular carcinoma (17). In cervical cancer, once a tumor-suppressor BLCAP is edited, it loses its ability to interact with and inactivate STAT3, thereby increasing cell proliferation (18).

Pancreatic cancer is one of the malignant tumors with the highest fatality rate, gradually increased incidence, difficult early diagnosis, poor treatment effect, and strong tumor heterogeneity. However, the pathogenesis of pancreatic cancer is still unclear. Here, we aim to explore the pathogenic mechanism of pancreatic ductal adenocarcinoma (PDAC) from the perspective of RNA editing. We obtained a global profile on RNA editing events of PDAC based on the paired RNA and WGS data from 41 patients and found specific RNA editing events, genes, and signal pathways related to PDAC. A series of differential RNA editing events (DREs) was identified to be located at the important function genes, including pancreatic secretion, tumor immune-related genes, most of which also showed significant prognosis associations in PDAC. Thus, RNA editing might take a role in pathogenesis and provide implications for clinical outcome in PDAC.

## Materials and methods

### Patients and samples

The patients diagnosed with PDAC were recruited from Changhai Hospital, Shanghai. All patients have signed a written informed consent form for collection and use of samples. A total of 41 pairs of tumors and their distal normal tissues were obtained from surgical specimens and frozen at -80°C immediately before nucleic acid extraction. All procedures have complied with the Code of Ethics of the World Medical Association (Declaration of Helsinki) and the guidelines of the institutional review committee of the Shanghai Institute of Nutrition and Health, Chinese Academy of Sciences.

### DNA/RNA extraction and sequencing

Total RNA was isolated using the Qiagen RNeasy Kit (Qiagen, Germantown, MD) and treated with DNase I (New England Biolabs). Total DNA was extracted using the QIAamp DNA Mini Kit (Qiagen, Germantown, MD). The concentration was quantified by the NanoDrop One (Thermo Fisher Scientific, MA, USA), and the RNA quality was analyzed by the 2100 Agilent Bioanalyzer (Agilent Technologies, Inc., Santa Clara, CA). Libraries were generated using a standard library preparation kit (KAPA) according to the manufacturer’s instructions. Libraries were quantified on the Qubit4 (Thermo Fisher Scientific, MA, USA) with the High Sensitivity DNA Assay (Thermo Fisher Scientific, MA, USA). Samples were equimolar pooled and sequenced on the Illumina NovaSeq (Illumina, San Diego, CA, USA) in 150-bp paired-end mode.

### Data preprocessing

The quality of raw reads was assessed by FastQC (https://github.com/s-andrews/FastQC), and subsequently the low-quality data were filtered by fastp (https://github.com/OpenGene/fastp). The hg19/GRCh37 of the human genome was used as reference. RNA-seq reads were aligned onto the reference genome using STAR (https://github.com/alexdobin/STAR) (parameters: –outSAMstrandField intronMotif –outSAMattributes All –readFilesCommand zcat –outFilterType BySJout –outFilterMultimapNmax 1 –alignSJoverhangMin8 –alignSJDBoverhangMin 1 –outFilterMismatchNmax 999 –outFilterMismatchNoverLmax 0.04 –alignIntronMin 20 –alignIntronMax 1000000 –alignMatesGapMax 1000000). WGS reads were aligned by BWA (https://github.com/lh3/bwa). All alignments were duplicated by Picard MarkDuplicates (http://broadinstitute.github.io/picard/), sorted, and indexed using SAMtools (https://github.com/samtools/samtools). Computationally, RNA editing is identified as a single-nucleotide base change between DNA and RNA. The RNA and DNA variants in each sample were both detected by REDItools (19) (https://github.com/BioinfoUNIBA/REDItools2) under unstranded strategy. Then, the sites containing DNA variants were removed and the rest were considered as RNA editing sites. Meanwhile, we also used SPRINT (20) (https://github.com/jumphone/SPRINT) to validate these RNA editing sites detected by REDItools.

### Quantification and comparison of RNA editing levels

**Figure 1:**
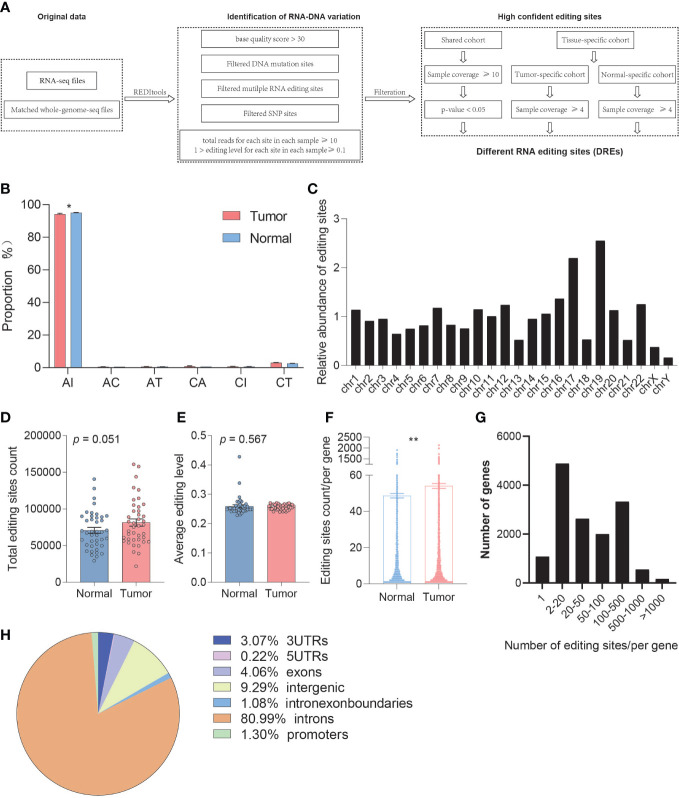
Comparison of the overall A-to-I RNA editing between paired tumor and normal samples. **(A)** The workflow for identification of editing sites with high confidence. **(B)** Genomic distribution of editing sites between normal and tumor samples. **(C)** The overall distribution of editing sites by chromosomes. **(D)** The change in editing count between normal and tumor samples. **(E)** The change in average editing level between normal and tumor samples based on REDItools. **(F)** The change in editing site count located in one gene between normal and tumor samples based on REDItools. **(G)** The overall distribution of editing site count located in one gene. **(H)** The distributions of RNA editing sites in different types of RNA regions. *p < 0.05, **p < 0.001.

The number of A-to-I RNA editing sites is huge, but most of the sites exhibit low editing levels and low sample coverage. These editing events are often caused by non-specific overediting, whereas most of these editing sites are neutral or slightly harmful. This poses a huge challenge for detecting editing sites with high confidence. Therefore, we further obtain credible RNA editing sites by satisfying stringent requirements (base quality score >30, total reads for each site in each sample ≥10, 1 > editing level for each site in each sample ≥0.1, remove sites with DNA mutation and multiple RNA variants, remove SNP sites) (**Figure 1A**).

RNA editing sites were classified into three groups, shared, normal-specific, and tumor-specific group, which implicated that RNA editing can not only produce new cancer-specific changes but also reedit existing editing sites in normal tissues to a certain extent. For the shared group, all editing sites were occurred in both tumor and normal samples and filtered by sample coverage over 10. The editing site was considered significantly different with a Fisher’s exact p value less than 0.05 based on editing level. For two specific groups, the editing sites were occurred only in normal or tumor samples and filtered by sample coverage (≥4).

### Identification of differentially expressed genes

Read counts per gene were calculated for each sample using hisat2 (http://ccb.jhu.edu/software/hisat2) and StringTie (http://ccb.jhu.edu/software/stringtie/). Differential expression analysis was performed underlying the Bioconductor package Deseq2 version 1.32.0 (http://www.bioconductor.org/packages/release/bioc/html/DESeq2.html), and the different expression genes were identified by filtering under log_2_|fold-change| ≥1 and padj <0.05.

### Gene ontology enrichment analysis

We used Kyoto Encyclopedia of Genes and Genomes (KEGG) pathways and Gene Ontology (GO) molecular pathway enrichment analysis for the genes with RNA editing sites between tumor and normal tissues through R package “clusterProfiler” (http://www.bioconductor.org/packages/release/bioc/html/clusterProfiler.html). Statistical significance was defined as *p.adjust* < 0.05.

### Motif search by MEME

The consensus motif in the MEME suite of motif-based sequence analysis tools (version 5.3.3, https://meme-suite.org/meme/) was identified using two motif widths of 30 and 50.

### Construction of the protein–protein interaction network and module

We used the database of Search Tool for Retrieval of Interacting Genes (STRING) (ver. 11.0, http://www.string-db.org/), an online tool to analyze protein network characteristics and make protein network visualization, to evaluate the network of protein–protein interaction (PPI) and calculate K-means for clustering.

### RNA secondary structure analysis

To predict the RNA secondary structure alteration derived from individual RNA editing events, we used the extended surrounding region (± 1000 nt around the editing site) to search for minimum free energy (MFE) and partition function using RNAfold (http://rna.tbi.univie.ac.at/cgi-bin/RNAWebSuite/RNAfold.cgi). Meanwhile, MXfold2 (https://github.com/mxfold/mxfold2) was also applied to predict the RNA secondary structure of RNA sequence surrounding the editing sites (± 250 nt).

### Alternative splicing event analysis

We used rMATS (https://rnaseq-mats.sourceforge.net/index.html) to detect and analyze alternative splicing events, including skipped exon, alternative 5′ splice site (A5SS), alternative 3′ splice site (A3SS), mutually exclusive exons (MXE), and retained intron (RI).

### RNA editing extraction from single-cell RNA sequencing

Cell Ranger (https://support.10xgenomics.com/single-cell-gene-expression/software/pipelines/latest/what-is-cell-ranger) and Seurat (https://satijalab.org/seurat/) were applied to cluster and identify the cell types based on known marker expression. The marker genes were PRSS1, CTRB1, CTRB2, and REG1B for acinar cells, MS4A1, CD79A, CD79B, and CD52 for B cells, AMBP, CFTR, and MMP7 for type1 ductal cells, KRT19, KRT7, TSPAN8, and SLPI for type 2 ductal cells, CHGB, CHGA, INS, and IAPP for endocrine cells, CDH5, PLVAP, VWF, and CLDN5 for endothelial cells, LUM, DCN, and COL1A1 for fibroblast cells, AIF1, CD64, CD14, and CD68 for macrophage cells, ACTA2, PDGFRB, and ADIRF for stellate cells, and CD3E, CD4, and CD8 T cells (21). The reads for each cell type were extracted and combined. RNA editing events for each type were identified by REDItools. Then, credible RNA editing sites were filtered by satisfying stringent requirements (total reads for each site in each sample ≥10, 1 > editing level for each site in each sample ≥0.1, remove SNP sites).

### Survival analyses

The survival analyses were calculated by GEPIA2 (http://gepia2.cancer-pku.cn/#index) based on TCGA-PAAD datasets and ONCOMINE (https://www.oncomine.org) integrated pancreatic cancer data from TCGA and GEO.

### *F_ST_* calculation

F_ST_ analysis, as a genetic signature of positive selection, was performed using VCFtools.

### Statistical analysis

Statistical analysis was performed by GraphPad Prism 9 (GraphPad Software, San Diego, CA, USA) and R packages. A value of p < 0.05 was considered statistically significant. Data were expressed as mean ± SEM.

## Results

### Global properties of the inferred RNA editing sites in tumor and normal samples

To better understand the relationship between the RNA editing and PDAC, we respectively delineated the RNA editing profiles of normal and tumor samples and found that PDAC showed a severe A-to-I RNA editing imbalance. We analyzed the composition of high confident editing events and found that these events were enriched in A-to-I alternation (95.11%), which showed significant differences between tumor and normal samples (REDItools, paired *t*-test, *p* = 0.012) (**Figure 1B**). The distribution of RNA editing events in chromosome was also analyzed, and a preference in chromosomes 17 and 19 was observed (**Figure 1C**, **Figure S1C**).

We then assessed whether RNA editing sites showed differential editing levels between tumor and normal samples. A total of 1,657,409 and 654,485 unique A-to-I RNA editing sites were identified by REDItools and SPRINT, respectively, in which 504,659 editing sites were overlapped. The average number of A-to-I editing sites in tumor (average = 81504) was slightly higher than that in normal tissues (average = 70812) (paired *t*-test, *p* = 0.051) (**Figure 1D**, **Figure S1D**). A percentage of 56.10% of the samples showed a hyper average editing level in tumors than those in normal tissues without difference overall (REDItools, paired *t*-test, *p* = 0.567) (**Figure 1E**, **Figure S1E**). We also observed several genes that have been reported to change the editing level in cancer and even drive early tumor invasion and metastasis, including FLNB, SLC22A3, and AZIN1, which had significantly different editing levels in PDAC.

We further performed gene annotation and found that the number of editing sites per gene in tumor tissue was significantly higher than that in normal tissue (paired *t*-test, *p* = 0.002) (**Figure 1F**). The distribution map showed that most genes have multiple editing sites (**Figure 1G**). The types of RNA region showed an enrichment in intronic (80.99%), intergenic (9.29%), 3′UTR (3.07%), and exonic (4.06%) (**Figure 1H**).

### RNA editing showed different mechanisms in tumors and normal tissues

**Figure 2:**
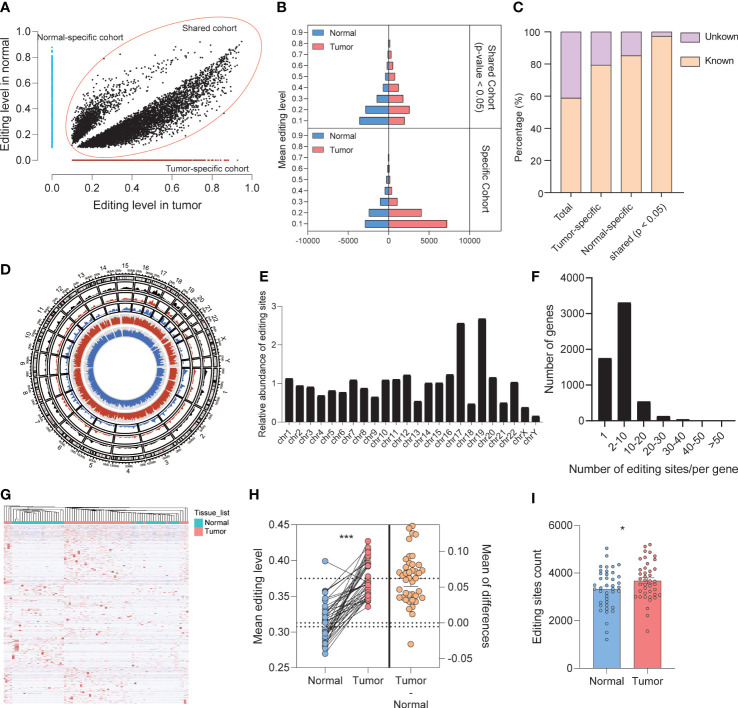
Identification and patterns of different A-to-I RNA editing sites. **(A)** The distribution of mean editing level on DREs among three groups. **(B)** The distribution of DREs’ editing level between normal and tumor samples. **(C)** Comparison of editing events with REDIportal including huge known editing events. **(D)** The circos diagram displayed the distribution of DREs among chromosomes. The black, red, and blue areas showed the density of DREs in the shared, tumor-specific, and normal-specific groups, respectively. The red and blue lines in the center displayed the editing level of each DRE. **(E)** The overall distribution of DREs upon chromosomes. **(F)** The overall distribution of DRE count located in one gene. **(G)** Heat map showing that DREs can effectively distinguish normal tissues from tumors. **(H)** The mean editing level of DREs in paired tumor and normal tissues. **(I)** The editing sites count of DREs in normal and tumor tissues. *p < 0.05, ***p < 0.0001.

From a quantitative and qualitative perspective, the imbalance of RNA editing detected by REDItools was divided into three modes, shared group, tumor-specific group, and normal-specific group, based on the distribution characteristics of RNA editing events that occurred in tumor and normal samples. For A-to-I editing events, 13,042 and 7,297 events (belonging to 3,816 and 2,594 genes, respectively) occurred in the tumor-specific and normal-specific groups, respectively (**Figure 2A**), whereas 9,343 events (belonging to 2,812 genes) with significant differences occurred in the shared group (**Figure 2A**). These editing sites among three groups were recognized as different RNA editing sites (DREs), in which 95.68% were identified by SPRINT. The level of DREs mainly ranged from 10% to 30% (**Figure 2B**). In the shared group, there were more sites with high editing levels in tumor samples. Moreover, in the specific group, a low level of editing sites was predominant. Compared with the public REDIportal database (http://srv00.recas.ba.infn.it/atlas/), 58.91% of all identified events were known and 79.35%, 85.19%, and 96.36% were known in the tumor-specific group, normal-specific group, and shared group, respectively (**Figure 2C**). The relative abundance of DREs showed a preference for chromosomes 17 and 19 (**Figures 2D, E**). The distribution of DREs in each gene was consistent with the total result (**Figure 2F**). Meanwhile, the types of RNA region of DREs showed a consistent result with total editing sites (**Figures S1G, H**), and low enrichment of DREs was found in TSS (**FigureS1I**). Eight A-to-I DREs were identified as non-synonymous mutation, which might lead to the change in protein (**Table S1**). Furthermore, the DREs in the shared group displayed a hyper mean editing level and a higher editing event count in tumor than normal tissues (paired *t*-test, editing level: *p* < 0.001, editing event count: *p* = 0.023) (**Figures 2H, I**). In addition, the profile of total 29,682 A-to-I DREs could distinguish tumor and normal tissues well (**Figure 2G**).

To investigate whether the genes with A-to-I DREs are involved in the physiological process of pancreas or tumorigenesis, we obtained 108 enriched pathways for total 5,319 genes with DREs by functional enrichment analysis. Pancreatic cancer, adherens junction, and MAPK signaling pathway were enriched in the tumor-specific group, whereas pancreatic secretion only existed in the normal-specific group (**FigureS2A**). In general, RNA editing events under different modes displayed different functional mechanisms.

### ADAR1 regulates RNA editing events in PDAC

To determine whether ADAR family members have effects on RNA editing imbalance in PDAC, we compared the expression level of ADAR1 and its relation with the editing level and the amount of editing sites.

**Figure 3:**
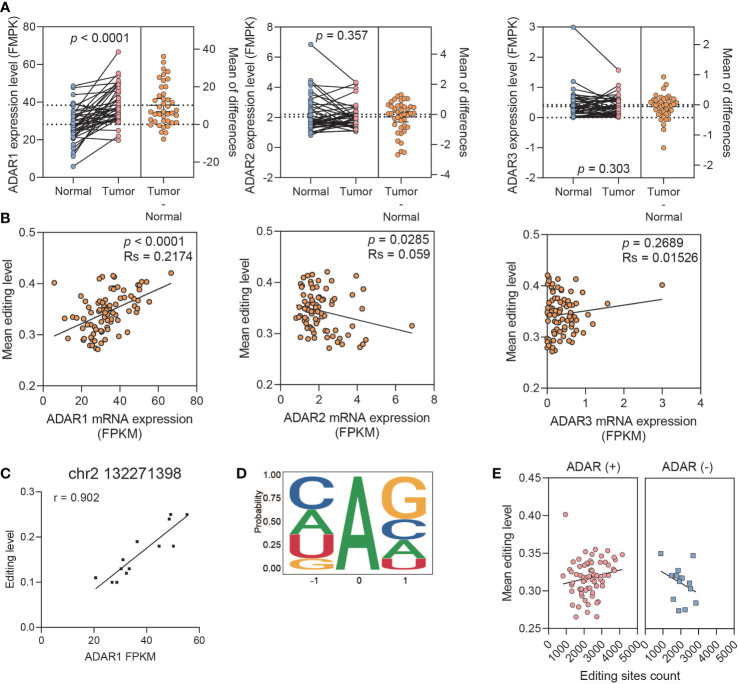
ADAR1 regulates A-to-I RNA editing events in PDAC. **(A)** The relative mRNA expression of ADARs. **(B)** The correlation between the ADAR expression and mean editing level. **(C)** A representative editing site, chr2 132271398, shows the high correlation between its editing level and ADAR1 expression. **(D)** The nucleotide neighboring editing sites show a pattern consistent with known ADAR preference. **(E)** Boxplots showing a positive correlation between editing level and editing site count upon ADAR1 overexpression samples, and a negative correlation upon ADAR1 downexpression.

The expression of ADAR1 in tumor was significantly higher than that in normal tissues, rather than ADAR2 and ADAR3 (paired *t*-test, ADAR1: *p* < 0.0001, ADAR2: *p* = 0.357; ADAR3: *p* = 0.303) (**Figure 3A**). Meanwhile, TCGA and GTEx data analysis showed that the expression profiles of the ADAR family were consistent with our results (**Figure S3A**). The A-to-I mean editing level was positively correlated with ADAR1 expression level, whereas it was lightly negative correlated with ADAR2 (spearman test, ADAR1: *p* < 0.0001, coef = 0.2174, ADAR2: *p* = 0.0285, coef = 0.059, ADAR3: *p* = 0.2689, coef = 0.01526) (**Figure 3B**). The global association detected between ADAR1 expression and average editing level was also observed at each editing site (**Figure 3C**). Meanwhile, only ADAR1 observed a significant association with prognosis (ADAR1, *p* = 0.042; ADAR2, *p* = 0.29; ADAR3, *p* = 0.066) (**Figure S3B**). We then explored the triplet codon characteristics of A-to-I editing sites and found sequence preference, especially upstream G underrepresentation and downstream G excess, which was consistent with the known ADAR1 motif (**Figure 3D**).

Then, we further classified samples with ADAR expression between paired tumor and normal tissues into ADAR1-upregulated and -downregulated groups to explore the function of ADAR1 on regulating RNA editing events. We found that the mean editing level was positively correlated with the amount of editing sites in the ADAR1 upregulated group. However, the opposite result was observed in the ADAR1-downregulated group (**Figure 3E**).

Above all, these results suggest that the differential expression of ADAR1 manipulate the A-to-I imbalance in tumor samples in PDAC.

### The genes with functional DREs

**Figure 4:**
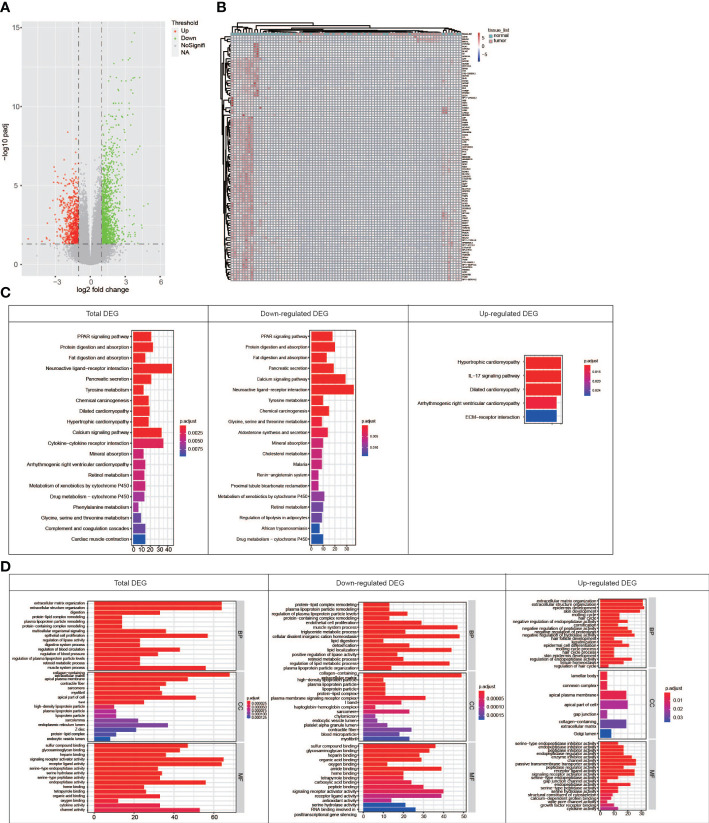
Comparison of the RNA expression between paired tumor and normal samples. **(A)** Volcano map showing genes with different RNA expressions between tumor and normal groups. **(B)** Heat map showing that top 100 DEGs can distinguish normal tissues from tumors well. **(C)** KEGG pathway enrichment for DEGs in total, downregulated, and upregulated patterns. **(D)** GO term enrichment for genes in total, downregulated, and upregulated patterns.

In order to explore the associations between RNA editing and gene expression in PDAC, differential gene expression analysis based on RNA-Seq was performed and 2,022 different expression genes (DEGs) were identified, including 712 upregulated and 1,310 downregulated genes in tumor (**Figure 4A**). The DEGs were also found to distinguish tumor and normal tissues, but not better than the DREs did (**Figure 4B**). Pathway analysis showed that downregulated DEGs were enriched in pancreatic secretion, PPAR signaling pathway, protein digestion and absorption, and cAMP signaling pathway, whereas upregulated DEGs were enriched in ECM–receptor interaction and IL-17 signaling pathway (**Figures 4C, D**). Notably, in the pathogenesis of PDAC, the expression of tumor-related genes changed greatly.

**Figure 5:**
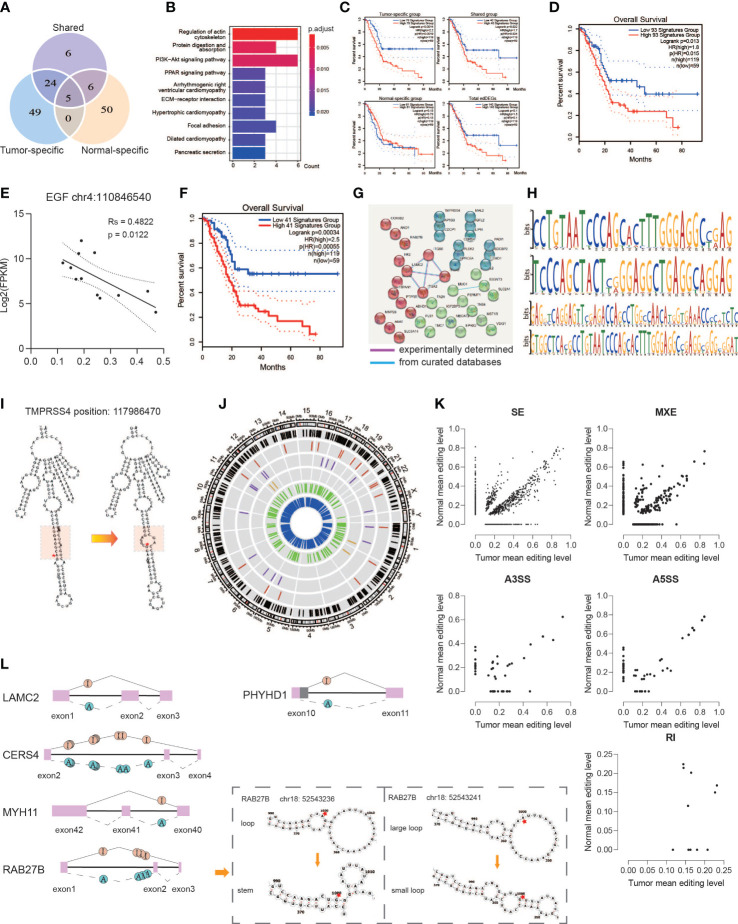
Functional alteration of A-to-I DREs. **(A)** Editing sites located in the 140 edDEGs coexisting in genes with DREs and DEGs among three groups. **(B)** KEGG pathway enrichment for overlapped genes. **(C)** Correlation analysis with overall survivals based on edDEGs in the tumor-specific group, shared group, and normal-specific group and in general. **(D)** Correlation analysis with overall survivals based on 93 overlapped genes matching with the expression status in GEPIA2. **(E)** A representative RNA editing site displaying the significant correlation between RNA editing level and RNA expression. **(F)** Correlation analysis with overall survivals based on 41 edDEGs significantly associated with prognosis in TCGA-PAAD dataset. **(G)** PPI shows two important interactions among overlapped genes. **(H)** Motif analysis by MEME showing a preference in GA/CA enrichment. **(I)** Representative change of RNA secondary structure in the GA enrichment region due to A-to-I RNA editing at position 117986470 in TMPRSS4. Red star indicates the editing site. Red region represents the GA-rich region. **(J)** Circos diagram displaying the distribution of sDREs in chromosomes. The black line represents total sDRE distribution, and the red, purple, brown, green, and blue lines represent sDREs belonging to A3SS, A5SS, RI, MXE, and SE. **(K)** Scatter diagrams showing the editing level of sDREs among each alternative splicing event. **(L)** Representative alternative splicing events displaying RNA editing events that altered the RNA secondary structure.

In order to detect whether the RNA editing events affect gene expression, we focus on the DEGs with DREs. A total of 140 genes were filtered out (**Figure 5A**), which included 40 genes with 73 editing sites in the shared group, 78 genes with 370 A-to-I editing sites in the tumor-specific group, and 61 genes with 266 A-to-I editing sites in the normal-specific group. These differentially edited sites accompanied by different expressions were named eDREs, and these genes were named as edDEGs. The genes in the shared group were enriched in pancreatic secretion; focal adhesion; protein digestion and absorption; regulation of actin cytoskeleton; alanine, aspartate, and glutamate metabolism; and ECM–receptor interaction (**Figure 5B**). For genes in the tumor-specific group, they were enriched in ECM–receptor interaction, focal adhesion, central carbon metabolism in cancer, regulation of actin cytoskeleton, and others. For genes in the normal-specific group, KEGG pathways were enriched in the PPAR signaling pathway; alanine, aspartate, and glutamate metabolism; pancreatic secretion; and protein digestion and absorption. Furthermore, only the gene set in the tumor-specific group observed a significant association with prognosis (total 140 edDEGs: *p* = 0.1, shared group: *p* = 0.022, normal-specific group: *p* = 0.13, tumor-specific group, *p* = 0.001) (**Figure 5C**). These results suggested that RNA editing might take a role in the pathogenesis of PDAC.

Then, the expression levels of the 140 edDEGs were validated using TCGA-PAAD datasets (http://gepia2.cancer-pku.cn/#index). A set of 93 edDEGs were filtered out to showed similar expression levels with our results and showed a significant association with prognosis (*p* = 0.013) (**Figure 5D**). We then analyzed the association between the level of RNA editing and the level of gene expression and found that it could be either positive or negative. For example, the level of the RNA editing event in chromosome 4: 110846540 on EGF showed a significantly negative correlation with its expression level (*p* = 0.0122) (**Figure 5E**).

Among them, 41 edDEGs were found to be significantly associated with prognosis in TCGA-PAAD dataset, the combination of which also showed a significant association with prognosis (*p* < 0.001) (**Figure 5F**). Moreover, the PPI network of 41 edDEGs was divided into three clusters, in which ITGA2, ITGA3, ITGB6, and LAMC2, MUC4, and B3GNT3 were the nodes with most connectivity (**Figure 5G**). These genes were reported to be related with the pathogenesis of PDAC. ITGA2 can promote cell migration, invasion, metastasis, and chemoresistance in PDAC through ECM remodeling (22). LAMC2 can promote cancer progression and gemcitabine resistance through modulation of EMT and ATP-binding cassette transporters in PDAC (23). ITGA3 has been reported with an upregulated expression in PDAC (24). MUC4-promoted neural invasion is mediated by the axon guidance factor Netrin-1 in PDAC (25). In summary, RNA editing indeed affects the expression of important functional genes, suggesting an important role of RNA editing in the pathogenesis of PDAC.

### Functional eDREs affect RNA stability

It was reported that RNA editing could affect RNA stability by altering the RNA secondary structure and alternative splicing (26). Thus, we analyzed the RNA sequences and the corresponding secondary structure close to the editing site. We predicted the potential motif surrounding 177 eDREs derived from the 41 edDEGs by MEME and found the preference of GA-rich and CA-rich regions in sequences (**Figure 5H**). Furthermore, we also found that 42.67% of these eDREs might impact the RNA secondary structure based on RNAfold analysis based on minimum free energy (MFE) and partition function (**Table S2**), which in turn might affect RNA stability and function. For example, the predicted RNA structure was changed from stem to loop at a GA-rich region due to the nearby RNA editing event in chromosome 11: 117986470 on TMPRSS4 (**Figure 5I**), which has been reported as an independent risk factor in PDAC to promote cell proliferation and inhibit apoptosis through activating ERK1/2 Signaling pathway (27).

A total of 3,647 significantly different alternative splicing events were identified between tumor and normal, of which 41.98% contained at least one RNA editing site (**Figure 5J**, **Table S3**). These differentially edited sites accompanied by different alternative splicing events were named sDREs, and these genes were named edDSGs. Major sDREs participated in skipped exons. Among them, LAMC2, RAB27B, PHYHD1, MYH11, and CERS4 were also occurred in 140 edDEGs with co-changes in expression and editing. The alternative splicing events of LAMC2, RAB27B, MYH11, and CERS4 showed that once an RNA editing event occurred on the upstream intron, at least one downstream exon was shipped (**Figure 5L**). Exon 3 of CERS4 is a protein-coding region, and its loss because of A-to-I editing in intron2 would result in truncated protein, thereby affecting its protein function. Two kinds of sDREs in intron 2 of RAB27B lead to an alteration of the RNA secondary structure and also generated an abnormal transcript (**Figure 5L**). PHYHD1 displayed alternative 5′ splicing sites due to A-to-I editing in intron 10 leading to a truncated exon 10 (**Figure 5L**). We further observed the editing levels among various alternative splicing types and found that hyper editing events accounted for a large proportion of each component in the tumor (**Figure 5K**). These suggested that aberrant RNA editing in tumors may lead to aberrant variable splicing through an unstable RNA secondary structure.

These results suggest that RNA editing regulates gene expression and protein synthesis by affecting alternative splicing and the RNA secondary structure.

### Positive selection pressure on the RNA editing events

**Figure 6:**
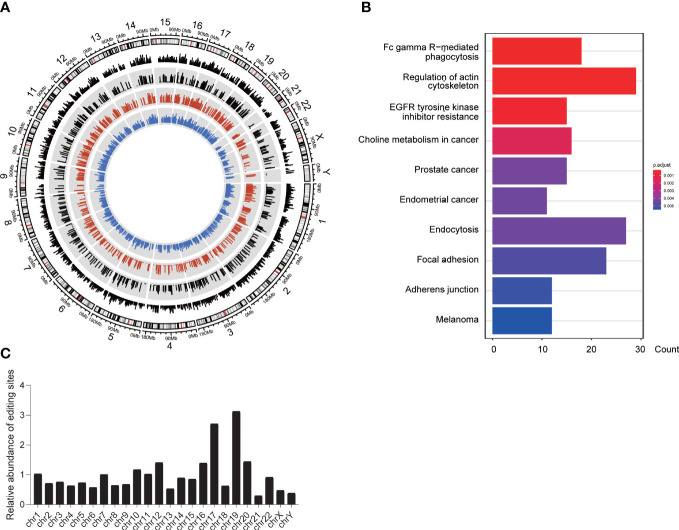
Positive selection of A-to-I DREs. **(A)** The F*_ST_* of DREs among three groups. The white background shows the F*_ST_* score of total DREs, whereas the gray background shows the DREs under positive selection (F_ST_ >= 0.25). The black, red, and blue lines represent the FST score and the mean RNA editing level in the tumor and normal groups, respectively. **(B)** The pathway of genes with a positive signal. **(C)** The chromosomal distribution of DREs with a positive selection.

It was reported that positive selection is one of the mechanisms driving RNA editing (28, 29). In order to determine whether the RNA editing events suffer from positive selection in the tumorigenesis, we performed calculation of *F_ST_* for each DREs. A percentage of 5.48% of DREs belonging to 1,005 genes showed the signal of positive selection (**Figure 6A**). These genes were enriched in regulation of actin cytoskeleton, focal adhesion, and adherens junction (**Figure 6B**), and 48 genes were immune-related genes. Furthermore, we screened out the positive selection signals of all immune genes (**Table S4**). Chromosomes 19 and 17 had relatively more selection signals (**Figure 6C**), which was consistent with the enrichment of editing events in chromosomes 19 and 17. Meanwhile, an interesting result was found in which the positively selected DREs were mainly located in the normal- and tumor-specific groups. The hot genes with the most positive signal events in the normal-specific group were RBPJ, PFKFB3, CAMK1D, ACACB, and WWOX, whereas the hot genes in the tumor-specific group were SLC35F3, PCDH7, NF1, and RAB27B. Among them, RBPJ was an important transcriptional regulator in the Notch signaling pathway (30). PFKFB3 was required for cell-cycle progression and prevention of apoptosis (31). RAB27B expression is considered an independent prognostic marker for PDAC (32).

In summary, positive selection indeed has an effect on the RNA editing in PDAC and has a preference for the RNA editing sites in tumor immune-related genes.

### RNA editing events has a cell type bias

**Figure 7:**
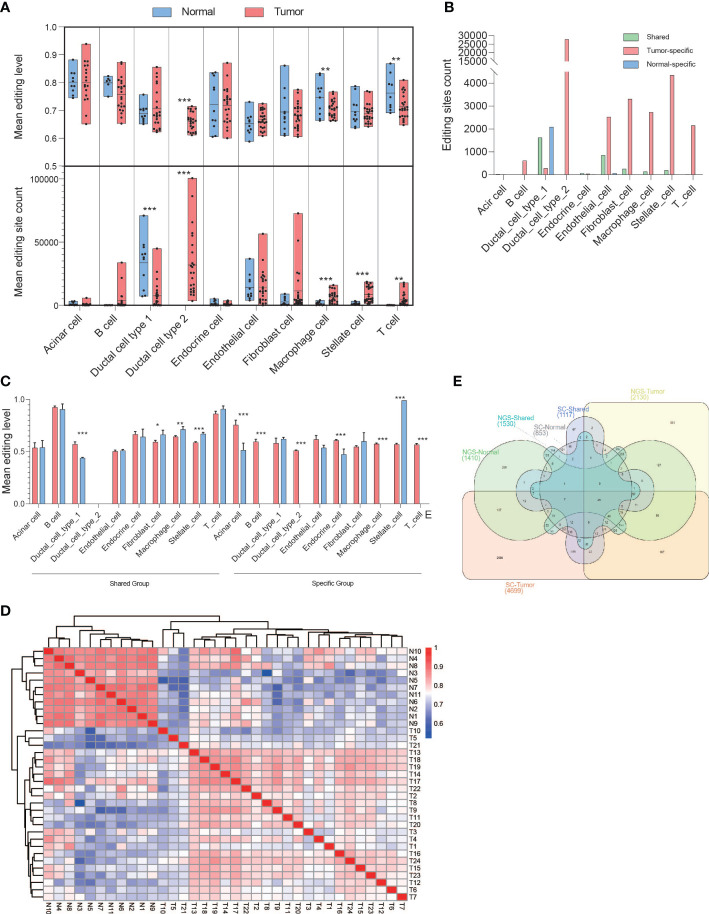
Biased A-to-I DREs in various PDAC cell types based on single-cell sequencing. **(A)** The box diagrams displayed mean editing level (upper) and mean editing site count (bottom) among 10 kinds of PDAC cell types in general. **(B)** Bar diagram displaying the distribution of DREs in each PDAC cell type among the shared, tumor-specific, and normal-specific groups. **(C)** Bar diagram displaying the mean editing level of DREs in each PDAC cell type among the shared, tumor-specific, and normal-specific groups. **(D)** Heatmap showing the correlation among tumor and normal samples. **(E)** Venn diagram showing the distribution of genes with DREs among the shared, tumor-specific, and normal-specific groups based on NGS and single-cell sequencing data. *p < 0.05, **p < 0.001, ***p < 0.0001.

We downloaded a single-cell RNA-seq data set (PRJCA001063) of pancreatic cancer containing 24 tumors and 11 normal pancreases and identified 10 major clusters including type 1 ductal, type 2 ductal, acinar, endocrine, endothelial, fibroblast, stellate, macrophage, T, and B cells, through principal component analysis based on gene expression. We then extracted the RNA editing events for each cluster. In general, the significant difference in editing level occurred in type 2 ductal, macrophage, and T cells, whereas the significant difference in editing sites occurred in type 1 ductal, type 2 ductal, macrophage, stellate, and T cells (**Figure 7A**). Among them, type 2 ductal cells greatly contributed to RNA editing events in pancreatic cancer tissues, whereas type 1 ductal cells had the largest number of RNA editing events in normal tissues. We then identified the DREs and their located genes and found that type 2 ductal, endothelial, fibroblast, stellate, macrophage, and T cells were dominated in tumor-specific editing sites, whereas normal-specific editing sites were dominated type 1 ductal cells (**Figure 7B**). The shared group showed a significantly higher editing level in type1 ductal cells and a significantly lower editing level in fibroblast, stellate, and macrophage in tumors, whereas in the specific group, a significantly higher editing level occurred in acinar, B, type 2 ductal, endocrine, macrophage, and T cells and a significantly lower editing level was displayed in stellate cells (**Figure 7C**). The Pearson analysis showed that it could be distinguished into normal and tumor based on the level of DREs (**Figure 7D**). Then, we compared the distribution of genes with RNA editing sites between RNA-seq of 41 paired PDAC samples and single-cell sequencing data of 35 PDAC (**Figure 7E**). There were 187 overlapped observed only in the tumor-specific group between RNA-seq and single-cell sequencing, of which 72.06% were detected in type 2 ductal cells.

In conclusion, the level and amount of RNA editing events were different among various cell types of PDAC, and type 2 ductal cells contributed the most to RNA editing in tumors.

## Discussion

A-to-I RNA editing is widespread in the human transcriptome, and the genetic variation generated by it can expand the diversity and complexity of the transcriptome. Recent studies have proved that RNA editing participates in the pathogenesis of cancer. In this study, we first focus on the pathogenic mechanism of PDAC from the perspective of RNA editing. Using matched genomic and transcriptomic data from 41 patients, we have delineated the comprehensive A-to-I RNA editing landscape and studied the distribution features of DREs and the associations with gene expression. We have observed that the spectrum of DREs can distinguish tumor and normal tissues well. The analysis of DREs and related genes suggests that RNA editing events are mainly regulated by ADAR1 and are involved in PDAC tumorigenesis. We have also found some RNA editing events that are functional and positively selected in tumor, and the genes with these editing events are closely related to the prognosis.

We have verified that RNA editing is mainly regulated by ADAR1 in PDAC. First, the vast majority of editing sites found fall in the ALU repeat sequence (**Figure S1F**), which is consistent with the reported preference for editing regions of ADAR1 (33). Second, ADAR1 is significantly highly expressed in tumors and is significantly positively correlated with the RNA editing level in PDAC, which is also consistent with the reported results. For example, Leng Han has revealed that the diversity of RNA editing events in tumor samples has the best correlation with the overall expression level of ADAR1 (1). A recent study has determined the frequency of RNA editing events in 17 cancer types in The Cancer Genome Atlas (TCGA) database 3 (34), in which the level of global RNA editing is positively correlated with ADAR1. Third, the triplet codon sequences centered on editing site A have a preference with 5′ U and 3′ G, which corresponds to the known motif of ADAR1 (35). Furthermore, several recent studies have emphasized the role of ADAR1 in cancer development. Chen found that ADAR1 edits multiple sites in the YXXQ motif of BLCAP (a tumor suppressor for bladder cancer), causing it to lose its inhibition of STAT3 activation, thereby promoting tumorigenesis in cervical cancer (CC) (18). Salameh found that ADAR1-mediated editing of prostate cancer antigen 3 (PCA3) increased its stability and expression in prostate cancer (PC), further promoting tumorigenesis (36). Meanwhile, we have also observed that some well-acknowledged pancreatic cancer genes contains DREs, such as BRCA2 (37), ATM (38), and SMAD4 (39). In summary, RNA editing is mainly regulated by ADAR1 to be involved in the occurrence and development of PDAC.

RNA editing events have been reported to suffer under selection pressure. Zhang Rui’s team has found that RNA-edited SNP sites are highly enriched near autoimmune disease-related sites represented by Crohn’s disease, asthma, and allergic dermatitis and are subject to balanced selection (40). Duan’s group has reported that there are a large number of non-synonymous RNA editing sites (Nonsyn) that change amino acids in *Drosophila*, which show adaptive signals and are subject to positive natural selection (28). We boldly speculate that natural selection has an effect on the process of ADAR1-regulated RNA editing events in PDAC. Interestingly, some positively selected RNA editing sites are located in immune genes. For example, in this study, two tumor-specific editing sites in PPARG, chr3:12404457 and chr3:12432364, were identified with positive selection. PPARG has been reported to be expressed in various tumor cells and shows significant association with prognosis in PDAC (*p* = 0.016). The positively selected RNA editing sites in cancer tissues are likely to be produced by self-regulation of constantly adapting to the microenvironment and changing the microenvironment during the survival and reproduction of cancer cells. In a word, the results further indicate that RNA editing may be driven by nature selection to play a role in pathogenesis of PDAC.

RNA editing may be involved in pathogenesis of PDAC by posttranscriptional regulation such as variable splicing and RNA secondary structure change. Sze Jing Tang et al. proposed a novel mechanism in which ADAR1-mediated editing can affect alternative splicing, which in turn affects protein binding (41). They found that ADAR1 specifically edits GA-rich ISS at intron 8 of CCDC15, leading to recruitment of SRSF7 to the edited region and repression of exon 9 inclusion. Our study also found some events, which might affect posttranscriptional regulation. A series of RNA editing sites that affect the level of RNA transcription and alter RNA secondary structure (**Table S2**) have been identified. For example, the RNA editing at chromosome 11: 117986470 in TMPRSS4 can change the RNA secondary structure, and the expression level of TMPRSS4 is significantly correlated with the editing level. These results show that RNA editing events can take a role in pathogenesis of PDAC by changing the stability of the secondary structure and the expression levels of key genes. However, experiments are needed for further validation.

In addition, our results suggest that RNA editing might act as a novel indicator for the diagnosis and prognosis of pancreatic cancer to some extent. We have observed that the RNA editing profile can more clearly distinguish tumors and normal samples, implying that RNA editing has potential for diagnosis of PDAC. The DEGs related to prognosis of PDAC (**Table S2**) suggest that RNA editing has the potential to judge the prognosis of PDAC. Among these genes, 16 genes have been reported to be associated with PDAC, including TMPRSS4 (42), ITGA2, ITGA3 (43), GPRC5A (1), LAMC2 (23), ARNTL2 (44), RAB27B (32), and PADI1 (45). RAB27B, a member of the Rab family GTPases involved in vesicle trafficking, has been found to be involved in PDAC invasion in a few studies. The exosome secretion pathway regulated by RAB27B is considered to be a novel therapeutic target for PDAC (32). In our study, we found that RAB27B can change the secondary structure of mRNA through RNA editing and then generate the transcript missing exon 2, which eventually leads to the structural change of protein. The three genes, VSIG1, IGFL2, and PLS1, have not been reported to be related with PDAC. VSIG1, a cell adhesion protein of the immunoglobulin superfamily, is preferentially expressed in gastric, testicular, esophageal, and ovarian cancers (46). VSIG1 has been reported to be related to the metastatic behavior of various colon cancer cell types (47), and nuclear positivity of VSIG1 has been observed in all cases of distant metastasis of gastrointestinal stromal tumors (48). PLS1 has been reported to promote metastasis of colorectal cancer through the IQGAP1/Rac1/ERK pathway (49). IGFL2 has been identified as a member of 12 marker panel of cancer-associated fibroblasts associated with the progression of hepatocellular carcinoma (50). The roles of these four genes with significantly differential expression and RNA editing levels in pancreatic cancer deserve further study.

## Conclusions

These findings further expand our understanding of the role of RNA editing events in the occurrence and development of tumors. The specific occurrence of events in tumors may also have the potential to serve as important markers for PDAC diagnosis and prognosis. However, the understanding of the function of RNA editing is not comprehensive enough, and what kind of RNA editing events in which PDAC cells play a critical role in the occurrence and development of PDAC still needs a large amount of data for further research.

## Data availability statement

The datasets presented in this study can be found in online repositories. The names of the repository/repositories and accession number(s) can be found below: https://www.ncbi.nlm.nih.gov/, GSE172356, https://db.cngb.org/, CNP0003871.

## Ethics statement

The studies involving human participants were reviewed and approved by Naval Medical University. The patients/participants provided their written informed consent to participate in this study.

## Author contributions

DY, GJ, and YM conceived the study. YM, DL, and TW processed the sequencing data. YM and BA analyzed the data. YM wrote the manuscript. SG and DY revised the manuscript. All authors contributed to the article and approved the submitted version.

## Supplementary material

Figure S1 Comparison of the Overall A-to-I RNA Editing between Paired Tumor and Normal Samples. (A) The RNA variant distribution in individual sample. (B) The general RNA variant distribution among total samples. (C) The overall distribution of editing sites by chromosomes based on REDItools and SPRINT. (D) The change of editing count between normal and tumor samples based on SPRINT. (E) The change of average editing level between normal and tumor samples based on SPRINT. (F)The distribution of total A-to-I editing sites in repeat region. (G-H) The distribution of A-to-I DREs in different types of RNA regions. (I) The distance to TSS of editing sites between tumor and normal.

Figure S2 (A) The enriched pathway of the genes with A-to-I DREs.

Figure S3 Effect of ADARs on pancreatic cancer. (A) The relative expression of ADARs in PDAC based on TCGA and GTEx data. (B) The correlation analysis with overall survivals based on ADARs expression in PDAC.

## References

[1] Kung CP Maggi LB Jr. Weber JD. The role of RNA editing in cancer development and metabolic disorders. Front Endocrinol (Lausanne) (2018) 9:762. doi: 10.3389/fendo.2018.00762 30619092 PMC6305585

[2] Chan THM Lin CH Qi LH Fei J Li Y Yong KJ. A disrupted RNA editing balance mediated by ADARs (Adenosine DeAminases that act on RNA) in human hepatocellular carcinoma. Gut (2014) 63:832–43. doi: 10.1136/gutjnl-2012-304037 PMC3995272 23766440

[3] Wang C Zou J Ma X Wang E Peng G. Mechanisms and implications of ADAR-mediated RNA editing in cancer. Cancer Lett (2017) 411:27–34. doi: 10.1016/j.canlet.2017.09.036 28974449

[4] Qi L Chan TH Tenen DG Chen L. RNA Editome imbalance in hepatocellular carcinoma. Cancer Res (2014) 74:1301–6. doi: 10.1158/0008-5472.CAN-13-3485 24556721

[5] Gassner FJ Zaborsky N Feldbacher D Greil R Geisberger R. RNA Editing alters miRNA function in chronic lymphocytic leukemia. Cancers (Basel) (2020) 12(5):1159. doi: 10.3390/cancers12051159 32380696 PMC7280959

[6] Zhou C Wei Z Zhang L Yang Z Liu Q. Systematically characterizing a-to-I RNA editing neoantigens in cancer. Front Oncol (2020) 10:593989. doi: 10.3389/fonc.2020.593989 33363023 PMC7758481

[7] Yang Y Okada S Sakurai M. Adenosine-to-inosine RNA editing in neurological development and disease. RNA Biol (2021) 18:999–1013. doi: 10.1080/15476286.2020.1867797 33393416 PMC8216190

[8] Peng X Xu X Wang Y Hawke DH Yu S Han L. A-to-I RNA editing contributes to proteomic diversity in cancer. Cancer Cell (2018) 33:817–28.e817. doi: 10.1016/j.ccell.2018.03.026 29706454 PMC5953833

[9] Roth SH Levanon EY Eisenberg E. Genome-wide quantification of ADAR adenosine-to-inosine RNA editing activity. Nat Methods (2019) 16:1131–8. doi: 10.1038/s41592-019-0610-9 31636457

[10] Gannon HS Zou T Kiessling MK Gao GF Cai D Choi PS. Identification of ADAR1 adenosine deaminase dependency in a subset of cancer cells. Nat Commun (2018) 9:5450. doi: 10.1038/s41467-018-07824-4 30575730 PMC6303303

[11] Jiang Q Isquith J Ladel L Mark A Holm F Mason C. Inflammation-driven deaminase deregulation fuels human pre-leukemia stem cell evolution. Cell Rep (2021) 34:108670. doi: 10.1016/j.celrep.2020.108670 33503434 PMC8477897

[12] Tomaselli S Galeano F Alon S Raho S Galardi S Polito VA. Modulation of microRNA editing, expression and processing by ADAR2 deaminase in glioblastoma. Genome Biol (2015) 16:5. doi: 10.1186/s13059-014-0575-z 25582055 PMC4326501

[13] Okugawa Y Toiyama Y Shigeyasu K Yamamoto A Shigemori T Yin C. Enhanced AZIN1 RNA editing and overexpression of its regulatory enzyme ADAR1 are important prognostic biomarkers in gastric cancer. J Transl Med (2018) 16:366. doi: 10.1186/s12967-018-1740-z 30563560 PMC6299520

[14] Chigaev M Yu H Samuels DC Sheng Q Oyebamiji O Ness S. Genomic positional dissection of RNA editomes in tumor and normal samples. Front Genet (2019) 10:211. doi: 10.3389/fgene.2019.00211 30949194 PMC6435843

[15] Shigeyasu K Okugawa Y Toden S Miyoshi J Toiyama Y Nagasaka T. AZIN1 RNA editing confers cancer stemness and enhances oncogenic potential in colorectal cancer. JCI Insight (2018) 3(12):e99976. doi: 10.1172/jci.insight.99976 29925690 PMC6124399

[16] Qin YR Qiao JJ Chan TH Zhu YH Li FF Liu H. Adenosine-to-inosine RNA editing mediated by ADARs in esophageal squamous cell carcinoma. Cancer Res (2014) 74:840–51. doi: 10.1158/0008-5472.CAN-13-2545 24302582

[17] Chen L Li Y Lin CH Chan TH Chow RK Song Y. Recoding RNA editing of AZIN1 predisposes to hepatocellular carcinoma. Nat Med (2013) 19:209–16. doi: 10.1038/nm.3043 PMC3783260 23291631

[18] Chen W He W Cai H Hu B Zheng C Ke X. A-to-I RNA editing of BLCAP lost the inhibition to STAT3 activation in cervical cancer. Oncotarget (2017) 8:39417–29. doi: 10.18632/oncotarget.17034 PMC5503622 28455960

[19] Lo Giudice C Tangaro MA Pesole G Picardi E. Investigating RNA editing in deep transcriptome datasets with REDItools and REDIportal. Nat Protoc (2020) 15:1098–131. doi: 10.1038/s41596-019-0279-7 31996844

[20] Zhang F Lu Y Yan S Xing Q Tian W. SPRINT: an SNP-free toolkit for identifying RNA editing sites. Bioinformatics (2017) 33:3538–48. doi: 10.1093/bioinformatics/btx473 PMC5870768 29036410

[21] Peng J Sun BF Chen CY Zhou JY Chen YS Chen H. Single-cell RNA-seq highlights intra-tumoral heterogeneity and malignant progression in pancreatic ductal adenocarcinoma. Cell Res (2019) 29:725–38. doi: 10.1038/s41422-019-0195-y PMC6796938 31273297

[22] Islam S Kitagawa T Baron B Abiko Y Chiba I Kuramitsu Y. ITGA2, LAMB3, and LAMC2 may be the potential therapeutic targets in pancreatic ductal adenocarcinoma: an integrated bioinformatics analysis. Sci Rep (2021) 11:10563. doi: 10.1038/s41598-021-90077-x 34007003 PMC8131351

[23] Okada Y Takahashi N Takayama T Goel A. LAMC2 promotes cancer progression and gemcitabine resistance through modulation of EMT and ATP-binding cassette transporters in pancreatic ductal adenocarcinoma. Carcinogenesis (2021) 42:546–56. doi: 10.1093/carcin/bgab011 PMC8086766 33624791

[24] Zhou J Hui X Mao Y Fan L. Identification of novel genes associated with a poor prognosis in pancreatic ductal adenocarcinoma. via Bioinf analysis Biosci Rep (2019) 39(8):BSR20190625. doi: 10.1042/BSR20190625 PMC6680377 31311829

[25] Wang L Zhi X Zhu Y Zhang Q Wang W Li Z. MUC4-promoted neural invasion is mediated by the axon guidance factor netrin-1 in PDAC. Oncotarget (2015) 6:33805–22. doi: 10.18632/oncotarget.5668 PMC4741804 26393880

[26] Wu S Yang M Kim P Zhou X. ADeditome provides the genomic landscape of a-to-I RNA editing in alzheimer's disease. Brief Bioinform (2021) 22. doi: 10.1093/bib/bbaa384 PMC8424397 33401309

[27] Min HJ Lee Y Zhao XF Park YK Lee MK Lee JW. TMPRSS4 upregulates uPA gene expression through JNK signaling activation to induce cancer cell invasion. Cell Signal (2014) 26:398–408. doi: 10.1016/j.cellsig.2013.08.002 23978400

[28] Duan Y Dou S Luo S Zhang H Lu J. Adaptation of a-to-I RNA editing in drosophila. PLoS Genet (2017) 13:e1006648. doi: 10.1371/journal.pgen.1006648 28282384 PMC5365144

[29] Yu Y Zhou H Kong Y Pan B Chen L Wang H. The landscape of a-to-I RNA editome is shaped by both positive and purifying selection. PLoS Genet (2016) 12:e1006191. doi: 10.1371/journal.pgen.1006191 27467689 PMC4965139

[30] Giaimo BD Gagliani EK Kovall RA Borggrefe T. Transcription factor RBPJ as a molecular switch in regulating the notch response. Notch Signaling Embryology Cancer: Notch Signaling Cancer (2020) 1287:9–30. doi: 10.1007/978-3-030-55031-8_2 33034023

[31] Yalcin A Clem BF Imbert-Fernandez Y Ozcan SC Peker S O'Neal J. 6-Phosphofructo-2-kinase (PFKFB3) promotes cell cycle progression and suppresses apoptosis *via* Cdk1-mediated phosphorylation of p27. Cell Death Dis (2014) 5:e1337. doi: 10.1038/cddis.2014.292 25032860 PMC4123086

[32] Zhao H Wang Q Wang X Zhu H Zhang S Wang W. Correlation between RAB27B and p53 expression and overall survival in pancreatic cancer. Pancreas (2016) 45:204–10. doi: 10.1097/MPA.0000000000000453 PMC4714634 26418905

[33] Nishikura K. Functions and regulation of RNA editing by ADAR deaminases. Annu Rev Biochem (2010) 79:321–49. doi: 10.1146/annurev-biochem-060208-105251 PMC2953425 20192758

[34] Han L Diao L Yu S Xu X Li J Zhang R. The genomic landscape and clinical relevance of a-to-I RNA editing in human cancers. Cancer Cell (2015) 28:515–28. doi: 10.1016/j.ccell.2015.08.013 PMC4605878 26439496

[35] Bahn JH Lee JH Li G Greer C Peng G Xiao X. Accurate identification of a-to-I RNA editing in human by transcriptome sequencing. Genome Res (2012) 22:142–50. doi: 10.1101/gr.124107.111 PMC3246201 21960545

[36] Salameh A Lee AK Cardo-Vila M Nunes DN Efstathiou E Staquicini FI. PRUNE2 is a human prostate cancer suppressor regulated by the intronic long noncoding RNA PCA3. Proc Natl Acad Sci U.S.A. (2015) 112:8403–8. doi: 10.1073/pnas.1507882112 PMC4500257 26080435

[37] Lucas AL Frado LE Hwang C Kumar S Khanna LG Levinson EJ. BRCA1 and BRCA2 germline mutations are frequently demonstrated in both high-risk pancreatic cancer screening and pancreatic cancer cohorts. Cancer (2014) 120:1960–7. doi: 10.1002/cncr.28662 PMC5494829 24737347

[38] Russell R Perkhofer L Liebau S Lin Q Lechel A Feld FM. Loss of ATM accelerates pancreatic cancer formation and epithelial-mesenchymal transition. Nat Commun (2015) 6:7677. doi: 10.1038/ncomms8677 26220524 PMC4532798

[39] Dai C Rennhack JP Arnoff TE Thaker M Younger ST Doench JG. SMAD4 represses FOSL1 expression and pancreatic cancer metastatic colonization. Cell Rep (2021) 36:109443. doi: 10.1016/j.celrep.2021.109443 34320363 PMC8350598

[40] Zhang H Fu Q Shi X Pan Z Yang W Huang Z. Human a-to-I RNA editing SNP loci are enriched in GWAS signals for autoimmune diseases and under balancing selection. Genome Biol (2020) 21:288. doi: 10.1186/s13059-020-02205-x 33256812 PMC7702712

[41] Tang SJ Shen H An O Hong H Li J Song Y. Cis- and trans-regulations of pre-mRNA splicing by RNA editing enzymes influence cancer development. Nat Commun (2020) 11:799. doi: 10.1038/s41467-020-14621-5 32034135 PMC7005744

[42] Gu J Huang W Zhang J Wang X Tao T Yang L. TMPRSS4 promotes cell proliferation and inhibits apoptosis in pancreatic ductal adenocarcinoma by activating ERK1/2 signaling pathway. Front Oncol (2021) 11:628353. doi: 10.3389/fonc.2021.628353 33816264 PMC8012900

[43] Chang X Yang MF Fan W Wang LS Yao J Li ZS. Bioinformatic analysis suggests that three hub genes may be a vital prognostic biomarker in pancreatic ductal adenocarcinoma. J Comput Biol (2020) 27:1595–609. doi: 10.1089/cmb.2019.0367 32216644

[44] Wang Z Liu T Xue W Fang Y Chen X Xu L. ARNTL2 promotes pancreatic ductal adenocarcinoma progression through TGF/BETA pathway and is regulated by miR-26a-5p. Cell Death Dis (2020) 11:692. doi: 10.1038/s41419-020-02839-6 32826856 PMC7443143

[45] Ji T Ma K Chen L Cao T. PADI1 contributes to EMT in PAAD by activating the ERK1/2-p38 signaling pathway. J Gastrointest Oncol (2021) 12:1180–90. doi: 10.21037/jgo-21-283 PMC8261327 34295566

[46] Oidovsambuu O Nyamsuren G Liu S Goring W Engel W Adham IM. Adhesion protein VSIG1 is required for the proper differentiation of glandular gastric epithelia. PloS One (2011) 6:e25908. doi: 10.1371/journal.pone.0025908 21991385 PMC3186807

[47] Bernal C Silvano M Tapponnier Y Anand S Angulo C Ruiz i Altaba A. Functional pro-metastatic heterogeneity revealed by spiked-scRNAseq is shaped by cancer cell interactions and restricted by VSIG1. Cell Rep (2020) 33:108372. doi: 10.1016/j.celrep.2020.108372 33176137

[48] Kovecsi A Gurzu S Szentirmay Z Kovacs Z Bara TJ Jung I. Paradoxical expression pattern of the epithelial mesenchymal transition-related biomarkers CD44, SLUG, n-cadherin and VSIG1/Glycoprotein A34 in gastrointestinal stromal tumors. World J Gastrointest Oncol (2017) 9:436–43. doi: 10.4251/wjgo.v9.i11.436 PMC5700385 29204252

[49] Zhang T Wang Z Liu Y Huo Y Liu H Xu C. Plastin 1 drives metastasis of colorectal cancer through the IQGAP1/Rac1/ERK pathway. Cancer Sci (2020) 111:2861–71. doi: 10.1111/cas.14438 PMC7419044 32350953

[50] Zou B Liu X Gong Y Cai C Li P Xing S. A novel 12-marker panel of cancer-associated fibroblasts involved in progression of hepatocellular carcinoma. Cancer Manag Res (2018) 10:5303–11. doi: 10.2147/CMAR.S176152 PMC6225911 30464627

