# Supplementary figures and images for: Genome-wide identification of A-to-I RNA editing events provides the functional implications in PDAC
Source: Front Oncol. 2023 Feb 21;13:1092046. doi: 10.3389/fonc.2023.1092046 (PMC9990869; doi:10.3389/fonc.2023.1092046)

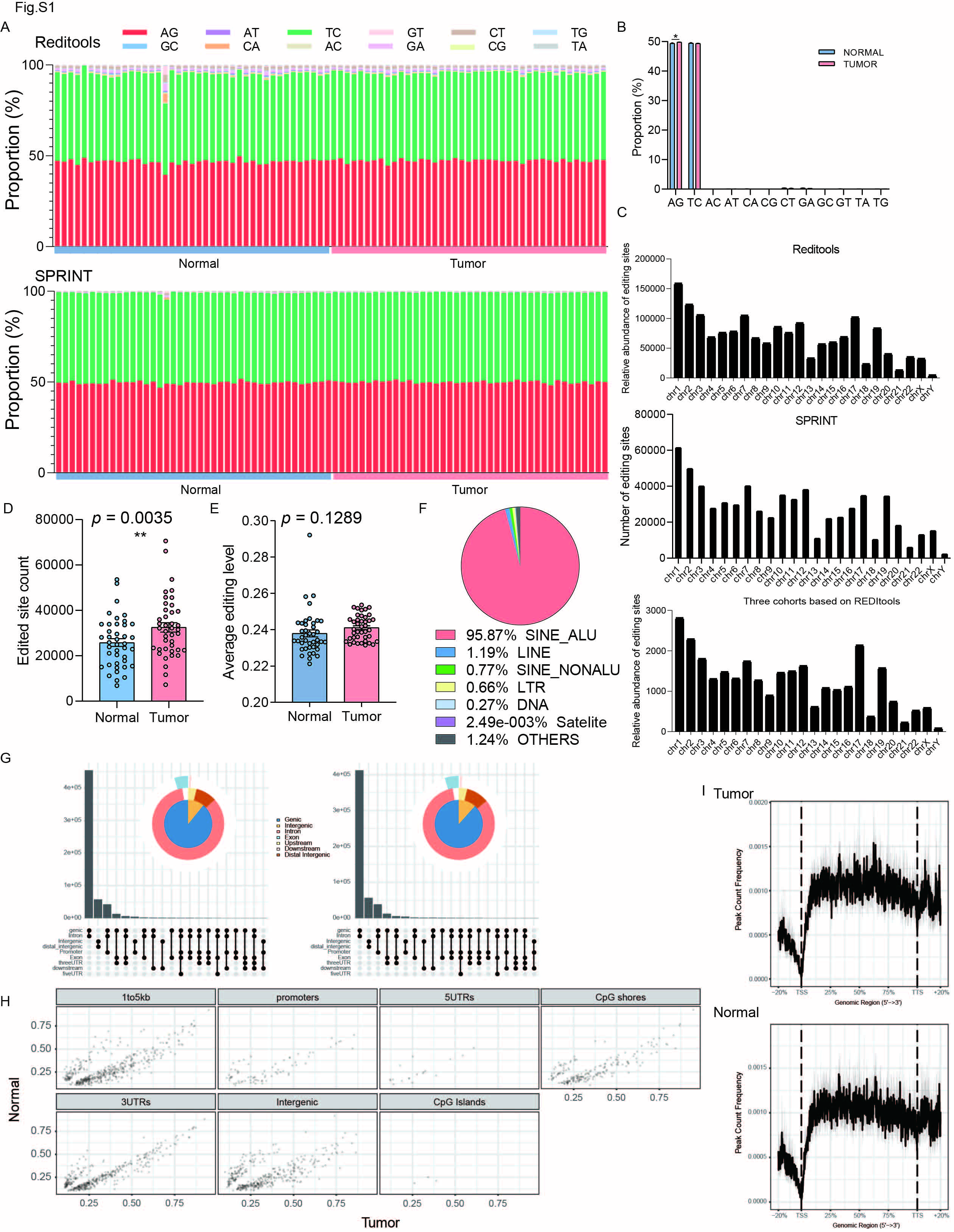

Supplement: Figure S1 — Comparison of the Overall A-to-I RNA Editing between Paired Tumor and Normal Samples. (A) The RNA variant distribution in individual sample. (B) The general RNA variant distribution among total samples. (C) The overall distribution of editing sites by chromosomes based on REDItools and SPRINT. (D) The change of editing count between normal and tumor samples based on SPRINT. (E) The change of average editing level between normal and tumor samples based on SPRINT. (F)The distribution of total A-to-I editing sites in repeat region. (G-H) The distribution of A-to-I DREs in different types of RNA regions. (I) The distance to TSS of editing sites between tumor and normal. [file Image_1.jpeg]

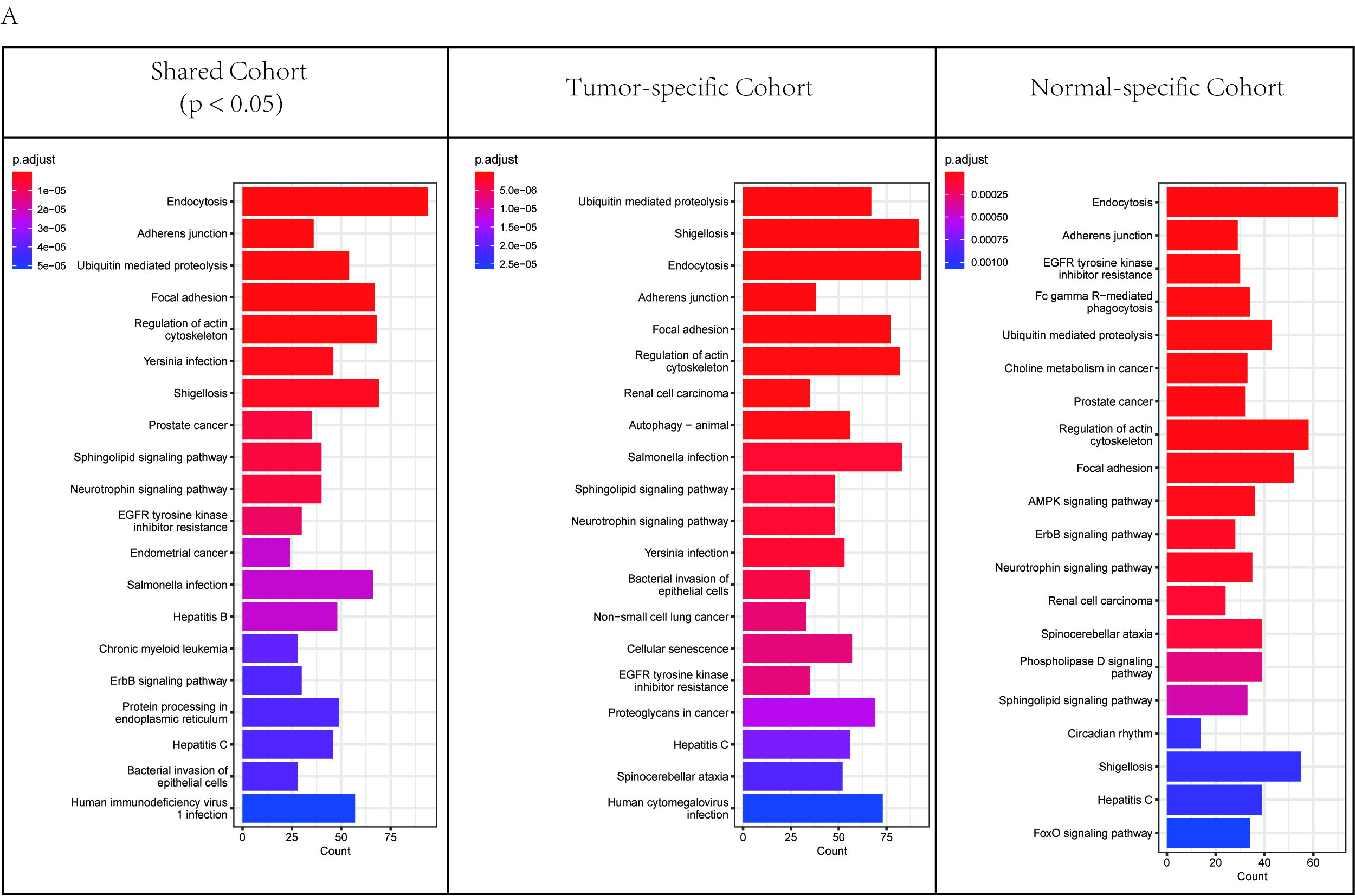

Supplement: Figure S2 — (A) The enriched pathway of the genes with A-to-I DREs. [file Image_2.tif]

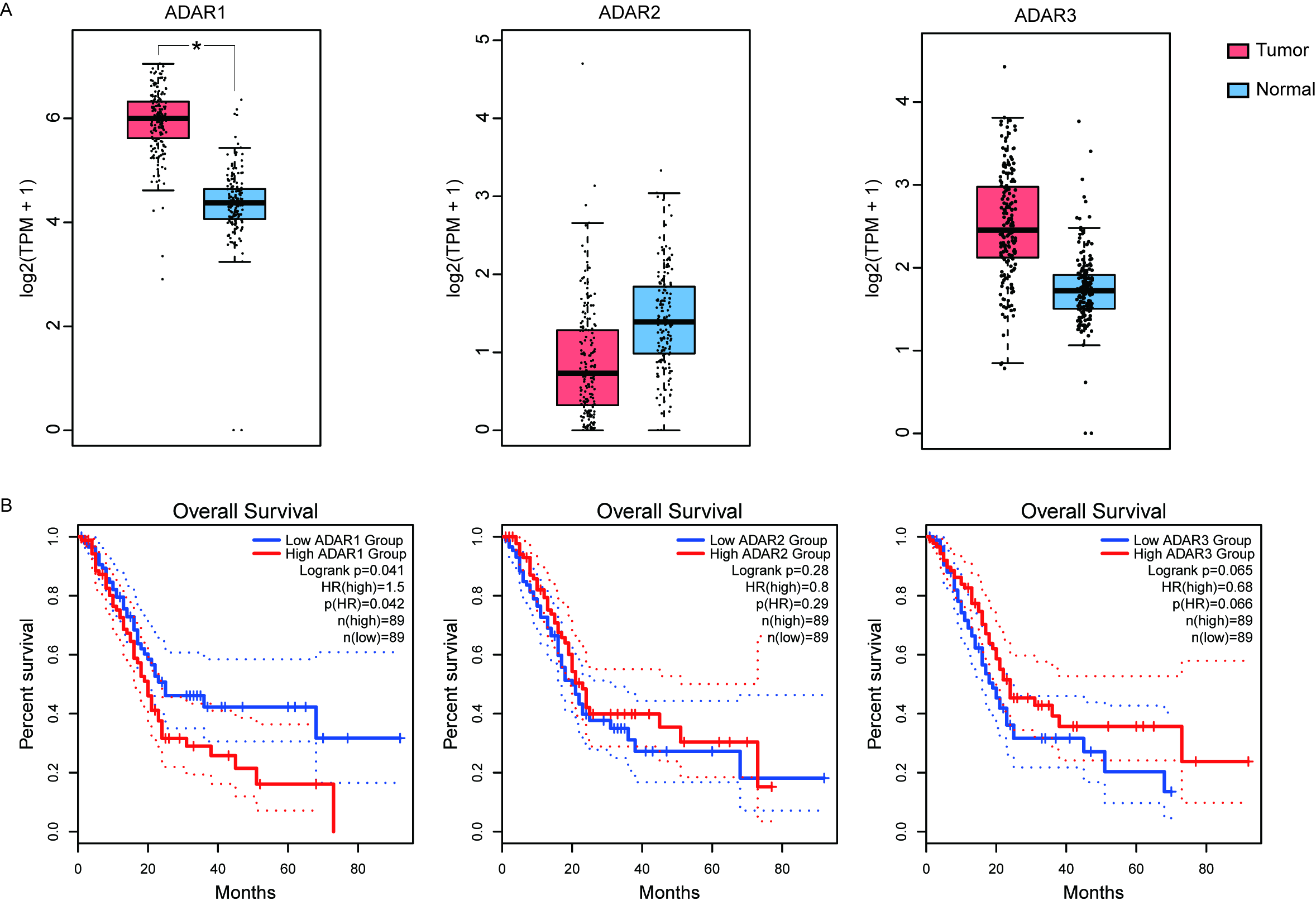

Supplement: Figure S3 — Effect of ADARs on pancreatic cancer. (A) The relative expression of ADARs in PDAC based on TCGA and GTEx data. (B) The correlation analysis with overall survivals based on ADARs expression in PDAC. [file Image_3.tif]
