# Supplementary material for: Genome-wide identification of A-to-I RNA editing events provides the functional implications in PDAC
Source: Front Oncol. 2023 Feb 21;13:1092046. doi: 10.3389/fonc.2023.1092046 (PMC9990869; doi:10.3389/fonc.2023.1092046)
Supplement: Supplementary file 4 [file DataSheet_1.docx]

**Table S1 List of edited exons in PDAC and normal tissues.**

| Group | Type | Chr | Position | *P* value | Tumor mean editing level | Normal mean editing level | Gene | Editing Effect |
| --- | --- | --- | --- | --- | --- | --- | --- | --- |
| shared cohort | synonymous | chr3 | 183384946 | 0.0227379 | 0.214 | 0.1125 | KLHL24 | p.T423T |
|  | synonymous | chr21 | 34923319 | 0.0147029 | 0.139615 | 0.116 | SON | p.L594L |
|  | nonsynonymous | chr7 | 39990527 | 0.0015095 | 0.340526 | 0.250571 | CDK13 | p.K96R |
|  | nonsynonymous | chr10 | 92662075 | 0.0372425 | 0.125714 | 0.336667 | RPP30 | p.K291R |
|  | nonsynonymous | chr15 | 65249466 | 0.0372116 | 0.115 | 0.160909 | ANKDD1A | p.Q503R |
| normal specific | synonymous | chr1 | 231990714 | NA | NA | 0.2 | DISC1 | p.S737S |
|  | synonymous | chr7 | 64259769 | NA | NA | 0.2125 | ZNF138 | p.P68P |
|  | nonsynonymous | chr3 | 58141801 | NA | NA | 0.1425 | FLNB | p.Q2296R |
|  | nonsynonymous | chr4 | 2721756 | NA | NA | 0.4075 | FAM193A | p.K1211E |
| tumor specific | synonymous | chr14 | 69061260 | NA | 0.252 | NA | RAD51B | p.P365P |
|  | nonsynonymous | chr4 | 2940028 | NA | 0.11 | NA | NOP14 | p.V778A |
|  | nonsynonymous | chr14 | 61545615 | NA | 0.164286 | NA | SLC38A6 | p.T460A |
|  | nonsynonymous | chr19 | 55525584 | NA | 0.431667 | NA | GP6 | p.W577R |

**Table S2 The characteristic of DREs among 41 overlapped genes**

| . | Chromosome | Position | Gene | RNA structure alteration by RNAfold | RNA structure alteration by Mxfold2 | Stable mean tumor editing level | stable mean normal editing level | p value (RNA editing) | p value (RNA expression) | Hyper/Hypo editing | Up/Down regulation | Known editing sites |
| --- | --- | --- | --- | --- | --- | --- | --- | --- | --- | --- | --- | --- |
| Normal-specific cohort | chr8 | 27365326 | EPHX2 | NA | NA | NA | 0.28 | NA | 0.00000444 | HYPO | DOWN | ed |
|  | chr8 | 27390576 | EPHX2 | loop_to_stem | small_loop_to_large _loop | NA | 0.22 | NA | 0.00000444 | HYPO | DOWN | ed |
|  | chr9 | 96013895 | WNK2 | loop_to_stem | loop_to_stem | NA | 0.4975 | NA | 0.014309398 | HYPO | DOWN | ed |
|  | chr16 | 8781297 | ABAT | large_loop_to_small _loop | loop_to_stem | NA | 0.418 | NA | 0.000021 | HYPO | DOWN | ed |
|  | chr16 | 8781325 | ABAT | loop_to_stem | NA | NA | 0.305 | NA | 0.000021 | HYPO | DOWN | ed |
|  | chr16 | 8816899 | ABAT | loop_to_stem | loop_to_stem | NA | 0.6375 | NA | 0.000021 | HYPO | DOWN | ed |
|  | chr19 | 8283912 | CERS4 | loop_to_stem | loop_to_stem | NA | 0.3525 | NA | 0.000107125 | HYPO | DOWN | ed |
|  | chr19 | 8284370 | CERS4 | small_loop_to_large _loop | NA | NA | 0.2075 | NA | 0.000107125 | HYPO | DOWN | ed |
|  | chr19 | 8284728 | CERS4 | NA | stem_to_loop | NA | 0.264 | NA | 0.000107125 | HYPO | DOWN | ed |
|  | chr19 | 8284746 | CERS4 | NA | NA | NA | 0.6225 | NA | 0.000107125 | HYPO | DOWN | ed |
|  | chr19 | 8288392 | CERS4 | NA | NA | NA | 0.256666667 | NA | 0.000107125 | HYPO | DOWN | ed |
|  | chr19 | 8291022 | CERS4 | NA | small_loop_to_large _loop | NA | 0.411666667 | NA | 0.000107125 | HYPO | DOWN | ed |
|  | chr19 | 8291961 | CERS4 | loop_to_stem | NA | NA | 0.2 | NA | 0.000107125 | HYPO | DOWN | ed |
|  | chr19 | 8293018 | CERS4 | NA | small_loop_to_large _loop | NA | 0.21 | NA | 0.000107125 | HYPO | DOWN | ed |
|  | chr19 | 8294089 | CERS4 | loop_to_stem | NA | NA | 0.316 | NA | 0.000107125 | HYPO | DOWN | ed |
|  | chr19 | 8295775 | CERS4 | loop_to_stem | loop_to_stem | NA | 0.17 | NA | 0.000107125 | HYPO | DOWN | ed |
|  | chr19 | 8295907 | CERS4 | loop_to_stem | loop_to_stem | NA | 0.2175 | NA | 0.000107125 | HYPO | DOWN | ed |
|  | chr19 | 8300667 | CERS4 | loop_to_stem | loop_to_stem | NA | 0.214285714 | NA | 0.000107125 | HYPO | DOWN | ed |
|  | chr19 | 8323611 | CERS4 | NA | loop_to_stem | NA | 0.165 | NA | 0.000107125 | HYPO | DOWN | ed |
| Shared cohort | chr2 | 161017966 | ITGB6 | stem_to_loop | large_loop_to_small _loop | 0.162857143 | 0.20125 | 0.049225882 | 0.000122465 | HYPO | UP | ed |
|  | chr2 | 161019898 | ITGB6 | stem_to_loop | NA | 0.134285714 | 0.215 | 0.017639996 | 0.000122465 | HYPO | UP | ed |
|  | chr2 | 161019939 | ITGB6 | stem_to_loop | NA | 0.197272727 | 0.132 | 0.046767357 | 0.000122465 | HYPER | UP | ed |
|  | chr2 | 161020031 | ITGB6 | stem_to_loop | stem_to_loop | 0.535333333 | 0.7475 | 0.045310905 | 0.000122465 | HYPO | UP | ed |
|  | chr2 | 224620812 | AP1S3 | stem_to_loop | stem_to_loop | 0.332692308 | 0.21 | 0.021498566 | 0.0000013 | HYPER | UP | ed |
|  | chr3 | 45183400 | CDCP1 | small_loop_to_large _loop | stem_to_loop | 0.3 | 0.1625 | 0.037201545 | 0.000584412 | HYPER | UP | - |
|  | chr5 | 52328945 | ITGA2 | NA | NA | 0.175 | 0.11 | 0.03985 | 0.000000222 | HYPER | Up | ed |
|  | chr11 | 117972247 | TMPRSS4 | loop_to_stem | large_loop_to_small _loop | 0.670476 | 0.81 | 0.040783 | 0.0000118 | HYPO | Up | ed |
|  | chr12 | 13051310 | GPRC5A | NA | loop_to_stem | 0.181579 | 0.126364 | 0.012011 | 0.000102756 | HYPER | Up | ed |
|  | chr12 | 13051394 | GPRC5A | loop_to_stem | large_loop_to_small _loop | 0.328889 | 0.247619 | 0.041525 | 0.000102756 | HYPER | Up | ed |
|  | chr12 | 13060705 | GPRC5A | NA | NA | 0.416154 | 0.195714 | 0.004305 | 0.000102756 | HYPER | Up | ed |
|  | chr12 | 27507232 | ARNTL2 | NA | loop_to_stem | 0.521429 | 0.696667 | 0.016569 | 0.0000963 | HYPO | Up | - |
|  | chr12 | 27574536 | ARNTL2 | NA | large_loop_to_small _loop | 0.570625 | 0.682 | 0.028399 | 0.0000963 | HYPO | Up | - |
|  | chr16 | 74799725 | FA2H | NA | NA | 0.772 | 0.342 | 0.01218578 | 0.016051072 | HYPER | UP | ed |
|  | chr16 | 74806973 | FA2H | small_loop_to_large _loop | loop_to_stem | 0.14 | 0.225 | 0.031949083 | 0.016051072 | HYPO | UP | ed |
|  | chr17 | 48164322 | ITGA3 | NA | NA | 0.328571 | 0.158 | 0.014024 | 0.00000408 | HYPER | Up | - |
|  | chrX | 107321660 | VSIG1 | NA | large_loop_to_small _loop | 0.220909 | 0.1375 | 0.049572 | 0.00000508 | HYPER | Up | - |
|  | chrX | 107321741 | VSIG1 | NA | NA | 0.215833 | 0.32 | 0.042935 | 0.00000508 | HYPO | Up | - |
| Tumor-specific cohort | chr1 | 17563164 | PADI1 | NA | NA | 0.225 | NA | NA | 0.00000149 | HYPER | UP | - |
|  | chr1 | 43406356 | SLC2A1 | NA | stem_to_loop | 0.184444444 | NA | NA | 0.00000436 | HYPER | UP | ed |
|  | chr1 | 46648113 | TSPAN1 | loop_to_stem | loop_to_stem | 0.45 | NA | NA | 0.0000163 | HYPER | UP | ed |
|  | chr1 | 46648555 | TSPAN1 | NA | NA | 0.375556 | NA | NA | 0.0000163 | HYPER | UP | ed |
|  | chr1 | 46648560 | TSPAN1 | NA | large_loop_to_small _loop | 0.244 | NA | NA | 0.0000163 | HYPER | UP | ed |
|  | chr1 | 183173215 | LAMC2 | loop_to_loop | NA | 0.235 | NA | NA | 0.0000016 | HYPER | UP | - |
|  | chr1 | 205366621 | LEMD1 | loop_to_stem | stem_to_loop | 0.28 | NA | NA | 0.000218343 | HYPER | UP | - |
|  | chr2 | 9007393 | MBOAT2 | NA | stem_to_loop | 0.2375 | NA | NA | 0.0000131 | HYPER | UP | ed |
|  | chr2 | 9007426 | MBOAT2 | NA | small_loop_to_large _loop | 0.2 | NA | NA | 0.0000131 | HYPER | UP | ed |
|  | chr2 | 75067320 | HK2 | loop_to_stem | NA | 0.24 | NA | NA | 0.00000508 | HYPER | UP | ed |
|  | chr2 | 160999790 | ITGB6 | small_loop_to_large _loop | stem_to_loop | 0.1125 | NA | NA | 0.000122465 | HYPER | UP | - |
|  | chr2 | 161017980 | ITGB6 | small_loop_to_large _loop | small_loop_to_large _loop | 0.1325 | NA | NA | 0.000122465 | HYPER | UP | ed |
|  | chr2 | 161019886 | ITGB6 | stem_to_loop | NA | 0.2 | NA | NA | 0.000122465 | HYPER | UP | - |
|  | chr2 | 161019924 | ITGB6 | NA | loop_to_stem | 0.161 | NA | NA | 0.000122465 | HYPER | UP | ed |
|  | chr2 | 161020136 | ITGB6 | NA | loop_to_stem | 0.212222222 | NA | NA | 0.000122465 | HYPER | UP | ed |
|  | chr2 | 161035481 | ITGB6 | small_loop_to_large _loop | NA | 0.2 | NA | NA | 0.000122465 | HYPER | UP | - |
|  | chr2 | 161036388 | ITGB6 | NA | NA | 0.1275 | NA | NA | 0.000122465 | HYPER | UP | - |
|  | chr2 | 224620295 | AP1S3 | NA | NA | 0.184 | NA | NA | 0.0000013 | HYPER | UP | ed |
|  | chr2 | 224620310 | AP1S3 | stem_to_loop | NA | 0.214 | NA | NA | 0.0000013 | HYPER | UP | ed |
|  | chr2 | 224620386 | AP1S3 | NA | NA | 0.188 | NA | NA | 0.0000013 | HYPER | UP | ed |
|  | chr2 | 224620418 | AP1S3 | NA | small_loop_to_large _loop | 0.204 | NA | NA | 0.0000013 | HYPER | UP | ed |
|  | chr2 | 224620880 | AP1S3 | NA | NA | 0.155 | NA | NA | 0.0000013 | HYPER | UP | ed |
|  | chr2 | 224621792 | AP1S3 | NA | loop_to_stem | 0.178 | NA | NA | 0.0000013 | HYPER | UP | ed |
|  | chr2 | 224621845 | AP1S3 | NA | small_loop_to_large _loop | 0.167 | NA | NA | 0.0000013 | HYPER | UP | ed |
|  | chr2 | 224677774 | AP1S3 | NA | loop_to_stem | 0.28 | NA | NA | 0.0000013 | HYPER | UP | ed |
|  | chr2 | 224692237 | AP1S3 | stem_to_loop | loop_to_stem | 0.395 | NA | NA | 0.0000013 | HYPER | UP | ed |
|  | chr2 | 224695986 | AP1S3 | small_loop_to_large _loop | NA | 0.185 | NA | NA | 0.0000013 | HYPER | UP | ed |
|  | chr3 | 45180294 | CDCP1 | NA | loop_to_stem | 0.1875 | NA | NA | 0.000584412 | HYPER | UP | - |
|  | chr3 | 45183366 | CDCP1 | stem_to_loop | NA | 0.182 | NA | NA | 0.000584412 | HYPER | UP | - |
|  | chr3 | 45183544 | CDCP1 | NA | NA | 0.168333333 | NA | NA | 0.000584412 | HYPER | UP | ed |
|  | chr3 | 49929819 | MST1R | NA | NA | 0.363333333 | NA | NA | 0.002574069 | HYPER | UP | ed |
|  | chr3 | 142318738 | PLS1 | stem_to_loop | small_loop_to_large _loop | 0.2575 | NA | NA | 0.000506066 | HYPER | UP | - |
|  | chr3 | 142354366 | PLS1 | loop_to_stem | loop_to_stem | 0.2475 | NA | NA | 0.000506066 | HYPER | UP | - |
|  | chr3 | 185228450 | LIPH | NA | NA | 0.4325 | NA | NA | 0.003058076 | HYPER | UP | ed |
|  | chr3 | 185233359 | LIPH | NA | NA | 0.376666667 | NA | NA | 0.003058076 | HYPER | UP | ed |
|  | chr3 | 185233368 | LIPH | NA | large_loop_to_small _loop | 0.2225 | NA | NA | 0.003058076 | HYPER | UP | - |
|  | chr3 | 185264369 | LIPH | stem_to_loop | loop_to_stem | 0.22 | NA | NA | 0.003058076 | HYPER | UP | ed |
|  | chr3 | 195475249 | MUC4 | NA | NA | 0.215 | NA | NA | 0.001468098 | HYPER | UP | - |
|  | chr5 | 52296941 | ITGA2 | NA | NA | 0.191667 | NA | NA | 0.000000222 | HYPER | UP | ed |
|  | chr5 | 52314773 | ITGA2 | NA | NA | 0.238 | NA | NA | 0.000000222 | HYPER | UP | ed |
|  | chr5 | 52315565 | ITGA2 | NA | loop_to_stem | 0.416667 | NA | NA | 0.000000222 | HYPER | UP | ed |
|  | chr5 | 52318522 | ITGA2 | large_loop_to_small _loop | NA | 0.268 | NA | NA | 0.000000222 | HYPER | UP | ed |
|  | chr5 | 52329451 | ITGA2 | large_loop_to_small _loop | NA | 0.15 | NA | NA | 0.000000222 | HYPER | UP | ed |
|  | chr7 | 23424450 | IGF2BP3 | NA | loop_to_stem | 0.22 | NA | NA | 0.015191767 | HYPER | UP | ed |
|  | chr8 | 120234961 | MAL2 | NA | small_loop_to_large _loop | 0.175 | NA | NA | 0.000766902 | HYPER | UP | - |
|  | chr8 | 120234983 | MAL2 | stem_to_loop | stem_to_loop | 0.1525 | NA | NA | 0.000766902 | HYPER | UP | - |
|  | chr8 | 120243389 | MAL2 | NA | stem_to_loop | 0.165 | NA | NA | 0.000766902 | HYPER | UP | - |
|  | chr11 | 69936387 | ANO1 | small_loop_to_large _loop | NA | 0.165 | NA | NA | 0.00000535 | HYPER | UP | ed |
|  | chr11 | 70026599 | ANO1 | small_loop_to_large _loop | NA | 0.168 | NA | NA | 0.00000535 | HYPER | UP | ed |
|  | chr11 | 70026608 | ANO1 | large_loop_to_small _loop | loop_to_stem | 0.18 | NA | NA | 0.00000535 | HYPER | UP | ed |
|  | chr11 | 117953142 | TMPRSS4 | loop_to_stem | loop_to_stem | 0.1625 | NA | NA | 0.0000118 | HYPER | UP | ed |
|  | chr11 | 117960660 | TMPRSS4 | NA | stem_to_loop | 0.224 | NA | NA | 0.0000118 | HYPER | UP | ed |
|  | chr11 | 117960818 | TMPRSS4 | NA | large_loop_to_small _loop | 0.23 | NA | NA | 0.0000118 | HYPER | UP | ed |
|  | chr11 | 117961372 | TMPRSS4 | NA | NA | 0.366 | NA | NA | 0.0000118 | HYPER | UP | ed |
|  | chr11 | 117967244 | TMPRSS4 | NA | NA | 0.116 | NA | NA | 0.0000118 | HYPER | UP | ed |
|  | chr11 | 117972314 | TMPRSS4 | NA | small_loop_to_large _loop | 0.168571 | NA | NA | 0.0000118 | HYPER | UP | ed |
|  | chr11 | 117973317 | TMPRSS4 | NA | small_loop_to_large _loop | 0.17 | NA | NA | 0.0000118 | HYPER | UP | ed |
|  | chr11 | 117986470 | TMPRSS4 | stem_to_loop | small_loop_to_large _loop | 0.3025 | NA | NA | 0.0000118 | HYPER | UP | ed |
|  | chr11 | 117992167 | TMPRSS4 | NA | small_loop_to_large _loop | 0.285 | NA | NA | 0.0000118 | HYPER | UP | ed |
|  | chr11 | 117992213 | TMPRSS4 | stem_to_loop | stem_to_loop | 0.215 | NA | NA | 0.0000118 | HYPER | UP | ed |
|  | chr12 | 13060589 | GPRC5A | NA | stem_to_loop | 0.13 | NA | NA | 0.000102756 | HYPER | UP | ed |
|  | chr12 | 13060745 | GPRC5A | loop_to_stem | NA | 0.1375 | NA | NA | 0.000102756 | HYPER | UP | ed |
|  | chr12 | 13062778 | GPRC5A | loop_to_stem | NA | 0.1525 | NA | NA | 0.000102756 | HYPER | UP | ed |
|  | chr12 | 27507097 | ARNTL2 | NA | loop_to_stem | 0.228571 | NA | NA | 0.0000963 | HYPER | UP | - |
|  | chr12 | 27507294 | ARNTL2 | NA | NA | 0.21 | NA | NA | 0.0000963 | HYPER | UP | - |
|  | chr12 | 27523719 | ARNTL2 | loop_to_stem | loop_to_stem | 0.1325 | NA | NA | 0.0000963 | HYPER | UP | - |
|  | chr12 | 71138941 | PTPRR | NA | NA | 0.1775 | NA | NA | 0.003028079 | HYPER | UP | - |
|  | chr12 | 71138983 | PTPRR | large_loop_to_small _loop | NA | 0.158 | NA | NA | 0.003028079 | HYPER | UP | - |
|  | chr14 | 67860282 | PLEK2 | NA | loop_to_stem | 0.1575 | NA | NA | 0.001661525 | HYPER | UP | - |
|  | chr14 | 67860417 | PLEK2 | loop_to_stem | small_loop_to_large _loop | 0.166 | NA | NA | 0.001661525 | HYPER | UP | - |
|  | chr14 | 67871316 | PLEK2 | NA | NA | 0.201666667 | NA | NA | 0.001661525 | HYPER | UP | - |
|  | chr14 | 67871334 | PLEK2 | NA | NA | 0.324285714 | NA | NA | 0.001661525 | HYPER | UP | - |
|  | chr15 | 80993121 | ABHD17C | NA | large_loop_to_small _loop | 0.183333333 | NA | NA | 0.0000127 | HYPER | UP | - |
|  | chr15 | 81000818 | ABHD17C | NA | NA | 0.2275 | NA | NA | 0.0000127 | HYPER | UP | - |
|  | chr15 | 81001572 | ABHD17C | large_loop_to_small _loop | NA | 0.1625 | NA | NA | 0.0000127 | HYPER | UP | ed |
|  | chr15 | 81006434 | ABHD17C | NA | NA | 0.19 | NA | NA | 0.0000127 | HYPER | UP | ed |
|  | chr15 | 81016424 | ABHD17C | loop_to_stem | NA | 0.2375 | NA | NA | 0.0000127 | HYPER | UP | ed |
|  | chr16 | 18999938 | TMC7 | NA | NA | 0.348 | NA | NA | 0.00000312 | HYPER | UP | ed |
|  | chr16 | 18999968 | TMC7 | NA | NA | 0.278 | NA | NA | 0.00000312 | HYPER | UP | ed |
|  | chr16 | 18999983 | TMC7 | NA | NA | 0.282 | NA | NA | 0.00000312 | HYPER | UP | ed |
|  | chr16 | 19034978 | TMC7 | NA | stem_to_loop | 0.4675 | NA | NA | 0.00000312 | HYPER | UP | ed |
|  | chr16 | 74755210 | FA2H | NA | large_loop_to_small _loop | 0.2775 | NA | NA | 0.016051072 | HYPER | UP | ed |
|  | chr16 | 74765863 | FA2H | NA | small_loop_to_large _loop | 0.1475 | NA | NA | 0.016051072 | HYPER | UP | ed |
|  | chr16 | 74780373 | FA2H | stem_to_loop | stem_to_loop | 0.192 | NA | NA | 0.016051072 | HYPER | UP | - |
|  | chr16 | 74792285 | FA2H | NA | loop_to_stem | 0.2925 | NA | NA | 0.016051072 | HYPER | UP | ed |
|  | chr16 | 74799670 | FA2H | stem_to_loop | stem_to_loop | 0.154 | NA | NA | 0.016051072 | HYPER | UP | ed |
|  | chr16 | 74805171 | FA2H | loop_to_stem | NA | 0.2 | NA | NA | 0.016051072 | HYPER | UP | - |
|  | chr16 | 74805174 | FA2H | NA | large_loop_to_small _loop | 0.176 | NA | NA | 0.016051072 | HYPER | UP | - |
|  | chr16 | 74806942 | FA2H | NA | loop_to_stem | 0.1425 | NA | NA | 0.016051072 | HYPER | UP | ed |
|  | chr17 | 34103281 | MMP28 | stem_to_loop | NA | 0.202 | NA | NA | 0.002613921 | HYPER | UP | ed |
|  | chr17 | 34103340 | MMP28 | small_loop_to_large _loop | stem_to_loop | 0.646666667 | NA | NA | 0.002613921 | HYPER | UP | ed |
|  | chr17 | 34104849 | MMP28 | small_loop_to_large _loop | NA | 0.214 | NA | NA | 0.002613921 | HYPER | UP | ed |
|  | chr17 | 34104860 | MMP28 | small_loop_to_large _loop | stem_to_loop | 0.3275 | NA | NA | 0.002613921 | HYPER | UP | ed |
|  | chr17 | 34104910 | MMP28 | stem_to_loop | NA | 0.1925 | NA | NA | 0.002613921 | HYPER | UP | - |
|  | chr17 | 34105025 | MMP28 | small_loop_to_large _loop | small_loop_to_large _loop | 0.3925 | NA | NA | 0.002613921 | HYPER | UP | ed |
|  | chr17 | 34112590 | MMP28 | NA | NA | 0.276 | NA | NA | 0.002613921 | HYPER | UP | - |
|  | chr17 | 34113171 | MMP28 | NA | NA | 0.16 | NA | NA | 0.002613921 | HYPER | UP | - |
|  | chr17 | 34113656 | MMP28 | stem_to_loop | stem_to_loop | 0.216 | NA | NA | 0.002613921 | HYPER | UP | ed |
|  | chr17 | 34115161 | MMP28 | stem_to_loop | small_loop_to_large _loop | 0.228 | NA | NA | 0.002613921 | HYPER | UP | ed |
|  | chr17 | 34117319 | MMP28 | stem_to_loop | stem_to_loop | 0.2175 | NA | NA | 0.002613921 | HYPER | UP | ed |
|  | chr17 | 34117735 | MMP28 | stem_to_loop | loop_to_stem | 0.143333333 | NA | NA | 0.002613921 | HYPER | UP | ed |
|  | chr17 | 34117774 | MMP28 | stem_to_loop | loop_to_stem | 0.165 | NA | NA | 0.002613921 | HYPER | UP | ed |
|  | chr17 | 34117825 | MMP28 | stem_to_loop | stem_to_loop | 0.184285714 | NA | NA | 0.002613921 | HYPER | UP | ed |
|  | chr17 | 34118420 | MMP28 | stem_to_loop | NA | 0.3 | NA | NA | 0.002613921 | HYPER | UP | ed |
|  | chr17 | 38648689 | TNS4 | small_loop_to_large _loop | stem_to_loop | 0.1325 | NA | NA | 0.006322398 | HYPER | UP | - |
|  | chr17 | 38655020 | TNS4 | NA | NA | 0.152 | NA | NA | 0.006322398 | HYPER | UP | ed |
|  | chr17 | 48143578 | ITGA3 | NA | large_loop_to_small _loop | 0.1925 | NA | NA | 0.00000408 | HYPER | UP | - |
|  | chr17 | 48147748 | ITGA3 | loop_to_stem | stem_to_loop | 0.498 | NA | NA | 0.00000408 | HYPER | UP | - |
|  | chr17 | 48159394 | ITGA3 | large_loop_to_small _loop | large_loop_to_small _loop | 0.155 | NA | NA | 0.00000408 | HYPER | UP | - |
|  | chr17 | 48159486 | ITGA3 | loop_to_stem | large_loop_to_small _loop | 0.1225 | NA | NA | 0.00000408 | HYPER | UP | - |
|  | chr17 | 48159682 | ITGA3 | loop_to_stem | loop_to_stem | 0.218 | NA | NA | 0.00000408 | HYPER | UP | - |
|  | chr17 | 48159866 | ITGA3 | NA | NA | 0.125 | NA | NA | 0.00000408 | HYPER | UP | - |
|  | chr17 | 48160961 | ITGA3 | NA | NA | 0.18 | NA | NA | 0.00000408 | HYPER | UP | - |
|  | chr17 | 48161368 | ITGA3 | large_loop_to_small _loop | NA | 0.1525 | NA | NA | 0.00000408 | HYPER | UP | - |
|  | chr17 | 48161504 | ITGA3 | stem_to_loop | small_loop_to_large _loop | 0.1325 | NA | NA | 0.00000408 | HYPER | UP | - |
|  | chr17 | 48162391 | ITGA3 | NA | NA | 0.1675 | NA | NA | 0.00000408 | HYPER | UP | - |
|  | chr17 | 48162445 | ITGA3 | NA | large_loop_to_small _loop | 0.134 | NA | NA | 0.00000408 | HYPER | UP | - |
|  | chr17 | 48162459 | ITGA3 | NA | NA | 0.1325 | NA | NA | 0.00000408 | HYPER | UP | - |
|  | chr17 | 48162921 | ITGA3 | NA | NA | 0.215714 | NA | NA | 0.00000408 | HYPER | UP | - |
|  | chr17 | 48163798 | ITGA3 | loop_to_stem | NA | 0.17 | NA | NA | 0.00000408 | HYPER | UP | - |
|  | chr17 | 48164296 | ITGA3 | loop_to_stem | loop_to_stem | 0.204 | NA | NA | 0.00000408 | HYPER | UP | - |
|  | chr18 | 52516873 | RAB27B | NA | NA | 0.122 | NA | NA | 0.00000153 | HYPER | UP | ed |
|  | chr18 | 52543236 | RAB27B | loop_to_stem | loop_to_stem | 0.475 | NA | NA | 0.00000153 | HYPER | UP | ed |
|  | chr18 | 52543241 | RAB27B | large_loop_to_small _loop | stem_to_loop | 0.435 | NA | NA | 0.00000153 | HYPER | UP | ed |
|  | chr18 | 52543397 | RAB27B | NA | NA | 0.423333 | NA | NA | 0.00000153 | HYPER | UP | ed |
|  | chr19 | 17920478 | B3GNT3 | loop_to_stem | small_loop_to_large _loop | 0.345 | NA | NA | 0.00000416 | HYPER | UP | - |
|  | chr19 | 17920492 | B3GNT3 | NA | NA | 0.245 | NA | NA | 0.00000416 | HYPER | UP | - |
|  | chr19 | 17921044 | B3GNT3 | NA | loop_to_stem | 0.145 | NA | NA | 0.00000416 | HYPER | UP | - |
|  | chr19 | 17921169 | B3GNT3 | small_loop_to_large _loop | NA | 0.156 | NA | NA | 0.00000416 | HYPER | UP | - |
|  | chr19 | 17921938 | B3GNT3 | NA | NA | 0.2275 | NA | NA | 0.00000416 | HYPER | UP | - |
|  | chr19 | 46654938 | IGFL2 | loop_to_stem | NA | 0.2725 | NA | NA | 0.001060612 | HYPER | UP | ed |
|  | chr19 | 55861237 | COX6B2 | stem_to_loop | NA | 0.12 | NA | NA | 0.00503728 | HYPER | UP | - |
|  | chr19 | 55861251 | COX6B2 | stem_to_loop | NA | 0.175 | NA | NA | 0.00503728 | HYPER | UP | - |
|  | chr20 | 1296635 | SDCBP2 | stem_to_loop | loop_to_stem | 0.225 | NA | NA | 0.003928931 | HYPER | UP | ed |
|  | chr20 | 1296647 | SDCBP2 | NA | small_loop_to_large _loop | 0.18 | NA | NA | 0.003928931 | HYPER | UP | ed |
|  | chr20 | 1297158 | SDCBP2 | NA | NA | 0.5 | NA | NA | 0.003928931 | HYPER | UP | ed |
|  | chr20 | 1297289 | SDCBP2 | large_loop_to_small _loop | NA | 0.401666667 | NA | NA | 0.003928931 | HYPER | UP | ed |
|  | chr20 | 1297307 | SDCBP2 | NA | NA | 0.23125 | NA | NA | 0.003928931 | HYPER | UP | ed |
|  | chr20 | 6073138 | FERMT1 | NA | NA | 0.39 | NA | NA | 0.0000254 | HYPER | UP | ed |
|  | chrX | 115579036 | SLC6A14 | NA | large_loop_to_small _loop | 0.1725 | NA | NA | 0.000327006 | HYPER | UP | ed |

**Table S3 List of alternative splicing events containing DREs.**

| Chromosome | Start | End | Type | Gene | Number of covered DREs |
| --- | --- | --- | --- | --- | --- |
| chr1 | 113134225 | 113153462 | MXE | ST7L | 1 |
| chr1 | 113143470 | 113153462 | MXE | ST7L | 1 |
| chr1 | 12640694 | 12677158 | MXE | DHRS3 | 1 |
| chr1 | 150634289 | 150667326 | SE | GOLPH3L | 5 |
| chr1 | 150825282 | 150830824 | MXE | ARNT | 2 |
| chr1 | 150974200 | 150979333 | SE | FAM63A | 1 |
| chr1 | 151196614 | 151199876 | SE | PIP5K1A | 2 |
| chr1 | 15480228 | 15545842 | SE | TMEM51 | 20 |
| chr1 | 155282186 | 155287731 | MXE | FDPS | 2 |
| chr1 | 155679563 | 155691409 | SE | DAP3 | 1 |
| chr1 | 155691409 | 155697398 | MXE | DAP3 | 1 |
| chr1 | 155691409 | 155701146 | MXE | DAP3 | 1 |
| chr1 | 156238080 | 156243241 | SE | SMG5 | 1 |
| chr1 | 15959933 | 15970145 | SE | DDI2 | 3 |
| chr1 | 161476497 | 161479609 | A5SS | FCGR2A | 2 |
| chr1 | 16199310 | 16235976 | SE | SPEN | 5 |
| chr1 | 16641930 | 16678749 | MXE | FBXO42 | 9 |
| chr1 | 167973770 | 168012378 | SE | DCAF6 | 1 |
| chr1 | 169206860 | 169268001 | SE | NME7 | 1 |
| chr1 | 1696885 | 1711343 | MXE | NADK | 1 |
| chr1 | 174844683 | 174926686 | SE | RABGAP1L | 10 |
| chr1 | 1749314 | 1756835 | MXE | GNB1 | 5 |
| chr1 | 176118210 | 176145045 | MXE | RFWD2 | 1 |
| chr1 | 176153828 | 176175707 | MXE | RFWD2 | 1 |
| chr1 | 183155372 | 183184723 | SE | LAMC2 | 1 |
| chr1 | 183441634 | 183471526 | SE | SMG7 | 3 |
| chr1 | 201958031 | 201965391 | SE | RNPEP | 6 |
| chr1 | 201965537 | 201969029 | MXE | RNPEP | 1 |
| chr1 | 205698749 | 205719084 | MXE | NUCKS1 | 9 |
| chr1 | 207940357 | 207943707 | RI | CD46 | 6 |
| chr1 | 21415630 | 21502851 | SE | EIF4G3 | 32 |
| chr1 | 227400836 | 227504748 | SE | CDC42BPA | 9 |
| chr1 | 230339036 | 230371759 | MXE | GALNT2 | 2 |
| chr1 | 231376955 | 231396399 | SE | GNPAT | 3 |
| chr1 | 233092092 | 233114228 | SE | NTPCR | 1 |
| chr1 | 235909767 | 235914662 | SE | LYST | 1 |
| chr1 | 246720023 | 246727647 | MXE | TFB2M | 1 |
| chr1 | 246899357 | 246903473 | MXE | SCCPDH | 1 |
| chr1 | 29213722 | 29313942 | A3SS | EPB41 | 2 |
| chr1 | 29486886 | 29508402 | SE | SRSF4 | 5 |
| chr1 | 31418330 | 31422973 | MXE | PUM1 | 1 |
| chr1 | 31501642 | 31532424 | A5SS | PUM1 | 2 |
| chr1 | 31501642 | 31532424 | SE | PUM1 | 2 |
| chr1 | 33479965 | 33487291 | SE | AK2 | 2 |
| chr1 | 38042028 | 38047937 | SE | GNL2 | 2 |
| chr1 | 39838268 | 39844874 | MXE | MACF1 | 3 |
| chr1 | 46016214 | 46032360 | SE | AKR1A1 | 1 |
| chr1 | 46072153 | 46078920 | SE | NASP | 10 |
| chr1 | 46072263 | 46078840 | MXE | NASP | 10 |
| chr1 | 47048899 | 47059878 | SE | MKNK1 | 3 |
| chr1 | 62175000 | 62190739 | SE | TM2D1 | 4 |
| chr1 | 78107340 | 78148269 | MXE | ZZZ3 | 11 |
| chr1 | 78444915 | 78479039 | SE | DNAJB4 | 7 |
| chr1 | 9795942 | 9801314 | SE | CLSTN1 | 1 |
| chr10 | 114724383 | 114799783 | MXE | TCF7L2 | 5 |
| chr10 | 114799885 | 114900942 | MXE | TCF7L2 | 5 |
| chr10 | 116232735 | 116247752 | SE | ABLIM1 | 1 |
| chr10 | 116232891 | 116247716 | MXE | ABLIM1 | 1 |
| chr10 | 116233715 | 116247716 | MXE | ABLIM1 | 1 |
| chr10 | 129847793 | 129859279 | SE | PTPRE | 3 |
| chr10 | 22218070 | 22292141 | MXE | DNAJC1 | 2 |
| chr10 | 24762156 | 24784125 | SE | KIAA1217 | 4 |
| chr10 | 24790474 | 24802187 | MXE | KIAA1217 | 1 |
| chr10 | 24821998 | 24834032 | SE | KIAA1217 | 9 |
| chr10 | 27044583 | 27054247 | SE | ABI1 | 5 |
| chr10 | 29813400 | 29818773 | SE | SVIL | 2 |
| chr10 | 32132388 | 32150586 | SE | ARHGAP12 | 1 |
| chr10 | 32141525 | 32150322 | MXE | ARHGAP12 | 1 |
| chr10 | 32150586 | 32197099 | MXE | ARHGAP12 | 3 |
| chr10 | 33466419 | 33474652 | SE | NRP1 | 2 |
| chr10 | 34648998 | 34663930 | SE | PARD3 | 3 |
| chr10 | 43892038 | 43904174 | MXE | HNRNPF | 1 |
| chr10 | 5726800 | 5762574 | SE | FAM208B | 7 |
| chr10 | 5756170 | 5762506 | MXE | FAM208B | 3 |
| chr10 | 5932242 | 5948595 | SE | FBXO18 | 4 |
| chr10 | 69651241 | 69666694 | SE | SIRT1 | 3 |
| chr10 | 69726439 | 69748592 | SE | HERC4 | 1 |
| chr10 | 69804160 | 69832943 | SE | HERC4 | 13 |
| chr10 | 70276507 | 70287173 | SE | SLC25A16 | 8 |
| chr10 | 70276507 | 70287195 | SE | SLC25A16 | 8 |
| chr10 | 73975805 | 73990198 | SE | ANAPC16 | 13 |
| chr10 | 74806859 | 74810810 | MXE | P4HA1 | 4 |
| chr10 | 82219057 | 82249070 | SE | TSPAN14 | 4 |
| chr10 | 95148786 | 95155981 | SE | MYOF | 3 |
| chr10 | 95169329 | 95191273 | SE | MYOF | 1 |
| chr10 | 97174250 | 97181830 | SE | SORBS1 | 1 |
| chr10 | 97174619 | 97181717 | MXE | SORBS1 | 1 |
| chr10 | 97893312 | 97915804 | SE | ZNF518A | 8 |
| chr11 | 107969132 | 107977579 | SE | CUL5 | 4 |
| chr11 | 108056129 | 108058906 | SE | NPAT | 5 |
| chr11 | 117014982 | 117030739 | SE | PAFAH1B2 | 3 |
| chr11 | 117110576 | 117115160 | MXE | RNF214 | 2 |
| chr11 | 118633920 | 118639060 | SE | DDX6 | 4 |
| chr11 | 124624525 | 124632186 | SE | ESAM | 2 |
| chr11 | 13443188 | 13484728 | SE | BTBD10 | 1 |
| chr11 | 18587893 | 18596959 | SE | UEVLD | 1 |
| chr11 | 33370833 | 33373153 | MXE | HIPK3 | 1 |
| chr11 | 35211381 | 35236408 | SE | CD44 | 1 |
| chr11 | 35211381 | 35236461 | SE | CD44 | 1 |
| chr11 | 46515673 | 46564531 | SE | AMBRA1 | 1 |
| chr11 | 47238408 | 47259552 | SE | DDB2 | 11 |
| chr11 | 57529233 | 57561553 | SE | CTNND1 | 24 |
| chr11 | 57529268 | 57559145 | SE | CTNND1 | 24 |
| chr11 | 57529268 | 57561553 | SE | CTNND1 | 24 |
| chr11 | 57529268 | 57563201 | SE | CTNND1 | 24 |
| chr11 | 57529272 | 57561553 | SE | CTNND1 | 24 |
| chr11 | 57529540 | 57561481 | A5SS | CTNND1 | 24 |
| chr11 | 57529540 | 57563048 | A5SS | CTNND1 | 24 |
| chr11 | 57529591 | 57558856 | A5SS | CTNND1 | 24 |
| chr11 | 57529591 | 57561481 | A5SS | CTNND1 | 24 |
| chr11 | 57529591 | 57563048 | A5SS | CTNND1 | 24 |
| chr11 | 58910555 | 58916426 | SE | FAM111A | 2 |
| chr11 | 67942650 | 67953247 | MXE | SUV420H1 | 4 |
| chr11 | 68207340 | 68214001 | SE | LRP5 | 1 |
| chr11 | 72470278 | 72492339 | A5SS | STARD10 | 1 |
| chr11 | 72712034 | 72794785 | SE | FCHSD2 | 12 |
| chr11 | 74559159 | 74617501 | SE | XRRA1 | 3 |
| chr11 | 85742510 | 85780853 | SE | PICALM | 7 |
| chr11 | 85742510 | 85780924 | SE | PICALM | 7 |
| chr11 | 88053978 | 88068250 | SE | CTSC | 11 |
| chr11 | 93466515 | 93467826 | A3SS | TAF1D | 2 |
| chr12 | 10365495 | 10370740 | SE | GABARAPL1 | 1 |
| chr12 | 105540819 | 105546229 | SE | KIAA1033 | 1 |
| chr12 | 112204751 | 112219826 | SE | ALDH2 | 1 |
| chr12 | 122967181 | 122974010 | SE | ZCCHC8 | 2 |
| chr12 | 12639961 | 12672934 | SE | DUSP16 | 4 |
| chr12 | 132416059 | 132426528 | SE | PUS1 | 4 |
| chr12 | 15263860 | 15370537 | SE | RERG | 2 |
| chr12 | 27450642 | 27455121 | SE | STK38L | 1 |
| chr12 | 2986364 | 2998437 | SE | RHNO1 | 1 |
| chr12 | 30872012 | 30873849 | RI | CAPRIN2 | 1 |
| chr12 | 31451010 | 31479070 | SE | FAM60A | 1 |
| chr12 | 46357971 | 46384135 | MXE | SCAF11 | 2 |
| chr12 | 47610380 | 47629021 | SE | PCED1B | 9 |
| chr12 | 47610380 | 47629348 | SE | PCED1B | 9 |
| chr12 | 49089541 | 49091966 | SE | CCNT1 | 2 |
| chr12 | 49893852 | 49908396 | SE | SPATS2 | 2 |
| chr12 | 49893988 | 49906644 | MXE | SPATS2 | 2 |
| chr12 | 50537734 | 50561086 | SE | CERS5 | 7 |
| chr12 | 50594559 | 50598483 | SE | LIMA1 | 1 |
| chr12 | 50594667 | 50598334 | MXE | LIMA1 | 1 |
| chr12 | 51442083 | 51450211 | SE | LETMD1 | 2 |
| chr12 | 51446029 | 51447560 | A5SS | LETMD1 | 1 |
| chr12 | 53416276 | 53421972 | SE | EIF4B | 3 |
| chr12 | 53689074 | 53691934 | SE | PFDN5 | 1 |
| chr12 | 69207333 | 69214154 | SE | MDM2 | 1 |
| chr12 | 69265563 | 69326620 | SE | CPM | 1 |
| chr12 | 70195388 | 70206813 | SE | RAB3IP | 1 |
| chr12 | 70195501 | 70206557 | A3SS | RAB3IP | 1 |
| chr12 | 72094611 | 72097836 | RI | TMEM19 | 1 |
| chr12 | 72094805 | 72097756 | A5SS | TMEM19 | 1 |
| chr12 | 89813494 | 89853495 | SE | POC1B | 4 |
| chr12 | 95645715 | 95651015 | SE | VEZT | 1 |
| chr12 | 95656857 | 95660132 | MXE | VEZT | 2 |
| chr12 | 96077487 | 96104218 | MXE | NTN4 | 2 |
| chr12 | 980514 | 987377 | MXE | WNK1 | 1 |
| chr13 | 100888095 | 100915085 | SE | PCCA | 3 |
| chr13 | 111806060 | 111870147 | SE | ARHGEF7 | 2 |
| chr13 | 111806060 | 111870227 | SE | ARHGEF7 | 2 |
| chr13 | 111857635 | 111870147 | SE | ARHGEF7 | 2 |
| chr13 | 20409616 | 20425588 | SE | ZMYM5 | 6 |
| chr13 | 21987790 | 22033447 | SE | ZDHHC20 | 3 |
| chr13 | 24796956 | 24826000 | SE | SPATA13 | 1 |
| chr13 | 26927876 | 26959479 | SE | CDK8 | 5 |
| chr13 | 41556418 | 41593363 | MXE | ELF1 | 10 |
| chr13 | 50601268 | 50642299 | SE | DLEU2 | 16 |
| chr13 | 52991191 | 52997774 | SE | VPS36 | 6 |
| chr13 | 96624833 | 96665665 | SE | UGGT2 | 4 |
| chr14 | 103957875 | 103969589 | SE | MARK3 | 5 |
| chr14 | 103958113 | 103969408 | SE | MARK3 | 5 |
| chr14 | 103966537 | 103969218 | MXE | MARK3 | 1 |
| chr14 | 21702111 | 21731495 | SE | HNRNPC | 20 |
| chr14 | 21731469 | 21737565 | SE | HNRNPC | 3 |
| chr14 | 21731469 | 21737584 | A3SS | HNRNPC | 3 |
| chr14 | 21731469 | 21737601 | A3SS | HNRNPC | 3 |
| chr14 | 23299395 | 23303554 | SE | MRPL52 | 3 |
| chr14 | 23299395 | 23303555 | SE | MRPL52 | 3 |
| chr14 | 23375236 | 23388338 | SE | RBM23 | 2 |
| chr14 | 23419619 | 23420763 | MXE | HAUS4 | 1 |
| chr14 | 31381212 | 31404510 | SE | STRN3 | 9 |
| chr14 | 39788412 | 39796226 | SE | CTAGE5 | 5 |
| chr14 | 39866872 | 39901704 | SE | FBXO33 | 3 |
| chr14 | 50272738 | 50281525 | SE | NEMF | 2 |
| chr14 | 53130565 | 53133092 | MXE | ERO1L | 1 |
| chr14 | 53149126 | 53162085 | MXE | ERO1L | 5 |
| chr14 | 60716158 | 60750255 | SE | PPM1A | 2 |
| chr14 | 60716377 | 60750255 | SE | PPM1A | 2 |
| chr14 | 68126447 | 68141548 | SE | VTI1B | 2 |
| chr14 | 70842392 | 70883762 | SE | SYNJ2BP | 2 |
| chr14 | 71996028 | 72054411 | SE | SIPA1L1 | 4 |
| chr14 | 73741923 | 73749213 | SE | NUMB | 2 |
| chr14 | 75248036 | 75269379 | SE | YLPM1 | 2 |
| chr14 | 91666100 | 91681900 | SE | C14orf159 | 1 |
| chr14 | 91666100 | 91682137 | SE | C14orf159 | 1 |
| chr14 | 93170153 | 93176217 | SE | LGMN | 2 |
| chr14 | 93730130 | 93799367 | SE | BTBD7 | 10 |
| chr15 | 100106191 | 100173378 | SE | MEF2A | 6 |
| chr15 | 33357275 | 33380999 | MXE | FMN1 | 1 |
| chr15 | 33381175 | 33445248 | MXE | FMN1 | 3 |
| chr15 | 34567590 | 34628610 | MXE | SLC12A6 | 2 |
| chr15 | 52203349 | 52208211 | MXE | TMOD3 | 9 |
| chr15 | 52571705 | 52587853 | SE | MYO5C | 1 |
| chr15 | 72231192 | 72252296 | SE | MYO9A | 2 |
| chr15 | 77578764 | 77712436 | SE | PEAK1 | 14 |
| chr15 | 89182825 | 89193465 | A3SS | ISG20 | 3 |
| chr16 | 11791980 | 11815526 | SE | TXNDC11 | 8 |
| chr16 | 15788021 | 15820210 | SE | NDE1 | 3 |
| chr16 | 15797028 | 15808938 | SE | MYH11 | 1 |
| chr16 | 16103632 | 16127027 | SE | ABCC1 | 3 |
| chr16 | 169124 | 180590 | SE | NPRL3 | 2 |
| chr16 | 19745019 | 19800244 | SE | IQCK | 1 |
| chr16 | 19745149 | 19800159 | MXE | IQCK | 1 |
| chr16 | 21430810 | 21436457 | A3SS | NPIPB3 | 1 |
| chr16 | 23507099 | 23521641 | MXE | GGA2 | 4 |
| chr16 | 270746 | 277240 | MXE | LUC7L | 2 |
| chr16 | 4526340 | 4555611 | SE | HMOX2 | 1 |
| chr16 | 4526412 | 4555611 | SE | HMOX2 | 1 |
| chr16 | 57207684 | 57219951 | SE | FAM192A | 5 |
| chr16 | 66586469 | 66599975 | SE | CKLF | 3 |
| chr16 | 66586471 | 66599972 | SE | CKLF | 3 |
| chr16 | 66592092 | 66599972 | SE | CKLF | 1 |
| chr16 | 70578436 | 70582272 | MXE | SF3B3 | 1 |
| chr16 | 71823181 | 71842735 | A5SS | AP1G1 | 2 |
| chr16 | 74709515 | 74712878 | SE | MLKL | 4 |
| chr16 | 89753077 | 89756965 | SE | CDK10 | 3 |
| chr16 | 89755732 | 89756960 | MXE | CDK10 | 1 |
| chr17 | 1268152 | 1303445 | SE | YWHAE | 11 |
| chr17 | 15943754 | 15950407 | A5SS | NCOR1 | 1 |
| chr17 | 17723403 | 17740301 | SE | SREBF1 | 10 |
| chr17 | 17754206 | 17764865 | SE | TOM1L2 | 4 |
| chr17 | 17754206 | 17769733 | SE | TOM1L2 | 4 |
| chr17 | 26989215 | 27000528 | SE | SUPT6H | 6 |
| chr17 | 28120955 | 28256955 | MXE | SSH2 | 8 |
| chr17 | 28443798 | 28499616 | SE | NSRP1 | 21 |
| chr17 | 28444171 | 28499616 | SE | NSRP1 | 21 |
| chr17 | 30536368 | 30552132 | SE | RHOT1 | 1 |
| chr17 | 33333008 | 33343599 | SE | RFFL | 3 |
| chr17 | 33348800 | 33390594 | MXE | RFFL | 4 |
| chr17 | 33353580 | 33390594 | MXE | RFFL | 4 |
| chr17 | 33353580 | 33416123 | MXE | RFFL | 10 |
| chr17 | 35646430 | 35687112 | MXE | ACACA | 3 |
| chr17 | 36912135 | 36918758 | SE | PSMB3 | 3 |
| chr17 | 40152499 | 40169441 | SE | DNAJC7 | 1 |
| chr17 | 42285231 | 42286790 | SE | UBTF | 1 |
| chr17 | 44249598 | 44301036 | MXE | KANSL1 | 2 |
| chr17 | 47700094 | 47755365 | SE | SPOP | 1 |
| chr17 | 47700094 | 47755408 | SE | SPOP | 1 |
| chr17 | 49157065 | 49197714 | MXE | SPAG9 | 15 |
| chr17 | 55729522 | 55752278 | MXE | MSI2 | 1 |
| chr17 | 60111147 | 60130066 | SE | MED13 | 18 |
| chr17 | 67246612 | 67249808 | SE | ABCA5 | 2 |
| chr17 | 70845772 | 70944014 | A5SS | SLC39A11 | 3 |
| chr17 | 73664595 | 73689562 | SE | SAP30BP | 1 |
| chr17 | 75398785 | 75478225 | MXE | SEPT9 | 6 |
| chr17 | 76705814 | 76732773 | MXE | CYTH1 | 6 |
| chr17 | 76705814 | 76778283 | MXE | CYTH1 | 18 |
| chr17 | 78247039 | 78262162 | SE | RNF213 | 4 |
| chr17 | 79220684 | 79225412 | A3SS | SLC38A10 | 1 |
| chr18 | 13621270 | 13645125 | MXE | LDLRAD4 | 1 |
| chr18 | 21124923 | 21134948 | SE | NPC1 | 1 |
| chr18 | 2655736 | 2667030 | SE | SMCHD1 | 3 |
| chr18 | 2772247 | 2777913 | SE | SMCHD1 | 2 |
| chr18 | 357399 | 500722 | SE | COLEC12 | 1 |
| chr18 | 43595868 | 43620013 | SE | PSTPIP2 | 1 |
| chr18 | 51731527 | 51750387 | MXE | MBD2 | 1 |
| chr18 | 52495429 | 52546685 | SE | RAB27B | 4 |
| chr18 | 55342254 | 55351267 | MXE | ATP8B1 | 1 |
| chr18 | 690548 | 693908 | A3SS | ENOSF1 | 1 |
| chr18 | 690548 | 693908 | SE | ENOSF1 | 1 |
| chr18 | 691242 | 693908 | SE | ENOSF1 | 1 |
| chr18 | 77798504 | 77804285 | SE | RBFA | 2 |
| chr18 | 9182379 | 9208801 | SE | ANKRD12 | 4 |
| chr19 | 1032695 | 1036065 | A3SS | CNN2 | 2 |
| chr19 | 10909248 | 10912963 | MXE | DNM2 | 2 |
| chr19 | 18668571 | 18672967 | SE | KXD1 | 4 |
| chr19 | 21688405 | 21712586 | SE | ZNF429 | 2 |
| chr19 | 22028308 | 22033739 | MXE | ZNF43 | 1 |
| chr19 | 34706500 | 34710782 | SE | LSM14A | 2 |
| chr19 | 34712643 | 34717312 | MXE | LSM14A | 1 |
| chr19 | 35752827 | 35757435 | SE | LSR | 2 |
| chr19 | 35753595 | 35757261 | MXE | LSR | 2 |
| chr19 | 44015718 | 44030666 | MXE | ETHE1 | 1 |
| chr19 | 44160792 | 44169467 | MXE | PLAUR | 14 |
| chr19 | 45952502 | 45953898 | MXE | ERCC1 | 2 |
| chr19 | 46357648 | 46366452 | SE | SYMPK | 4 |
| chr19 | 49593777 | 49601683 | MXE | SNRNP70 | 2 |
| chr19 | 50410799 | 50432686 | SE | NUP62 | 2 |
| chr19 | 50411361 | 50432692 | SE | NUP62 | 2 |
| chr19 | 50412651 | 50431072 | SE | NUP62 | 2 |
| chr19 | 53119970 | 53122315 | A3SS | ZNF83 | 5 |
| chr19 | 53901195 | 53915260 | SE | ZNF765 | 4 |
| chr19 | 54705354 | 54711512 | SE | RPS9 | 3 |
| chr19 | 54705354 | 54711515 | SE | RPS9 | 3 |
| chr19 | 56884899 | 56890922 | SE | ZNF542 | 1 |
| chr19 | 58331098 | 58370181 | SE | CTD-2583A14.10 | 20 |
| chr19 | 58331173 | 58369945 | SE | CTD-2583A14.10 | 20 |
| chr19 | 8275589 | 8319486 | SE | CERS4 | 12 |
| chr19 | 8275589 | 8319500 | SE | CERS4 | 12 |
| chr2 | 101565828 | 101584890 | SE | NPAS2 | 3 |
| chr2 | 101911643 | 101924590 | MXE | RNF149 | 12 |
| chr2 | 112125063 | 112252628 | SE | MIR4435-1HG | 11 |
| chr2 | 128603839 | 128615731 | SE | POLR2D | 1 |
| chr2 | 15557650 | 15601461 | SE | NBAS | 2 |
| chr2 | 15618349 | 15644337 | SE | NBAS | 1 |
| chr2 | 159954148 | 160005783 | SE | TANC1 | 2 |
| chr2 | 190611385 | 190615382 | A3SS | OSGEPL1 | 2 |
| chr2 | 191874601 | 191878923 | SE | STAT1 | 1 |
| chr2 | 197028151 | 197036056 | MXE | STK17B | 1 |
| chr2 | 201194153 | 201254006 | SE | SPATS2L | 1 |
| chr2 | 201746143 | 201750495 | SE | PPIL3 | 3 |
| chr2 | 202005162 | 202010100 | MXE | CFLAR | 4 |
| chr2 | 203914536 | 203933198 | SE | NBEAL1 | 1 |
| chr2 | 203977737 | 203987066 | SE | NBEAL1 | 1 |
| chr2 | 206927968 | 206950624 | MXE | INO80D | 2 |
| chr2 | 223725651 | 223773754 | SE | ACSL3 | 2 |
| chr2 | 224621705 | 224640726 | SE | AP1S3 | 2 |
| chr2 | 231280656 | 231307814 | SE | SP100 | 2 |
| chr2 | 233562008 | 233599948 | SE | GIGYF2 | 4 |
| chr2 | 236653341 | 236706530 | SE | AGAP1 | 2 |
| chr2 | 238285414 | 238305460 | SE | COL6A3 | 3 |
| chr2 | 238285414 | 238305490 | SE | COL6A3 | 3 |
| chr2 | 238289557 | 238305460 | SE | COL6A3 | 3 |
| chr2 | 238289557 | 238305490 | SE | COL6A3 | 3 |
| chr2 | 238290142 | 238296224 | MXE | COL6A3 | 3 |
| chr2 | 238290142 | 238305369 | MXE | COL6A3 | 3 |
| chr2 | 242207956 | 242254984 | MXE | HDLBP | 5 |
| chr2 | 242343242 | 242347023 | SE | FARP2 | 3 |
| chr2 | 28631733 | 28634745 | A3SS | FOSL2 | 2 |
| chr2 | 32157204 | 32168370 | MXE | MEMO1 | 1 |
| chr2 | 48725631 | 48734524 | SE | PPP1R21 | 1 |
| chr2 | 48725874 | 48734407 | MXE | PPP1R21 | 1 |
| chr2 | 55214626 | 55277680 | SE | RTN4 | 5 |
| chr2 | 55750856 | 55761096 | SE | CCDC104 | 1 |
| chr2 | 61726847 | 61749818 | SE | XPO1 | 25 |
| chr2 | 63206322 | 63217975 | SE | EHBP1 | 1 |
| chr2 | 64199299 | 64211153 | SE | VPS54 | 1 |
| chr2 | 65313988 | 65325200 | SE | RAB1A | 8 |
| chr2 | 70035037 | 70037805 | RI | ANXA4 | 3 |
| chr2 | 73959032 | 73961718 | SE | TPRKB | 2 |
| chr2 | 86684105 | 86691339 | SE | KDM3A | 4 |
| chr2 | 97285095 | 97297219 | SE | KANSL3 | 5 |
| chr20 | 30126162 | 30132749 | A5SS | HM13 | 2 |
| chr20 | 32661368 | 32663845 | SE | RALY | 1 |
| chr20 | 33470596 | 33500990 | SE | ACSS2 | 3 |
| chr20 | 34238247 | 34252822 | SE | RBM12 | 1 |
| chr20 | 34680601 | 34763637 | SE | EPB41L1 | 5 |
| chr20 | 35173261 | 35178228 | SE | MYL9 | 7 |
| chr20 | 3893281 | 3897573 | MXE | PANK2 | 2 |
| chr20 | 40085903 | 40112159 | SE | CHD6 | 1 |
| chr20 | 47790806 | 47804652 | MXE | STAU1 | 1 |
| chr20 | 49518553 | 49547750 | SE | ADNP | 13 |
| chr20 | 50139619 | 50179370 | A3SS | NFATC2 | 3 |
| chr20 | 60699672 | 60705008 | SE | LSM14B | 1 |
| chr21 | 27354656 | 27394358 | SE | APP | 1 |
| chr21 | 27484463 | 27542881 | MXE | APP | 1 |
| chr21 | 35445822 | 35515334 | SE | MRPS6 | 1 |
| chr21 | 36265260 | 36421138 | MXE | RUNX1 | 11 |
| chr22 | 18222114 | 18226779 | A3SS | BID | 1 |
| chr22 | 20891403 | 20918787 | SE | MED15 | 4 |
| chr22 | 20907383 | 20918753 | SE | MED15 | 1 |
| chr22 | 24219228 | 24224833 | SE | SLC2A11 | 1 |
| chr22 | 24627351 | 24641027 | SE | GGT5 | 2 |
| chr22 | 26936754 | 26961334 | SE | TPST2 | 5 |
| chr22 | 26940569 | 26986089 | SE | TPST2 | 5 |
| chr22 | 30367051 | 30374413 | A3SS | MTMR3 | 2 |
| chr22 | 30806584 | 30812392 | SE | RP4-539M6.19 | 2 |
| chr22 | 32017634 | 32044262 | SE | PISD | 1 |
| chr22 | 36334945 | 36424287 | MXE | RBFOX2 | 1 |
| chr22 | 39101727 | 39111967 | SE | GTPBP1 | 1 |
| chr22 | 50810500 | 50832564 | SE | PPP6R2 | 7 |
| chr3 | 122176695 | 122180070 | MXE | KPNA1 | 3 |
| chr3 | 128598446 | 128612499 | SE | ACAD9 | 1 |
| chr3 | 128598462 | 128612499 | SE | ACAD9 | 1 |
| chr3 | 129170756 | 129182469 | SE | IFT122 | 3 |
| chr3 | 141105779 | 141115100 | MXE | ZBTB38 | 1 |
| chr3 | 141697361 | 141724386 | SE | TFDP2 | 1 |
| chr3 | 142280263 | 142284962 | MXE | ATR | 1 |
| chr3 | 146251256 | 146262317 | SE | PLSCR1 | 6 |
| chr3 | 149530494 | 149570383 | SE | RNF13 | 2 |
| chr3 | 150128965 | 150174914 | SE | TSC22D2 | 4 |
| chr3 | 15345806 | 15372010 | MXE | SH3BP5 | 3 |
| chr3 | 155547476 | 155560408 | SE | SLC33A1 | 2 |
| chr3 | 157920860 | 158015864 | SE | RSRC1 | 2 |
| chr3 | 180687945 | 180693192 | SE | FXR1 | 2 |
| chr3 | 183353355 | 183368378 | SE | KLHL24 | 2 |
| chr3 | 183446477 | 183465504 | SE | YEATS2 | 6 |
| chr3 | 187871071 | 188124101 | SE | LPP | 33 |
| chr3 | 195415254 | 195435101 | SE | LINC00969 | 7 |
| chr3 | 196630403 | 196654750 | SE | SENP5 | 5 |
| chr3 | 196630481 | 196654666 | MXE | SENP5 | 5 |
| chr3 | 19992297 | 20017244 | SE | RAB5A | 6 |
| chr3 | 27465527 | 27475595 | SE | SLC4A7 | 1 |
| chr3 | 37292885 | 37315092 | SE | GOLGA4 | 16 |
| chr3 | 37903450 | 38006163 | SE | CTDSPL | 2 |
| chr3 | 38278151 | 38287712 | SE | OXSR1 | 2 |
| chr3 | 47898920 | 47912595 | SE | MAP4 | 1 |
| chr3 | 47918906 | 47956429 | SE | MAP4 | 5 |
| chr3 | 48784011 | 48793877 | SE | PRKAR2A | 3 |
| chr3 | 49413024 | 49449252 | MXE | RHOA | 6 |
| chr3 | 49507748 | 49548252 | SE | DAG1 | 1 |
| chr3 | 50609140 | 50614563 | SE | HEMK1 | 1 |
| chr3 | 56593596 | 56597853 | SE | CCDC66 | 1 |
| chr3 | 56807844 | 56916319 | MXE | ARHGEF3 | 6 |
| chr3 | 57882599 | 57894877 | SE | SLMAP | 4 |
| chr4 | 128905493 | 128930112 | SE | C4orf29 | 1 |
| chr4 | 152147198 | 152246784 | SE | SH3D19 | 4 |
| chr4 | 20706282 | 20715139 | SE | PACRGL | 1 |
| chr4 | 20709425 | 20714454 | SE | PACRGL | 1 |
| chr4 | 20709425 | 20715139 | SE | PACRGL | 1 |
| chr4 | 26322408 | 26388013 | SE | RBPJ | 14 |
| chr4 | 2845671 | 2877837 | SE | ADD1 | 1 |
| chr4 | 41015599 | 41102767 | SE | APBB2 | 1 |
| chr4 | 41015963 | 41102767 | SE | APBB2 | 1 |
| chr4 | 41067592 | 41145159 | SE | APBB2 | 1 |
| chr4 | 41621204 | 41646643 | SE | LIMCH1 | 1 |
| chr4 | 48385801 | 48422141 | MXE | SLAIN2 | 1 |
| chr4 | 48636307 | 48712715 | SE | FRYL | 1 |
| chr4 | 48686689 | 48782265 | SE | FRYL | 4 |
| chr4 | 48894788 | 48906628 | SE | OCIAD2 | 6 |
| chr4 | 55095459 | 55124984 | SE | PDGFRA | 3 |
| chr4 | 56230240 | 56239263 | SE | SRD5A3 | 1 |
| chr4 | 76573822 | 76580430 | SE | G3BP2 | 1 |
| chr4 | 84026048 | 84035868 | SE | PLAC8 | 1 |
| chr4 | 84026173 | 84035818 | MXE | PLAC8 | 1 |
| chr4 | 85853516 | 85887361 | MXE | WDFY3 | 1 |
| chr4 | 89708903 | 89744352 | SE | FAM13A | 1 |
| chr4 | 89708903 | 89744382 | SE | FAM13A | 1 |
| chr5 | 112487119 | 112676215 | MXE | MCC | 1 |
| chr5 | 130517921 | 130541119 | SE | LYRM7 | 4 |
| chr5 | 132240059 | 132267914 | SE | AFF4 | 13 |
| chr5 | 137854380 | 137878625 | A5SS | ETF1 | 6 |
| chr5 | 139851880 | 139862164 | MXE | ANKHD1 | 1 |
| chr5 | 141358395 | 141362944 | MXE | RNF14 | 1 |
| chr5 | 141358395 | 141364367 | MXE | RNF14 | 1 |
| chr5 | 145083466 | 145176094 | SE | PRELID2 | 4 |
| chr5 | 150443173 | 150466719 | SE | TNIP1 | 1 |
| chr5 | 150443173 | 150466727 | SE | TNIP1 | 1 |
| chr5 | 154238098 | 154250264 | SE | CNOT8 | 2 |
| chr5 | 154238105 | 154250382 | SE | CNOT8 | 2 |
| chr5 | 159502888 | 159521072 | SE | PWWP2A | 4 |
| chr5 | 159504850 | 159520187 | SE | PWWP2A | 4 |
| chr5 | 179004075 | 179012866 | SE | RUFY1 | 1 |
| chr5 | 179004137 | 179012796 | MXE | RUFY1 | 1 |
| chr5 | 179125977 | 179132853 | SE | CANX | 1 |
| chr5 | 304291 | 314801 | SE | PDCD6 | 1 |
| chr5 | 304291 | 315084 | SE | PDCD6 | 1 |
| chr5 | 304291 | 315089 | SE | PDCD6 | 1 |
| chr5 | 34182972 | 34218399 | MXE | RP11-1023L17.1 | 2 |
| chr5 | 40775605 | 40777546 | MXE | PRKAA1 | 1 |
| chr5 | 49962771 | 50046022 | SE | PARP8 | 5 |
| chr5 | 65222302 | 65307955 | SE | ERBB2IP | 14 |
| chr5 | 65349233 | 65371058 | SE | ERBB2IP | 3 |
| chr5 | 65349233 | 65372236 | SE | ERBB2IP | 3 |
| chr5 | 66254815 | 66350320 | SE | MAST4 | 1 |
| chr5 | 66259546 | 66350320 | SE | MAST4 | 1 |
| chr5 | 66350320 | 66385989 | MXE | MAST4 | 1 |
| chr5 | 77717784 | 77755067 | MXE | SCAMP1 | 1 |
| chr5 | 82807921 | 82838087 | SE | VCAN | 3 |
| chr5 | 96031611 | 96058342 | MXE | CAST | 2 |
| chr5 | 96058342 | 96063234 | SE | CAST | 2 |
| chr5 | 96058402 | 96062457 | A3SS | CAST | 2 |
| chr5 | 96058402 | 96062497 | MXE | CAST | 2 |
| chr6 | 10530069 | 10621583 | A5SS | GCNT2 | 2 |
| chr6 | 127635960 | 127648282 | SE | ECHDC1 | 3 |
| chr6 | 128718710 | 128841710 | SE | PTPRK | 2 |
| chr6 | 128718710 | 128841744 | SE | PTPRK | 2 |
| chr6 | 131190798 | 131206408 | SE | EPB41L2 | 2 |
| chr6 | 131191266 | 131206235 | MXE | EPB41L2 | 2 |
| chr6 | 150139933 | 150157433 | SE | LRP11 | 3 |
| chr6 | 155054980 | 155095206 | SE | SCAF8 | 20 |
| chr6 | 157431605 | 157470085 | SE | ARID1B | 1 |
| chr6 | 157469923 | 157488319 | SE | ARID1B | 1 |
| chr6 | 167271581 | 167352496 | SE | RP11-514O12.4 | 1 |
| chr6 | 21065278 | 21198335 | SE | CDKAL1 | 3 |
| chr6 | 35447081 | 35452401 | SE | TEAD3 | 1 |
| chr6 | 4117541 | 4126471 | A3SS | ECI2 | 4 |
| chr6 | 86253322 | 86256944 | A5SS | SNX14 | 1 |
| chr6 | 88240501 | 88265223 | SE | RARS2 | 6 |
| chr6 | 88255333 | 88265223 | SE | RARS2 | 4 |
| chr7 | 102727076 | 102739092 | SE | ARMC10 | 1 |
| chr7 | 129479046 | 129519482 | SE | UBE2H | 16 |
| chr7 | 140419126 | 140439746 | SE | BRAF | 1 |
| chr7 | 151329224 | 151372505 | MXE | PRKAG2 | 1 |
| chr7 | 151722882 | 151791607 | SE | GALNT11 | 4 |
| chr7 | 151791274 | 151800259 | SE | GALNT11 | 1 |
| chr7 | 22206687 | 22233362 | SE | RAPGEF5 | 1 |
| chr7 | 27856657 | 27867356 | MXE | TAX1BP1 | 1 |
| chr7 | 30590183 | 30603614 | SE | AC005154.6 | 5 |
| chr7 | 30590251 | 30603614 | SE | AC005154.6 | 5 |
| chr7 | 43927013 | 43946225 | SE | URGCP | 4 |
| chr7 | 44687042 | 44706450 | SE | OGDH | 5 |
| chr7 | 44687133 | 44706334 | MXE | OGDH | 5 |
| chr7 | 44687358 | 44706334 | MXE | OGDH | 5 |
| chr7 | 56101653 | 56119297 | A3SS | PSPH | 4 |
| chr7 | 64126510 | 64139714 | SE | ZNF107 | 4 |
| chr7 | 6431554 | 6439819 | SE | RAC1 | 1 |
| chr7 | 66240213 | 66260579 | SE | RABGEF1 | 1 |
| chr7 | 75659845 | 75676979 | MXE | STYXL1 | 7 |
| chr7 | 75677840 | 75684147 | A5SS | MDH2 | 1 |
| chr7 | 76903106 | 76903798 | MXE | CCDC146 | 1 |
| chr7 | 77026662 | 77045717 | SE | GSAP | 1 |
| chr7 | 8275635 | 8302069 | MXE | ICA1 | 1 |
| chr7 | 90233531 | 90355958 | SE | CDK14 | 6 |
| chr7 | 99158155 | 99169941 | SE | ZNF655 | 5 |
| chr8 | 109215629 | 109240620 | SE | EIF3E | 1 |
| chr8 | 116632139 | 116681228 | SE | TRPS1 | 1 |
| chr8 | 131249167 | 131414216 | SE | ASAP1 | 8 |
| chr8 | 134487961 | 134558169 | SE | ST3GAL1 | 10 |
| chr8 | 141874410 | 141900702 | SE | PTK2 | 2 |
| chr8 | 144669019 | 144679517 | MXE | EEF1D | 2 |
| chr8 | 17513371 | 17542051 | SE | MTUS1 | 4 |
| chr8 | 17542051 | 17570722 | MXE | MTUS1 | 3 |
| chr8 | 22224817 | 22262490 | SE | SLC39A14 | 6 |
| chr8 | 30361802 | 30405000 | SE | RBPMS | 9 |
| chr8 | 38315052 | 38325498 | MXE | FGFR1 | 1 |
| chr8 | 38585703 | 38677149 | SE | TACC1 | 28 |
| chr8 | 38585703 | 38682886 | SE | TACC1 | 28 |
| chr8 | 38586104 | 38645261 | SE | TACC1 | 26 |
| chr8 | 38586104 | 38646337 | SE | TACC1 | 26 |
| chr8 | 38599868 | 38646337 | SE | TACC1 | 22 |
| chr8 | 38614798 | 38677149 | SE | TACC1 | 16 |
| chr8 | 38758861 | 38775588 | SE | PLEKHA2 | 7 |
| chr8 | 38913095 | 38934930 | SE | ADAM9 | 1 |
| chr8 | 42935584 | 42938260 | MXE | FNTA | 1 |
| chr8 | 48626050 | 48647936 | SE | SPIDR | 1 |
| chr8 | 54975887 | 55013499 | SE | LYPLA1 | 4 |
| chr8 | 71082436 | 71126310 | SE | NCOA2 | 4 |
| chr8 | 74742615 | 74791110 | SE | UBE2W | 9 |
| chr8 | 80831174 | 80942524 | SE | MRPS28 | 1 |
| chr8 | 99607557 | 99718862 | SE | STK3 | 7 |
| chr9 | 100872170 | 100881418 | SE | TRIM14 | 8 |
| chr9 | 115030328 | 115060196 | SE | PTBP3 | 2 |
| chr9 | 115446205 | 115456513 | SE | INIP | 4 |
| chr9 | 124030337 | 124064445 | SE | GSN | 4 |
| chr9 | 124043747 | 124064427 | SE | GSN | 4 |
| chr9 | 124043747 | 124064445 | SE | GSN | 4 |
| chr9 | 124043749 | 124064427 | SE | GSN | 4 |
| chr9 | 125613359 | 125616365 | SE | RC3H2 | 4 |
| chr9 | 128420078 | 128469249 | MXE | MAPKAP1 | 1 |
| chr9 | 128510460 | 128692124 | SE | PBX3 | 15 |
| chr9 | 131223346 | 131231461 | MXE | ODF2 | 1 |
| chr9 | 131700223 | 131702647 | A5SS | PHYHD1 | 2 |
| chr9 | 132371162 | 132396500 | SE | NTMT1 | 3 |
| chr9 | 134477447 | 134497225 | SE | RAPGEF1 | 2 |
| chr9 | 140513443 | 140611502 | SE | EHMT1 | 6 |
| chr9 | 33116111 | 33120416 | MXE | B4GALT1 | 2 |
| chr9 | 4490443 | 4561541 | SE | SLC1A1 | 1 |
| chr9 | 6814745 | 6849506 | MXE | KDM4C | 2 |
| chr9 | 72914961 | 72929752 | SE | SMC5 | 1 |
| chr9 | 84235355 | 84267203 | SE | TLE1 | 2 |
| chr9 | 99086372 | 99122503 | SE | SLC35D2 | 3 |
| chrX | 100291962 | 100296360 | SE | TRMT2B | 1 |
| chrX | 102930423 | 102940188 | SE | MORF4L2 | 1 |
| chrX | 102930427 | 102940188 | SE | MORF4L2 | 1 |
| chrX | 102930432 | 102941149 | SE | MORF4L2 | 1 |
| chrX | 102931753 | 102940188 | SE | MORF4L2 | 1 |
| chrX | 106312539 | 106358684 | SE | RBM41 | 2 |
| chrX | 129208555 | 129244691 | SE | ELF4 | 1 |
| chrX | 134429693 | 134478012 | A5SS | ZNF75D | 1 |
| chrX | 149984551 | 149996778 | MXE | CD99L2 | 2 |
| chrX | 19947902 | 19955650 | SE | CXorf23 | 2 |
| chrX | 3735568 | 3746900 | SE | RP11-706O15.1 | 3 |
| chrX | 40498260 | 40506393 | A3SS | CXorf38 | 2 |
| chrX | 57407344 | 57473472 | SE | FAAH2 | 1 |
| chrX | 73214674 | 73224186 | SE | JPX | 3 |
| chrX | 76954061 | 77041702 | SE | ATRX | 6 |
| chrX | 77086301 | 77111087 | SE | MAGT1 | 1 |

**Table S4 The charactericstic of DREs under positive selection among immune-related genes**

| group | chromosome | position | gene | RNA editing |  |  | RNA expression |  |  |
| --- | --- | --- | --- | --- | --- | --- | --- | --- | --- |
|  |  |  |  | *F_ST_* | mean_tumor_editing_level | mean_normal_editing_level | log2FoldChange | pvalue | padj |
| Normal-specific Cohort | chr1 | 9765521 | PIK3CD | 0.344086022 | 0 | 0.512 | 0.243320905 | 0.222958579 | 0.431863191 |
|  | chr1 | 41187732 | NFYC | 0.261261261 | 0 | 0.414285714 | 0.142387468 | 0.008299458 | 0.046016972 |
|  | chr1 | 41189826 | NFYC | 0.288936627 | 0 | 0.448333333 | 0.142387468 | 0.008299458 | 0.046016972 |
|  | chr1 | 65493009 | JAK1 | 0.320132013 | 0 | 0.485 | 0.180228458 | 0.017231422 | 0.077243281 |
|  | chr1 | 65509302 | JAK1 | 0.335113485 | 0 | 0.502 | 0.180228458 | 0.017231422 | 0.077243281 |
|  | chr1 | 156136834 | SEMA4A | 0.25984252 | 0 | 0.4125 | 0.721625872 | 0.0000122 | 0.000417378 |
|  | chr1 | 217205050 | ESRRG | 0.350048216 | 0 | 0.518571429 | 0.641336987 | 0.08115343 | 0.22493269 |
|  | chr10 | 33569801 | NRP1 | 0.27388535 | 0 | 0.43 | 0.206796779 | 0.062073457 | 0.187581715 |
|  | chr11 | 75002193 | ARRB1 | 0.388888889 | 0 | 0.56 | 0.367325243 | 0.000102782 | 0.002033407 |
|  | chr11 | 103969348 | PDGFD | 0.629327902 | 0 | 0.7725 | 0.501771431 | 0.013269817 | 0.064280312 |
|  | chr12 | 1837829 | ADIPOR2 | 0.255230126 | 0 | 0.406666667 | 0.079668058 | 0.45101202 | 0.658431718 |
|  | chr12 | 64868813 | TBK1 | 0.267427123 | 0 | 0.422 | -0.287541772 | 0.001849165 | 0.015685926 |
|  | chr14 | 81546408 | TSHR | 0.467889908 | 0 | 0.6375 | 0.825325563 | 0.0000431 | 0.001085539 |
|  | chr15 | 61147989 | RORA | 0.377726751 | 0 | 0.548333333 | 0.128934909 | 0.353663345 | 0.572529979 |
|  | chr15 | 61204191 | RORA | 0.57480315 | 0 | 0.73 | 0.128934909 | 0.353663345 | 0.572529979 |
|  | chr15 | 61204759 | RORA | 0.257861635 | 0 | 0.41 | 0.128934909 | 0.353663345 | 0.572529979 |
|  | chr15 | 76240779 | NRG4 | 0.26984127 | 0 | 0.425 | 0.698727808 | 0.024867822 | 0.099886719 |
|  | chr15 | 76240874 | NRG4 | 0.369863014 | 0 | 0.54 | 0.698727808 | 0.024867822 | 0.099886719 |
|  | chr16 | 27430416 | IL21R | 0.282051282 | 0 | 0.44 | -0.137397174 | 0.589480831 | 0.763960417 |
|  | chr18 | 316979 | COLEC12 | 0.6563147 | 0 | 0.7925 | 0.964902871 | 0.000139918 | 0.002531836 |
|  | chr19 | 6812611 | VAV1 | 0.300813008 | 0 | 0.4625 | 0.047921859 | 0.801044338 | 0.900764865 |
|  | chr19 | 7253346 | INSR | 0.320132013 | 0 | 0.485 | 0.480426796 | 0.023282064 | 0.09540999 |
|  | chr19 | 38868793 | PSMD8 | 0.315789474 | 0 | 0.48 | -0.362546602 | 0.000229376 | 0.003556472 |
|  | chr19 | 50019894 | FCGRT | 0.257861635 | 0 | 0.41 | 0.785251627 | 4.24E-08 | 0.00000574 |
|  | chr2 | 203316691 | BMPR2 | 0.344537815 | 0 | 0.5125 | -0.310654756 | 0.00696361 | 0.04067495 |
|  | chr22 | 22155484 | MAPK1 | 0.589825119 | 0 | 0.742 | 0.006436153 | 0.887854527 | 0.945094478 |
|  | chr22 | 46605755 | PPARA | 0.317957166 | 0 | 0.4825 | 0.243895877 | 0.191960733 | 0.39391195 |
|  | chr3 | 12679195 | RAF1 | 0.367521368 | 0 | 0.5375 | 0.129212981 | 0.002857093 | 0.021616675 |
|  | chr4 | 110931660 | EGF | 0.386481802 | 0 | 0.5575 | 1.712876364 | 0.000432803 | 0.005544402 |
|  | chr4 | 110931773 | EGF | 0.39275766 | 0 | 0.564 | 1.712876364 | 0.000432803 | 0.005544402 |
|  | chr5 | 96242638 | ERAP2 | 0.367521368 | 0 | 0.5375 | -0.45884838 | 0.059216882 | 0.181438069 |
|  | chr5 | 142724386 | NR3C1 | 0.314060447 | 0 | 0.478 | 0.102532915 | 0.264891599 | 0.481222823 |
|  | chr5 | 179128490 | CANX | 0.371742112 | 0 | 0.542 | -0.24402816 | 0.008166357 | 0.045511948 |
|  | chr6 | 33275281 | TAPBP | 0.25984252 | 0 | 0.4125 | -0.251269384 | 0.168834361 | 0.36247844 |
|  | chr7 | 140488527 | BRAF | 0.360544218 | 0 | 0.53 | 0.10508962 | 0.148916705 | 0.33419699 |
|  | chr7 | 140588486 | BRAF | 0.25 | 0 | 0.4 | 0.10508962 | 0.148916705 | 0.33419699 |
|  | chr8 | 38305858 | FGFR1 | 0.287948482 | 0 | 0.447142857 | 1.048568741 | 0.00000268 | 0.000136531 |
| Tumor-specific Cohort | chr1 | 151232087 | PSMD4 | 0.267427123 | 0.422 | 0 | -0.066688896 | 0.4069049 | 0.620881851 |
|  | chr10 | 83760995 | NRG3 | 0.584786054 | 0.738 | 0 | -1.032597085 | 0.002180902 | 0.017765668 |
|  | chr10 | 83832714 | NRG3 | 0.253918495 | 0.405 | 0 | -1.032597085 | 0.002180902 | 0.017765668 |
|  | chr11 | 77059981 | PAK1 | 0.337792642 | 0.505 | 0 | -0.510387672 | 0.000000224 | 0.0000211 |
|  | chr11 | 89157119 | NOX4 | 0.47601476 | 0.645 | 0 | -0.728489954 | 0.000814495 | 0.008736486 |
|  | chr11 | 89157140 | NOX4 | 0.32231405 | 0.4875 | 0 | -0.728489954 | 0.000814495 | 0.008736486 |
|  | chr12 | 25382928 | KRAS | 0.388888889 | 0.56 | 0 | -0.492995854 | 0.0000212 | 0.000631801 |
|  | chr12 | 95451162 | NR2C1 | 0.503759398 | 0.67 | 0 | -0.128185667 | 0.147251029 | 0.331795554 |
|  | chr15 | 78760655 | IREB2 | 0.290322581 | 0.45 | 0 | -0.110349712 | 0.095175206 | 0.250302834 |
|  | chr15 | 99472025 | IGF1R | 0.420454545 | 0.592 | 0 | -0.014300314 | 0.912802303 | 0.957800119 |
|  | chr2 | 208458068 | CREB1 | 0.257861635 | 0.41 | 0 | -0.133154902 | 0.078672931 | 0.220123854 |
|  | chr20 | 35998768 | SRC | 0.309328969 | 0.4725 | 0 | -0.460836817 | 0.000576555 | 0.006841982 |
|  | chr22 | 22155785 | MAPK1 | 0.535508637 | 0.6975 | 0 | 0.006436153 | 0.887854527 | 0.945094478 |
|  | chr3 | 12404457 | PPARG | 0.262626263 | 0.416 | 0 | 0.085705294 | 0.716727004 | 0.849197406 |
|  | chr3 | 12432364 | PPARG | 0.407035176 | 0.578571429 | 0 | 0.085705294 | 0.716727004 | 0.849197406 |
|  | chr3 | 119714098 | GSK3B | 0.251956182 | 0.4025 | 0 | -0.112195341 | 0.129092471 | 0.304428575 |
|  | chr3 | 138530847 | PIK3CB | 0.25588697 | 0.4075 | 0 | -0.13703593 | 0.288907643 | 0.507726961 |
|  | chr3 | 195535224 | MUC4 | 0.272534464 | 0.428333333 | 0 | -1.259607041 | 0.001468098 | 0.013350855 |
|  | chr4 | 148421096 | EDNRA | 0.393728223 | 0.565 | 0 | -0.372385178 | 0.051309555 | 0.165384039 |
|  | chr5 | 142692517 | NR3C1 | 0.351351351 | 0.52 | 0 | 0.102532915 | 0.264891599 | 0.481222823 |
|  | chr8 | 56834901 | LYN | 0.360544218 | 0.53 | 0 | -0.106486049 | 0.413915196 | 0.626966403 |
|  | chr8 | 76349096 | HNF4G | 0.337792642 | 0.505 | 0 | -0.107468664 | 0.748209202 | 0.869406651 |
|  | chr9 | 130238690 | LRSAM1 | 0.255230126 | 0.406666667 | 0 | -0.020465209 | 0.77752232 | 0.887641491 |

**Table S5 The list of different expression genes betweeen PDAC and normal tissues.**

| GENE | baseMean | log2FoldChange | lfcSE | stat | pvalue | padj |
| --- | --- | --- | --- | --- | --- | --- |
| DES | 1886.475239 | 1.686591207 | 0.388619198 | 4.339958537 | 1.43E-05 | 0.000468563 |
| DNAJB3 | 42.07833709 | -2.506805855 | 0.525713274 | -4.768389881 | 1.86E-06 | 0.000103156 |
| IL13 | 9.839762876 | 1.408643735 | 0.532880473 | 2.643451593 | 0.00820655 | 0.045677193 |
| CYP24A1 | 218.4441759 | -2.534913869 | 0.527168013 | -4.808550227 | 1.52E-06 | 8.98E-05 |
| CILP | 6572.28967 | 1.093226508 | 0.311472926 | 3.509860462 | 0.000448342 | 0.00568371 |
| S100A11 | 12986.03079 | -1.021730628 | 0.186197593 | -5.487346071 | 4.08E-08 | 5.58E-06 |
| AC015977.6 | 15.02462034 | 2.091185031 | 0.583775514 | 3.582173254 | 0.000340748 | 0.004698535 |
| TUSC5 | 1057.869403 | 3.961529404 | 0.621641665 | 6.372689653 | 1.86E-10 | 7.71E-08 |
| LINC00341 | 885.6754081 | 1.340025487 | 0.235227609 | 5.696718576 | 1.22E-08 | 2.17E-06 |
| RP11-547D24.1 | 14.46205388 | -1.150544404 | 0.425440337 | -2.704361348 | 0.006843582 | 0.040228946 |
| DCT | 75.15272946 | 1.278332021 | 0.231428321 | 5.523662854 | 3.32E-08 | 4.85E-06 |
| KCNA4 | 38.74479391 | 2.204098519 | 0.562432983 | 3.918864267 | 8.90E-05 | 0.001821142 |
| KRT19P4 | 19.68546191 | 1.208906645 | 0.375463849 | 3.219768426 | 0.001282942 | 0.012136904 |
| SMIM9 | 4.487449597 | 1.885147482 | 0.588642882 | 3.202531687 | 0.001362253 | 0.012641852 |
| SERPINB7 | 184.4606574 | -1.649366338 | 0.505006151 | -3.266032176 | 0.001090659 | 0.010838489 |
| AC020907.2 | 54.81641386 | 1.097760322 | 0.275740887 | 3.981130015 | 6.86E-05 | 0.001495864 |
| TMEM88 | 224.6780716 | 1.599519447 | 0.218403452 | 7.32369123 | 2.41E-13 | 3.24E-10 |
| AC011294.3 | 12.68323354 | -1.980724108 | 0.644804072 | -3.071823199 | 0.002127557 | 0.017425769 |
| WFDC13 | 48.10915581 | -1.731149762 | 0.369688129 | -4.682730186 | 2.83E-06 | 0.000142253 |
| EGR3 | 5383.060345 | 1.702374449 | 0.323908725 | 5.255722738 | 1.47E-07 | 1.51E-05 |
| CRYAB | 1398.738829 | 1.315789365 | 0.237252338 | 5.545948992 | 2.92E-08 | 4.44E-06 |
| ACADL | 1280.666211 | 1.035887282 | 0.329668576 | 3.142208138 | 0.001676788 | 0.014670483 |
| SNORD3B-2 | 357.0607921 | 1.133254977 | 0.220114703 | 5.148474681 | 2.63E-07 | 2.36E-05 |
| SLC2A1 | 9010.575881 | -1.720405909 | 0.309929145 | -5.55096523 | 2.84E-08 | 4.36E-06 |
| RN7SKP154 | 7.25513108 | 1.026044605 | 0.307845868 | 3.332981573 | 0.000859206 | 0.009114459 |
| RP11-169D4.1 | 47.2887909 | 1.399305371 | 0.378502858 | 3.696947968 | 0.000218207 | 0.003433811 |
| DRC1 | 39.93116962 | -1.569791343 | 0.457605971 | -3.430443311 | 0.000602596 | 0.007077779 |
| MT1G | 4877.732385 | 1.909289442 | 0.464081706 | 4.114123477 | 3.89E-05 | 0.001001688 |
| HAPLN2 | 12.20763588 | 1.595300605 | 0.554669438 | 2.876128548 | 0.004025857 | 0.027551475 |
| BIRC7 | 97.58779183 | -1.113559017 | 0.333291798 | -3.341093377 | 0.000834492 | 0.00890593 |
| MIR4740 | 4.518614824 | 1.044429262 | 0.323944757 | 3.224096828 | 0.001263707 | 0.012013257 |
| RP11-735G4.1 | 6.239121325 | 1.802832507 | 0.617531889 | 2.919416047 | 0.003506878 | 0.02504592 |
| RP11-574O7.1 | 10.51924542 | 1.270109615 | 0.441832641 | 2.874639622 | 0.004044889 | 0.027642909 |
| SIM2 | 556.8729906 | -1.32028471 | 0.292556264 | -4.512925796 | 6.39E-06 | 0.000262731 |
| RPS20P22 | 479.7548338 | 1.533477192 | 0.25979557 | 5.902630252 | 3.58E-09 | 8.40E-07 |
| SPTA1 | 106.8045898 | 1.186733053 | 0.349712854 | 3.393449906 | 0.000690182 | 0.007776895 |
| GLS2 | 1110.644529 | 1.163159396 | 0.273125174 | 4.258704454 | 2.06E-05 | 0.000618321 |
| TRBV6-4 | 37.70132143 | 1.791473804 | 0.501059306 | 3.575372784 | 0.000349729 | 0.004788515 |
| ALDH1L1-AS2 | 54.79004573 | 2.121996953 | 0.412964706 | 5.138446271 | 2.77E-07 | 2.45E-05 |
| CTD-3222D19.2 | 9.904818521 | 1.269085003 | 0.368525067 | 3.443687059 | 0.000573839 | 0.006815983 |
| RP11-217B1.2 | 29.40248944 | 1.350745346 | 0.396587287 | 3.405921953 | 0.00065941 | 0.007536255 |
| CNTNAP4 | 38.30115252 | 1.069065421 | 0.388919502 | 2.748808984 | 0.005981223 | 0.036477603 |
| TNNT3 | 140.5764212 | 1.971799388 | 0.335673822 | 5.874152992 | 4.25E-09 | 9.52E-07 |
| NPSR1-AS1 | 123.9004875 | -1.615067963 | 0.480090743 | -3.36408895 | 0.000767968 | 0.008400594 |
| ADHFE1 | 675.0675716 | 1.104701808 | 0.187691504 | 5.885731545 | 3.96E-09 | 9.04E-07 |
| DEFB132 | 10.382787 | 4.591162356 | 0.999279292 | 4.594473628 | 4.34E-06 | 0.000197637 |
| RNU6-401P | 2.788399592 | 1.118451242 | 0.427223463 | 2.617953691 | 0.008845881 | 0.048122925 |
| RP11-336A10.5 | 11.39237254 | -1.50583631 | 0.493970095 | -3.048436181 | 0.002300358 | 0.018457294 |
| AL590822.1 | 97.46440624 | 1.201985981 | 0.246491826 | 4.876372586 | 1.08E-06 | 6.87E-05 |
| CDC20B | 30.44393962 | -1.292931772 | 0.432744641 | -2.987747621 | 0.002810415 | 0.021376889 |
| TNMD | 86.31408362 | 2.473099379 | 0.425443427 | 5.812992327 | 6.14E-09 | 1.26E-06 |
| RP11-678G14.4 | 50.96683814 | 1.173149775 | 0.389697755 | 3.010409378 | 0.002608958 | 0.020197799 |
| FAM180B | 49.00006962 | 1.375536386 | 0.428669911 | 3.208847533 | 0.001332681 | 0.012442456 |
| CEACAMP2 | 2.990472059 | 1.253856846 | 0.408800294 | 3.06716229 | 0.002161015 | 0.017638172 |
| OR52N3P | 79.37045611 | 2.298889172 | 0.403521998 | 5.697060339 | 1.22E-08 | 2.17E-06 |
| FAM47E | 260.873272 | 1.505189271 | 0.470022314 | 3.202378325 | 0.001362979 | 0.012645575 |
| ZNF355P | 24.72607489 | -1.630498295 | 0.611371366 | -2.666952341 | 0.007654253 | 0.043528887 |
| RP11-96H17.1 | 97.4666168 | -1.471493251 | 0.399562306 | -3.682762938 | 0.00023072 | 0.003571621 |
| RP11-556E13.1 | 11.51353406 | -1.077390768 | 0.353883821 | -3.04447591 | 0.002330861 | 0.01861014 |
| CEACAM16 | 49.1584532 | 1.050598933 | 0.287209267 | 3.657956241 | 0.000254234 | 0.003821756 |
| AC005009.2 | 9.682224007 | 1.37195239 | 0.453503343 | 3.025231039 | 0.002484433 | 0.019489127 |
| A2ML1 | 390.4731491 | -1.980956218 | 0.424420493 | -4.667437717 | 3.05E-06 | 0.000149912 |
| RP11-9M16.3 | 3.852363807 | 1.276626478 | 0.447899187 | 2.850254064 | 0.004368432 | 0.02929945 |
| GULOP | 25.69865332 | 1.693440435 | 0.372612015 | 4.544782156 | 5.50E-06 | 0.00023643 |
| SAA1 | 5571.587882 | 2.145448464 | 0.46693737 | 4.594724262 | 4.33E-06 | 0.000197637 |
| CTSG | 257.6434975 | 1.751108479 | 0.330821311 | 5.293215461 | 1.20E-07 | 1.28E-05 |
| CTC-786C10.1 | 147.0799684 | 1.193927685 | 0.209929143 | 5.687288909 | 1.29E-08 | 2.28E-06 |
| IGHV4-80 | 8.668193375 | -1.016342875 | 0.383646689 | -2.649163681 | 0.008069124 | 0.045133546 |
| PCDH19 | 397.6170983 | 1.073978064 | 0.276200435 | 3.888401065 | 0.000100907 | 0.002007582 |
| ALAS2 | 39.01497658 | 1.398569588 | 0.29410609 | 4.755323451 | 1.98E-06 | 0.000108208 |
| RP11-99J16__A.2 | 59.53933235 | -1.333572911 | 0.465088591 | -2.86735245 | 0.004139218 | 0.028144515 |
| RP11-10A14.5 | 24.35081225 | -1.650970759 | 0.466444801 | -3.539477245 | 0.00040092 | 0.005260009 |
| IRX6 | 52.95816284 | 2.424032536 | 0.536256327 | 4.520287057 | 6.18E-06 | 0.000255728 |
| SNRPEP10 | 35.87367366 | -1.653527816 | 0.492169289 | -3.359672888 | 0.000780348 | 0.008492792 |
| DUXAP8 | 99.15055405 | -1.081105238 | 0.332085332 | -3.25550434 | 0.001131912 | 0.01113213 |
| RAMP3 | 922.1010218 | 1.014505017 | 0.207936724 | 4.878912199 | 1.07E-06 | 6.82E-05 |
| CSF2 | 29.13006275 | -1.165543296 | 0.407035153 | -2.863495421 | 0.004189949 | 0.028385519 |
| RP11-673E11.2 | 2.297553825 | 3.826112116 | 1.149330454 | 3.328992199 | 0.000871608 | 0.009215936 |
| RN7SL608P | 49.23156254 | 1.28961196 | 0.449662234 | 2.867957016 | 0.004131317 | 0.028120211 |
| TRBV5-5 | 48.47485624 | 1.471632988 | 0.422720947 | 3.481334431 | 0.000498922 | 0.006147067 |
| HRC | 121.1096541 | 1.622364417 | 0.248750303 | 6.522060069 | 6.93E-11 | 3.61E-08 |
| RP11-1H8.3 | 33.98493746 | 1.043913258 | 0.288047093 | 3.624106206 | 0.000289962 | 0.004194023 |
| CDY9P | 9.848316883 | -3.761750111 | 1.24723112 | -3.016081023 | 0.002560648 | 0.019918707 |
| AC016995.3 | 30.98498774 | 1.06378806 | 0.32245007 | 3.299078402 | 0.000970028 | 0.009951657 |
| TRBV10-2 | 50.58960783 | 1.720767828 | 0.487306778 | 3.531179753 | 0.00041371 | 0.005377547 |
| CYP4F3 | 601.3620638 | -1.010916589 | 0.331391086 | -3.050524388 | 0.002284421 | 0.018378586 |
| UGT1A7 | 139.6854915 | -1.995081076 | 0.503964268 | -3.958774867 | 7.53E-05 | 0.001606176 |
| PROX1-AS1 | 242.2090275 | 1.073572546 | 0.373013056 | 2.878109838 | 0.004000658 | 0.027460407 |
| SYN2 | 108.2464336 | 1.199865673 | 0.288462732 | 4.159517126 | 3.19E-05 | 0.000864657 |
| MIR5683 | 6.526838057 | 1.138666646 | 0.300699597 | 3.786724884 | 0.000152646 | 0.002677656 |
| RP1-251M9.3 | 5.471955728 | 1.987315254 | 0.474843082 | 4.185204183 | 2.85E-05 | 0.000788507 |
| CTB-167B5.2 | 710.993889 | 1.055845233 | 0.223276444 | 4.728869796 | 2.26E-06 | 0.00012044 |
| CCNB1IP1P3 | 6.347300262 | 2.106541024 | 0.677615311 | 3.108756531 | 0.001878765 | 0.015840525 |
| RP4-553F4.6 | 7.028869683 | -1.035916399 | 0.397874652 | -2.603625019 | 0.009224362 | 0.049628034 |
| LRRC15 | 1711.811045 | -1.087167395 | 0.350596874 | -3.100904418 | 0.001929306 | 0.016172242 |
| MIR3617 | 4.01490025 | -1.769050003 | 0.496147303 | -3.56557416 | 0.00036306 | 0.004914107 |
| MRPL48P1 | 2.530604606 | -2.148656849 | 0.76966054 | -2.791694179 | 0.005243289 | 0.033278327 |
| RP11-165P7.1 | 43.42434624 | -1.210401204 | 0.265278846 | -4.56275056 | 5.05E-06 | 0.000222712 |
| PARD6A | 319.3199672 | 1.02102056 | 0.263633879 | 3.872873105 | 0.00010756 | 0.002106628 |
| LRP3 | 1385.501862 | 1.172393086 | 0.207918527 | 5.638713888 | 1.71E-08 | 2.92E-06 |
| RPS3AP53 | 3.827046404 | -2.075580084 | 0.713225573 | -2.91013133 | 0.003612769 | 0.025595918 |
| WNT7A | 364.2578578 | -1.225016832 | 0.381309193 | -3.212660106 | 0.001315118 | 0.012342028 |
| GPR35 | 1069.49833 | -1.016346598 | 0.281146211 | -3.615010825 | 0.000300335 | 0.004304233 |
| LMOD2 | 15.67991203 | 1.057700535 | 0.315955828 | 3.34762154 | 0.000815082 | 0.00873651 |
| LGALS7 | 21.88142106 | -1.914172391 | 0.735101151 | -2.603957821 | 0.00921541 | 0.049593555 |
| CPN2 | 10.6645377 | 1.764522599 | 0.644995845 | 2.735711576 | 0.006224556 | 0.037585406 |
| AC026703.1 | 1386.631335 | -1.260121111 | 0.314223236 | -4.010273484 | 6.06E-05 | 0.001379001 |
| AMIGO2 | 2840.112408 | -1.269320811 | 0.210021883 | -6.043755032 | 1.51E-09 | 4.19E-07 |
| KRT6A | 894.5176211 | -2.239136009 | 0.498802148 | -4.489026394 | 7.15E-06 | 0.000282396 |
| RP11-286H14.4 | 10.18906481 | 1.777798773 | 0.476063077 | 3.734376516 | 0.000188181 | 0.003090746 |
| IGFBP1 | 194.8485242 | -1.33773223 | 0.468391017 | -2.85601598 | 0.004289935 | 0.028892233 |
| RNU6-509P | 19.68008862 | 1.4433732 | 0.360126438 | 4.007962335 | 6.12E-05 | 0.001386901 |
| CTD-2297D10.2 | 17.06709768 | 1.732422228 | 0.442656075 | 3.913698072 | 9.09E-05 | 0.001848922 |
| LINC00844 | 9.559250269 | 2.272376209 | 0.773254158 | 2.938718385 | 0.003295724 | 0.023993 |
| CDO1 | 1087.475167 | 1.875494916 | 0.294020307 | 6.378793805 | 1.78E-10 | 7.48E-08 |
| RP11-64C12.7 | 26.38900859 | 1.626925847 | 0.618688883 | 2.629634849 | 0.008547662 | 0.046985635 |
| SIGLEC11 | 259.7678532 | 1.390050297 | 0.262631498 | 5.292778321 | 1.20E-07 | 1.28E-05 |
| GFRA1 | 2279.403364 | 1.311101819 | 0.250248356 | 5.239202529 | 1.61E-07 | 1.63E-05 |
| CTC-338M12.1 | 9.899583518 | -1.227474495 | 0.394747152 | -3.109520837 | 0.001873911 | 0.015822798 |
| RP11-496B10.3 | 3.946560611 | 1.337088802 | 0.474966622 | 2.815121612 | 0.004875878 | 0.031699709 |
| AC007881.4 | 5.488630932 | 1.164922684 | 0.335115157 | 3.4761862 | 0.000508599 | 0.006225661 |
| RP11-89K21.1 | 60.57251834 | -2.338235907 | 0.528642437 | -4.423095351 | 9.73E-06 | 0.000355252 |
| TMPRSS11F | 5.523362792 | -1.395159735 | 0.481173282 | -2.899495434 | 0.003737638 | 0.026186888 |
| TM4SF20 | 170.2490409 | -1.154875114 | 0.411310192 | -2.807796006 | 0.004988181 | 0.032145783 |
| RP11-328D5.1 | 9.335774441 | 1.211994178 | 0.424612498 | 2.854353519 | 0.004312451 | 0.029008805 |
| SDR16C5 | 1016.599497 | -1.355080033 | 0.406966257 | -3.329711028 | 0.000869362 | 0.009202159 |
| MIR140 | 3.786998259 | 1.158140602 | 0.314064933 | 3.687583301 | 0.000226394 | 0.003518626 |
| RP11-624L4.2 | 24.20423613 | 1.63288009 | 0.334617306 | 4.879843518 | 1.06E-06 | 6.80E-05 |
| PGM5 | 1604.386617 | 1.174413022 | 0.255240769 | 4.601196849 | 4.20E-06 | 0.000193254 |
| SPINK13 | 33.92378651 | -1.125663697 | 0.382600056 | -2.942142011 | 0.003259504 | 0.023806773 |
| S100A5 | 18.28632485 | -1.381331746 | 0.318580017 | -4.335902048 | 1.45E-05 | 0.000474092 |
| CEACAM6 | 31557.90771 | -1.586047978 | 0.446710482 | -3.550505402 | 0.000384492 | 0.005120654 |
| IGHV3-23 | 3966.848902 | -1.069395245 | 0.359035888 | -2.978519087 | 0.00289645 | 0.021846627 |
| SNORD113 | 5.257517559 | 1.246112949 | 0.317686001 | 3.922467291 | 8.76E-05 | 0.001799782 |
| CELA2B | 45920.05062 | 1.653336688 | 0.619381544 | 2.669334764 | 0.007600166 | 0.0433034 |
| TRBV5-7 | 65.61898048 | 1.986968782 | 0.44851388 | 4.430116593 | 9.42E-06 | 0.000345507 |
| RGN | 1561.877218 | 1.418644512 | 0.360877753 | 3.931094394 | 8.46E-05 | 0.001754659 |
| AQP6 | 61.78373547 | -1.645654093 | 0.376201481 | -4.374395572 | 1.22E-05 | 0.000417625 |
| RP11-336K24.5 | 11.05298512 | -1.431462877 | 0.428939461 | -3.337214242 | 0.000846227 | 0.009003716 |
| KIAA1239 | 206.0736423 | 1.482697301 | 0.337942259 | 4.38742791 | 1.15E-05 | 0.000399099 |
| AC006262.4 | 25.31117974 | -1.129858343 | 0.390823406 | -2.89096898 | 0.00384056 | 0.026710332 |
| RPS12P2 | 4.734229956 | -1.37693991 | 0.420982936 | -3.270773687 | 0.001072537 | 0.010686758 |
| CELF2-AS2 | 38.41900639 | 1.545676909 | 0.291957376 | 5.294186874 | 1.20E-07 | 1.28E-05 |
| PNLIPRP2 | 97704.96678 | 2.005362361 | 0.627735695 | 3.194596672 | 0.001400264 | 0.012881172 |
| SEPP1 | 24981.46485 | 1.272372926 | 0.252134336 | 5.046408774 | 4.50E-07 | 3.56E-05 |
| IGFL2 | 282.8510761 | -1.286908563 | 0.393077076 | -3.273934409 | 0.001060612 | 0.010615651 |
| IGFBP3 | 20973.44007 | -1.064365459 | 0.223398917 | -4.764416382 | 1.89E-06 | 0.000104613 |
| GCAT | 1001.470408 | 1.292268086 | 0.318279641 | 4.060165712 | 4.90E-05 | 0.001192909 |
| CTC-457E21.1 | 178.2946781 | 1.362748224 | 0.403167846 | 3.380101457 | 0.000724591 | 0.008038525 |
| RP11-342F17.1 | 64.42101591 | 1.972980467 | 0.336266671 | 5.867309012 | 4.43E-09 | 9.78E-07 |
| RP11-116D17.3 | 10.98568995 | 1.392471638 | 0.399444178 | 3.486023121 | 0.000490259 | 0.006067164 |
| RPL37P1 | 9.755645518 | -1.29850026 | 0.422329031 | -3.074617572 | 0.002107726 | 0.017303323 |
| ACOX2 | 666.4688901 | 1.215182208 | 0.217716972 | 5.581476706 | 2.38E-08 | 3.78E-06 |
| FOXL1 | 1062.042958 | -1.626998157 | 0.331558347 | -4.907124714 | 9.24E-07 | 6.09E-05 |
| SLC22A6 | 8.351101004 | 2.948208118 | 0.808520106 | 3.646425235 | 0.000265914 | 0.003935221 |
| RN7SL502P | 18.75148502 | 1.017009469 | 0.282387825 | 3.601463587 | 0.000316431 | 0.004452983 |
| EBF1 | 3230.142336 | 1.11030826 | 0.231883282 | 4.788220385 | 1.68E-06 | 9.58E-05 |
| DPP10 | 497.3017844 | 1.670360694 | 0.509932146 | 3.275652863 | 0.00105418 | 0.010570267 |
| SGCG | 36.89800344 | 2.311205729 | 0.418892295 | 5.51742239 | 3.44E-08 | 4.97E-06 |
| AC006445.8 | 14.08523463 | -1.006409935 | 0.366551915 | -2.745613632 | 0.006039785 | 0.036742809 |
| ASB16 | 289.9974731 | 1.080507489 | 0.248979834 | 4.339738971 | 1.43E-05 | 0.000468637 |
| RP11-112L18.1 | 24.12946382 | -1.55471793 | 0.436824537 | -3.559135983 | 0.000372077 | 0.005001426 |
| CTD-2183H9.3 | 8.763221286 | 1.283682993 | 0.487157466 | 2.635047354 | 0.008412555 | 0.046498592 |
| GALR3 | 8.446028269 | 1.894109195 | 0.710763585 | 2.664893413 | 0.007701273 | 0.043668915 |
| BEND2 | 3.132383861 | 2.696405692 | 1.022807421 | 2.636278969 | 0.008382079 | 0.046363002 |
| FBXW12 | 164.8407389 | 1.278641939 | 0.355341689 | 3.598344859 | 0.000320249 | 0.004488051 |
| RP11-81H3.2 | 12.59097241 | -2.57670343 | 0.556127123 | -4.633299336 | 3.60E-06 | 0.000170726 |
| ZNF385D | 745.1410098 | 1.055209682 | 0.262939691 | 4.01312437 | 5.99E-05 | 0.001368029 |
| NTRK2 | 7067.370818 | 1.259391715 | 0.25328017 | 4.972326559 | 6.62E-07 | 4.79E-05 |
| CYP11A1 | 77.03096013 | 1.316163165 | 0.296680537 | 4.436297642 | 9.15E-06 | 0.000339159 |
| RAB26 | 303.0049374 | 1.07709201 | 0.274590337 | 3.922541561 | 8.76E-05 | 0.001799782 |
| CETP | 309.0122564 | 1.498268479 | 0.320409013 | 4.676112148 | 2.92E-06 | 0.000145498 |
| MAL2 | 11309.36298 | -1.083833743 | 0.322140774 | -3.364472404 | 0.000766902 | 0.008393299 |
| NRG3-AS1 | 26.02771839 | -1.312851128 | 0.420125687 | -3.124900879 | 0.00177865 | 0.015277192 |
| RNY4P20 | 6.363551578 | 1.236784072 | 0.353856589 | 3.49515626 | 0.000473784 | 0.005919647 |
| TMC7 | 1027.460485 | -1.060708646 | 0.227487884 | -4.662703899 | 3.12E-06 | 0.000152885 |
| TMPRSS15 | 24.91283428 | 3.862637748 | 1.202645467 | 3.211784231 | 0.001319134 | 0.01236827 |
| TMEM52 | 704.672873 | 1.702158884 | 0.483245691 | 3.522346741 | 0.000427744 | 0.005504914 |
| IGKV1OR-2 | 67.77766156 | -1.120823257 | 0.307651966 | -3.64315324 | 0.000269318 | 0.003966039 |
| AC025280.1 | 24.23611294 | 1.285098057 | 0.27266934 | 4.713027349 | 2.44E-06 | 0.000127577 |
| AC073850.6 | 48.8117135 | 2.109133474 | 0.318512375 | 6.621825839 | 3.55E-11 | 2.10E-08 |
| SLC25A25 | 4108.083206 | 1.118139549 | 0.207621654 | 5.385466905 | 7.23E-08 | 8.51E-06 |
| KARSP2 | 16.86476084 | 1.268810916 | 0.437391474 | 2.900858824 | 0.003721415 | 0.026109496 |
| RP11-95P9.1 | 26.65753797 | 1.004331791 | 0.284487705 | 3.53031704 | 0.000415062 | 0.005389724 |
| NR4A3 | 6838.181135 | 1.988450985 | 0.342201885 | 5.810754033 | 6.22E-09 | 1.26E-06 |
| ANO1 | 10035.19673 | -1.008494197 | 0.221610318 | -4.550754704 | 5.35E-06 | 0.000232119 |
| AC068896.1 | 3.355507603 | 1.261000645 | 0.375178811 | 3.361065728 | 0.000776423 | 0.008468988 |
| ZBBX | 40.44807333 | -1.985121614 | 0.495686286 | -4.004794307 | 6.21E-05 | 0.001397504 |
| RP1-193H18.3 | 51.20646191 | 1.482946954 | 0.330747779 | 4.483618788 | 7.34E-06 | 0.000287667 |
| RP11-798K3.2 | 40.02754876 | 1.711627144 | 0.424287373 | 4.034122276 | 5.48E-05 | 0.00128824 |
| MYOC | 70.4630847 | 1.766483148 | 0.584161844 | 3.023961881 | 0.002494879 | 0.019551406 |
| SMCO2 | 46.75234112 | -1.206585225 | 0.274468599 | -4.396077469 | 1.10E-05 | 0.000387226 |
| CEBPA-AS1 | 153.4666295 | 1.083179457 | 0.214486902 | 5.050096048 | 4.42E-07 | 3.52E-05 |
| ARL5C | 33.11892932 | 1.266183748 | 0.311225098 | 4.068385731 | 4.73E-05 | 0.00116102 |
| AL391416.1 | 3.327495791 | 1.196301302 | 0.351626633 | 3.402191959 | 0.000668477 | 0.007604215 |
| KLF4 | 5191.871129 | 1.151017229 | 0.255747962 | 4.50059198 | 6.78E-06 | 0.00027214 |
| HOGA1 | 771.9874197 | 1.271190715 | 0.344424515 | 3.690767239 | 0.000223579 | 0.003490172 |
| SERBP1P6 | 66.65096202 | 1.209082796 | 0.199189575 | 6.070010435 | 1.28E-09 | 3.75E-07 |
| PCDHGA1 | 104.6678044 | -1.014573697 | 0.23052565 | -4.401131482 | 1.08E-05 | 0.00038037 |
| C1QTNF1-AS1 | 42.52253188 | 1.032600618 | 0.223831449 | 4.613295505 | 3.96E-06 | 0.000184648 |
| RP11-346I3.2 | 8.475445002 | 1.289316163 | 0.466985492 | 2.76093409 | 0.005763631 | 0.035573406 |
| ABCA4 | 71.65208106 | 1.089747748 | 0.265256355 | 4.108281396 | 3.99E-05 | 0.001020615 |
| EMCN | 1570.270591 | 1.052170424 | 0.210410425 | 5.000562242 | 5.72E-07 | 4.34E-05 |
| FOXH1 | 35.86331156 | -1.365120237 | 0.421309131 | -3.240186688 | 0.001194515 | 0.011544015 |
| RAC1P7 | 12.23674232 | -1.536377472 | 0.514821769 | -2.984290027 | 0.002842372 | 0.021542916 |
| RNU7-159P | 11.85724684 | -1.858104264 | 0.447242907 | -4.154575142 | 3.26E-05 | 0.000876896 |
| CTD-2258A20.3 | 11.31446423 | -1.553136781 | 0.508501317 | -3.054341706 | 0.00225555 | 0.018203765 |
| CEACAM22P | 16.24937723 | 1.003513165 | 0.324390332 | 3.093535979 | 0.001977866 | 0.016477091 |
| RP11-1260E13.1 | 52.21716944 | 1.022545332 | 0.270630556 | 3.778380937 | 0.000157851 | 0.002737898 |
| TRBV11-2 | 87.37144997 | 1.30108376 | 0.358213863 | 3.6321424 | 0.000281078 | 0.004100524 |
| F10 | 704.3411434 | 1.488660538 | 0.290787672 | 5.119407327 | 3.06E-07 | 2.64E-05 |
| RP11-124N14.3 | 342.1317338 | 1.075406694 | 0.225348839 | 4.772186525 | 1.82E-06 | 0.000101664 |
| RP1-106C24.1 | 6.887821231 | 1.672085001 | 0.473107874 | 3.534257392 | 0.000408923 | 0.005331307 |
| CTD-2171N6.1 | 50.4693734 | -1.375839264 | 0.362723859 | -3.793076274 | 0.000148792 | 0.002633754 |
| AC006262.6 | 188.6281651 | -1.151246172 | 0.392409067 | -2.933790959 | 0.003348496 | 0.02424792 |
| LINC01070 | 2.475109868 | 3.458142919 | 1.124559308 | 3.075109419 | 0.002104253 | 0.01728147 |
| MC5R | 8.30867108 | -1.480795524 | 0.560365158 | -2.642554597 | 0.00822832 | 0.045755995 |
| G0S2 | 2751.467756 | 1.800474235 | 0.346330945 | 5.198710255 | 2.01E-07 | 1.96E-05 |
| PADI4 | 51.81726827 | 2.262210601 | 0.371245302 | 6.093573678 | 1.10E-09 | 3.31E-07 |
| ZNF804B | 35.32026893 | 1.599469457 | 0.60433929 | 2.646641521 | 0.008129548 | 0.045371651 |
| CASC9 | 41.24649142 | -1.855912667 | 0.667879444 | -2.778813877 | 0.005455778 | 0.034165416 |
| TMED11P | 554.7834711 | 1.169172142 | 0.43989207 | 2.657861375 | 0.007863823 | 0.044371261 |
| BPIFB1 | 264.7479879 | -2.288206001 | 0.502664083 | -4.552157354 | 5.31E-06 | 0.000230834 |
| NTF4 | 55.82866203 | -1.7244491 | 0.34999028 | -4.927134269 | 8.34E-07 | 5.67E-05 |
| ACVR1C | 890.1749853 | 1.956829031 | 0.279675679 | 6.996779393 | 2.62E-12 | 2.08E-09 |
| FZD4 | 8011.785065 | 1.16277058 | 0.180671786 | 6.435817154 | 1.23E-10 | 5.56E-08 |
| UPK1B | 855.54618 | -1.103090373 | 0.396055022 | -2.785194764 | 0.005349558 | 0.033684162 |
| RNVU1-6 | 15.08970115 | 1.036889433 | 0.3102111 | 3.342528474 | 0.000830188 | 0.008874231 |
| TAC1 | 34.5187699 | 1.246972045 | 0.374177351 | 3.332569544 | 0.00086048 | 0.009125483 |
| RDH5 | 408.1534234 | 1.116080131 | 0.221827199 | 5.031304263 | 4.87E-07 | 3.80E-05 |
| SLC39A5 | 3538.009426 | 2.318083344 | 0.491023099 | 4.720925248 | 2.35E-06 | 0.000123716 |
| POF1B | 4451.25979 | -1.030231291 | 0.295563255 | -3.485654164 | 0.000490935 | 0.006070663 |
| CTB-174D11.2 | 21.83242459 | 1.643108645 | 0.313428576 | 5.242370265 | 1.59E-07 | 1.61E-05 |
| HSPD1P16 | 19.72143008 | 1.284552102 | 0.378232507 | 3.396196991 | 0.000683292 | 0.007735952 |
| SCGB3A2 | 21.7752011 | -1.21813453 | 0.401274052 | -3.03566733 | 0.00240004 | 0.019026862 |
| RP5-1063M23.1 | 116.6032693 | 1.140299163 | 0.30309332 | 3.762204866 | 0.000168422 | 0.002867955 |
| MIR455 | 4.116486059 | 1.162022438 | 0.336944578 | 3.448704964 | 0.000563282 | 0.006711748 |
| NPY4R | 139.6380052 | -1.368111967 | 0.307654503 | -4.446910255 | 8.71E-06 | 0.000326951 |
| RP11-839G9.1 | 7.494425796 | -1.29723555 | 0.318731882 | -4.069989927 | 4.70E-05 | 0.001158153 |
| MROH3P | 70.21233176 | -1.199510188 | 0.364678947 | -3.289222471 | 0.001004646 | 0.010212764 |
| MTND1P36 | 74.16560061 | -1.059002946 | 0.245300348 | -4.317168545 | 1.58E-05 | 0.000503917 |
| RNVU1-13 | 72.39168639 | 1.287125244 | 0.367684571 | 3.500623483 | 0.000464171 | 0.005833762 |
| RNU1-34P | 9.11079539 | 1.461558266 | 0.343071345 | 4.260216686 | 2.04E-05 | 0.000614973 |
| LMO3 | 1874.243435 | 1.240671103 | 0.270010543 | 4.59489873 | 4.33E-06 | 0.000197637 |
| ANK2 | 5883.995393 | 1.622754155 | 0.279206192 | 5.812027819 | 6.17E-09 | 1.26E-06 |
| RP11-313F23.4 | 8.064180868 | 1.165532859 | 0.408118014 | 2.855872126 | 0.00429188 | 0.028900335 |
| MMRN1 | 2558.83378 | 1.040514642 | 0.275136225 | 3.781816232 | 0.000155688 | 0.002712719 |
| FAM3D | 729.425385 | -1.386962011 | 0.447576157 | -3.098829076 | 0.001942871 | 0.01627196 |
| CLCNKB | 52.41219539 | 1.013790412 | 0.378880169 | 2.675754751 | 0.007456118 | 0.042613416 |
| RP11-613D13.8 | 169.5514811 | 1.44012442 | 0.270425162 | 5.325408368 | 1.01E-07 | 1.12E-05 |
| RPSAP52 | 52.10026384 | -1.331374927 | 0.475437664 | -2.800314377 | 0.005105286 | 0.03268271 |
| IGSF10 | 1123.102893 | 1.663811124 | 0.31776633 | 5.235957891 | 1.64E-07 | 1.65E-05 |
| AMHR2 | 93.48581348 | 1.439694121 | 0.478618538 | 3.008019972 | 0.002629559 | 0.0203089 |
| LOXHD1 | 173.625769 | 1.021488828 | 0.234295068 | 4.359839226 | 1.30E-05 | 0.000436791 |
| CTD-2033D15.1 | 12.91463859 | 1.366721619 | 0.410729133 | 3.327549742 | 0.000876133 | 0.00923627 |
| PBK | 287.6948738 | -1.210272147 | 0.252373851 | -4.795552879 | 1.62E-06 | 9.37E-05 |
| GDF10 | 195.5140318 | 1.402416676 | 0.342583 | 4.093655194 | 4.25E-05 | 0.001071733 |
| CDKN2B-AS1 | 168.501882 | -1.225710048 | 0.217061573 | -5.646831127 | 1.63E-08 | 2.82E-06 |
| ZG16 | 82.77869031 | 2.04846968 | 0.585712139 | 3.497400075 | 0.000469817 | 0.005887051 |
| BRINP2 | 20.7200732 | 1.215270822 | 0.389414206 | 3.120766538 | 0.00180381 | 0.015424657 |
| ST6GALNAC1 | 1437.974998 | -1.118534023 | 0.326690247 | -3.423836597 | 0.000617437 | 0.007191448 |
| RP11-284F21.10 | 541.3108229 | -1.185937808 | 0.310349227 | -3.821300988 | 0.00013275 | 0.002444017 |
| GOLGA8M | 148.8708413 | 1.226106698 | 0.217242284 | 5.643959705 | 1.66E-08 | 2.85E-06 |
| SMPX | 14.367888 | -2.070936049 | 0.512802779 | -4.038464952 | 5.38E-05 | 0.00127384 |
| GNMT | 2324.741149 | 1.86738458 | 0.517052182 | 3.611597911 | 0.000304316 | 0.004336006 |
| WISP3 | 77.93690995 | -1.287868753 | 0.336210655 | -3.830541156 | 0.000127862 | 0.002377667 |
| MIR216A | 4.605867952 | 1.449933839 | 0.517416565 | 2.802256319 | 0.005074654 | 0.032541707 |
| AP001604.3 | 12.83940584 | 1.540475241 | 0.394421033 | 3.905661999 | 9.40E-05 | 0.001894661 |
| FEN1P1 | 10.16286878 | 1.087050199 | 0.366352909 | 2.967221419 | 0.003005045 | 0.022454468 |
| GYS2 | 25.57479786 | 1.176165031 | 0.245604676 | 4.788854383 | 1.68E-06 | 9.58E-05 |
| RP11-160H22.3 | 4.896209447 | 1.277258512 | 0.444904558 | 2.87085958 | 0.004093573 | 0.027887646 |
| RASIP1 | 1121.291133 | 1.387834361 | 0.194072327 | 7.151119281 | 8.61E-13 | 8.19E-10 |
| RP11-380J14.1 | 38.90939136 | -2.389853684 | 0.679680227 | -3.516144193 | 0.000437863 | 0.005589025 |
| RP5-884M6.1 | 143.3109069 | -2.321208308 | 0.44757316 | -5.186209793 | 2.15E-07 | 2.07E-05 |
| AKR1C5P | 3.986875369 | 1.521630225 | 0.502703257 | 3.026895494 | 0.002470794 | 0.01941756 |
| AQP5 | 1487.73643 | -1.6711728 | 0.443695834 | -3.766482966 | 0.000165563 | 0.002832885 |
| MIR4260 | 12.47291587 | -1.093777094 | 0.341737407 | -3.200636138 | 0.001371246 | 0.012700583 |
| RP11-554A11.9 | 63.24930793 | 1.236837996 | 0.310781916 | 3.979761788 | 6.90E-05 | 0.001502403 |
| MGAM | 575.3570161 | 1.071557342 | 0.295855529 | 3.621893923 | 0.000292454 | 0.004222231 |
| CAPN9 | 472.1806113 | -1.239471244 | 0.438113287 | -2.829111284 | 0.004667747 | 0.030673875 |
| AC090103.1 | 77.46048726 | 1.352112976 | 0.295917647 | 4.569220488 | 4.90E-06 | 0.000217353 |
| RNA5SP156 | 20.24207878 | -1.079964135 | 0.320343891 | -3.371264964 | 0.000748239 | 0.008234403 |
| PGM5-AS1 | 20.55572375 | 1.449197173 | 0.494275372 | 2.931963142 | 0.003368267 | 0.024354827 |
| MIR487A | 2.465593487 | 1.088038436 | 0.387731611 | 2.806163867 | 0.005013519 | 0.032261084 |
| MIR4322 | 4.755540507 | 1.028578516 | 0.274731526 | 3.743940611 | 0.000181156 | 0.00301928 |
| RP11-703I16.1 | 71.80234253 | 1.178564841 | 0.331702239 | 3.553080749 | 0.000380748 | 0.005082934 |
| ALLC | 21.21594909 | 1.197964876 | 0.450186244 | 2.661042826 | 0.007789905 | 0.044075354 |
| CSTB | 4740.907631 | -1.226683419 | 0.182228803 | -6.731556131 | 1.68E-11 | 1.11E-08 |
| DNAJB13 | 193.9436357 | -1.389591209 | 0.3313824 | -4.193316266 | 2.75E-05 | 0.000770665 |
| AC006033.22 | 12.00328602 | 1.778755747 | 0.509695717 | 3.489838519 | 0.000483312 | 0.006011729 |
| CTD-2183H9.7 | 5.143010087 | 1.337262368 | 0.447265192 | 2.989864607 | 0.002791011 | 0.021256931 |
| CA4 | 390.5052269 | 1.434911374 | 0.390020167 | 3.67906969 | 0.000234086 | 0.003608313 |
| LL22NC03-N64E9.1 | 128.5508771 | -1.023647036 | 0.234242772 | -4.370026129 | 1.24E-05 | 0.000421987 |
| PLA2G5 | 118.7591781 | 1.336139991 | 0.313172222 | 4.266470325 | 1.99E-05 | 0.00060171 |
| CORO7-PAM16 | 72.2086778 | -1.242653408 | 0.427674312 | -2.905606845 | 0.003665417 | 0.025870213 |
| RERGL | 124.1134343 | 1.70895699 | 0.282690565 | 6.045327294 | 1.49E-09 | 4.19E-07 |
| RP11-544M22.8 | 44.90268125 | -1.086654126 | 0.366634567 | -2.963861633 | 0.003038049 | 0.022625411 |
| SLC7A10 | 132.2339088 | 3.520011337 | 0.435746209 | 8.078122679 | 6.58E-16 | 2.14E-12 |
| LGALS12 | 174.6630889 | 3.206559992 | 0.438620349 | 7.310559117 | 2.66E-13 | 3.46E-10 |
| ADAM33 | 1512.952076 | 1.107985231 | 0.247741306 | 4.472347581 | 7.74E-06 | 0.000299031 |
| AL138751.1 | 11.95044566 | 1.023572131 | 0.322945325 | 3.169490478 | 0.001527065 | 0.013765114 |
| NKX6-2 | 15.55721805 | 1.960095832 | 0.58129425 | 3.371951181 | 0.000746377 | 0.008217098 |
| C1orf106 | 4656.476949 | -1.079924851 | 0.301581961 | -3.580866866 | 0.000342456 | 0.004712877 |
| RNASE1 | 19907.96776 | 1.326942589 | 0.297701781 | 4.457288038 | 8.30E-06 | 0.000315355 |
| TFAP2A | 853.0567779 | -1.554051083 | 0.313837772 | -4.951765597 | 7.35E-07 | 5.20E-05 |
| CD300LG | 291.235839 | 3.652103791 | 0.469053704 | 7.786110112 | 6.91E-15 | 1.42E-11 |
| APOBEC3A | 106.8862325 | 1.123777852 | 0.353849274 | 3.175865923 | 0.0014939 | 0.013541288 |
| AC006262.5 | 76.8171505 | -1.301251329 | 0.438034538 | -2.970659196 | 0.002971613 | 0.022286757 |
| MESP1 | 108.5918856 | 1.307208768 | 0.259805848 | 5.03148323 | 4.87E-07 | 3.80E-05 |
| DPT | 3029.985754 | 1.695177434 | 0.311856759 | 5.435756591 | 5.46E-08 | 7.05E-06 |
| CLEC4G | 242.3107598 | 1.922914279 | 0.614190861 | 3.130809004 | 0.001743255 | 0.015088452 |
| TDRD5 | 98.26505868 | -1.234206606 | 0.428563409 | -2.879869303 | 0.003978401 | 0.027373289 |
| IDO1 | 954.7870686 | -1.400430376 | 0.342194714 | -4.092495641 | 4.27E-05 | 0.001076411 |
| RP11-863K10.7 | 36.21382952 | 1.083234957 | 0.226213729 | 4.78854649 | 1.68E-06 | 9.58E-05 |
| HIST1H1T | 21.47555824 | -1.195794218 | 0.353151951 | -3.386061478 | 0.000709035 | 0.00793225 |
| TMEM72 | 151.7753481 | 1.214789803 | 0.327307562 | 3.711462691 | 0.000206065 | 0.003295942 |
| RP11-1134I14.2 | 2.630933855 | -2.533540243 | 0.825813462 | -3.067932845 | 0.002155451 | 0.017613536 |
| CXorf61 | 46.7308014 | -2.564774382 | 0.808268647 | -3.173170691 | 0.001507838 | 0.013633807 |
| FOXC2 | 385.8766805 | -1.218165762 | 0.3481262 | -3.499207366 | 0.000466644 | 0.005858585 |
| FXYD1 | 627.7568036 | 1.790964826 | 0.276165266 | 6.485119779 | 8.87E-11 | 4.32E-08 |
| TDRD10 | 196.350044 | 1.298368078 | 0.198391782 | 6.544465029 | 5.97E-11 | 3.21E-08 |
| MIR31HG | 172.8093455 | -1.728954411 | 0.468478498 | -3.690573668 | 0.000223749 | 0.003490707 |
| TF | 484.4997976 | 1.963572499 | 0.299495358 | 6.556270217 | 5.52E-11 | 3.07E-08 |
| AC012512.1 | 17.27066875 | -1.363181691 | 0.404768014 | -3.367809819 | 0.000757678 | 0.008313357 |
| TRBV7-3 | 65.41220843 | 1.026695178 | 0.329439762 | 3.116488347 | 0.001830189 | 0.01557807 |
| LGR5 | 329.7758052 | -1.158558157 | 0.290208956 | -3.99215163 | 6.55E-05 | 0.001444937 |
| RNVU1-15 | 58.40742861 | 1.171365191 | 0.236946293 | 4.943589436 | 7.67E-07 | 5.35E-05 |
| CTD-2363C16.1 | 98.24931628 | 1.101334266 | 0.257555596 | 4.276103037 | 1.90E-05 | 0.000583065 |
| RP11-58O3.1 | 2.829891838 | 2.761169357 | 0.816103385 | 3.383357313 | 0.000716054 | 0.007986993 |
| NRG3 | 356.6318496 | -1.032597085 | 0.33696295 | -3.064423214 | 0.002180902 | 0.017765668 |
| CTD-2015G9.2 | 32.25385366 | -1.628549539 | 0.507652703 | -3.207999344 | 0.001336618 | 0.012463594 |
| KCNB1 | 574.5495054 | 1.995810758 | 0.301941484 | 6.609925652 | 3.85E-11 | 2.24E-08 |
| TNXA | 244.6790946 | 2.245253735 | 0.457561862 | 4.90699492 | 9.25E-07 | 6.09E-05 |
| C5orf27 | 55.85844202 | 1.301350216 | 0.231543518 | 5.620326694 | 1.91E-08 | 3.20E-06 |
| LRRC2 | 362.6958081 | 1.284751417 | 0.237974759 | 5.398687763 | 6.71E-08 | 8.07E-06 |
| PENK | 239.9237942 | 1.836160046 | 0.44441678 | 4.131617275 | 3.60E-05 | 0.000941635 |
| EPHX1 | 7261.489394 | 1.323568048 | 0.224919603 | 5.884627343 | 3.99E-09 | 9.04E-07 |
| RP5-1070A16.1 | 14.23475458 | -2.374273624 | 0.79530776 | -2.985352014 | 0.002832522 | 0.021488997 |
| RP11-550F7.1 | 42.31423237 | 1.509611458 | 0.309806624 | 4.87275397 | 1.10E-06 | 6.94E-05 |
| ANK1 | 807.1073109 | -1.398048269 | 0.281014193 | -4.97500947 | 6.52E-07 | 4.75E-05 |
| CTD-2366F13.2 | 56.3541025 | -1.142494598 | 0.27147933 | -4.208403632 | 2.57E-05 | 0.000732028 |
| SERPINB5 | 2508.323041 | -1.563587336 | 0.428660541 | -3.647612007 | 0.000264689 | 0.003924989 |
| FUT1 | 824.1149818 | 1.151618725 | 0.268539295 | 4.288455161 | 1.80E-05 | 0.000557266 |
| GPX2 | 2939.402691 | -1.023765848 | 0.315679773 | -3.243051779 | 0.001182567 | 0.011479763 |
| COCH | 608.2269748 | 1.213302638 | 0.29384859 | 4.129006161 | 3.64E-05 | 0.000950316 |
| PLEKHN1 | 329.7124898 | -1.049565233 | 0.245770113 | -4.270516141 | 1.95E-05 | 0.000594593 |
| DLGAP5 | 738.6928103 | -1.010580707 | 0.244608344 | -4.131423687 | 3.61E-05 | 0.000941635 |
| SYPL2 | 170.1056943 | 1.094965264 | 0.283616236 | 3.860728426 | 0.000113049 | 0.002185605 |
| RP11-298O21.5 | 15.14316182 | 1.418186799 | 0.351315891 | 4.036785225 | 5.42E-05 | 0.001279108 |
| AC004870.4 | 74.46589139 | -1.234764292 | 0.433288637 | -2.849749997 | 0.004375361 | 0.029315667 |
| AC112229.6 | 11.96072521 | -1.036779529 | 0.359560923 | -2.883459972 | 0.003933326 | 0.027151719 |
| HTR4 | 17.36370226 | 1.382886068 | 0.440243841 | 3.141182088 | 0.001682674 | 0.014705483 |
| EVX1 | 25.16426829 | -1.845742748 | 0.638481588 | -2.890831596 | 0.003842239 | 0.026716856 |
| CELA2A | 260938.9033 | 2.246654348 | 0.685189722 | 3.278879228 | 0.001042202 | 0.010490624 |
| ADCYAP1R1 | 320.7710355 | 1.516536302 | 0.273098032 | 5.553083971 | 2.81E-08 | 4.34E-06 |
| RP4-594A5.1 | 2.558289954 | -4.147854081 | 1.495658956 | -2.773261956 | 0.005549743 | 0.034614878 |
| GPR62 | 37.65229058 | 1.02360677 | 0.29079131 | 3.52007345 | 0.000431427 | 0.005537692 |
| GDPD2 | 59.33413341 | -1.104517325 | 0.369931853 | -2.985731876 | 0.002829006 | 0.021475 |
| ITGA7 | 2549.796579 | 1.609781179 | 0.248401905 | 6.480550872 | 9.14E-11 | 4.40E-08 |
| C16orf96 | 53.87753689 | 1.122123782 | 0.277194071 | 4.048152184 | 5.16E-05 | 0.001237284 |
| RP11-61I13.3 | 407.5479428 | 1.140773772 | 0.167306602 | 6.818462393 | 9.20E-12 | 6.41E-09 |
| GPD1 | 2824.520503 | 3.402067999 | 0.394618157 | 8.621164382 | 6.63E-18 | 8.61E-14 |
| RNA5SP300 | 3.73322651 | 1.329485885 | 0.441666266 | 3.010159454 | 0.002611106 | 0.020202394 |
| RP11-700H6.4 | 26.20892422 | 1.741608726 | 0.4402371 | 3.956069863 | 7.62E-05 | 0.001621802 |
| RSPO2 | 45.37446641 | 1.01247291 | 0.335050537 | 3.021851328 | 0.002512339 | 0.019648749 |
| AC004019.18 | 11.76755836 | 1.035679013 | 0.234332842 | 4.419692119 | 9.88E-06 | 0.000359546 |
| RP11-717H13.1 | 3.959723774 | 3.273949909 | 1.02912643 | 3.18129028 | 0.001466206 | 0.013336766 |
| RN7SL411P | 7.410148635 | 1.384531866 | 0.348266532 | 3.975495034 | 7.02E-05 | 0.001523213 |
| TRBV21-1 | 21.7475016 | 1.107339831 | 0.418653565 | 2.645002753 | 0.008169025 | 0.045520311 |
| RP11-438N16.1 | 10.05901326 | -2.429143486 | 0.680919999 | -3.567443297 | 0.000360481 | 0.004887681 |
| IGLV7-46 | 589.7687661 | -1.17405174 | 0.409199836 | -2.869140296 | 0.004115892 | 0.028029899 |
| KRT19 | 29658.68972 | -1.282535594 | 0.346150216 | -3.705141687 | 0.000211273 | 0.003364057 |
| IBSP | 62.16489726 | -1.201557036 | 0.453773581 | -2.647921971 | 0.008098822 | 0.045251977 |
| RP11-435B5.3 | 12.47493172 | 1.314599826 | 0.497751679 | 2.641075623 | 0.008264327 | 0.045909731 |
| SOSTDC1 | 79.07381152 | -1.717024949 | 0.418409143 | -4.10369844 | 4.07E-05 | 0.00103359 |
| RP11-1260E13.2 | 16.73364739 | 1.053080281 | 0.285362766 | 3.690321254 | 0.000223971 | 0.003490707 |
| MUC5B | 34107.81109 | -1.697524532 | 0.452222514 | -3.753737327 | 0.000174217 | 0.002938412 |
| CTD-2354A18.1 | 33.0376123 | 1.194974931 | 0.290500267 | 4.113507169 | 3.90E-05 | 0.00100238 |
| RP11-830F9.7 | 193.196143 | 1.056617163 | 0.248154654 | 4.25789783 | 2.06E-05 | 0.000619472 |
| RP11-64C12.3 | 128.051211 | 1.420192966 | 0.504849788 | 2.813100055 | 0.004906638 | 0.031830703 |
| MTND4P20 | 218.5493251 | -1.467086251 | 0.523423751 | -2.802865264 | 0.005065082 | 0.032496362 |
| SYBU | 2841.640398 | 1.010280592 | 0.342536826 | 2.949407231 | 0.003183841 | 0.023398774 |
| PRKAR2B | 3581.5525 | 2.008682765 | 0.265424839 | 7.56780251 | 3.80E-14 | 6.17E-11 |
| CTC-232P5.3 | 72.22573084 | 1.462801159 | 0.262046966 | 5.582209871 | 2.37E-08 | 3.78E-06 |
| MTND2P11 | 58.05805474 | 1.13042653 | 0.370896767 | 3.047819851 | 0.002305081 | 0.018474988 |
| AP1S3 | 1107.852743 | -1.254328295 | 0.216122354 | -5.803787868 | 6.48E-09 | 1.30E-06 |
| AC131097.3 | 235.155659 | 1.003395824 | 0.250284316 | 4.009023981 | 6.10E-05 | 0.001383091 |
| PHYHD1 | 631.2301422 | 1.044162884 | 0.202034205 | 5.168248037 | 2.36E-07 | 2.18E-05 |
| NAV2-AS5 | 138.6796352 | 1.531193372 | 0.261839477 | 5.847832388 | 4.98E-09 | 1.05E-06 |
| E2F7 | 389.5215214 | -1.121543528 | 0.255388183 | -4.391524751 | 1.13E-05 | 0.000392947 |
| PPP2R2C | 466.8359326 | -1.491795368 | 0.379823106 | -3.927605621 | 8.58E-05 | 0.001770161 |
| CGB8 | 29.77089698 | -2.272073556 | 0.438616284 | -5.18009394 | 2.22E-07 | 2.10E-05 |
| CTD-2337J16.1 | 46.58204817 | 1.411567299 | 0.344600327 | 4.096244805 | 4.20E-05 | 0.001060507 |
| PFKFB1 | 202.9984888 | 2.686644693 | 0.334240155 | 8.038066801 | 9.13E-16 | 2.37E-12 |
| RP11-344G13.1 | 4.550221106 | 2.042146548 | 0.712825669 | 2.864861126 | 0.004171922 | 0.028297805 |
| PLTP | 7908.414496 | 1.256475239 | 0.200928969 | 6.253330439 | 4.02E-10 | 1.37E-07 |
| CDON | 3147.603503 | 1.155053791 | 0.197334914 | 5.853266235 | 4.82E-09 | 1.02E-06 |
| C15orf48 | 1178.632469 | -1.037619717 | 0.335749995 | -3.090453407 | 0.001998512 | 0.016595988 |
| NFATC1 | 2520.147849 | 1.02648058 | 0.206921369 | 4.960727766 | 7.02E-07 | 5.02E-05 |
| HSPB6 | 2773.862863 | 1.63757212 | 0.276232045 | 5.928248193 | 3.06E-09 | 7.37E-07 |
| QRFP | 23.56460329 | 1.600128525 | 0.295905059 | 5.407574075 | 6.39E-08 | 7.81E-06 |
| OR2S1P | 3.761263514 | 1.873358488 | 0.618167839 | 3.030501381 | 0.002441481 | 0.01926068 |
| CTD-2369P2.12 | 3.371892826 | 2.965626977 | 1.069021455 | 2.774151036 | 0.005534598 | 0.034536987 |
| ODAM | 94.40244152 | -1.638065767 | 0.491357688 | -3.333754221 | 0.000856823 | 0.009096604 |
| TUBA8P2 | 12.45706017 | 1.518853663 | 0.449847296 | 3.376376109 | 0.000734474 | 0.008109394 |
| RP11-374A22.1 | 4.481857582 | 1.786263626 | 0.686431995 | 2.602244125 | 0.00926159 | 0.049787957 |
| AC006486.9 | 2.621048311 | 1.298318878 | 0.468206299 | 2.772963287 | 0.005554839 | 0.034635583 |
| CDY5P | 10.88399312 | -3.72423637 | 1.263735652 | -2.947005859 | 0.003208671 | 0.02351941 |
| DUSP1 | 32369.32693 | 1.247394883 | 0.229610282 | 5.432661268 | 5.55E-08 | 7.08E-06 |
| ALPL | 899.5751083 | 1.30760767 | 0.260714412 | 5.015479048 | 5.29E-07 | 4.08E-05 |
| LEPR | 4327.187571 | 1.112239469 | 0.250688546 | 4.436738282 | 9.13E-06 | 0.000339159 |
| KRT6C | 80.61301341 | -2.602654519 | 0.566913723 | -4.590918183 | 4.41E-06 | 0.000200332 |
| PLA2G2A | 2475.907843 | 1.753304135 | 0.445678877 | 3.934007702 | 8.35E-05 | 0.001738359 |
| CASQ2 | 303.0484281 | 2.772148622 | 0.383137889 | 7.235381058 | 4.64E-13 | 4.76E-10 |
| RP11-982M15.6 | 38.9182454 | -1.355229008 | 0.328966058 | -4.119662122 | 3.79E-05 | 0.00098311 |
| C1orf95 | 323.6561223 | 1.037764825 | 0.277187588 | 3.743907983 | 0.00018118 | 0.00301928 |
| C10orf105 | 150.9597439 | 1.186732636 | 0.312834355 | 3.793485649 | 0.000148547 | 0.002630608 |
| RNU6-2 | 5.959703204 | 1.242619768 | 0.452254226 | 2.747613389 | 0.006003075 | 0.036570833 |
| NAV2-IT1 | 20.12252813 | 1.300361432 | 0.322750312 | 4.02900131 | 5.60E-05 | 0.001307951 |
| RP11-811P12.3 | 21.99278904 | 1.313305605 | 0.361203359 | 3.635917472 | 0.000276993 | 0.004057604 |
| CTSV | 178.4220649 | -1.10318791 | 0.326147113 | -3.382485593 | 0.00071833 | 0.008000941 |
| CDKN2A | 676.6093215 | -1.375367826 | 0.294068644 | -4.677029856 | 2.91E-06 | 0.000145139 |
| RP11-138H8.6 | 13.20402379 | 1.167243932 | 0.313419924 | 3.724217392 | 0.000195922 | 0.003171432 |
| ITGA3 | 14406.13146 | -1.20669853 | 0.261912085 | -4.607265564 | 4.08E-06 | 0.000188954 |
| VGLL1 | 144.1980102 | -1.55388902 | 0.566374807 | -2.743570157 | 0.006077506 | 0.036897454 |
| AC068134.5 | 19.66799264 | -1.829703833 | 0.675972157 | -2.706773963 | 0.00679405 | 0.040056543 |
| ASIP | 18.47834281 | 1.001055681 | 0.251635625 | 3.978195381 | 6.94E-05 | 0.001509866 |
| TRBV8-2 | 26.16344039 | 2.034789965 | 0.596993364 | 3.408396285 | 0.000653459 | 0.007479202 |
| ABAT | 4512.828909 | 1.793894899 | 0.346284489 | 5.180407888 | 2.21E-07 | 2.10E-05 |
| DUSP8P4 | 27.79600385 | 1.029070872 | 0.34612521 | 2.973117364 | 0.002947917 | 0.022140236 |
| AC072062.3 | 9.067989002 | -1.011006675 | 0.324948235 | -3.111285324 | 0.001862749 | 0.015777125 |
| NBPF18P | 50.93868227 | -1.164094809 | 0.266365913 | -4.370284454 | 1.24E-05 | 0.000421856 |
| KCNJ5 | 1859.103139 | 1.043830908 | 0.279084861 | 3.740191799 | 0.00018388 | 0.003043462 |
| RP4-533D7.4 | 33.07907781 | 1.02633456 | 0.273534463 | 3.752121573 | 0.000175344 | 0.002953549 |
| LEMD1-AS1 | 61.97888027 | -1.481179004 | 0.412480298 | -3.590908493 | 0.000329527 | 0.004586229 |
| S100A4 | 7336.204024 | -1.050679763 | 0.26293273 | -3.99600218 | 6.44E-05 | 0.001431544 |
| WFDC10B | 14.27754915 | -1.753218836 | 0.452926321 | -3.870869839 | 0.000108448 | 0.002116575 |
| ENHO | 38.75855569 | 1.146049286 | 0.335839068 | 3.412495432 | 0.00064371 | 0.007404565 |
| GJB6 | 137.5517992 | -3.024923549 | 0.520606856 | -5.810379777 | 6.23E-09 | 1.26E-06 |
| CTD-2650P22.1 | 5.503611782 | 1.254047967 | 0.479786363 | 2.613763259 | 0.008955106 | 0.048561309 |
| CA3 | 231.4846325 | 1.047683189 | 0.261203706 | 4.010981338 | 6.05E-05 | 0.001376478 |
| FAM83B | 1435.491344 | -1.153317953 | 0.33432531 | -3.449687833 | 0.000561235 | 0.006696868 |
| TCF23 | 55.74006541 | 1.503071656 | 0.439235441 | 3.422018162 | 0.000621582 | 0.007226767 |
| CCDC3 | 2319.499727 | 1.265687544 | 0.248374239 | 5.095888967 | 3.47E-07 | 2.92E-05 |
| AC098592.7 | 18.93977945 | -1.746394224 | 0.602063024 | -2.900683408 | 0.003723499 | 0.026119415 |
| PKHD1L1 | 846.6986272 | 2.318198488 | 0.394155111 | 5.881437084 | 4.07E-09 | 9.17E-07 |
| TRBV21OR9-2 | 2.895731632 | 2.729999848 | 0.960919575 | 2.841028448 | 0.00449683 | 0.029893264 |
| CTD-2562J17.4 | 6.389342568 | 1.422902125 | 0.400854048 | 3.549676327 | 0.000385705 | 0.005135053 |
| CTC-422A18.1 | 4.782489324 | 1.180145286 | 0.423250005 | 2.788293617 | 0.00529865 | 0.0335065 |
| FER1L4 | 1529.796141 | -1.033681777 | 0.246381702 | -4.195448649 | 2.72E-05 | 0.000766001 |
| RP11-415D17.4 | 17.94462114 | 1.370033717 | 0.253440047 | 5.405750727 | 6.45E-08 | 7.86E-06 |
| HCN1 | 74.20619524 | 1.233771469 | 0.456392067 | 2.703314888 | 0.006865168 | 0.040287014 |
| MEOX2 | 977.2083813 | 1.030886459 | 0.257981023 | 3.995977864 | 6.44E-05 | 0.001431544 |
| PSCA | 4369.644104 | -2.457039395 | 0.527917313 | -4.654212574 | 3.25E-06 | 0.00015754 |
| TMEM132C | 881.2510898 | 1.590209194 | 0.348844321 | 4.558506758 | 5.15E-06 | 0.000226235 |
| EFNA3 | 204.0130564 | -1.299319223 | 0.257055648 | -5.054622353 | 4.31E-07 | 3.47E-05 |
| MAP1LC3C | 48.19242896 | 1.357805423 | 0.335554768 | 4.046449494 | 5.20E-05 | 0.00124402 |
| NRN1 | 574.3317072 | 1.362049976 | 0.277546783 | 4.907460873 | 9.23E-07 | 6.09E-05 |
| DLEU7 | 259.0297467 | -1.104972248 | 0.203463508 | -5.430812925 | 5.61E-08 | 7.13E-06 |
| MSLNL | 50.11800031 | -1.321694344 | 0.495895223 | -2.665269359 | 0.007692668 | 0.043658214 |
| CTC-558O2.1 | 101.5585415 | 1.44085447 | 0.226230379 | 6.368969886 | 1.90E-10 | 7.81E-08 |
| RP11-350N15.4 | 101.0008364 | 1.088973061 | 0.237298129 | 4.589050352 | 4.45E-06 | 0.000201663 |
| SHANK3 | 5549.679812 | 1.100508438 | 0.16083162 | 6.842612391 | 7.78E-12 | 5.51E-09 |
| METTL11B | 11.95006352 | -1.423584978 | 0.489571371 | -2.90781909 | 0.003639588 | 0.025739163 |
| RMST | 525.1128861 | -1.080693322 | 0.392844394 | -2.750945002 | 0.005942362 | 0.036303055 |
| RP11-6N13.1 | 5.250571534 | -2.003733189 | 0.676230192 | -2.963093355 | 0.003045642 | 0.022664812 |
| SNTG2 | 231.9059689 | 1.199844407 | 0.301016654 | 3.9859735 | 6.72E-05 | 0.001472259 |
| AC008753.6 | 40.18731877 | 1.28963304 | 0.25129075 | 5.132035459 | 2.87E-07 | 2.52E-05 |
| CCDC148-AS1 | 3.325548168 | -1.152090296 | 0.422400186 | -2.727485298 | 0.00638191 | 0.038245364 |
| MT1H | 587.907819 | 1.951709211 | 0.50746762 | 3.845977819 | 0.000120073 | 0.002283938 |
| RP11-125B21.2 | 117.3035609 | 1.381875725 | 0.259256356 | 5.330151764 | 9.81E-08 | 1.09E-05 |
| AC114730.2 | 23.18216499 | 1.440286185 | 0.361908707 | 3.979694756 | 6.90E-05 | 0.001502403 |
| CTC-558O19.1 | 41.89947095 | 1.299506028 | 0.288120196 | 4.510291352 | 6.47E-06 | 0.000264621 |
| MIR511-2 | 7.450376494 | 1.031182222 | 0.311015491 | 3.315533309 | 0.000914684 | 0.009538259 |
| CTD-2529O21.2 | 16.66814014 | 1.072475315 | 0.279520872 | 3.836834469 | 0.00012463 | 0.002337645 |
| LCN2 | 8902.668754 | -1.731602237 | 0.409307596 | -4.230564623 | 2.33E-05 | 0.000676839 |
| RP11-298O21.3 | 6.953855608 | 1.682331628 | 0.503875517 | 3.338784228 | 0.000841459 | 0.008969416 |
| RP11-96C23.11 | 128.0131155 | 1.239067973 | 0.473719343 | 2.615616168 | 0.008906662 | 0.048368151 |
| RP11-1101K5.1 | 12.65041639 | 2.589331362 | 0.463169788 | 5.59045825 | 2.26E-08 | 3.65E-06 |
| CTD-2175A23.1 | 41.7195558 | -1.044436529 | 0.253734862 | -4.116251583 | 3.85E-05 | 0.000995119 |
| CUX2 | 101.3640886 | 1.096805529 | 0.269708137 | 4.066638632 | 4.77E-05 | 0.001166088 |
| WNK2 | 7708.440749 | 1.314182903 | 0.416923599 | 3.152095267 | 0.001621034 | 0.014309398 |
| ALB | 14216.28925 | 1.776337754 | 0.536374015 | 3.311752073 | 0.000927137 | 0.009635847 |
| RP1-290I10.7 | 5.349870791 | -1.814653858 | 0.587772399 | -3.087341054 | 0.002019557 | 0.016755881 |
| GAMT | 2436.465442 | 1.704823831 | 0.350151275 | 4.86882086 | 1.12E-06 | 7.04E-05 |
| CTD-2231E14.5 | 9.336218675 | 1.499318392 | 0.365055888 | 4.107092752 | 4.01E-05 | 0.001022522 |
| AC093642.3 | 643.1305197 | 1.084967511 | 0.282169409 | 3.845092621 | 0.000120507 | 0.002287813 |
| RHCG | 87.02958234 | 1.355843788 | 0.391004316 | 3.467592894 | 0.000525142 | 0.006381402 |
| BCAS1 | 2376.161604 | -1.339294786 | 0.362371451 | -3.69591694 | 0.000219095 | 0.003444998 |
| RP4-604G5.1 | 7.428174154 | 1.030384445 | 0.377553976 | 2.729105008 | 0.006350648 | 0.038104864 |
| RP11-338N10.3 | 8.037095098 | -1.315586146 | 0.31332348 | -4.198811236 | 2.68E-05 | 0.000756007 |
| RP11-736K20.5 | 355.1892483 | 1.107230217 | 0.21326296 | 5.191854295 | 2.08E-07 | 2.02E-05 |
| LIPE | 1816.874473 | 2.228231867 | 0.306071972 | 7.28009118 | 3.34E-13 | 4.07E-10 |
| LINC00552 | 6.307353547 | 1.798882868 | 0.545366481 | 3.298484467 | 0.000972083 | 0.009965962 |
| ARC | 318.6044153 | 2.411011043 | 0.401877235 | 5.999372026 | 1.98E-09 | 5.29E-07 |
| RP11-33A14.1 | 53.56815549 | 1.374741227 | 0.47122277 | 2.917391338 | 0.003529726 | 0.025172215 |
| MRGPRF | 815.0721443 | 1.032331338 | 0.223688514 | 4.615039548 | 3.93E-06 | 0.000183543 |
| TNNI3 | 41.93772764 | -2.472411143 | 0.519429285 | -4.759860898 | 1.94E-06 | 0.0001067 |
| KRT4 | 39.52346455 | -1.377193345 | 0.505157486 | -2.726265339 | 0.006405548 | 0.038322239 |
| AC005351.1 | 27.90355462 | -1.03306974 | 0.349970092 | -2.951880072 | 0.003158456 | 0.023286818 |
| CTD-3247F14.2 | 190.7005503 | 1.78898456 | 0.327153441 | 5.468334832 | 4.54E-08 | 6.09E-06 |
| IL6 | 1141.092585 | 2.284336165 | 0.420463214 | 5.432903736 | 5.54E-08 | 7.08E-06 |
| RPS12P26 | 207.9149209 | -1.12459213 | 0.23343115 | -4.817660933 | 1.45E-06 | 8.70E-05 |
| COL26A1 | 86.65769703 | 1.300411188 | 0.319592288 | 4.068969243 | 4.72E-05 | 0.001159575 |
| TSPAN7 | 1219.933908 | 1.109665586 | 0.225992187 | 4.910194462 | 9.10E-07 | 6.03E-05 |
| CPA1 | 895827.0787 | 1.847890551 | 0.687504665 | 2.687822564 | 0.00719196 | 0.041634386 |
| AC007193.6 | 13.8074825 | -1.444126014 | 0.423884893 | -3.406882475 | 0.000657094 | 0.007514189 |
| KRT6B | 515.3223582 | -1.662489042 | 0.463552394 | -3.586410218 | 0.000335261 | 0.004645885 |
| RP11-15J10.8 | 100.4768924 | -1.012641267 | 0.240315534 | -4.213798619 | 2.51E-05 | 0.000717369 |
| ANXA10 | 2740.97085 | -1.765262035 | 0.427836146 | -4.126023601 | 3.69E-05 | 0.00096015 |
| STMN2 | 840.3494535 | -1.342427131 | 0.339793824 | -3.950710806 | 7.79E-05 | 0.001649548 |
| MMP7 | 25723.25312 | -1.052224044 | 0.377436136 | -2.787820097 | 0.0053064 | 0.033519506 |
| RP5-1158E12.3 | 171.7817227 | -1.26360211 | 0.285465599 | -4.426460192 | 9.58E-06 | 0.000350414 |
| TRBV7-7 | 62.20944614 | 1.699054951 | 0.480502363 | 3.535997073 | 0.000406239 | 0.005305363 |
| TTC36 | 41.83846646 | 1.044247579 | 0.210493821 | 4.960941712 | 7.02E-07 | 5.02E-05 |
| SVEP1 | 14279.17506 | 1.16256913 | 0.222352822 | 5.228488302 | 1.71E-07 | 1.70E-05 |
| IL36B | 2.586522761 | -2.364514127 | 0.869364413 | -2.719819321 | 0.00653176 | 0.038886407 |
| PC | 1975.712544 | 1.419988729 | 0.261346232 | 5.43336216 | 5.53E-08 | 7.08E-06 |
| HTR6 | 24.99297035 | 1.107621799 | 0.302386936 | 3.662928741 | 0.000249348 | 0.003771653 |
| RP11-815D16.1 | 5.581777014 | 1.944810783 | 0.741751179 | 2.621918019 | 0.008743646 | 0.047759977 |
| WNT11 | 661.2006601 | 1.020394295 | 0.281213741 | 3.628536398 | 0.000285033 | 0.0041394 |
| SYCN | 42064.18555 | 2.114400209 | 0.659322004 | 3.206931052 | 0.001341591 | 0.012488746 |
| CTC-327F10.5 | 28.69019387 | -1.323083324 | 0.454179737 | -2.913127151 | 0.003578289 | 0.025425541 |
| CLPS | 237852.1391 | 1.955555609 | 0.642216806 | 3.04500846 | 0.002326738 | 0.018584829 |
| RP11-109G23.3 | 11.37998764 | 1.346826181 | 0.297228092 | 4.531288313 | 5.86E-06 | 0.000247144 |
| HNRNPA1P2 | 3.268722727 | 1.198124735 | 0.412904424 | 2.901699919 | 0.003711439 | 0.026067643 |
| SYT8 | 2180.767926 | -1.979650035 | 0.427086587 | -4.635242817 | 3.57E-06 | 0.000169749 |
| RNU1-60P | 30.20738982 | 1.840582634 | 0.360390516 | 5.107189431 | 3.27E-07 | 2.80E-05 |
| RP11-680F20.4 | 2.897212783 | 2.730506197 | 0.823384521 | 3.316198115 | 0.000912511 | 0.009528945 |
| RN7SL515P | 7.22130907 | 1.065981577 | 0.37119892 | 2.871725963 | 0.004082368 | 0.027843086 |
| ELF5 | 38.82622928 | -1.847113494 | 0.593090893 | -3.114385192 | 0.001843287 | 0.015649677 |
| VWC2 | 19.66422151 | 1.580850712 | 0.418375534 | 3.778544834 | 0.000157747 | 0.002737898 |
| RP11-386G21.2 | 10.64367471 | 1.072416867 | 0.362762233 | 2.9562528 | 0.003114017 | 0.023046329 |
| ASTL | 22.40812115 | 1.353644336 | 0.352417677 | 3.841022809 | 0.000122523 | 0.002313502 |
| AL117190.1 | 12.33110361 | 1.133484998 | 0.312639016 | 3.625539161 | 0.000288359 | 0.00417393 |
| APOLD1 | 8226.467524 | 1.642969171 | 0.270715616 | 6.068985584 | 1.29E-09 | 3.75E-07 |
| CCL8 | 277.7648135 | 1.014037645 | 0.353828069 | 2.865905032 | 0.00415819 | 0.028229214 |
| KLHL31 | 281.3034839 | 1.187617537 | 0.294905566 | 4.027111295 | 5.65E-05 | 0.001315027 |
| JUN | 34573.3108 | 1.283602152 | 0.233323731 | 5.501378472 | 3.77E-08 | 5.36E-06 |
| TRBV22-1 | 14.5561022 | 1.2067618 | 0.455340124 | 2.65024261 | 0.008043399 | 0.045039107 |
| HSPB8 | 1766.702049 | 1.195625859 | 0.205442928 | 5.819746987 | 5.89E-09 | 1.22E-06 |
| BTNL9 | 946.1534375 | 2.026688555 | 0.239818056 | 8.450942314 | 2.89E-17 | 1.88E-13 |
| IP6K3 | 38.95135918 | 1.40985529 | 0.404231224 | 3.48774465 | 0.000487113 | 0.006044201 |
| MT1JP | 15.01668189 | 1.757049436 | 0.477319996 | 3.681072341 | 0.000232255 | 0.003589947 |
| CES3 | 467.1687091 | 1.058474317 | 0.344006757 | 3.076899786 | 0.002091656 | 0.01718949 |
| MRO | 126.0033256 | 1.078482671 | 0.243164288 | 4.435201733 | 9.20E-06 | 0.000339678 |
| RP4-663N10.1 | 27.05784468 | 2.294734469 | 0.447189496 | 5.131458782 | 2.88E-07 | 2.52E-05 |
| RP11-304F15.4 | 15.34608472 | -1.142821479 | 0.421928878 | -2.708564263 | 0.006757502 | 0.039883276 |
| FGF16 | 5.822431951 | 2.266277368 | 0.845892229 | 2.679156151 | 0.007380797 | 0.042313245 |
| COL6A4P1 | 23.15247539 | -1.814131132 | 0.44436867 | -4.082491078 | 4.46E-05 | 0.001112319 |
| RP11-397O4.1 | 32.39767281 | 1.654772631 | 0.306200887 | 5.404205869 | 6.51E-08 | 7.88E-06 |
| AC144831.1 | 263.0411156 | 1.073333183 | 0.203592601 | 5.271965585 | 1.35E-07 | 1.40E-05 |
| GBP6 | 46.96518398 | -1.215190173 | 0.29023858 | -4.186866455 | 2.83E-05 | 0.000785544 |
| AL603965.1 | 215.477664 | -1.301522626 | 0.422093721 | -3.083492031 | 0.002045866 | 0.016934524 |
| AC003682.17 | 15.61824943 | -1.124118032 | 0.412672093 | -2.723998182 | 0.006449686 | 0.038539 |
| CREG2 | 201.4093477 | -2.099091221 | 0.39320077 | -5.338471798 | 9.37E-08 | 1.05E-05 |
| RP11-53A1.2 | 144.5827534 | 1.07191264 | 0.34827337 | 3.077790993 | 0.002085411 | 0.017149011 |
| TMEM105 | 72.6425098 | -1.337110305 | 0.462126907 | -2.893383365 | 0.003811158 | 0.026567056 |
| TRBV10-1 | 39.30330621 | 1.781001137 | 0.553499073 | 3.217712953 | 0.001292171 | 0.012194626 |
| CTD-3080P12.3 | 53.00027078 | 1.486349275 | 0.457763381 | 3.2469816 | 0.001166359 | 0.01137908 |
| IL37 | 6.791718125 | -1.61825303 | 0.520819641 | -3.107127505 | 0.001889149 | 0.015917754 |
| RP11-353P15.1 | 3.143158857 | 2.872515018 | 0.898019142 | 3.198723595 | 0.001380374 | 0.012752358 |
| CYP1A1 | 45.85421828 | 3.114608707 | 0.702495888 | 4.433632651 | 9.27E-06 | 0.000341514 |
| GALNT13 | 442.7732996 | 1.015667341 | 0.288043483 | 3.526090329 | 0.000421743 | 0.00544385 |
| RIMS4 | 127.1123098 | 1.079073032 | 0.296534581 | 3.638944999 | 0.000273757 | 0.004019264 |
| AP006621.8 | 98.26216056 | 1.628107878 | 0.258285657 | 6.303516411 | 2.91E-10 | 1.07E-07 |
| RN7SKP268 | 33.43222545 | 1.095469538 | 0.253070248 | 4.328717203 | 1.50E-05 | 0.000484949 |
| CEACAMP5 | 7.276539866 | 2.109671148 | 0.624506326 | 3.378142161 | 0.000729773 | 0.008084521 |
| MIR541 | 3.191953272 | 1.104602886 | 0.343861551 | 3.212347768 | 0.001316549 | 0.012349971 |
| DHRS9 | 1567.842436 | -1.94356371 | 0.360836222 | -5.386276638 | 7.19E-08 | 8.50E-06 |
| TRBV6-5 | 131.776498 | 1.310725941 | 0.358357411 | 3.657594066 | 0.000254594 | 0.003821756 |
| PLIN4 | 12045.75284 | 3.793314989 | 0.414458644 | 9.152457178 | 5.57E-20 | 2.17E-15 |
| PTH1R | 344.1605212 | 1.448490003 | 0.231013474 | 6.270153767 | 3.61E-10 | 1.28E-07 |
| HSPB7 | 692.4878885 | 1.194362061 | 0.266056569 | 4.489128253 | 7.15E-06 | 0.000282396 |
| RP1L1 | 131.5268079 | -1.491437179 | 0.347560174 | -4.291162482 | 1.78E-05 | 0.000552269 |
| LILRB5 | 1112.249546 | 1.543284588 | 0.263438552 | 5.85823364 | 4.68E-09 | 1.01E-06 |
| LRRC31 | 219.7213079 | -1.234142868 | 0.466464731 | -2.645736721 | 0.008151323 | 0.045460647 |
| NEURL | 1432.208317 | 1.019773097 | 0.322851269 | 3.158646709 | 0.001585035 | 0.014110675 |
| TRIM29 | 3676.686133 | -1.217090477 | 0.408553312 | -2.979024872 | 0.002891673 | 0.021823261 |
| CNTFR | 265.2538679 | 2.956449891 | 0.429402416 | 6.885033203 | 5.78E-12 | 4.25E-09 |
| LGSN | 34.47910261 | 1.140936609 | 0.397182185 | 2.872577505 | 0.004071382 | 0.027789872 |
| OR5P2 | 8.967923977 | -2.072362702 | 0.62274605 | -3.327781366 | 0.000875405 | 0.00923627 |
| RBPJL | 8481.670881 | 2.210062401 | 0.597640146 | 3.697981829 | 0.00021732 | 0.00342438 |
| ALDH7A1P2 | 15.63298929 | 1.006362412 | 0.31442928 | 3.20060019 | 0.001371417 | 0.012700583 |
| WNT10A | 459.4099399 | -1.190557071 | 0.286165817 | -4.160374864 | 3.18E-05 | 0.000862793 |
| PRRG3 | 247.4248453 | 1.316768694 | 0.358624523 | 3.671719607 | 0.000240924 | 0.003685692 |
| MAGEB17 | 26.83791476 | 1.417459465 | 0.484814245 | 2.92371662 | 0.003458795 | 0.02478877 |
| RP11-64B16.4 | 42.5476419 | 1.741181424 | 0.316643706 | 5.498866369 | 3.82E-08 | 5.40E-06 |
| MRAP | 38.02429629 | 2.454734736 | 0.348213731 | 7.049505846 | 1.80E-12 | 1.49E-09 |
| SOX21-AS1 | 174.4061355 | -1.833756974 | 0.439040945 | -4.176733387 | 2.96E-05 | 0.000813819 |
| CTD-2196E14.5 | 13.01848045 | -1.288427575 | 0.33947099 | -3.795398173 | 0.000147407 | 0.002617464 |
| MDS2 | 16.42025909 | 1.100216629 | 0.398029058 | 2.764161578 | 0.005706927 | 0.035334424 |
| LINC00460 | 20.75945335 | -2.154514574 | 0.54704773 | -3.938439838 | 8.20E-05 | 0.001715614 |
| RN7SL472P | 15.68109965 | 1.725238277 | 0.596708887 | 2.891256213 | 0.003837052 | 0.026695063 |
| RNU2-70P | 16.70584123 | 1.097145866 | 0.320318151 | 3.425175449 | 0.000614403 | 0.007166988 |
| RP3-333A15.1 | 27.27081501 | 1.02377232 | 0.328515372 | 3.116360472 | 0.001830983 | 0.015578111 |
| OXER1 | 300.6073555 | 1.027677036 | 0.229507273 | 4.477753679 | 7.54E-06 | 0.000294444 |
| CTD-2263F21.1 | 17.97575194 | 1.856417496 | 0.447856194 | 4.145119618 | 3.40E-05 | 0.000905271 |
| RP11-44K6.2 | 9.96075869 | -1.214217066 | 0.391860757 | -3.098593175 | 0.001944418 | 0.016281423 |
| PLEK2 | 1226.094 | -1.165857614 | 0.295259258 | -3.948589529 | 7.86E-05 | 0.001661525 |
| QPRT | 1902.162292 | 1.124231292 | 0.231831555 | 4.849345434 | 1.24E-06 | 7.63E-05 |
| CCDC58P3 | 40.44190943 | 1.217154893 | 0.372599266 | 3.266659397 | 0.001088245 | 0.010822703 |
| LINC00968 | 90.34817044 | 1.713848388 | 0.337379393 | 5.079884608 | 3.78E-07 | 3.12E-05 |
| NKAIN4 | 52.04509808 | -1.487034886 | 0.305611237 | -4.865772929 | 1.14E-06 | 7.12E-05 |
| FOSB | 9727.648123 | 2.323771007 | 0.344696295 | 6.741502713 | 1.57E-11 | 1.05E-08 |
| PTGES | 207.3112216 | -1.217774336 | 0.246146833 | -4.947349185 | 7.52E-07 | 5.29E-05 |
| MIR487B | 2.470560178 | 1.375814275 | 0.448533242 | 3.067363007 | 0.002159564 | 0.017636064 |
| OR7E22P | 13.50527161 | -1.766729451 | 0.483148195 | -3.656702996 | 0.00025548 | 0.003833185 |
| TPST2 | 8662.275304 | 1.860446578 | 0.360596881 | 5.159352941 | 2.48E-07 | 2.26E-05 |
| RNU6-619P | 11.28237316 | -1.306083733 | 0.324438711 | -4.02567168 | 5.68E-05 | 0.001321061 |
| FLRT3 | 2109.071918 | -1.399649699 | 0.301247164 | -4.646183823 | 3.38E-06 | 0.000162583 |
| ITFG3 | 45.38144986 | 1.069195997 | 0.216309964 | 4.94288832 | 7.70E-07 | 5.36E-05 |
| RP11-115D19.1 | 45.22039752 | -1.855210597 | 0.459437836 | -4.038001339 | 5.39E-05 | 0.001274246 |
| MIR22HG | 1095.228554 | 1.001938508 | 0.220748773 | 4.538818026 | 5.66E-06 | 0.000240563 |
| DBH-AS1 | 337.3567525 | 1.513164947 | 0.286292019 | 5.285389904 | 1.25E-07 | 1.31E-05 |
| OR52N1 | 44.96520151 | 2.25253321 | 0.439606348 | 5.123977896 | 2.99E-07 | 2.60E-05 |
| TINAG | 89.63235984 | -1.954774747 | 0.65863269 | -2.967928522 | 0.00299814 | 0.022414204 |
| GATM | 41781.9582 | 1.137876064 | 0.417264327 | 2.726990996 | 0.006391478 | 0.038285053 |
| RP11-302L19.3 | 37.73162698 | 1.106840262 | 0.236072221 | 4.688566303 | 2.75E-06 | 0.000139332 |
| AC096534.1 | 3.988173698 | 1.576981301 | 0.424554112 | 3.714441238 | 0.000203653 | 0.003271027 |
| TRBV7-4 | 74.52393192 | 1.760330746 | 0.534545623 | 3.29313471 | 0.00099077 | 0.010103314 |
| AFAP1-AS1 | 2798.021105 | -1.230016522 | 0.304109503 | -4.044650073 | 5.24E-05 | 0.00124978 |
| RN7SL395P | 5.129201553 | 1.140214409 | 0.404770902 | 2.816937687 | 0.004848393 | 0.031592058 |
| AC129778.7 | 25.97220426 | -1.001928555 | 0.337745987 | -2.966515052 | 0.003011956 | 0.022483008 |
| ATOH7 | 16.29593528 | 1.400720954 | 0.47152406 | 2.97062456 | 0.002971949 | 0.022286757 |
| IGFL4 | 71.45243655 | -1.297968375 | 0.326247779 | -3.978474216 | 6.94E-05 | 0.001509292 |
| IL10 | 178.7523048 | 1.285666735 | 0.321722421 | 3.996198744 | 6.44E-05 | 0.001431544 |
| REEP6 | 1056.07394 | 1.237082989 | 0.336067592 | 3.681054104 | 0.000232272 | 0.003589947 |
| CMTM5 | 18.66258693 | 1.198824281 | 0.404041353 | 2.967083128 | 0.003006397 | 0.022454468 |
| TCEB1P26 | 2.615061675 | -3.134098359 | 1.184837604 | -2.645171245 | 0.008164958 | 0.045511948 |
| RP11-331F4.4 | 25088.62895 | 2.1980674 | 0.570193875 | 3.854947407 | 0.000115755 | 0.002218107 |
| RP11-419C23.1 | 9.796646959 | -1.426260597 | 0.52761775 | -2.70320814 | 0.006867373 | 0.040293892 |
| ERO1L | 14966.18395 | -1.25265359 | 0.196766949 | -6.366178858 | 1.94E-10 | 7.87E-08 |
| PADI1 | 1553.047522 | -2.407877237 | 0.500351199 | -4.812374271 | 1.49E-06 | 8.90E-05 |
| CTD-2571E19.1 | 28.15184136 | -2.010483237 | 0.546508358 | -3.678778574 | 0.000234354 | 0.003609249 |
| SLC34A2 | 2151.952769 | -1.124391627 | 0.413872369 | -2.716759346 | 0.006592452 | 0.039126747 |
| UGT1A13P | 28.26045288 | -1.799198378 | 0.495419648 | -3.631665367 | 0.000281598 | 0.004105016 |
| LEFTY2 | 153.988653 | 1.288008278 | 0.465611788 | 2.766270769 | 0.005670143 | 0.035187546 |
| LYVE1 | 2744.644714 | 2.260785858 | 0.347954944 | 6.497352297 | 8.17E-11 | 4.04E-08 |
| POU2F3 | 272.4075052 | -1.148611216 | 0.345592194 | -3.323602893 | 0.000888626 | 0.009343935 |
| AP000925.2 | 289.8476596 | 1.235204642 | 0.169884488 | 7.270850074 | 3.57E-13 | 4.13E-10 |
| OR51J1 | 12.36045003 | 1.270086666 | 0.343290927 | 3.69973852 | 0.000215822 | 0.003411418 |
| AL591069.1 | 122.7703165 | -1.016734392 | 0.347469943 | -2.926107458 | 0.003432324 | 0.024671605 |
| SNORD113-9 | 10.31996664 | 1.047049362 | 0.273751482 | 3.824817146 | 0.000130869 | 0.002418599 |
| PRDX4 | 5879.664313 | 1.001328721 | 0.244941097 | 4.088038854 | 4.35E-05 | 0.001092802 |
| KLHL4 | 430.3039769 | 1.162453009 | 0.291564239 | 3.986953318 | 6.69E-05 | 0.001468669 |
| RP11-343B22.2 | 6.686272426 | -2.118950315 | 0.798351289 | -2.654157819 | 0.007950663 | 0.044693111 |
| ROBO4 | 1601.648425 | 1.063296852 | 0.150754284 | 7.05317835 | 1.75E-12 | 1.49E-09 |
| RP13-463N16.6 | 19.69855834 | -1.72296362 | 0.499087335 | -3.4522287 | 0.000555976 | 0.006644283 |
| ALPPL2 | 56.4656313 | -1.814415161 | 0.617306023 | -2.93924746 | 0.003290103 | 0.023962935 |
| CHST6 | 272.6766985 | -1.373141615 | 0.346702103 | -3.960580575 | 7.48E-05 | 0.001598448 |
| FRMD1 | 38.67482948 | 2.311307944 | 0.492723584 | 4.690881493 | 2.72E-06 | 0.000138123 |
| CHRDL1 | 5337.540435 | 2.136244121 | 0.375460049 | 5.689670914 | 1.27E-08 | 2.26E-06 |
| TNFRSF11B | 1010.013162 | -1.310243399 | 0.256813288 | -5.101929916 | 3.36E-07 | 2.86E-05 |
| GSTA1 | 2029.64221 | 1.485229982 | 0.462176989 | 3.213552425 | 0.001311039 | 0.012319024 |
| RP11-779O18.2 | 15.21661905 | 2.309620769 | 0.413262164 | 5.58875448 | 2.29E-08 | 3.67E-06 |
| AC114808.2 | 10.2014074 | 1.062407963 | 0.352359355 | 3.015126314 | 0.002568723 | 0.019961265 |
| SNORA26 | 9.05572695 | 2.518014835 | 0.540900929 | 4.655223723 | 3.24E-06 | 0.00015716 |
| FGFBP1 | 175.3440279 | -1.632588594 | 0.485819467 | -3.360484101 | 0.00077806 | 0.008475581 |
| HBB | 9854.850241 | 3.558900358 | 0.398371439 | 8.933623283 | 4.12E-19 | 8.04E-15 |
| LMOD1 | 3205.797758 | 1.069090492 | 0.24546417 | 4.355383069 | 1.33E-05 | 0.000443483 |
| RP11-334A14.8 | 18.7462092 | 1.268084414 | 0.394525843 | 3.214198608 | 0.001308092 | 0.012294924 |
| TFF2 | 2779.020246 | -2.556835517 | 0.513267079 | -4.981491358 | 6.31E-07 | 4.62E-05 |
| CLIC3 | 368.9740183 | -1.515785133 | 0.257408425 | -5.888638396 | 3.89E-09 | 8.93E-07 |
| ZYG11A | 42.98399939 | 1.214873661 | 0.286309954 | 4.243211394 | 2.20E-05 | 0.000646272 |
| AC092377.1 | 7.101940874 | 1.478507414 | 0.330995217 | 4.466854323 | 7.94E-06 | 0.000305563 |
| SERPINB2 | 737.8367855 | -1.182600618 | 0.43760798 | -2.702420138 | 0.006883672 | 0.040353095 |
| AL136985.1 | 4.510236515 | -1.218807241 | 0.377458058 | -3.228987205 | 0.001242295 | 0.011864628 |
| GPR110 | 1643.657428 | -1.575525069 | 0.438035367 | -3.596798771 | 0.000322158 | 0.004507115 |
| HMCN2 | 1024.25531 | 1.385172797 | 0.275945662 | 5.019730295 | 5.17E-07 | 4.00E-05 |
| PCDH15 | 70.51870078 | 1.56882105 | 0.570482266 | 2.749990918 | 0.005959692 | 0.036380428 |
| NRG2 | 252.4710374 | 1.105329085 | 0.274250643 | 4.030360968 | 5.57E-05 | 0.00130353 |
| SLAMF9 | 24.78400017 | -1.177777142 | 0.43589322 | -2.701985461 | 0.006892678 | 0.040385596 |
| PCP4L1 | 56.04814592 | 1.300058942 | 0.263919844 | 4.925961323 | 8.39E-07 | 5.69E-05 |
| ZSCAN4 | 17.3986037 | -1.30928323 | 0.416853318 | -3.140872754 | 0.001684452 | 0.014710493 |
| AF064858.6 | 10.48072789 | -1.445362929 | 0.508287927 | -2.84359091 | 0.004460828 | 0.029709649 |
| RP11-310I9.1 | 3.200704602 | -1.78882892 | 0.671970234 | -2.662065714 | 0.007766271 | 0.043960772 |
| KLK1 | 25845.08384 | 1.590598693 | 0.571263424 | 2.784352415 | 0.005363472 | 0.033719443 |
| B3GNT3 | 2536.552203 | -1.486900747 | 0.323011375 | -4.603245773 | 4.16E-06 | 0.000191729 |
| MUC1 | 9636.503202 | -1.224960472 | 0.32448171 | -3.775129485 | 0.000159925 | 0.002760628 |
| MIR135B | 4.120435459 | -1.293050056 | 0.333849139 | -3.873156778 | 0.000107435 | 0.002105234 |
| CTD-2530H12.2 | 30.80864055 | 1.834927704 | 0.375934245 | 4.880980466 | 1.06E-06 | 6.77E-05 |
| CTB-43E15.4 | 17.29917889 | 2.283477736 | 0.504086133 | 4.52993563 | 5.90E-06 | 0.000248195 |
| MIR323B | 3.706609158 | 1.250751563 | 0.328750172 | 3.804565506 | 0.000142053 | 0.0025586 |
| MIR4705 | 2.550475971 | 1.184035022 | 0.411709957 | 2.87589601 | 0.004028824 | 0.02755727 |
| GPR87 | 283.5389281 | -1.221778747 | 0.43921309 | -2.781744843 | 0.005406753 | 0.033891068 |
| RP11-57H12.2 | 25.15397124 | 1.172215453 | 0.279727118 | 4.190567798 | 2.78E-05 | 0.000778941 |
| AC005329.7 | 90.88203682 | 1.344019251 | 0.317213184 | 4.236958988 | 2.27E-05 | 0.000659328 |
| RP11-115P21.1 | 46.56571718 | 1.081551653 | 0.301690154 | 3.584974979 | 0.000337111 | 0.004658536 |
| MLPH | 3961.402827 | -1.00740172 | 0.289591273 | -3.478701929 | 0.000503849 | 0.006190164 |
| CD164L2 | 20.12189089 | -1.246920266 | 0.443251352 | -2.813122304 | 0.004906299 | 0.031830703 |
| MTRNR2L6 | 54.47406548 | 1.203708877 | 0.375391225 | 3.206545055 | 0.001343393 | 0.012493584 |
| RP11-254F19.2 | 72.55109968 | 1.254405579 | 0.267894371 | 4.682463362 | 2.83E-06 | 0.000142253 |
| MUSTN1 | 174.2283131 | 2.175664424 | 0.408935859 | 5.320307274 | 1.04E-07 | 1.14E-05 |
| TWIST2 | 254.7676072 | 1.495688418 | 0.280173589 | 5.338434732 | 9.38E-08 | 1.05E-05 |
| AC092159.2 | 21.55951013 | 1.088764256 | 0.253793555 | 4.289960228 | 1.79E-05 | 0.000554825 |
| HBG1 | 6.775469855 | 2.85449603 | 0.976600049 | 2.92289155 | 0.003467973 | 0.024845416 |
| RBM24 | 118.2467648 | 1.40321501 | 0.310342353 | 4.521506634 | 6.14E-06 | 0.000254716 |
| FBXO40 | 26.27322653 | 1.600276847 | 0.470287858 | 3.402760288 | 0.000667088 | 0.007597281 |
| ZNF365 | 565.5461291 | -1.179658481 | 0.26564491 | -4.440734374 | 8.97E-06 | 0.000333906 |
| SDCBP2 | 1586.356886 | -1.017840578 | 0.279087395 | -3.647031703 | 0.000265287 | 0.003928931 |
| F8 | 3408.282805 | 1.298467931 | 0.250962394 | 5.173954201 | 2.29E-07 | 2.14E-05 |
| AC009262.2 | 4.650937383 | -2.352160699 | 0.879608141 | -2.674100646 | 0.007492996 | 0.042792821 |
| RP11-118D22.3 | 32.32279263 | 1.238849679 | 0.411331446 | 3.01180396 | 0.002597003 | 0.020127376 |
| RP11-368L12.1 | 98.89867789 | -1.129250638 | 0.37212534 | -3.034597529 | 0.002408569 | 0.019085566 |
| CBS | 3548.806455 | 1.293096231 | 0.344640327 | 3.752016605 | 0.000175418 | 0.002953549 |
| LPL | 6590.780905 | 2.712028546 | 0.373052219 | 7.269836245 | 3.60E-13 | 4.13E-10 |
| PRODH2 | 41.89356909 | 1.588276665 | 0.507349996 | 3.130534498 | 0.001744885 | 0.015093565 |
| ZNF295-AS1 | 53.0038429 | 1.01954354 | 0.201353764 | 5.06344417 | 4.12E-07 | 3.35E-05 |
| PEX5L-AS2 | 11.31669111 | 1.952215974 | 0.591637932 | 3.299680207 | 0.000967951 | 0.009938187 |
| RP11-384L8.1 | 272.8072443 | 1.175258343 | 0.314579318 | 3.735968242 | 0.000186994 | 0.003076726 |
| RP11-561I11.3 | 8.688203117 | 1.869585792 | 0.529230055 | 3.532652338 | 0.000411413 | 0.005351157 |
| ANXA8L1 | 425.5425113 | -1.237495391 | 0.410716321 | -3.013017323 | 0.002586642 | 0.020076848 |
| RP11-175K6.1 | 123.1246978 | 1.541243107 | 0.291345789 | 5.290081972 | 1.22E-07 | 1.30E-05 |
| ATOH8 | 890.5440283 | 1.5554118 | 0.266255595 | 5.841799484 | 5.16E-09 | 1.08E-06 |
| AC079753.4 | 17.52737416 | -1.690454489 | 0.422517213 | -4.000912711 | 6.31E-05 | 0.0014127 |
| SPSB4 | 458.391782 | 1.511717991 | 0.357892821 | 4.223940534 | 2.40E-05 | 0.000690371 |
| RP4-781K5.7 | 7.381072553 | -1.645204442 | 0.499178198 | -3.295825919 | 0.000981328 | 0.010022757 |
| LGI1 | 33.53123177 | 1.51433895 | 0.496737114 | 3.048572187 | 0.002299317 | 0.018457294 |
| CTB-60B18.18 | 24.67093739 | -1.87301873 | 0.417320362 | -4.48820355 | 7.18E-06 | 0.000283202 |
| MIR365B | 4.05823976 | 1.000217974 | 0.384500432 | 2.601344211 | 0.009285923 | 0.049862926 |
| TRBV12-1 | 38.1340733 | 1.886842224 | 0.592640269 | 3.183790105 | 0.001453604 | 0.013244476 |
| MIR1193 | 5.206681568 | 1.191667676 | 0.36664448 | 3.250199415 | 0.001153241 | 0.011282147 |
| AP000892.4 | 23.60100114 | 1.305445783 | 0.255962168 | 5.100151296 | 3.39E-07 | 2.88E-05 |
| SPINK5 | 256.3759671 | -1.040933364 | 0.297171049 | -3.502808796 | 0.00046038 | 0.005802363 |
| MEG9 | 116.8778523 | 1.00174175 | 0.237093933 | 4.225083871 | 2.39E-05 | 0.000688398 |
| AC011298.2 | 506.4405163 | 1.905630621 | 0.602041569 | 3.165280803 | 0.001549333 | 0.013901578 |
| RP11-320G24.1 | 26.33599362 | -2.059200305 | 0.514260553 | -4.004196497 | 6.22E-05 | 0.001400234 |
| HBA1 | 5628.042449 | 3.272834575 | 0.383059593 | 8.543930597 | 1.30E-17 | 1.10E-13 |
| SHE | 1631.995576 | 1.010418238 | 0.175703934 | 5.750686484 | 8.89E-09 | 1.72E-06 |
| ICAM5 | 112.7850331 | -1.059876417 | 0.263666093 | -4.019767597 | 5.83E-05 | 0.001344983 |
| FAM107A | 948.3838843 | 1.519716856 | 0.262730919 | 5.784309137 | 7.28E-09 | 1.45E-06 |
| AC011298.1 | 6.175575539 | 1.630245711 | 0.617103521 | 2.641770231 | 0.008247399 | 0.045841074 |
| COL6A6 | 769.1990811 | 1.161901828 | 0.230895797 | 5.032148026 | 4.85E-07 | 3.80E-05 |
| HNRNPA1P33 | 161.8926496 | -1.293376169 | 0.327997194 | -3.943253759 | 8.04E-05 | 0.001690699 |
| MIR654 | 9.532829096 | 1.205085719 | 0.339905147 | 3.545358847 | 0.000392079 | 0.005187029 |
| AC004004.2 | 26.54135009 | 1.167055217 | 0.446190245 | 2.615600027 | 0.008907083 | 0.048368151 |
| NLRP6 | 183.1701743 | 1.066934253 | 0.275554153 | 3.871958535 | 0.000107964 | 0.002112428 |
| RP11-38L15.2 | 9.220724784 | 1.088508607 | 0.355924609 | 3.058256099 | 0.002226292 | 0.018041202 |
| AQP7P4 | 46.93570814 | 1.417357595 | 0.351008052 | 4.03796319 | 5.39E-05 | 0.001274246 |
| TRBV5-4 | 122.8258452 | 1.439897927 | 0.403403155 | 3.569376963 | 0.000357831 | 0.004863586 |
| ANKRD30A | 16.48966217 | 2.183651 | 0.555283455 | 3.93249786 | 8.41E-05 | 0.001745592 |
| SLC26A3 | 14.67564091 | 1.090462813 | 0.384827543 | 2.833640248 | 0.004602111 | 0.030370505 |
| FOXD4L5 | 40.57359119 | -1.017152429 | 0.286799738 | -3.546559823 | 0.000390296 | 0.005173215 |
| Y_RNA | 2.825042816 | 1.319830892 | 0.450211934 | 2.931576867 | 0.003372459 | 0.024367393 |
| RP11-554A11.6 | 23.62258797 | 1.405582423 | 0.381627624 | 3.683125475 | 0.000230392 | 0.00356796 |
| CLSTN2 | 3576.318598 | 1.042461393 | 0.221486542 | 4.706657947 | 2.52E-06 | 0.00013023 |
| CPA6 | 59.82860372 | -1.772330233 | 0.476100544 | -3.72259653 | 0.000197184 | 0.003187898 |
| SULT1A2 | 118.3008256 | 1.255616542 | 0.244311142 | 5.139415806 | 2.76E-07 | 2.45E-05 |
| RP3-414A15.10 | 25.5226157 | 1.042022329 | 0.362731978 | 2.872705998 | 0.004069727 | 0.027783708 |
| CYB5A | 3707.856506 | 1.115716468 | 0.224278598 | 4.974689868 | 6.54E-07 | 4.75E-05 |
| RP1-169K13.3 | 35.71591432 | 1.04986052 | 0.27784958 | 3.778521168 | 0.000157762 | 0.002737898 |
| SLC44A4 | 419.853891 | -1.463939625 | 0.477298015 | -3.067139565 | 0.002161179 | 0.017638172 |
| AOC4P | 205.5855442 | 1.787039425 | 0.255224102 | 7.001844318 | 2.53E-12 | 2.05E-09 |
| IGFN1 | 839.0667342 | 1.161465427 | 0.343546137 | 3.380813528 | 0.000722716 | 0.008024573 |
| AC139712.1 | 3.548225723 | 2.40492367 | 0.792744356 | 3.033668613 | 0.002415997 | 0.019109896 |
| KRT18P15 | 331.186039 | 1.013269683 | 0.223796741 | 4.527633767 | 5.96E-06 | 0.000250331 |
| C8orf12 | 64.10917074 | 1.248672662 | 0.409979435 | 3.045695849 | 0.002321425 | 0.018557602 |
| EGF | 3183.532472 | 1.712876364 | 0.48671923 | 3.519228866 | 0.000432803 | 0.005544402 |
| ADAMTS15 | 1748.36772 | 1.84792288 | 0.301375371 | 6.13163204 | 8.70E-10 | 2.71E-07 |
| AC114812.8 | 10.96336759 | -1.459841267 | 0.552804391 | -2.640791736 | 0.008271255 | 0.045934427 |
| RAMP2-AS1 | 152.6755281 | 1.210554578 | 0.218135352 | 5.54955705 | 2.86E-08 | 4.38E-06 |
| AP000688.29 | 63.81280481 | -1.40026287 | 0.301070821 | -4.650941819 | 3.30E-06 | 0.000159663 |
| CCL7 | 25.18689168 | -1.675514162 | 0.511867183 | -3.273337727 | 0.001062854 | 0.010627979 |
| ODF3L1 | 55.15489629 | 1.112515762 | 0.255271656 | 4.358164078 | 1.31E-05 | 0.000439391 |
| ARL14 | 1153.872646 | -1.599657668 | 0.359087176 | -4.454789184 | 8.40E-06 | 0.000318234 |
| LINC00994 | 15.1750127 | 1.418044801 | 0.378664943 | 3.744853675 | 0.000180499 | 0.003017835 |
| PAWRP1 | 14.95650193 | 1.111988919 | 0.255454407 | 4.352983886 | 1.34E-05 | 0.000446743 |
| NR4A1 | 12898.952 | 1.891738403 | 0.278954733 | 6.781524663 | 1.19E-11 | 8.14E-09 |
| OGN | 3800.097958 | 1.311992312 | 0.308225079 | 4.256604673 | 2.08E-05 | 0.000622106 |
| VSIG1 | 2224.689351 | -2.063901211 | 0.452474056 | -4.561369177 | 5.08E-06 | 0.000223676 |
| MYBPC1 | 844.1268963 | -2.043983497 | 0.639052751 | -3.198458178 | 0.001381646 | 0.012761079 |
| AP001412.1 | 26.50291521 | 1.076155723 | 0.292722882 | 3.676363511 | 0.000236582 | 0.003633332 |
| ALDH3A1 | 232.8271726 | -1.782668952 | 0.329344233 | -5.412783269 | 6.21E-08 | 7.66E-06 |
| NTRK3 | 401.4818929 | 1.840057016 | 0.289799862 | 6.349406106 | 2.16E-10 | 8.43E-08 |
| PCDH7 | 7468.313813 | -1.232881809 | 0.219882369 | -5.607006225 | 2.06E-08 | 3.40E-06 |
| SLC38A3 | 1453.282282 | 2.069512212 | 0.51530293 | 4.016107982 | 5.92E-05 | 0.001357984 |
| SDIM1 | 97.27403837 | 1.093836395 | 0.350141582 | 3.123983131 | 0.001784207 | 0.015304696 |
| AKR7A3 | 2388.788292 | 1.131748199 | 0.391894317 | 2.887891327 | 0.003878338 | 0.026876809 |
| CECR2 | 625.5260958 | 1.5075099 | 0.264158389 | 5.706840915 | 1.15E-08 | 2.09E-06 |
| CEBPA-AS1 | 44.62780807 | 1.192403976 | 0.408579544 | 2.9184133 | 0.003518177 | 0.025122013 |
| RNU6-1189P | 33.52144458 | 1.427978343 | 0.447587666 | 3.190388058 | 0.001420819 | 0.013021111 |
| PLXNA4 | 1922.329656 | 1.464557856 | 0.267884542 | 5.467123419 | 4.57E-08 | 6.11E-06 |
| CIB4 | 13.34286653 | 1.23195877 | 0.422701513 | 2.914488672 | 0.003562717 | 0.025333782 |
| KCNA5 | 156.9230471 | 1.174049674 | 0.271031834 | 4.33177777 | 1.48E-05 | 0.000481047 |
| CALCB | 91.41713141 | 1.083437103 | 0.29176423 | 3.713399353 | 0.000204494 | 0.00327888 |
| GAL3ST3 | 18.09018301 | 1.219801042 | 0.43407831 | 2.810094435 | 0.004952697 | 0.031985823 |
| TMEM40 | 32.26684707 | -1.866646557 | 0.491149821 | -3.800564464 | 0.000144367 | 0.002582208 |
| C12orf36 | 838.6776567 | -1.978256212 | 0.457923828 | -4.32005519 | 1.56E-05 | 0.000499171 |
| ASPM | 2942.695289 | -1.039519275 | 0.240504401 | -4.322246376 | 1.54E-05 | 0.000496517 |
| RP11-293M10.4 | 3.025193156 | 1.503628635 | 0.545087653 | 2.758507973 | 0.005806589 | 0.035776257 |
| RP11-473L15.3 | 15.02120099 | 1.449076681 | 0.363800639 | 3.983161449 | 6.80E-05 | 0.001487289 |
| AC093390.1 | 23.85739545 | 1.345567627 | 0.41634794 | 3.231834482 | 0.001229983 | 0.011772994 |
| ATP1A2 | 1109.056101 | 2.506505063 | 0.384487674 | 6.519077812 | 7.07E-11 | 3.63E-08 |
| TRDN | 20.32737938 | 2.453718511 | 0.555784226 | 4.414876128 | 1.01E-05 | 0.00036492 |
| GJA3 | 43.99021071 | -1.084695339 | 0.286223751 | -3.789676208 | 0.000150844 | 0.0026556 |
| PF4V1 | 8.767502882 | 1.288766306 | 0.493404578 | 2.611986925 | 0.00900177 | 0.048787215 |
| ZIC2 | 72.52073459 | -1.508069652 | 0.535146427 | -2.818050492 | 0.004831621 | 0.031506533 |
| TBCAP2 | 13.40571751 | 1.2566014 | 0.32566218 | 3.858604024 | 0.000114037 | 0.002197554 |
| VENTXP7 | 4.917400535 | 1.789087181 | 0.603449875 | 2.964765188 | 0.00302914 | 0.022576707 |
| WISP2 | 625.6177361 | 1.434805288 | 0.348379986 | 4.118506651 | 3.81E-05 | 0.000987045 |
| NMU | 140.3039246 | -1.421202295 | 0.472337186 | -3.008872345 | 0.002622193 | 0.020272086 |
| IL33 | 2447.87116 | 1.167755587 | 0.268891562 | 4.342849503 | 1.41E-05 | 0.000462828 |
| BX842568.1 | 13.75363988 | 1.623601617 | 0.469823242 | 3.455771171 | 0.000548721 | 0.006580392 |
| PM20D1 | 921.5809734 | 1.581549797 | 0.427299997 | 3.701263302 | 0.000214529 | 0.003402012 |
| ITGB4 | 18554.24768 | -1.07885671 | 0.239075377 | -4.512621594 | 6.40E-06 | 0.00026277 |
| MIR4284 | 6.420389621 | -1.096315209 | 0.393031277 | -2.789384132 | 0.005280839 | 0.03344659 |
| MIR23A | 56.46888742 | 1.138733117 | 0.291232708 | 3.910045421 | 9.23E-05 | 0.001867858 |
| RP11-252I14.1 | 16.62690352 | 1.023629615 | 0.239302098 | 4.277562224 | 1.89E-05 | 0.000581543 |
| AQP7P2 | 31.94795039 | 1.157418817 | 0.334941438 | 3.455585626 | 0.000549099 | 0.006580392 |
| RP11-14D22.2 | 18.48060246 | 1.127976791 | 0.342163206 | 3.29660458 | 0.000978612 | 0.010002874 |
| VWFP1 | 40.32653869 | 1.510998934 | 0.466311482 | 3.240321103 | 0.001193952 | 0.011544015 |
| RP11-191A15.4 | 7.21066208 | 1.227016574 | 0.396643404 | 3.093500516 | 0.001978102 | 0.016477091 |
| CYP3A4 | 483.696389 | 3.349349303 | 0.449365446 | 7.453508795 | 9.09E-14 | 1.36E-10 |
| C9orf53 | 4.63702561 | -1.785972464 | 0.686380523 | -2.602015068 | 0.009267778 | 0.049802889 |
| PRAME | 85.51973324 | -1.919725356 | 0.597209913 | -3.214490105 | 0.001306764 | 0.012290539 |
| AC007556.3 | 6.1685855 | -1.967056384 | 0.649109364 | -3.030392864 | 0.002442358 | 0.019263705 |
| RP11-311C24.1 | 5.593825028 | 1.575792597 | 0.464482245 | 3.392578753 | 0.00069238 | 0.00778753 |
| MIR193A | 5.690729021 | 1.402131255 | 0.297415791 | 4.714380659 | 2.42E-06 | 0.000127244 |
| RN7SKP161 | 10.04913131 | 1.208857389 | 0.268121848 | 4.508612021 | 6.53E-06 | 0.000265928 |
| MUC4 | 3871.232792 | -1.259607041 | 0.395988674 | -3.18091684 | 0.001468098 | 0.013350855 |
| RP11-423H2.3 | 125.3903643 | 1.123207974 | 0.318389329 | 3.527781461 | 0.000419058 | 0.005425354 |
| RN7SL69P | 4.48625334 | 1.155596292 | 0.349152235 | 3.30972045 | 0.000933892 | 0.009680503 |
| ATP5G1P6 | 16.5157596 | -1.802235275 | 0.531497738 | -3.390861612 | 0.000696733 | 0.007827454 |
| RP11-354K4.2 | 105.4569318 | 1.345532294 | 0.431320472 | 3.119565108 | 0.001811182 | 0.015468036 |
| RP11-380D23.1 | 58.58233928 | 1.725368833 | 0.359070401 | 4.805099025 | 1.55E-06 | 9.02E-05 |
| GPR111 | 14.20281284 | -2.316291011 | 0.526116418 | -4.402620658 | 1.07E-05 | 0.000378282 |
| RP11-251M1.1 | 15.92098712 | 1.009583178 | 0.354965316 | 2.844174155 | 0.00445267 | 0.02967559 |
| CD209 | 1463.932407 | 1.215023854 | 0.286135942 | 4.246316785 | 2.17E-05 | 0.00064004 |
| NGF | 76.83905692 | 1.535469118 | 0.279736645 | 5.488980966 | 4.04E-08 | 5.58E-06 |
| U1 | 20.22177655 | 1.5255092 | 0.305820779 | 4.988245747 | 6.09E-07 | 4.54E-05 |
| IRX5 | 153.0535145 | -1.654062439 | 0.400298325 | -4.132074345 | 3.60E-05 | 0.000941495 |
| CCL16 | 25.44893158 | 1.241596275 | 0.359828292 | 3.450524326 | 0.000559499 | 0.006678192 |
| RNU5A-1 | 10.23549466 | 1.007014732 | 0.300891066 | 3.34677511 | 0.000817575 | 0.00875861 |
| SNORD112 | 6.605818817 | 1.129609136 | 0.318582348 | 3.545736743 | 0.000391517 | 0.005182352 |
| CENPF | 5046.37388 | -1.031274343 | 0.215705097 | -4.780945648 | 1.74E-06 | 9.82E-05 |
| RP11-841O20.2 | 15.70312497 | -1.527323821 | 0.418172418 | -3.652378191 | 0.000259823 | 0.003875972 |
| CTC-297N7.7 | 4.785210409 | -1.622466322 | 0.513618916 | -3.158891294 | 0.001583706 | 0.014110675 |
| HMGA2 | 1946.940766 | -1.329590063 | 0.445956306 | -2.981435728 | 0.002869003 | 0.021668944 |
| EGLN3 | 3305.794872 | -1.935262793 | 0.280820099 | -6.891468241 | 5.52E-12 | 4.14E-09 |
| RN7SL463P | 7.675669167 | 1.917879575 | 0.435959353 | 4.39921649 | 1.09E-05 | 0.000382702 |
| CIDEC | 718.518479 | 3.335724512 | 0.439801003 | 7.584622333 | 3.33E-14 | 6.16E-11 |
| ACACB | 10190.15493 | 1.728156395 | 0.210560343 | 8.207416303 | 2.26E-16 | 1.26E-12 |
| MIR3188 | 4.082963395 | 2.260916854 | 0.413962094 | 5.461651887 | 4.72E-08 | 6.24E-06 |
| RNU12 | 188.3232091 | 1.586926204 | 0.230679941 | 6.879341997 | 6.01E-12 | 4.34E-09 |
| EBF3 | 1061.551686 | 1.240079852 | 0.251284879 | 4.934956131 | 8.02E-07 | 5.52E-05 |
| FAM74A3 | 3.289811647 | 2.124084236 | 0.745656739 | 2.848608649 | 0.004391086 | 0.029390729 |
| RNU6-80P | 2.507334175 | -1.064788175 | 0.326315179 | -3.263066643 | 0.001102136 | 0.010913609 |
| RP11-815M8.1 | 61.92315446 | -1.576986062 | 0.365025601 | -4.320206737 | 1.56E-05 | 0.000499171 |
| PKM | 27547.87564 | -1.014737827 | 0.167597328 | -6.054618181 | 1.41E-09 | 4.01E-07 |
| RN7SL600P | 188.0368169 | 1.016437104 | 0.185075057 | 5.492026441 | 3.97E-08 | 5.51E-06 |
| AC004840.8 | 9.381558014 | 1.442673951 | 0.536156885 | 2.690768303 | 0.007128769 | 0.041391652 |
| RP11-345I18.1 | 22.38269938 | 1.237885334 | 0.303133757 | 4.083627462 | 4.43E-05 | 0.0011076 |
| SPERT | 9.498220882 | -1.910032569 | 0.628824049 | -3.037467433 | 0.002385752 | 0.018951394 |
| CTD-2568A17.8 | 2.306169783 | 1.615975081 | 0.6021805 | 2.683539371 | 0.00728474 | 0.041966085 |
| WBSCR17 | 581.9867676 | 1.183654257 | 0.248746154 | 4.758482641 | 1.95E-06 | 0.000107125 |
| LMX1A | 6.611106784 | 3.358942392 | 0.803239963 | 4.181742125 | 2.89E-05 | 0.000798346 |
| RP11-440G5.2 | 7.176783843 | 1.449678419 | 0.503781488 | 2.877593666 | 0.004007209 | 0.027490907 |
| RP11-47A17.2 | 154.1296898 | 1.508813984 | 0.39651709 | 3.805167599 | 0.000141708 | 0.002554744 |
| NPAS4 | 27.13391327 | 1.080302845 | 0.368860368 | 2.928758251 | 0.00340319 | 0.024502842 |
| MIR377 | 2.984816573 | 1.742582791 | 0.361146298 | 4.825143721 | 1.40E-06 | 8.43E-05 |
| CTB-174D11.1 | 213.9023987 | 1.5895313 | 0.268726749 | 5.91504682 | 3.32E-09 | 7.94E-07 |
| CDR1 | 25661.78445 | 1.181217992 | 0.262401568 | 4.501566054 | 6.75E-06 | 0.00027168 |
| CHAD | 462.7998568 | 1.307582179 | 0.294102957 | 4.44600147 | 8.75E-06 | 0.000327391 |
| HS3ST1 | 2135.458188 | -1.248165272 | 0.246977201 | -5.053767179 | 4.33E-07 | 3.48E-05 |
| AP000445.1 | 5.367676454 | 2.128449111 | 0.682613776 | 3.118086955 | 0.001820291 | 0.015524045 |
| TP63 | 279.1917631 | -1.489678802 | 0.275187353 | -5.413325814 | 6.19E-08 | 7.66E-06 |
| MIR381HG | 19.74640946 | 1.056159319 | 0.287973953 | 3.667551554 | 0.000244884 | 0.003727267 |
| TPO | 177.4800274 | 1.54611368 | 0.336901402 | 4.589217118 | 4.45E-06 | 0.000201663 |
| NPR3 | 1874.054948 | -1.109040687 | 0.262945132 | -4.217764664 | 2.47E-05 | 0.000706356 |
| AC002075.3 | 11.74121121 | -2.195252226 | 0.79524026 | -2.760489297 | 0.005771485 | 0.035604976 |
| XPNPEP2 | 463.6280993 | 2.742386316 | 0.486564821 | 5.636219883 | 1.74E-08 | 2.94E-06 |
| COX6B2 | 208.1587997 | -1.212159097 | 0.340825802 | -3.5565356 | 0.000375778 | 0.00503728 |
| BTG2 | 20153.13171 | 1.181946352 | 0.236616189 | 4.995204919 | 5.88E-07 | 4.45E-05 |
| SLITRK1 | 133.3361881 | 1.415879076 | 0.448697898 | 3.155528655 | 0.001602075 | 0.014211312 |
| CYP2C8 | 305.0804313 | 1.755168085 | 0.359071657 | 4.888071926 | 1.02E-06 | 6.59E-05 |
| FST | 571.3874689 | 1.24737079 | 0.306684833 | 4.067272505 | 4.76E-05 | 0.001164096 |
| AC138517.4 | 4.025754386 | -2.30461384 | 0.76019955 | -3.031590638 | 0.002432689 | 0.019210753 |
| C4BPB | 407.9996937 | -1.27560691 | 0.257316881 | -4.957338611 | 7.15E-07 | 5.09E-05 |
| RP11-359K18.4 | 143.9802741 | 1.123565815 | 0.189445236 | 5.930821168 | 3.01E-09 | 7.30E-07 |
| CHRNE | 108.779551 | 1.043039023 | 0.212295208 | 4.913153861 | 8.96E-07 | 5.95E-05 |
| GRM7 | 83.15016581 | 1.006460231 | 0.298867216 | 3.367583248 | 0.000758301 | 0.008315511 |
| TNXB | 2352.177133 | 2.236019174 | 0.316849732 | 7.057033499 | 1.70E-12 | 1.49E-09 |
| U1 | 94.15589974 | 1.562576317 | 0.345803393 | 4.518684173 | 6.22E-06 | 0.000257041 |
| CAPN8 | 4187.779623 | -1.680081969 | 0.381559749 | -4.403194976 | 1.07E-05 | 0.000378282 |
| NXT1P1 | 2.312844935 | 1.562174498 | 0.567384927 | 2.753288683 | 0.005899985 | 0.036157457 |
| RP11-320N7.1 | 33.8071886 | 1.418688425 | 0.504632592 | 2.811329366 | 0.004933725 | 0.03191616 |
| RP11-248G5.8 | 18.543161 | 4.961854914 | 1.060233475 | 4.679964397 | 2.87E-06 | 0.000143628 |
| CNTFR-AS1 | 6.872683576 | 1.887650648 | 0.648628522 | 2.910218383 | 0.003611763 | 0.025593439 |
| RP11-469H8.6 | 88.62748235 | -1.470264252 | 0.446029671 | -3.296337324 | 0.000979543 | 0.010009772 |
| RP11-13N12.2 | 18.25211161 | -1.999176694 | 0.513336192 | -3.894478363 | 9.84E-05 | 0.001970985 |
| AL391319.1 | 369.5102224 | 1.733890438 | 0.539016408 | 3.216767453 | 0.001296436 | 0.012220096 |
| CTB-35F21.4 | 4.781539932 | 1.388320095 | 0.531796342 | 2.610623626 | 0.00903773 | 0.048941297 |
| SULT1C2 | 4124.370437 | -1.444460185 | 0.339879513 | -4.249918362 | 2.14E-05 | 0.000634542 |
| RP1-251M9.2 | 8.163593718 | 1.462265419 | 0.481667484 | 3.035840012 | 0.002398666 | 0.019026862 |
| FCER2 | 285.7061838 | 1.336750184 | 0.495582218 | 2.697332824 | 0.006989737 | 0.040778695 |
| VWCE | 160.7274211 | 1.823683318 | 0.274329142 | 6.647792883 | 2.98E-11 | 1.81E-08 |
| PSMC1P2 | 83.75039828 | -1.444396634 | 0.502072149 | -2.876870657 | 0.004016402 | 0.02751574 |
| TDH | 276.1624534 | 1.887307064 | 0.544371812 | 3.466944874 | 0.00052641 | 0.006390368 |
| KCNIP2 | 532.7999083 | 1.709757046 | 0.264362111 | 6.467481439 | 9.96E-11 | 4.74E-08 |
| CP | 7840.030509 | -1.542107915 | 0.313843355 | -4.913622966 | 8.94E-07 | 5.95E-05 |
| OASL | 981.9148441 | -1.104392999 | 0.250082252 | -4.416119059 | 1.00E-05 | 0.000363424 |
| RP11-286N3.2 | 12.34260319 | 1.572173399 | 0.403457861 | 3.89674747 | 9.75E-05 | 0.001955631 |
| CLCNKA | 103.9366573 | 1.168607328 | 0.380236395 | 3.073370522 | 0.002116555 | 0.017353883 |
| IGF2BP3 | 1266.306896 | -1.034641612 | 0.330852574 | -3.127198317 | 0.001764808 | 0.015191767 |
| MIR33B | 3.751272361 | 1.239852343 | 0.367064056 | 3.377754706 | 0.000730802 | 0.008089026 |
| KIF1A | 4446.884558 | 1.366059036 | 0.456461207 | 2.992716612 | 0.002765064 | 0.02110052 |
| RP11-384K6.2 | 291.5848777 | 1.210281257 | 0.448199882 | 2.700315875 | 0.006927367 | 0.0405422 |
| AL022344.7 | 12.86100795 | -1.161587354 | 0.295566954 | -3.930031204 | 8.49E-05 | 0.001758913 |
| PLIN1 | 4502.284752 | 3.800027723 | 0.466101605 | 8.152788318 | 3.56E-16 | 1.46E-12 |
| HBG2 | 34.91455552 | 1.34951223 | 0.274470721 | 4.916780287 | 8.80E-07 | 5.87E-05 |
| RP11-282E4.1 | 733.0144446 | -1.811277163 | 0.333925066 | -5.424202451 | 5.82E-08 | 7.32E-06 |
| CTB-66B24.1 | 10.30993037 | 1.537546372 | 0.304606439 | 5.047648956 | 4.47E-07 | 3.55E-05 |
| C16orf78 | 2.804063758 | 1.321298742 | 0.507733247 | 2.602348278 | 0.009258777 | 0.049785717 |
| FABP1 | 253.5816046 | 3.407353308 | 0.766205976 | 4.447046115 | 8.71E-06 | 0.000326951 |
| NOX1 | 57.71838997 | -1.829584563 | 0.371641759 | -4.922978974 | 8.52E-07 | 5.73E-05 |
| RP11-44F21.4 | 11.68305545 | -1.427585724 | 0.54239939 | -2.631982541 | 0.008488822 | 0.046780898 |
| RNF183 | 110.3344842 | -1.28821229 | 0.39721341 | -3.243123862 | 0.001182268 | 0.011479716 |
| MIR210HG | 220.2228087 | -1.405493043 | 0.284125412 | -4.946734728 | 7.55E-07 | 5.29E-05 |
| STAB2 | 1116.327168 | 1.05307011 | 0.398455972 | 2.642876962 | 0.00822049 | 0.045722153 |
| SHD | 23.22569777 | 1.684223872 | 0.474289602 | 3.551045318 | 0.000383704 | 0.005113654 |
| PADI3 | 40.46134422 | -1.534150568 | 0.54843387 | -2.797330091 | 0.005152686 | 0.032891759 |
| RP11-12A2.3 | 19.00739736 | 1.16239249 | 0.43484728 | 2.673105119 | 0.00751527 | 0.042889747 |
| ERICH1-AS1 | 9.395408718 | 1.511410788 | 0.580548102 | 2.603420429 | 0.009229869 | 0.049650813 |
| CMA1 | 87.23004727 | 1.678257295 | 0.298729642 | 5.617980478 | 1.93E-08 | 3.23E-06 |
| MIR218-2 | 3.03719125 | 1.536148009 | 0.42253167 | 3.635580759 | 0.000277355 | 0.004061382 |
| ANGPT4 | 37.66461265 | 1.284270004 | 0.340058905 | 3.776610425 | 0.000158977 | 0.002749234 |
| TRBV12-2 | 41.75399497 | 1.805493536 | 0.657202469 | 2.747240952 | 0.006009896 | 0.036600954 |
| CTD-2369P2.8 | 83.28407832 | 1.084091157 | 0.284299054 | 3.813207046 | 0.000137175 | 0.002498475 |
| BMPER | 296.5207873 | 1.881676326 | 0.325933978 | 5.773182471 | 7.78E-09 | 1.54E-06 |
| ASPHD2 | 581.6672516 | -1.008329232 | 0.207151619 | -4.867590389 | 1.13E-06 | 7.07E-05 |
| RCAN1 | 6573.511538 | 1.074256315 | 0.223903144 | 4.797861671 | 1.60E-06 | 9.29E-05 |
| SNORD39 | 29.61584608 | -1.802378044 | 0.408914465 | -4.407714079 | 1.04E-05 | 0.000373392 |
| NTSR1 | 655.9390795 | -1.808897727 | 0.431690871 | -4.190261715 | 2.79E-05 | 0.000779433 |
| LEP | 1059.103345 | 4.236976238 | 0.520118783 | 8.146170401 | 3.76E-16 | 1.46E-12 |
| GPAM | 5878.71846 | 2.392568824 | 0.303767171 | 7.876324548 | 3.37E-15 | 8.22E-12 |
| TEX15 | 65.57681259 | -1.610451 | 0.423541958 | -3.802341115 | 0.000143335 | 0.002571001 |
| RADIL | 389.4370169 | 1.440151342 | 0.249642206 | 5.768861617 | 7.98E-09 | 1.57E-06 |
| CD36 | 9183.738762 | 2.449907195 | 0.323487685 | 7.573417187 | 3.64E-14 | 6.16E-11 |
| LINC00941 | 258.0762894 | -1.406936368 | 0.275537601 | -5.106150164 | 3.29E-07 | 2.81E-05 |
| HPN-AS1 | 75.93982671 | 1.674031074 | 0.41114945 | 4.071587775 | 4.67E-05 | 0.00115315 |
| ERP27 | 10046.02503 | 1.344066544 | 0.477320661 | 2.815856621 | 0.004864737 | 0.031653667 |
| FAM81B | 59.78399553 | -1.41679738 | 0.328329368 | -4.315171044 | 1.59E-05 | 0.000507251 |
| SLC6A11 | 13.97775025 | 1.200363925 | 0.430825374 | 2.786195978 | 0.005333062 | 0.033618291 |
| DARC | 1068.40232 | 1.360541195 | 0.296944647 | 4.581800718 | 4.61E-06 | 0.000207339 |
| DUXAP10 | 53.20559631 | -1.365106445 | 0.442548321 | -3.084649472 | 0.002037922 | 0.016886689 |
| SLC1A7 | 250.2623804 | 1.061391566 | 0.234201889 | 4.531951339 | 5.84E-06 | 0.000246903 |
| CTC-296K1.4 | 21.475352 | 1.677748036 | 0.471295498 | 3.559864342 | 0.000371046 | 0.004989295 |
| HMGB1P27 | 12.56534258 | 1.263866538 | 0.34181211 | 3.697547577 | 0.000217692 | 0.003427096 |
| AQP12B | 2550.877466 | 2.313122487 | 0.622941827 | 3.713223908 | 0.000204636 | 0.003279806 |
| ABCA12 | 440.4128454 | -1.432875572 | 0.349649602 | -4.0980329 | 4.17E-05 | 0.001054399 |
| RP11-1124B17.1 | 24.76184645 | -1.171458781 | 0.319249955 | -3.669409379 | 0.000243111 | 0.003707522 |
| FAM228A | 24.66550669 | 1.374676811 | 0.361602722 | 3.801621857 | 0.000143752 | 0.002574069 |
| TNNT1 | 345.805275 | -2.792890446 | 0.493927848 | -5.654450259 | 1.56E-08 | 2.72E-06 |
| BRSK2 | 2086.559706 | 1.797807688 | 0.409041461 | 4.395172274 | 1.11E-05 | 0.000388144 |
| RP11-567G11.1 | 12.17613098 | -1.924050311 | 0.441517281 | -4.357814282 | 1.31E-05 | 0.000439716 |
| AC103801.1 | 35.41135173 | 1.473165219 | 0.401752381 | 3.666848753 | 0.000245558 | 0.003733151 |
| RP11-13N12.1 | 17.90791366 | -2.236541504 | 0.550180872 | -4.065102259 | 4.80E-05 | 0.001173061 |
| CYP4Z2P | 13.06966727 | 1.330352007 | 0.457263847 | 2.909375007 | 0.003621522 | 0.025648608 |
| SFRP1 | 5591.433019 | 2.179164644 | 0.346401651 | 6.290861028 | 3.16E-10 | 1.14E-07 |
| Y_RNA | 3.034249017 | 1.329712677 | 0.371929611 | 3.575172929 | 0.000349996 | 0.004788811 |
| HMGN2P15 | 21.50290953 | 1.168262371 | 0.297037264 | 3.933049865 | 8.39E-05 | 0.001742761 |
| LINC00589 | 30.3284064 | -1.584961669 | 0.524187206 | -3.023655764 | 0.002497404 | 0.019563335 |
| SHISA2 | 731.0569418 | -1.101936579 | 0.274267627 | -4.017742056 | 5.88E-05 | 0.001353032 |
| ERN2 | 1727.564583 | -1.362335882 | 0.382626184 | -3.560487859 | 0.000370166 | 0.004982616 |
| PRRT4 | 50.64936066 | 2.386322582 | 0.456295675 | 5.229772518 | 1.70E-07 | 1.70E-05 |
| PTPRQ | 171.7935796 | 1.631465664 | 0.330851993 | 4.931104231 | 8.18E-07 | 5.60E-05 |
| RP11-680B3.2 | 5284.62007 | 1.5262335 | 0.57610118 | 2.649245572 | 0.008067169 | 0.045133322 |
| MIR485 | 2.3185022 | 1.241525622 | 0.372349859 | 3.334298618 | 0.000855148 | 0.00908129 |
| LINC01043 | 4.952191681 | 4.40237412 | 1.183195168 | 3.720750591 | 0.000198632 | 0.003208632 |
| FGF20 | 38.73926914 | -2.169395355 | 0.379792048 | -5.712061024 | 1.12E-08 | 2.04E-06 |
| IL11 | 130.3739903 | -1.062502806 | 0.337010286 | -3.152731089 | 0.001617507 | 0.014301783 |
| FOXD1 | 208.2361554 | -1.084722543 | 0.392169588 | -2.765952733 | 0.005675676 | 0.035201826 |
| AC105053.3 | 11.33524423 | 1.017826223 | 0.357486997 | 2.847169919 | 0.004410981 | 0.029483409 |
| AF067845.1 | 6.472080828 | 1.405591925 | 0.477568452 | 2.943226086 | 0.003248111 | 0.02374623 |
| RP11-635N19.1 | 67.42104094 | 1.024564435 | 0.194684898 | 5.262680601 | 1.42E-07 | 1.46E-05 |
| FCN2 | 18.75679194 | 2.556235837 | 0.542929212 | 4.708230426 | 2.50E-06 | 0.000129664 |
| SNORD113-8 | 12.24880495 | 1.130770655 | 0.274326439 | 4.121989328 | 3.76E-05 | 0.000974524 |
| RP11-20J15.3 | 28.32987582 | 1.626126167 | 0.326055932 | 4.987261405 | 6.12E-07 | 4.55E-05 |
| C20orf85 | 9.911924687 | -2.58028914 | 0.829361963 | -3.111173714 | 0.001863453 | 0.015779662 |
| RN7SL494P | 3.747372849 | 1.865807144 | 0.581491963 | 3.208655084 | 0.001333574 | 0.012443816 |
| C2orf40 | 195.6004752 | 1.343739525 | 0.284156782 | 4.72886664 | 2.26E-06 | 0.00012044 |
| RP11-99A1.2 | 55.82217697 | -1.2300918 | 0.253742136 | -4.847802658 | 1.25E-06 | 7.67E-05 |
| RP11-234K19.1 | 10.12433226 | 1.357698696 | 0.396233476 | 3.426511841 | 0.000611387 | 0.007150882 |
| ABCC6P1 | 263.4925927 | 1.696830436 | 0.38540467 | 4.402724121 | 1.07E-05 | 0.000378282 |
| RP11-48O20.4 | 550.3429963 | -1.179782444 | 0.317908913 | -3.711070674 | 0.000206384 | 0.003299698 |
| COL25A1 | 364.2566762 | 2.434086735 | 0.310449975 | 7.840511938 | 4.49E-15 | 9.72E-12 |
| CTA-384D8.31 | 39.97214545 | -2.199545436 | 0.48699396 | -4.516576416 | 6.28E-06 | 0.000259063 |
| IMPA2 | 3982.030313 | 1.384945217 | 0.338896771 | 4.086628537 | 4.38E-05 | 0.00109689 |
| CYP2T3P | 9.100082216 | -1.070767996 | 0.353030634 | -3.033073882 | 0.002420763 | 0.019135954 |
| GPR42 | 14.58449245 | 1.125218196 | 0.333878055 | 3.370147208 | 0.00075128 | 0.008264084 |
| RP11-326L17.1 | 68.36520444 | 1.222210865 | 0.4535451 | 2.694794554 | 0.007043204 | 0.041023115 |
| DBX2 | 46.25011761 | 1.312456311 | 0.421255198 | 3.115584845 | 0.001835805 | 0.01560704 |
| CACNG8 | 235.9210138 | -1.840861019 | 0.374180636 | -4.919712151 | 8.67E-07 | 5.81E-05 |
| P2RX1 | 1930.810641 | 1.277029536 | 0.333515071 | 3.829000984 | 0.000128664 | 0.002389177 |
| LAMC2 | 23812.78548 | -1.554332239 | 0.323953248 | -4.798014062 | 1.60E-06 | 9.29E-05 |
| GFRA3 | 140.5088922 | 1.063858245 | 0.319638659 | 3.328315316 | 0.000873729 | 0.009230755 |
| GPX3 | 5817.803216 | 1.123703904 | 0.241147305 | 4.659823604 | 3.16E-06 | 0.000154072 |
| RP11-475B2.1 | 6.921782994 | -1.150208312 | 0.419643025 | -2.740920837 | 0.006126727 | 0.037121148 |
| MMP28 | 1543.738057 | -1.070297499 | 0.281956429 | -3.795967706 | 0.000147069 | 0.002613921 |
| IGFL3 | 15.89233047 | -1.486448275 | 0.448516441 | -3.314144456 | 0.00091924 | 0.009571633 |
| RPL17P3 | 5.590394606 | 1.437028399 | 0.50348939 | 2.854138394 | 0.004315373 | 0.029018445 |
| NPHS1 | 1009.089806 | 1.645013584 | 0.508071453 | 3.23776031 | 0.00120472 | 0.011611768 |
| LDB3 | 359.057166 | 2.245969945 | 0.274854557 | 8.171485201 | 3.05E-16 | 1.46E-12 |
| IGKV2-28 | 1437.111784 | -1.021068511 | 0.330165142 | -3.092599376 | 0.001984118 | 0.016518078 |
| DMRT3 | 16.26227439 | 2.709737818 | 0.617262648 | 4.389926762 | 1.13E-05 | 0.000395492 |
| RP11-138I17.1 | 12.90180182 | 2.723283433 | 0.767649256 | 3.547562133 | 0.000388814 | 0.005158831 |
| MAGEA5 | 14.87074816 | -1.274930918 | 0.432037742 | -2.950971161 | 0.003167765 | 0.023332741 |
| ZNF385D-AS1 | 25.65717257 | 1.299159859 | 0.306124982 | 4.243887087 | 2.20E-05 | 0.000645065 |
| SPINT4 | 15.78457031 | -1.987666253 | 0.502757233 | -3.953530895 | 7.70E-05 | 0.001633766 |
| GOLGA6D | 4.898982501 | 1.776784366 | 0.613869283 | 2.894401815 | 0.003798817 | 0.026509461 |
| MIR4454 | 102.8177196 | -2.305584063 | 0.475482291 | -4.848937819 | 1.24E-06 | 7.63E-05 |
| AC010969.1 | 41.38612485 | 1.702014061 | 0.417842678 | 4.073337046 | 4.63E-05 | 0.001148155 |
| RAET1L | 51.23460065 | -1.714562298 | 0.483269974 | -3.547835352 | 0.000388411 | 0.005157219 |
| AC093375.1 | 351.3544529 | -1.044899864 | 0.301424017 | -3.466544818 | 0.000527194 | 0.006392389 |
| RNF5P1 | 44.10313551 | 1.142030021 | 0.307005698 | 3.719898456 | 0.000199303 | 0.003216811 |
| RP11-386G11.10 | 586.0835072 | -1.352418371 | 0.323075982 | -4.186069051 | 2.84E-05 | 0.000786627 |
| CXorf67 | 31.62837152 | 1.077827538 | 0.333698142 | 3.229947673 | 0.001238129 | 0.011835786 |
| MTND2P26 | 84.89537865 | -1.013431012 | 0.277376172 | -3.653634002 | 0.000258555 | 0.003862967 |
| OR56B1 | 28.33991737 | 1.1478552 | 0.341487999 | 3.361333935 | 0.00077567 | 0.008465504 |
| EXO1 | 302.4238457 | -1.002281203 | 0.209737472 | -4.778741696 | 1.76E-06 | 9.90E-05 |
| RP11-116B13.1 | 8.271305652 | 1.846234337 | 0.531992512 | 3.47041414 | 0.000519656 | 0.00632657 |
| S100P | 2643.392294 | -1.535115908 | 0.428602119 | -3.581680627 | 0.000341391 | 0.004704078 |
| CTD-2377D24.8 | 104.9173742 | -3.142795238 | 0.575645711 | -5.45959985 | 4.77E-08 | 6.24E-06 |
| PRSS3 | 37182.01795 | 1.775739918 | 0.571691565 | 3.106115299 | 0.001895628 | 0.01594866 |
| RP11-492E3.2 | 66.64536911 | -1.04975818 | 0.354023365 | -2.965222877 | 0.003024637 | 0.022556077 |
| KLK7 | 956.5141235 | -1.672285028 | 0.523558337 | -3.194075828 | 0.001402793 | 0.012895309 |
| LINC00473 | 316.7730865 | 1.361277357 | 0.276554674 | 4.922272094 | 8.55E-07 | 5.74E-05 |
| MIR141 | 3.517182243 | -1.112424231 | 0.345489408 | -3.21985047 | 0.001282575 | 0.012136904 |
| RP11-830F9.6 | 82.29829732 | 1.155473025 | 0.258336489 | 4.472744173 | 7.72E-06 | 0.000299031 |
| EMP1 | 5764.959689 | 1.179031162 | 0.2483425 | 4.747601249 | 2.06E-06 | 0.000111951 |
| MATN4 | 394.640904 | 2.210888886 | 0.486604668 | 4.543501185 | 5.53E-06 | 0.00023761 |
| MIR27A | 31.41436809 | 1.141568735 | 0.295277951 | 3.866081876 | 0.000110598 | 0.002147919 |
| USHBP1 | 332.4790158 | 1.124452363 | 0.189562823 | 5.931819037 | 3.00E-09 | 7.30E-07 |
| MIR770 | 39.98649201 | 1.608131118 | 0.269384117 | 5.969658267 | 2.38E-09 | 6.10E-07 |
| LGI4 | 396.644409 | 1.112841466 | 0.216726236 | 5.134779658 | 2.82E-07 | 2.49E-05 |
| RP4-609E1.2 | 9.843443036 | 1.423085372 | 0.346763614 | 4.10390628 | 4.06E-05 | 0.001033335 |
| CTD-2363C16.2 | 100.8684201 | 1.133513876 | 0.252598124 | 4.487420018 | 7.21E-06 | 0.00028353 |
| PHACTR3 | 580.9757031 | -1.301621092 | 0.395579337 | -3.290417295 | 0.001000389 | 0.010177451 |
| CTC-327F10.4 | 49.49266409 | -1.33802069 | 0.401128228 | -3.335643308 | 0.000851023 | 0.009042407 |
| GPT | 539.6500087 | 1.137274974 | 0.256144798 | 4.439969037 | 9.00E-06 | 0.000334457 |
| CACNA1G | 187.5752336 | 1.860084874 | 0.313572236 | 5.93191826 | 2.99E-09 | 7.30E-07 |
| RP11-598O12.1 | 7.352197795 | 1.219800596 | 0.439089798 | 2.778020809 | 0.005469112 | 0.034221439 |
| RP5-1086L22.1 | 14.7584726 | 2.285054574 | 0.795715892 | 2.871696539 | 0.004082748 | 0.027843086 |
| RP11-264M12.2 | 23.25145334 | 1.477044203 | 0.313735373 | 4.707930095 | 2.50E-06 | 0.000129664 |
| DNAJB1P1 | 11.23058273 | 1.727146286 | 0.366727958 | 4.709611707 | 2.48E-06 | 0.000129387 |
| HAPLN1 | 189.5921445 | -2.824651802 | 0.517068612 | -5.462818151 | 4.69E-08 | 6.22E-06 |
| RHBG | 36.24225775 | 1.973242636 | 0.564370814 | 3.496358398 | 0.000471655 | 0.005902496 |
| C16orf89 | 519.9221561 | 1.544790735 | 0.308357871 | 5.009733429 | 5.45E-07 | 4.17E-05 |
| KCNA7 | 75.88323411 | -1.632803575 | 0.369876274 | -4.414458804 | 1.01E-05 | 0.000365047 |
| NGB | 7.034256566 | 3.631295175 | 0.969826093 | 3.744274568 | 0.000180916 | 0.003018815 |
| RP11-720L2.3 | 15.38053306 | 1.04828266 | 0.376486216 | 2.784385233 | 0.00536293 | 0.033719443 |
| PSG7 | 14.06420608 | 1.181199721 | 0.377157921 | 3.131843862 | 0.001737122 | 0.015056476 |
| ADIPOQ-AS1 | 40.8111474 | 3.748480703 | 0.98882008 | 3.79086224 | 0.000150125 | 0.002648929 |
| RP11-785F11.1 | 7.499544272 | 1.972340313 | 0.725455 | 2.718763138 | 0.006552651 | 0.038955954 |
| HPD | 103.0221976 | 1.001625647 | 0.247930491 | 4.039945392 | 5.35E-05 | 0.001268907 |
| ANKRD62 | 488.4259527 | 1.325419224 | 0.485019504 | 2.732713245 | 0.006281499 | 0.037812144 |
| MIR5002 | 10.41528881 | 1.071258302 | 0.252870798 | 4.236385976 | 2.27E-05 | 0.000660519 |
| RP11-357H14.17 | 150.6965515 | -1.779153014 | 0.42479509 | -4.188261722 | 2.81E-05 | 0.000783172 |
| RP5-855F14.1 | 131.2756919 | 1.41079174 | 0.311624626 | 4.527215193 | 5.98E-06 | 0.000250331 |
| AC135178.7 | 10.9551107 | 1.44586932 | 0.405175079 | 3.568505064 | 0.000359024 | 0.004875418 |
| HOXB9 | 169.5139447 | -2.283604438 | 0.407975314 | -5.597408376 | 2.18E-08 | 3.52E-06 |
| AP000438.4 | 2.392143735 | 2.099060508 | 0.779959402 | 2.69124329 | 0.007118626 | 0.041357393 |
| LRRN4CL | 437.4674991 | 1.434975484 | 0.276743421 | 5.185219858 | 2.16E-07 | 2.08E-05 |
| TRBV23OR9-2 | 5.708961741 | 2.510839798 | 0.860393191 | 2.918246941 | 0.003520055 | 0.025126218 |
| CBLN1 | 118.248342 | 1.574843253 | 0.381665229 | 4.126242408 | 3.69E-05 | 0.000959877 |
| DMRT2 | 23.35258275 | 1.6847317 | 0.527629354 | 3.193021173 | 0.001407926 | 0.012930307 |
| PTX3 | 695.0061386 | 1.67900606 | 0.338526237 | 4.95975165 | 7.06E-07 | 5.04E-05 |
| RP11-231L11.1 | 11.52040992 | 1.061697954 | 0.380825257 | 2.787887452 | 0.005305297 | 0.033519506 |
| CR1 | 3149.92127 | 1.077435036 | 0.288964522 | 3.728606645 | 0.000192541 | 0.003136238 |
| IL13RA2 | 72.21228089 | 1.610848464 | 0.397457139 | 4.05288598 | 5.06E-05 | 0.001219259 |
| RP11-1080G15.1 | 53.80160923 | 1.297068519 | 0.292784688 | 4.430110493 | 9.42E-06 | 0.000345507 |
| RP4-533D7.3 | 18.95365474 | 1.045407883 | 0.283104797 | 3.692653366 | 0.000221926 | 0.003474568 |
| MIR212 | 17.26911349 | 2.192969275 | 0.389122903 | 5.635672587 | 1.74E-08 | 2.94E-06 |
| HOXB-AS3 | 170.1290176 | -1.193938553 | 0.271046169 | -4.40492687 | 1.06E-05 | 0.000376843 |
| CYP3A43 | 130.689998 | 2.908691574 | 0.496041502 | 5.863806886 | 4.52E-09 | 9.85E-07 |
| MIR300 | 3.854900795 | 1.12055794 | 0.33934781 | 3.302092737 | 0.000959663 | 0.009879111 |
| HEMGN | 47.11424295 | 2.151666888 | 0.456168448 | 4.716825322 | 2.40E-06 | 0.000125895 |
| PCOLCE2 | 572.7463508 | 1.633923272 | 0.360186286 | 4.536328383 | 5.72E-06 | 0.000242362 |
| AC066593.1 | 105.5361066 | 2.049072865 | 0.632733993 | 3.238442834 | 0.001201841 | 0.011594703 |
| AC011625.1 | 7.290798142 | 1.665556916 | 0.528842944 | 3.149435829 | 0.00163586 | 0.014406137 |
| FGF10 | 820.9599567 | 1.912558697 | 0.302680406 | 6.3187397 | 2.64E-10 | 9.98E-08 |
| RPL21P23 | 65.78625821 | 1.581135588 | 0.196416542 | 8.04991053 | 8.29E-16 | 2.37E-12 |
| COL10A1 | 12817.61204 | -1.150907646 | 0.261545747 | -4.400406659 | 1.08E-05 | 0.000381224 |
| THBD | 4026.244179 | 1.00260129 | 0.198730137 | 5.045039 | 4.53E-07 | 3.58E-05 |
| DCC | 143.2128516 | -1.203426615 | 0.333630927 | -3.607059531 | 0.000309687 | 0.00438976 |
| GRIN2C | 103.8374472 | 1.114039528 | 0.177800593 | 6.265668238 | 3.71E-10 | 1.30E-07 |
| CTD-2377D24.6 | 328.4139613 | -3.105209925 | 0.521787787 | -5.951097367 | 2.66E-09 | 6.70E-07 |
| CTRC | 280039.8706 | 2.029462181 | 0.630298199 | 3.219844485 | 0.001282602 | 0.012136904 |
| WT1-AS | 242.2503773 | 1.080375781 | 0.309885441 | 3.486371536 | 0.00048962 | 0.006064128 |
| CRYAA | 9.092424455 | 1.556110665 | 0.443752645 | 3.506707353 | 0.000453688 | 0.005734702 |
| CCKBR | 191.5080228 | 1.927039146 | 0.580198412 | 3.321345088 | 0.000895847 | 0.009393265 |
| RP11-93K22.13 | 15.62531818 | -1.477321207 | 0.416772912 | -3.544667045 | 0.000393109 | 0.005194611 |
| AC108479.2 | 5.803807028 | 1.15282711 | 0.408498735 | 2.822106925 | 0.004770927 | 0.031205842 |
| WTAPP1 | 317.6435083 | -1.77474879 | 0.392012467 | -4.527276399 | 5.97E-06 | 0.000250331 |
| NDNF | 777.7733154 | -1.593346253 | 0.290835693 | -5.478510003 | 4.29E-08 | 5.79E-06 |
| FTCD | 112.8190163 | 1.42412494 | 0.32820023 | 4.339195434 | 1.43E-05 | 0.000468789 |
| RP11-796E10.1 | 5.297371352 | -2.304284311 | 0.796190214 | -2.894137948 | 0.003802011 | 0.026524336 |
| C8orf86 | 8.445183976 | 1.12665759 | 0.406343595 | 2.772672204 | 0.005559809 | 0.034661034 |
| NR4A2 | 3249.234534 | 1.66758959 | 0.279472619 | 5.966915821 | 2.42E-09 | 6.16E-07 |
| ABCA13 | 612.875807 | -1.27142551 | 0.379008545 | -3.354609085 | 0.000794772 | 0.008611319 |
| KIF25 | 11.9749886 | 1.239745516 | 0.382002916 | 3.245382332 | 0.00117293 | 0.011415836 |
| GALNT5 | 4676.921917 | -1.38359317 | 0.360312487 | -3.839981185 | 0.000123044 | 0.002317918 |
| LINC00311 | 42.99178973 | 1.015627301 | 0.227436158 | 4.465548969 | 7.99E-06 | 0.000306524 |
| MIR208B | 7.016318172 | 1.68690777 | 0.629660717 | 2.679074181 | 0.007382604 | 0.042313245 |
| KRT15 | 605.8270783 | -1.634426848 | 0.369075379 | -4.42843641 | 9.49E-06 | 0.000347872 |
| CLU | 19366.31194 | 1.048125421 | 0.203516749 | 5.150069593 | 2.60E-07 | 2.35E-05 |
| AHNAK2 | 20865.652 | -1.310293264 | 0.248951032 | -5.26325701 | 1.42E-07 | 1.46E-05 |
| RP11-238F2.1 | 3.631997603 | 3.33973887 | 1.004071774 | 3.326195356 | 0.000880402 | 0.009276216 |
| WT1 | 508.5480393 | 1.37306788 | 0.34661385 | 3.961376268 | 7.45E-05 | 0.001594003 |
| AVPR1A | 522.8252924 | 1.551026531 | 0.255746912 | 6.064693087 | 1.32E-09 | 3.82E-07 |
| CADM3 | 746.0002984 | 1.483390107 | 0.331369952 | 4.476537776 | 7.59E-06 | 0.000295533 |
| ADAMTS9-AS1 | 518.5137826 | 1.453314792 | 0.243017743 | 5.980282642 | 2.23E-09 | 5.83E-07 |
| CTD-2299I21.1 | 5.011505943 | 1.729842553 | 0.653207919 | 2.648226547 | 0.008091528 | 0.045223645 |
| GPIHBP1 | 307.3026852 | 1.828068049 | 0.299505997 | 6.103610843 | 1.04E-09 | 3.16E-07 |
| TFF3 | 1251.881904 | -1.146226489 | 0.389871905 | -2.940007924 | 0.003282039 | 0.023917116 |
| H19 | 16013.10779 | 1.901168355 | 0.261774058 | 7.262630874 | 3.80E-13 | 4.18E-10 |
| CHCHD3P2 | 42.62842989 | 1.91036649 | 0.581488214 | 3.285305609 | 0.001018718 | 0.010318159 |
| RP11-129B9.2 | 43.69417481 | -1.030174471 | 0.184529291 | -5.58271517 | 2.37E-08 | 3.78E-06 |
| SFN | 2810.742827 | -1.438134513 | 0.391274379 | -3.67551414 | 0.000237371 | 0.003639905 |
| AC017104.2 | 11.67830839 | 1.148236206 | 0.341515169 | 3.362182154 | 0.000773291 | 0.008446633 |
| CTC-497E21.3 | 8.029341199 | -1.803751751 | 0.577513699 | -3.123305566 | 0.00178832 | 0.015321992 |
| AL035610.1 | 156.9917742 | 1.751691163 | 0.298893349 | 5.860589311 | 4.61E-09 | 9.99E-07 |
| SERPINB4 | 116.9654631 | -2.149667363 | 0.664753705 | -3.233780188 | 0.001221634 | 0.011710333 |
| ADCK3 | 5296.131765 | 1.05512174 | 0.216488129 | 4.873808768 | 1.09E-06 | 6.92E-05 |
| PODN | 7077.151557 | 1.062561563 | 0.214640068 | 4.950434346 | 7.40E-07 | 5.22E-05 |
| EPHX4 | 116.3711044 | -1.240747282 | 0.304708859 | -4.07191076 | 4.66E-05 | 0.001152281 |
| CLDN18 | 8608.067833 | -1.148092064 | 0.435185208 | -2.638168861 | 0.008335506 | 0.046157778 |
| MMP12 | 1286.523427 | -1.602424332 | 0.370926376 | -4.320060354 | 1.56E-05 | 0.000499171 |
| ADAMTS20 | 12.15455389 | -1.579621339 | 0.590584074 | -2.674676489 | 0.007480139 | 0.042731912 |
| BCHE | 496.9133326 | 1.307868846 | 0.279793491 | 4.674407694 | 2.95E-06 | 0.000146444 |
| AL117190.3 | 74.18922894 | 1.462801454 | 0.27598558 | 5.300282195 | 1.16E-07 | 1.25E-05 |
| CCL3 | 714.4400372 | 1.20463443 | 0.283055883 | 4.25581839 | 2.08E-05 | 0.000622861 |
| AC002398.5 | 22.89316086 | 1.557117392 | 0.405778278 | 3.837360148 | 0.000124364 | 0.002334903 |
| RP11-17A4.2 | 69.99888831 | 2.134638494 | 0.401880186 | 5.311629101 | 1.09E-07 | 1.19E-05 |
| GYG2 | 510.8832658 | 1.639085639 | 0.235776341 | 6.951866461 | 3.60E-12 | 2.81E-09 |
| ADIRF | 2092.146309 | 1.124914041 | 0.304499534 | 3.694304636 | 0.000220489 | 0.003462739 |
| MOCS1 | 1313.280474 | 1.018939062 | 0.154859259 | 6.579774883 | 4.71E-11 | 2.70E-08 |
| BAK1P2 | 2.821469263 | 2.372337531 | 0.85084316 | 2.788219548 | 0.005299861 | 0.0335065 |
| C2CD4B | 1792.438048 | 1.125280618 | 0.37194378 | 3.025405127 | 0.002483003 | 0.019481831 |
| KLF5 | 9277.277033 | -1.324756266 | 0.281368397 | -4.708262476 | 2.50E-06 | 0.000129664 |
| PSG11 | 8.13717705 | 1.350316423 | 0.49240018 | 2.742315045 | 0.00610078 | 0.03699843 |
| RYR3 | 474.5258419 | 1.105206434 | 0.199682401 | 5.53482145 | 3.12E-08 | 4.60E-06 |
| AC114812.9 | 22.43384417 | -2.709335695 | 0.498229972 | -5.437921939 | 5.39E-08 | 6.98E-06 |
| CTSE | 15622.11504 | -1.554927917 | 0.404858727 | -3.8406679 | 0.0001227 | 0.002314798 |
| CTD-2281E23.2 | 2.792064613 | 1.847852855 | 0.683895362 | 2.701952606 | 0.006893359 | 0.040385596 |
| C19orf69 | 14.99040451 | 2.434749663 | 0.556736289 | 4.373254823 | 1.22E-05 | 0.00041871 |
| RP11-293M10.2 | 10.43401632 | 1.380340866 | 0.315652816 | 4.372971811 | 1.23E-05 | 0.000418842 |
| RP11-494M8.1 | 4.898087 | -2.016601268 | 0.711395677 | -2.834711165 | 0.004586713 | 0.030304793 |
| RP11-172E9.2 | 20.36397606 | 1.272218787 | 0.314794483 | 4.041426569 | 5.31E-05 | 0.001263224 |
| ANO1-AS2 | 53.14964659 | -1.055786989 | 0.348149481 | -3.032568042 | 0.002424824 | 0.019160288 |
| RP11-634B7.4 | 13.93778203 | 1.406971247 | 0.429055392 | 3.279229845 | 0.001040908 | 0.010485718 |
| RNA5SP78 | 2.543238188 | 1.235952618 | 0.363991767 | 3.395551027 | 0.000684906 | 0.007741424 |
| S100A11P1 | 5.429616574 | -1.17744274 | 0.444623021 | -2.648182135 | 0.008092591 | 0.045223645 |
| MTTP | 40.84911364 | 1.979776091 | 0.388353236 | 5.097874585 | 3.43E-07 | 2.91E-05 |
| RP11-460N11.3 | 554.9507942 | -1.691269529 | 0.358836276 | -4.713206667 | 2.44E-06 | 0.000127577 |
| RP11-597K23.2 | 9.163274842 | -1.594762225 | 0.498274932 | -3.200566844 | 0.001371576 | 0.012700583 |
| CDH23 | 2256.2986 | 1.204267665 | 0.206399927 | 5.834632228 | 5.39E-09 | 1.12E-06 |
| ATP1B2 | 424.7358368 | 1.093170888 | 0.201897986 | 5.414471481 | 6.15E-08 | 7.66E-06 |
| U3 | 110.1265901 | 1.064539201 | 0.295389908 | 3.603844185 | 0.000313545 | 0.004428356 |
| SLC26A9 | 1596.358906 | -1.888354275 | 0.428248325 | -4.409484326 | 1.04E-05 | 0.000372282 |
| HOXB-AS4 | 27.45807611 | -1.640015761 | 0.532635262 | -3.079059683 | 0.002076551 | 0.017119471 |
| RP11-384P7.5 | 27.57410813 | 2.402009825 | 0.817022279 | 2.939956335 | 0.003282585 | 0.023917116 |
| RN7SL377P | 4.940393123 | 1.192257133 | 0.448942412 | 2.655701715 | 0.007914358 | 0.044559689 |
| SNORD114-15 | 12.36273732 | 1.013212691 | 0.288155217 | 3.516204567 | 0.000437764 | 0.005589025 |
| SLC6A14 | 5113.945709 | -1.655889037 | 0.460876865 | -3.592909875 | 0.000327006 | 0.004559023 |
| GAPDHP49 | 96.7241826 | -1.211757169 | 0.259335192 | -4.672551988 | 2.97E-06 | 0.000147398 |
| NMUR1 | 197.1725946 | 1.137681187 | 0.227847666 | 4.993165865 | 5.94E-07 | 4.47E-05 |
| NAT2 | 42.41275629 | 2.248906976 | 0.574937715 | 3.911566272 | 9.17E-05 | 0.00185989 |
| SIGLEC30P | 4.679066963 | -2.915125728 | 0.749805992 | -3.887840001 | 0.00010114 | 0.002010176 |
| FAM83A | 1131.712258 | -1.432513941 | 0.470211962 | -3.046528071 | 0.002315009 | 0.018532901 |
| CGB2 | 7.047346244 | -1.591994606 | 0.575315604 | -2.76716744 | 0.00565457 | 0.035111461 |
| RP11-473L15.2 | 81.53907318 | 1.341519649 | 0.308169544 | 4.353186982 | 1.34E-05 | 0.000446743 |
| NEK2 | 358.3571637 | -1.232698829 | 0.263224377 | -4.683072453 | 2.83E-06 | 0.000142253 |
| AC007128.1 | 7.062357921 | -2.178106705 | 0.723089751 | -3.012221791 | 0.00259343 | 0.02011261 |
| RP11-164N3.1 | 3.311925826 | -2.371572463 | 0.689084791 | -3.441626481 | 0.000578228 | 0.006858749 |
| MYL3 | 51.76141742 | 1.005427935 | 0.236097003 | 4.258537468 | 2.06E-05 | 0.000618321 |
| LGALS4 | 5706.16449 | -1.113131543 | 0.364990286 | -3.049756624 | 0.002290269 | 0.018414233 |
| CDCP1 | 6567.600038 | -1.112720742 | 0.260260359 | -4.275413849 | 1.91E-05 | 0.000584412 |
| DRD1 | 133.7785286 | -1.087912178 | 0.352532057 | -3.085995034 | 0.002028722 | 0.016824759 |
| LIPT1P1 | 15.96528055 | 1.35922841 | 0.409453235 | 3.319618198 | 0.000901406 | 0.009433801 |
| C14orf180 | 155.0299992 | 3.498593877 | 0.445127596 | 7.859755057 | 3.85E-15 | 8.83E-12 |
| AC093850.2 | 369.1463828 | -1.103586916 | 0.30068004 | -3.670303206 | 0.000242263 | 0.003699474 |
| RP11-865I6.2 | 12.95601222 | -1.359245736 | 0.486217564 | -2.795550461 | 0.005181141 | 0.033012844 |
| ACSL1 | 10253.21514 | 1.196265773 | 0.204363644 | 5.853613449 | 4.81E-09 | 1.02E-06 |
| PLIN5 | 2129.545131 | 2.056335556 | 0.378851256 | 5.427817707 | 5.70E-08 | 7.20E-06 |
| ECE2 | 827.0530269 | 1.168585428 | 0.343235812 | 3.404613935 | 0.000662577 | 0.007554742 |
| EPHX3 | 103.0563112 | -1.167481868 | 0.302277363 | -3.862286793 | 0.000112331 | 0.002174941 |
| CTD-3110P2.2 | 7.964407457 | 3.232540388 | 1.050100494 | 3.078315272 | 0.002081745 | 0.017134072 |
| EPO | 47.54410709 | 1.302196652 | 0.481449072 | 2.704744337 | 0.006835698 | 0.040207428 |
| RP11-342D14.1 | 14.23523039 | 1.078496434 | 0.257848877 | 4.18266873 | 2.88E-05 | 0.000796225 |
| CST1 | 3248.359832 | -1.688888978 | 0.438911098 | -3.847906756 | 0.000119131 | 0.002270755 |
| RP11-483G21.3 | 8.985368052 | 1.791015193 | 0.683610408 | 2.619935525 | 0.008794639 | 0.047911003 |
| RP11-326C3.10 | 2.55756674 | 1.123829836 | 0.404888146 | 2.775655071 | 0.005509062 | 0.034427223 |
| RN7SL422P | 3.982990215 | 1.17680096 | 0.346851919 | 3.392805102 | 0.000691808 | 0.007783344 |
| STEAP4 | 6277.560652 | 1.082874897 | 0.240962677 | 4.493952797 | 6.99E-06 | 0.000278474 |
| CXCR5 | 150.3427452 | 1.288580651 | 0.450420092 | 2.860841855 | 0.004225178 | 0.028554731 |
| FHL1 | 7139.300733 | 1.692667226 | 0.326655583 | 5.181810187 | 2.20E-07 | 2.10E-05 |
| RP11-88H9.2 | 127.5703084 | 1.070895224 | 0.246169405 | 4.350236876 | 1.36E-05 | 0.000450548 |
| PPP1R14C | 242.260931 | -1.457954371 | 0.343184038 | -4.248316384 | 2.15E-05 | 0.000635634 |
| LAMB3 | 15912.25483 | -1.354013325 | 0.329231078 | -4.112653444 | 3.91E-05 | 0.001004769 |
| RP11-116O18.1 | 17.09732351 | 1.036926928 | 0.299098018 | 3.466846534 | 0.000526602 | 0.006390368 |
| CCL14 | 788.6140499 | 1.901914114 | 0.300726013 | 6.324408363 | 2.54E-10 | 9.72E-08 |
| FIGF | 175.5619239 | 2.302516143 | 0.36932892 | 6.234323982 | 4.54E-10 | 1.53E-07 |
| SNORD5 | 3.711903965 | 2.635238506 | 0.43523771 | 6.054710898 | 1.41E-09 | 4.01E-07 |
| MST1R | 2918.652954 | -1.060679394 | 0.279010045 | -3.801581383 | 0.000143775 | 0.002574069 |
| CAMK2N2 | 358.1304614 | 1.37270404 | 0.415588021 | 3.30304044 | 0.000956426 | 0.009861404 |
| RP11-384P7.7 | 131.9611774 | 1.428840541 | 0.302366431 | 4.725526369 | 2.30E-06 | 0.000121771 |
| SPINT5P | 7.077740847 | -2.337812588 | 0.477095878 | -4.900089684 | 9.58E-07 | 6.29E-05 |
| HP | 918.3692871 | 1.446817467 | 0.3646123 | 3.968098355 | 7.24E-05 | 0.001557402 |
| RP11-64B16.2 | 38.59624223 | 1.430223186 | 0.288254619 | 4.961666145 | 6.99E-07 | 5.02E-05 |
| snoU13 | 12.92958458 | -1.301189481 | 0.31426909 | -4.14036735 | 3.47E-05 | 0.000917958 |
| CYR61 | 23720.17371 | 1.195938103 | 0.25805249 | 4.634476129 | 3.58E-06 | 0.000169964 |
| MMP13 | 491.1773552 | -1.878633858 | 0.397211172 | -4.72955947 | 2.25E-06 | 0.000120359 |
| GJB3 | 1021.225649 | -1.179336429 | 0.334956396 | -3.520865524 | 0.000430141 | 0.005524814 |
| RP11-96C23.5 | 199.0904344 | 1.117751233 | 0.298838834 | 3.740314525 | 0.00018379 | 0.003043268 |
| HLF | 774.9391585 | 1.50833173 | 0.266497947 | 5.659824961 | 1.52E-08 | 2.65E-06 |
| C10orf85 | 56.3502368 | -1.234190094 | 0.376498981 | -3.278070213 | 0.001045194 | 0.010515309 |
| CTC-296K1.3 | 16.82707881 | 1.336795357 | 0.450401883 | 2.968005701 | 0.002997388 | 0.022412873 |
| VILL | 3542.323738 | -1.044833351 | 0.261513315 | -3.995335203 | 6.46E-05 | 0.001433857 |
| MIR27B | 63.75069415 | 1.170277962 | 0.269121433 | 4.348512674 | 1.37E-05 | 0.00045273 |
| LINC00710 | 26.6692714 | 1.09742552 | 0.285915445 | 3.838286948 | 0.000123896 | 0.002329665 |
| IL20RB | 541.0858539 | -1.191887831 | 0.372456529 | -3.200072323 | 0.001373931 | 0.012707885 |
| RP11-863P13.5 | 30.48197601 | 1.890945717 | 0.392989803 | 4.811691558 | 1.50E-06 | 8.91E-05 |
| ECEL1P2 | 30.30235617 | -2.403026591 | 0.550716661 | -4.363453587 | 1.28E-05 | 0.000431117 |
| AC004603.4 | 7.539224458 | 1.499771507 | 0.557133463 | 2.691942966 | 0.007103709 | 0.041283031 |
| AK2P2 | 12.01846985 | 1.010288905 | 0.269909437 | 3.743066254 | 0.000181788 | 0.003025663 |
| MS4A10 | 3.750907401 | 3.554452374 | 1.28270522 | 2.771059413 | 0.005587423 | 0.034795519 |
| RP6-65G23.3 | 61.04251745 | -1.422341554 | 0.306471429 | -4.641024966 | 3.47E-06 | 0.000166285 |
| KLF15 | 2720.203833 | 1.622916249 | 0.392788147 | 4.131785195 | 3.60E-05 | 0.000941635 |
| TFF1 | 5321.136131 | -1.52877519 | 0.507702323 | -3.011164458 | 0.002602479 | 0.020155641 |
| GAPDHP55 | 61.78280837 | -1.1455241 | 0.320830512 | -3.57049613 | 0.000356306 | 0.00485342 |
| TRIM54 | 117.2926524 | -1.061588549 | 0.336246832 | -3.157170411 | 0.001593082 | 0.014164883 |
| VIT | 215.8037288 | 2.009677943 | 0.402848731 | 4.988666439 | 6.08E-07 | 4.54E-05 |
| RP11-589M4.4 | 103.3875988 | 1.011975534 | 0.198683287 | 5.09341048 | 3.52E-07 | 2.94E-05 |
| CPXM1 | 3927.380648 | 1.24304245 | 0.264294328 | 4.703250567 | 2.56E-06 | 0.000131206 |
| AC016735.2 | 341.1035011 | -1.736365363 | 0.475490369 | -3.651736138 | 0.000260473 | 0.003884192 |
| PYY2 | 89.39033057 | 1.328249503 | 0.378443405 | 3.509770511 | 0.000448494 | 0.005683785 |
| KCNN2 | 212.2208209 | 1.129366688 | 0.288717561 | 3.911666066 | 9.17E-05 | 0.00185989 |
| SRPX | 2080.495854 | 1.416526995 | 0.222840213 | 6.356693778 | 2.06E-10 | 8.12E-08 |
| IFITM9P | 47.47891064 | -1.354815132 | 0.396927054 | -3.41325974 | 0.000641907 | 0.00739255 |
| RP1-34B20.4 | 15.51132699 | -1.286817756 | 0.469851865 | -2.738773329 | 0.006166888 | 0.037306512 |
| FGF14-IT1 | 88.91855111 | 1.145087557 | 0.212304273 | 5.393615218 | 6.91E-08 | 8.23E-06 |
| PNLIP | 1322499.119 | 1.654324377 | 0.573858426 | 2.882809247 | 0.00394146 | 0.027186113 |
| SNORA1 | 3.978042282 | 1.787222066 | 0.518234301 | 3.448675746 | 0.000563343 | 0.006711748 |
| RP11-191N8.2 | 15.30417338 | -1.22150117 | 0.377018984 | -3.239893007 | 0.001195746 | 0.011550186 |
| F13A1 | 15770.55771 | 1.042297798 | 0.257309837 | 4.050749904 | 5.11E-05 | 0.001227399 |
| AC104809.4 | 47.31977556 | 1.165063791 | 0.446501632 | 2.609315861 | 0.009072346 | 0.049047016 |
| KLF14 | 21.69096993 | 1.14523398 | 0.337178934 | 3.396516998 | 0.000682493 | 0.007729835 |
| RP11-304C12.3 | 17.57550256 | 1.106673516 | 0.343388086 | 3.222806968 | 0.001269411 | 0.012055692 |
| TMPRSS3 | 1048.867554 | -1.033233293 | 0.286199179 | -3.610189578 | 0.000305973 | 0.004347899 |
| KLB | 962.31861 | 1.763645412 | 0.272840435 | 6.464017749 | 1.02E-10 | 4.76E-08 |
| HTR1B | 85.93995944 | -1.52014206 | 0.33817461 | -4.495139533 | 6.95E-06 | 0.000277777 |
| VSIG2 | 1250.907071 | -1.345107357 | 0.347821336 | -3.867236479 | 0.000110076 | 0.002140848 |
| ADAMTS19 | 25.13311248 | 1.588685768 | 0.33304696 | 4.770155437 | 1.84E-06 | 0.000102548 |
| ANXA8 | 633.4535146 | -1.14358274 | 0.387968119 | -2.947620398 | 0.0032023 | 0.023499427 |
| LARP1P1 | 16.05411564 | -1.228244473 | 0.286722883 | -4.283733695 | 1.84E-05 | 0.000567428 |
| SLC6A2 | 14.4420783 | 1.81246241 | 0.454248091 | 3.990027581 | 6.61E-05 | 0.001453854 |
| TIMP4 | 473.1542555 | 2.326614706 | 0.353764505 | 6.57673303 | 4.81E-11 | 2.72E-08 |
| FAM110D | 265.6074219 | 1.238688094 | 0.2509238 | 4.93651097 | 7.95E-07 | 5.50E-05 |
| TMEM252 | 33.56603535 | 1.680169084 | 0.346115705 | 4.854356681 | 1.21E-06 | 7.45E-05 |
| MIR4530 | 16.66315645 | 1.909837339 | 0.302562002 | 6.312218073 | 2.75E-10 | 1.03E-07 |
| RP5-894D12.3 | 453.0699033 | 1.987138254 | 0.435872802 | 4.558986583 | 5.14E-06 | 0.000225973 |
| ARNTL2 | 2727.872389 | -1.061029077 | 0.272077426 | -3.899732111 | 9.63E-05 | 0.001935663 |
| MT-TS1 | 27.83277976 | 1.344335516 | 0.250765684 | 5.360922961 | 8.28E-08 | 9.58E-06 |
| LINC00456 | 28.68691202 | -1.088447138 | 0.358780947 | -3.03373729 | 0.002415447 | 0.019109423 |
| CTB-25B13.6 | 3.697211663 | -1.610327036 | 0.527000141 | -3.055648208 | 0.002245746 | 0.018153577 |
| AC017060.1 | 274.8722821 | -1.269066147 | 0.392315529 | -3.234809875 | 0.001217238 | 0.011699001 |
| RP11-1100L3.8 | 95.41023318 | 2.011401392 | 0.316101527 | 6.363149881 | 1.98E-10 | 7.95E-08 |
| DHH | 76.02911737 | 1.020373826 | 0.265177928 | 3.847883695 | 0.000119143 | 0.002270755 |
| RP11-855A2.5 | 19.29151871 | 2.358650902 | 0.684224099 | 3.447190629 | 0.000566449 | 0.006742573 |
| RP11-560I19.2 | 7.887737871 | -1.948355619 | 0.674458255 | -2.888771252 | 0.003867503 | 0.026816017 |
| CORIN | 728.8429348 | -1.376636328 | 0.248633828 | -5.536802204 | 3.08E-08 | 4.57E-06 |
| OR5P4P | 7.229967097 | -1.81088922 | 0.671334125 | -2.697448486 | 0.006987309 | 0.040770632 |
| RASL10B | 389.0037524 | 1.023160448 | 0.231078932 | 4.42775306 | 9.52E-06 | 0.000348648 |
| SPTSSB | 503.5521562 | -1.426704452 | 0.335433698 | -4.253312826 | 2.11E-05 | 0.000628909 |
| AC079753.5 | 2.959364442 | -1.935763254 | 0.66278895 | -2.920632964 | 0.003493211 | 0.024994085 |
| APOA4 | 31.22940877 | 4.439899179 | 1.243504302 | 3.570473517 | 0.000356336 | 0.00485342 |
| MIR196A1 | 5.239737636 | -1.899005567 | 0.556019894 | -3.415355436 | 0.000636988 | 0.007346749 |
| RNU6-1245P | 2.523151036 | 1.499016161 | 0.408568261 | 3.668949117 | 0.00024355 | 0.003711299 |
| MIR329-2 | 4.298834226 | 1.418807011 | 0.376021809 | 3.773204051 | 0.000161164 | 0.002777066 |
| KRT17 | 6608.218381 | -1.428306561 | 0.406910929 | -3.510120916 | 0.000447903 | 0.005679992 |
| SCN4A | 216.8504874 | 2.474448849 | 0.394687679 | 6.269384576 | 3.62E-10 | 1.28E-07 |
| SLN | 26.24571983 | 2.026558158 | 0.619476208 | 3.27140596 | 0.001070142 | 0.010675593 |
| RP11-491F9.1 | 5.207290924 | 1.689979864 | 0.554774837 | 3.046244624 | 0.002317192 | 0.018537175 |
| RP11-400N13.2 | 65.21521626 | -2.451644084 | 0.529188311 | -4.632838698 | 3.61E-06 | 0.000170898 |
| SMTNL2 | 88.30193364 | 1.404315488 | 0.314709774 | 4.46225572 | 8.11E-06 | 0.000310663 |
| HPN | 3342.24615 | 2.07815225 | 0.4613345 | 4.504653887 | 6.65E-06 | 0.000269484 |
| MIR3676 | 3.678959027 | 1.623488753 | 0.495057762 | 3.279392585 | 0.001040308 | 0.01048238 |
| PRAMENP | 25.67912142 | 1.772225924 | 0.64312458 | 2.755649495 | 0.005857573 | 0.035992873 |
| MIR379 | 5.397354791 | 1.691150541 | 0.371705398 | 4.549706701 | 5.37E-06 | 0.000232516 |
| SYNE3 | 2120.362999 | 1.002078 | 0.193953071 | 5.166600338 | 2.38E-07 | 2.20E-05 |
| GJB2 | 2769.064367 | -1.25033514 | 0.332172755 | -3.76411106 | 0.000167143 | 0.002852132 |
| DKK1 | 1201.234483 | -1.397089158 | 0.397739074 | -3.512577089 | 0.000443783 | 0.00564611 |
| RP11-276H19.1 | 173.767276 | 1.00682104 | 0.223334696 | 4.508126394 | 6.54E-06 | 0.000266219 |
| MBOAT2 | 4340.181413 | -1.159932073 | 0.219409052 | -5.286619054 | 1.25E-07 | 1.31E-05 |
| RP11-44M6.3 | 28.64050276 | 1.151196015 | 0.316955387 | 3.632044319 | 0.000281185 | 0.004100524 |
| ASPA | 666.0746892 | 1.422637133 | 0.248844227 | 5.716978659 | 1.08E-08 | 1.99E-06 |
| SNORD114-4 | 11.71226318 | 1.193638559 | 0.273367286 | 4.366427946 | 1.26E-05 | 0.000426765 |
| MIR126 | 4.524461034 | 1.074476923 | 0.357464573 | 3.005827726 | 0.00264859 | 0.020427565 |
| RP11-554A11.4 | 64.5918706 | 1.493677342 | 0.299575036 | 4.985987358 | 6.16E-07 | 4.57E-05 |
| CTA-363E6.1 | 25.07955021 | -1.560223019 | 0.479583026 | -3.253290747 | 0.001140767 | 0.011205817 |
| RP11-611O2.2 | 15.24528947 | 1.870353405 | 0.450901105 | 4.148034642 | 3.35E-05 | 0.000896661 |
| PPP1R1A | 934.76127 | 2.476413987 | 0.333546087 | 7.424503199 | 1.13E-13 | 1.63E-10 |
| PRG4 | 1044.285377 | 1.122597875 | 0.317406947 | 3.536777896 | 0.00040504 | 0.005298403 |
| A3GALT2 | 6.723040586 | 1.035090993 | 0.38092443 | 2.717313232 | 0.006581429 | 0.039080679 |
| RP11-96C23.14 | 23.22849654 | 1.174623664 | 0.383628409 | 3.061878724 | 0.002199525 | 0.017858152 |
| SLC8A2 | 502.0504233 | 1.601352245 | 0.431441939 | 3.711628607 | 0.00020593 | 0.003295133 |
| ADIPOQ | 7398.066353 | 4.099421933 | 0.635005002 | 6.455731722 | 1.08E-10 | 4.94E-08 |
| RP11-249C24.10 | 46.30220094 | 1.574405985 | 0.359678375 | 4.377260616 | 1.20E-05 | 0.000413265 |
| ADH4 | 165.8519326 | 1.420750295 | 0.304184948 | 4.670679157 | 3.00E-06 | 0.000148372 |
| RP11-569G9.7 | 12.51296241 | -1.264309634 | 0.39335323 | -3.214183937 | 0.001308158 | 0.012294924 |
| PSAT1P3 | 17.7398491 | 1.905151681 | 0.490976755 | 3.880329696 | 0.000104315 | 0.002056503 |
| FAM65C | 726.3384933 | 1.351053615 | 0.203536593 | 6.637890487 | 3.18E-11 | 1.91E-08 |
| PAEP | 11.59626002 | -1.529576397 | 0.579215183 | -2.640774 | 0.008271688 | 0.045934427 |
| SOCS3 | 14452.25846 | 1.230461202 | 0.233418105 | 5.271489981 | 1.35E-07 | 1.40E-05 |
| AC073316.1 | 47.30029802 | 1.127433156 | 0.289818286 | 3.890138101 | 0.000100187 | 0.001997341 |
| FOXN4 | 17.68606389 | 2.523796102 | 0.529164757 | 4.769395669 | 1.85E-06 | 0.000102788 |
| KCNK1 | 1952.448145 | -1.181829793 | 0.24587377 | -4.806652579 | 1.53E-06 | 8.99E-05 |
| SLC19A3 | 983.9094666 | 1.394616376 | 0.261088193 | 5.341552833 | 9.22E-08 | 1.04E-05 |
| ATP1B3-AS1 | 150.6953747 | 1.098431607 | 0.263921339 | 4.161965873 | 3.16E-05 | 0.000857997 |
| MIR425 | 2.703851647 | 1.134879319 | 0.387827504 | 2.926247643 | 0.003430777 | 0.024669585 |
| RP11-572C15.6 | 12180.30334 | 1.057104368 | 0.263646964 | 4.009545012 | 6.08E-05 | 0.001381034 |
| FLYWCH1P1 | 15.57155586 | 1.322974301 | 0.365289849 | 3.6217111 | 0.000292661 | 0.004223653 |
| LHX6 | 373.2067274 | 1.207664451 | 0.221432454 | 5.453872862 | 4.93E-08 | 6.41E-06 |
| SYCP1 | 3.797362669 | 3.768697933 | 0.903033173 | 4.173377067 | 3.00E-05 | 0.000824742 |
| TLL1 | 1072.90147 | 1.300455382 | 0.236790446 | 5.4920095 | 3.97E-08 | 5.51E-06 |
| AC008592.5 | 9.124661008 | 1.405632005 | 0.463888498 | 3.030107475 | 0.002444667 | 0.019274121 |
| CERS4 | 1699.417531 | 1.141144652 | 0.239826681 | 4.758205589 | 1.95E-06 | 0.000107125 |
| EGLN3-AS1 | 3.067637748 | -2.380786449 | 0.710534075 | -3.350699895 | 0.000806076 | 0.008666373 |
| LHX9 | 43.94822945 | -1.764781958 | 0.582236533 | -3.031039546 | 0.002437133 | 0.019234165 |
| SGPP2 | 2955.596035 | -1.174559933 | 0.260388605 | -4.510796213 | 6.46E-06 | 0.000264546 |
| SOX17 | 778.8173972 | 1.923696654 | 0.26517253 | 7.254509555 | 4.03E-13 | 4.25E-10 |
| AOX3P | 24.83510382 | 1.639631011 | 0.407432821 | 4.024297817 | 5.71E-05 | 0.001327213 |
| KIF14 | 799.8471358 | -1.027028898 | 0.237820664 | -4.318501524 | 1.57E-05 | 0.000501294 |
| AC114812.10 | 21.00141805 | -2.181369088 | 0.462755364 | -4.713870997 | 2.43E-06 | 0.000127391 |
| RNY1P2 | 2.629205892 | 1.719124799 | 0.523751164 | 3.282331224 | 0.001029526 | 0.010397043 |
| RP11-703I16.3 | 9.231702374 | -1.162068966 | 0.37040937 | -3.137255857 | 0.001705372 | 0.014847286 |
| GREM2 | 257.7833277 | 1.086134929 | 0.308416635 | 3.521648338 | 0.000428873 | 0.005516622 |
| CCDC178 | 52.24680006 | 1.303733108 | 0.344594248 | 3.783386162 | 0.000154709 | 0.00270288 |
| SLIT3 | 11778.17067 | 1.760749732 | 0.289858379 | 6.074517278 | 1.24E-09 | 3.67E-07 |
| ADAMTS16 | 1613.23043 | 1.970818939 | 0.306686544 | 6.426166965 | 1.31E-10 | 5.73E-08 |
| RP11-503N18.3 | 9.041587795 | 1.304744554 | 0.498271465 | 2.618541592 | 0.008830653 | 0.048067172 |
| MYH11 | 22357.13414 | 1.621361792 | 0.296091376 | 5.475883201 | 4.35E-08 | 5.85E-06 |
| GDF7 | 317.3623212 | 1.384967501 | 0.277269291 | 4.99502667 | 5.88E-07 | 4.45E-05 |
| CHRD | 1183.676917 | 1.037003749 | 0.188485188 | 5.501778473 | 3.76E-08 | 5.36E-06 |
| RN7SL421P | 11.18205081 | 1.942124998 | 0.718160488 | 2.704304999 | 0.006844743 | 0.040228946 |
| TMEM92 | 568.2987936 | -1.229712765 | 0.306971341 | -4.005952998 | 6.18E-05 | 0.001394696 |
| AC018643.4 | 7.290262537 | 1.156639894 | 0.421481223 | 2.744226387 | 0.006065369 | 0.03683524 |
| GP2 | 377497.5502 | 1.661045381 | 0.553360897 | 3.001739715 | 0.002684416 | 0.02063043 |
| PRYP1 | 4.622549257 | -5.330716388 | 1.811792774 | -2.942232944 | 0.003258547 | 0.023804242 |
| MEP1A | 104.6148984 | 2.13269197 | 0.412030035 | 5.176059481 | 2.27E-07 | 2.12E-05 |
| SSPO | 414.0310343 | 1.132047877 | 0.197481091 | 5.732436805 | 9.90E-09 | 1.87E-06 |
| LINC00639 | 181.4939258 | 1.323167673 | 0.215199121 | 6.148573778 | 7.82E-10 | 2.48E-07 |
| RP11-323D18.5 | 3.848990838 | -1.963263991 | 0.662939527 | -2.961452607 | 0.003061916 | 0.022747079 |
| ESM1 | 755.8483516 | -1.534355627 | 0.274064459 | -5.598520999 | 2.16E-08 | 3.51E-06 |
| PAX9 | 127.7267324 | -1.16219506 | 0.368656815 | -3.152512074 | 0.001618721 | 0.014301783 |
| LINC00332 | 7.855274301 | 1.437842641 | 0.406873502 | 3.533881252 | 0.000409505 | 0.005334848 |
| DDC | 481.1054062 | 1.282454777 | 0.427831256 | 2.997571496 | 0.0027214 | 0.020828459 |
| CFD | 4224.100631 | 2.431493308 | 0.343949303 | 7.069336342 | 1.56E-12 | 1.41E-09 |
| RP11-757A13.1 | 12.63507602 | 1.317710602 | 0.410401451 | 3.210784465 | 0.001323732 | 0.012390524 |
| NPW | 77.5738721 | 1.187544936 | 0.374699296 | 3.169327908 | 0.001527919 | 0.013769634 |
| TRBV7-5 | 53.03062764 | 1.999697361 | 0.560162279 | 3.569853662 | 0.000357181 | 0.004859826 |
| SNORD114-5 | 4.552842061 | 1.143714456 | 0.326055747 | 3.50772672 | 0.000451953 | 0.005715014 |
| SH2D5 | 21.25377618 | -1.137113937 | 0.397117873 | -2.863416671 | 0.00419099 | 0.028387644 |
| AOC3 | 5978.555265 | 1.192384594 | 0.246792038 | 4.831535907 | 1.35E-06 | 8.22E-05 |
| CXorf30 | 11.08680593 | -1.931360903 | 0.619652413 | -3.116845608 | 0.001827973 | 0.015563711 |
| TRHDE-AS1 | 1008.161775 | 1.236341542 | 0.332894776 | 3.713910918 | 0.000204081 | 0.003275466 |
| MIR3194 | 18.0680642 | 1.51203449 | 0.275551062 | 5.487311419 | 4.08E-08 | 5.58E-06 |
| RN7SL512P | 7.228671514 | 1.531957078 | 0.297101884 | 5.156335789 | 2.52E-07 | 2.29E-05 |
| SBF1P1 | 40.17314242 | 1.046974788 | 0.332399403 | 3.1497493 | 0.001634106 | 0.01439394 |
| RP11-475N22.4 | 508.8285108 | 1.00685915 | 0.262013613 | 3.842774193 | 0.000121651 | 0.002303932 |
| RPS4XP5 | 21.28949667 | 1.136881885 | 0.34003112 | 3.343464225 | 0.000827394 | 0.008849208 |
| LINC00982 | 620.3456523 | 1.149468513 | 0.320434513 | 3.587218186 | 0.000334225 | 0.004634812 |
| SERPINB3 | 857.8415582 | -3.202207049 | 0.66486217 | -4.816347197 | 1.46E-06 | 8.74E-05 |
| PEX5L | 513.4351338 | 1.375574091 | 0.338600217 | 4.062531633 | 4.85E-05 | 0.001183093 |
| ANGPTL5 | 34.27307984 | 1.389764466 | 0.343201247 | 4.049415546 | 5.13E-05 | 0.001232139 |
| GLTPD2 | 151.4008798 | 1.06278038 | 0.237747576 | 4.470204901 | 7.81E-06 | 0.00030141 |
| ATP4A | 89.17807483 | 2.380330453 | 0.428606349 | 5.553651874 | 2.80E-08 | 4.34E-06 |
| PTPRS | 14391.95575 | 1.233581499 | 0.19697179 | 6.26273184 | 3.78E-10 | 1.32E-07 |
| NAT8L | 862.29781 | 1.77197411 | 0.31960895 | 5.544194279 | 2.95E-08 | 4.44E-06 |
| RP11-90K6.1 | 20.61409749 | 1.457130685 | 0.314668454 | 4.630685623 | 3.64E-06 | 0.000172058 |
| CALCA | 24.9335185 | 1.436946541 | 0.270777703 | 5.30673879 | 1.12E-07 | 1.22E-05 |
| LCN6 | 45.01304553 | 1.373370681 | 0.408812594 | 3.359413827 | 0.00078108 | 0.008498387 |
| USP2 | 538.8504894 | 1.166658342 | 0.228051326 | 5.115770926 | 3.12E-07 | 2.69E-05 |
| GSDMC | 215.1862309 | -2.08097312 | 0.375421237 | -5.54303517 | 2.97E-08 | 4.44E-06 |
| AQP12A | 1001.663305 | 2.310899426 | 0.662466876 | 3.488324489 | 0.000486058 | 0.006034328 |
| CD55 | 10500.72113 | -1.063569007 | 0.230954273 | -4.605106434 | 4.12E-06 | 0.000190473 |
| SFTPA2 | 77.41534579 | -1.389699579 | 0.533419966 | -2.605263522 | 0.009180362 | 0.049445887 |
| CLSTN2-AS1 | 31.21336139 | 1.004752529 | 0.246612 | 4.074224006 | 4.62E-05 | 0.001145243 |
| RP3-416H24.1 | 609.5245544 | -1.198516156 | 0.354483783 | -3.381018294 | 0.000722177 | 0.008020879 |
| SCARA5 | 2071.849552 | 2.468806374 | 0.431685655 | 5.718990998 | 1.07E-08 | 1.98E-06 |
| AP000647.3 | 3.393522403 | -2.85345002 | 0.850705639 | -3.354215478 | 0.000795904 | 0.008619622 |
| ZG16B | 438.0540055 | -1.262120821 | 0.362708837 | -3.479707939 | 0.000501961 | 0.006170856 |
| GLYAT | 45.21055606 | 3.477192226 | 0.493110143 | 7.051552833 | 1.77E-12 | 1.49E-09 |
| CBFA2T3 | 1421.838043 | 1.250452546 | 0.277232839 | 4.510477725 | 6.47E-06 | 0.000264621 |
| HUNK-AS1 | 3.568520453 | 1.687787682 | 0.596168797 | 2.831056725 | 0.004639449 | 0.030518779 |
| OAF | 6082.661806 | 1.208048949 | 0.188104402 | 6.422225836 | 1.34E-10 | 5.82E-08 |
| ECT2 | 2812.916528 | -1.109669785 | 0.212320001 | -5.226402513 | 1.73E-07 | 1.72E-05 |
| ZPLD1 | 86.57465544 | -1.110826816 | 0.363635298 | -3.054782694 | 0.002252236 | 0.018183429 |
| PTGFR | 646.6070663 | 1.019870477 | 0.253732949 | 4.019464092 | 5.83E-05 | 0.00134592 |
| AC004019.10 | 27.44711954 | 1.83505188 | 0.392970398 | 4.669694948 | 3.02E-06 | 0.000148707 |
| TMPRSS11E | 35.34789959 | -2.148408347 | 0.777408744 | -2.76355053 | 0.005717624 | 0.035362211 |
| TMPRSS4 | 4189.052201 | -1.754372522 | 0.400402798 | -4.381519134 | 1.18E-05 | 0.000407424 |
| SYT6 | 184.6047004 | 1.301115211 | 0.37685225 | 3.452587081 | 0.000555238 | 0.006637497 |
| RP11-138H8.7 | 13.84488006 | 1.441192261 | 0.346744007 | 4.1563581 | 3.23E-05 | 0.000873836 |
| CTD-2545H1.2 | 27.06103372 | 1.432562981 | 0.375100024 | 3.819149267 | 0.000133913 | 0.002458534 |
| AZGP1 | 5033.760159 | 1.532730101 | 0.398716685 | 3.844158418 | 0.000120967 | 0.002293195 |
| RNU6-1247P | 7.198870266 | 1.081935253 | 0.336339094 | 3.216798973 | 0.001296294 | 0.012220096 |
| RP11-227G15.2 | 8.335445348 | 1.092513343 | 0.362004176 | 3.017957842 | 0.002544843 | 0.019832611 |
| ANKRD1 | 524.5214487 | -2.006719616 | 0.395327889 | -5.076089169 | 3.85E-07 | 3.17E-05 |
| RP11-395L14.4 | 35.90460821 | 1.645505344 | 0.447061565 | 3.680713069 | 0.000232583 | 0.003593328 |
| RP11-667K14.4 | 84.48799309 | 1.066504888 | 0.21949569 | 4.858887606 | 1.18E-06 | 7.31E-05 |
| ADH1A | 93.75843636 | 1.196294725 | 0.209656934 | 5.705963081 | 1.16E-08 | 2.09E-06 |
| ABCC6 | 1512.912853 | 1.484208107 | 0.313469939 | 4.734770136 | 2.19E-06 | 0.000117631 |
| IGF2 | 19270.54419 | 1.886640347 | 0.30223782 | 6.242237815 | 4.31E-10 | 1.46E-07 |
| POC1B-GALNT4 | 1094.206358 | -1.514455078 | 0.523456282 | -2.8931835 | 0.003813584 | 0.02657867 |
| RPS3AP23 | 10.36077038 | -2.706143251 | 0.767220658 | -3.527203319 | 0.000419974 | 0.005429452 |
| MIR3612 | 3.922506105 | 1.1646298 | 0.361855747 | 3.218491923 | 0.001288666 | 0.012167441 |
| CSRNP1 | 6307.212397 | 1.38310597 | 0.219520726 | 6.300571218 | 2.97E-10 | 1.08E-07 |
| LINC00842 | 243.377434 | -1.19603225 | 0.297470905 | -4.020669682 | 5.80E-05 | 0.001341431 |
| SNORA58 | 3.589931298 | -1.456007493 | 0.389976641 | -3.733576164 | 0.00018878 | 0.00309566 |
| RP11-183M13.1 | 50.19448665 | 1.234956432 | 0.342829997 | 3.602241466 | 0.000315485 | 0.004444531 |
| AGR2 | 12487.02924 | -1.527579498 | 0.403425619 | -3.786520806 | 0.000152771 | 0.00267865 |
| KLHDC7B | 734.2285205 | -1.705404441 | 0.369518851 | -4.61520281 | 3.93E-06 | 0.000183543 |
| ENTHD1 | 57.54294511 | -1.689760196 | 0.395951837 | -4.267590242 | 1.98E-05 | 0.00060036 |
| CLCN1 | 40.61611571 | -1.238507508 | 0.413820755 | -2.992859817 | 0.002763767 | 0.021097328 |
| CTC-340A15.2 | 5.069633124 | 2.482603573 | 0.796647044 | 3.116315552 | 0.001831262 | 0.015578111 |
| MPZL2 | 3844.901316 | -1.156689718 | 0.227931086 | -5.074734383 | 3.88E-07 | 3.19E-05 |
| NPY5R | 78.47226577 | 1.278937631 | 0.345837077 | 3.698092874 | 0.000217225 | 0.00342438 |
| OR2B11 | 22.94601118 | 1.931703892 | 0.434156806 | 4.449323069 | 8.61E-06 | 0.000324493 |
| RP11-1L12.3 | 61.39618773 | -1.642723253 | 0.499767509 | -3.286974889 | 0.001012699 | 0.010273201 |
| KCNAB1-AS2 | 17.87379533 | 1.106437419 | 0.341038732 | 3.244316011 | 0.00117733 | 0.011448879 |
| PPEF1 | 98.76012673 | -1.359916932 | 0.263995331 | -5.151291598 | 2.59E-07 | 2.35E-05 |
| CCM2L | 411.3160262 | 1.042308241 | 0.169953078 | 6.132917719 | 8.63E-10 | 2.71E-07 |
| MUC5AC | 18399.79913 | -1.695781258 | 0.520699138 | -3.25673913 | 0.001127 | 0.011103423 |
| ADCY1 | 2039.88367 | 1.471214894 | 0.25133717 | 5.853550801 | 4.81E-09 | 1.02E-06 |
| RP4-575N6.4 | 67.46515616 | 1.158843056 | 0.279694409 | 4.143247128 | 3.42E-05 | 0.000909172 |
| CYP2E1 | 713.3444695 | 1.909810634 | 0.344457217 | 5.544405922 | 2.95E-08 | 4.44E-06 |
| RP11-650L12.2 | 52.77567764 | -1.030645663 | 0.263250189 | -3.915080423 | 9.04E-05 | 0.001843168 |
| RP11-329B9.1 | 10.01153789 | 1.544615046 | 0.47658445 | 3.241010163 | 0.001191069 | 0.011519283 |
| MRPL49P2 | 4.436316506 | -1.570703952 | 0.567879599 | -2.765910159 | 0.005676417 | 0.035201826 |
| ADAMTS1 | 17616.08194 | 1.786638654 | 0.265559582 | 6.727825986 | 1.72E-11 | 1.12E-08 |
| RP11-459D20.1 | 2.482216642 | 2.993269095 | 1.077199035 | 2.778752114 | 0.005456815 | 0.034166426 |
| TRBV6-7 | 63.52628647 | 1.738556162 | 0.517464804 | 3.359757317 | 0.00078011 | 0.008492567 |
| RNU1-123P | 6.112998269 | 1.078881663 | 0.36172033 | 2.982640377 | 0.002857736 | 0.021617343 |
| RP11-146F11.1 | 98.28734803 | 1.094301808 | 0.29234052 | 3.743243692 | 0.00018166 | 0.003025663 |
| CGB7 | 123.9718223 | -1.774655754 | 0.357502708 | -4.964034437 | 6.90E-07 | 4.97E-05 |
| PDE2A | 1012.55292 | 1.452043814 | 0.204786285 | 7.090532526 | 1.34E-12 | 1.24E-09 |
| RP11-667K14.3 | 64.70022265 | 1.798807174 | 0.279635697 | 6.432680769 | 1.25E-10 | 5.56E-08 |
| CTA-445C9.15 | 1517.160076 | 1.189383089 | 0.321976709 | 3.694003491 | 0.000220751 | 0.003464293 |
| RP5-1112F19.2 | 100.1375908 | -1.643077685 | 0.443568604 | -3.704224488 | 0.000212038 | 0.00337212 |
| HRASLS2 | 153.3688974 | -1.439747305 | 0.450340674 | -3.197018143 | 0.001388562 | 0.012804317 |
| RP11-63L7.5 | 11.66828165 | 1.238640816 | 0.41662106 | 2.973063379 | 0.002948436 | 0.022140236 |
| PROK2 | 26.05627087 | 1.215962286 | 0.388979569 | 3.126031242 | 0.001771827 | 0.015228654 |
| TRIM58 | 73.0522238 | 1.271243984 | 0.285279323 | 4.456137838 | 8.34E-06 | 0.000316548 |
| C1QTNF9B | 23.58723618 | 1.03530775 | 0.353959191 | 2.924935346 | 0.003445278 | 0.024728256 |
| RBP7 | 531.5425641 | 1.830147116 | 0.292391429 | 6.25923653 | 3.87E-10 | 1.34E-07 |
| TRBV11-1 | 52.16509232 | 1.797701848 | 0.52888283 | 3.39905504 | 0.000676191 | 0.007671827 |
| MMP19 | 3651.119354 | 1.578092365 | 0.284288459 | 5.551025076 | 2.84E-08 | 4.36E-06 |
| CTD-2562J15.6 | 9.625561655 | 1.077765984 | 0.363107711 | 2.96817157 | 0.002995771 | 0.022410261 |
| GPT2 | 3665.881961 | 1.479223617 | 0.337867 | 4.378123986 | 1.20E-05 | 0.000411995 |
| ANKRD20A17P | 11.2809098 | 1.552465591 | 0.553399036 | 2.805327601 | 0.005026546 | 0.032323577 |
| IGFBP2 | 14817.54904 | 1.579273184 | 0.362853318 | 4.352373546 | 1.35E-05 | 0.000447318 |
| TRBV5-2 | 15.55565566 | 1.938381288 | 0.743647126 | 2.606587479 | 0.009144946 | 0.049302803 |
| RP11-626H12.3 | 85.83305021 | -1.001071542 | 0.33165733 | -3.018391127 | 0.002541207 | 0.019818873 |
| CTB-60B18.12 | 30.73867785 | -1.948975272 | 0.420236449 | -4.637806355 | 3.52E-06 | 0.000168068 |
| Y_RNA | 8.085248275 | 1.152903609 | 0.352008936 | 3.275211199 | 0.00105583 | 0.010581366 |
| RP11-863P13.6 | 13.37404992 | 1.900339838 | 0.462607011 | 4.107892431 | 3.99E-05 | 0.001021541 |
| GHR | 2584.578737 | 2.163987811 | 0.300842473 | 7.193092736 | 6.33E-13 | 6.33E-10 |
| RNU4-56P | 6.042612906 | 1.39725238 | 0.392458378 | 3.560256215 | 0.000370493 | 0.004985293 |
| MYH7 | 165.6493805 | 1.76904952 | 0.550970036 | 3.210790793 | 0.001323703 | 0.012390524 |
| GRASP | 1579.623209 | 1.077607839 | 0.207341172 | 5.197268961 | 2.02E-07 | 1.97E-05 |
| KLK5 | 26.4481713 | -2.123070758 | 0.763277648 | -2.781518317 | 0.005410528 | 0.033909278 |
| DPYS | 95.07981133 | 1.758229808 | 0.349552801 | 5.029940543 | 4.91E-07 | 3.82E-05 |
| CLCP1 | 10.8013167 | 1.540363514 | 0.403015028 | 3.82209944 | 0.00013232 | 0.002439636 |
| RP11-93M12.1 | 40.86765197 | -1.270421676 | 0.30197465 | -4.207047429 | 2.59E-05 | 0.000735359 |
| AC010090.1 | 3.890951383 | 1.505909979 | 0.531653151 | 2.83250457 | 0.004618491 | 0.030423597 |
| SPRR1A | 69.70102099 | -2.099704671 | 0.786411727 | -2.669981385 | 0.007585545 | 0.04323273 |
| ACSM5 | 446.2247518 | 2.037736538 | 0.334276126 | 6.095967904 | 1.09E-09 | 3.29E-07 |
| LINC00989 | 77.32168162 | 1.620781503 | 0.314813893 | 5.148379841 | 2.63E-07 | 2.36E-05 |
| SCN11A | 107.568318 | 1.238056876 | 0.226831467 | 5.458047288 | 4.81E-08 | 6.28E-06 |
| ITGB6 | 10114.78258 | -1.561155939 | 0.330466719 | -4.724094284 | 2.31E-06 | 0.000122465 |
| STYK1 | 573.2249627 | -1.325629667 | 0.277187861 | -4.782423236 | 1.73E-06 | 9.79E-05 |
| EGR1 | 39906.92316 | 1.307552632 | 0.253164516 | 5.164833736 | 2.41E-07 | 2.21E-05 |
| PLS1 | 4637.11274 | -1.050324328 | 0.302032145 | -3.477524983 | 0.000506066 | 0.006205673 |
| ELANE | 17.05188894 | 1.078254108 | 0.374496637 | 2.879209053 | 0.00398674 | 0.027399931 |
| FAHD2P1 | 39.26364657 | -1.542582335 | 0.579949326 | -2.659857104 | 0.007817381 | 0.044192342 |
| RP11-366M4.3 | 75.28849285 | -1.420547395 | 0.546122208 | -2.601152953 | 0.009291102 | 0.049883866 |
| RP11-20I20.1 | 60.71470635 | 1.006135813 | 0.379025778 | 2.654531359 | 0.007941866 | 0.044662973 |
| RP11-284F21.7 | 139.4095726 | -1.160736997 | 0.311437599 | -3.727029104 | 0.00019375 | 0.003149348 |
| RP11-66B24.4 | 1316.589991 | 1.137136702 | 0.192937809 | 5.893799177 | 3.77E-09 | 8.80E-07 |
| CTRB1 | 763399.8163 | 2.047800898 | 0.633096623 | 3.234578772 | 0.001218223 | 0.011699001 |
| TSPAN1 | 4419.912339 | -1.536852568 | 0.35658873 | -4.3098742 | 1.63E-05 | 0.000517444 |
| RP11-776H12.1 | 49.36135014 | -1.944050788 | 0.459878988 | -4.227309438 | 2.37E-05 | 0.000684663 |
| AC098592.8 | 21.54218195 | -1.448183542 | 0.379535534 | -3.81567314 | 0.000135812 | 0.00248386 |
| RP11-203J24.9 | 143.3295417 | 1.047162402 | 0.317720468 | 3.295860691 | 0.000981207 | 0.010022757 |
| ANO2 | 320.0906743 | 1.177266665 | 0.220666543 | 5.335048293 | 9.55E-08 | 1.07E-05 |
| IL1A | 161.7793336 | -1.550353754 | 0.324285264 | -4.780833198 | 1.75E-06 | 9.82E-05 |
| KLK10 | 1802.187849 | -1.390361442 | 0.42222781 | -3.292917729 | 0.000991535 | 0.010108471 |
| PNPLA2 | 7913.804622 | 1.092524496 | 0.190865919 | 5.724041792 | 1.04E-08 | 1.93E-06 |
| ARHGDIG | 495.1323578 | 2.146024572 | 0.508488236 | 4.220401617 | 2.44E-05 | 0.000699617 |
| RN7SKP54 | 23.01401606 | -1.843916562 | 0.61531697 | -2.996693819 | 0.002729247 | 0.020876222 |
| PRED60 | 7.160007465 | 1.124290156 | 0.288061724 | 3.902948779 | 9.50E-05 | 0.001912079 |
| ABHD17C | 2321.101661 | -1.305394559 | 0.24642988 | -5.297225144 | 1.18E-07 | 1.27E-05 |
| PYGM | 256.019798 | 1.041377503 | 0.247576775 | 4.206281065 | 2.60E-05 | 0.000737319 |
| CTD-2265D6.2 | 105.7790377 | 1.677672595 | 0.262440673 | 6.392578462 | 1.63E-10 | 6.99E-08 |
| RP11-96H17.3 | 14.71502244 | -1.453135732 | 0.486368206 | -2.987727639 | 0.002810599 | 0.021376889 |
| CDCA2 | 465.6632434 | -1.119249374 | 0.233956537 | -4.784005554 | 1.72E-06 | 9.74E-05 |
| RP11-344A5.1 | 11.72038733 | 1.426109468 | 0.414822093 | 3.437882151 | 0.000586283 | 0.006938421 |
| ABCB5 | 201.5196079 | 1.288927654 | 0.363125471 | 3.549537993 | 0.000385908 | 0.005135998 |
| TRBVA | 18.55693134 | 1.396350746 | 0.532357391 | 2.622957378 | 0.008717018 | 0.047654578 |
| MIR369 | 3.001412692 | 1.263783457 | 0.380445468 | 3.32185178 | 0.000894222 | 0.009381682 |
| CTRL | 17590.94495 | 1.568147388 | 0.569238267 | 2.754817235 | 0.005872493 | 0.036051301 |
| IFNE | 157.5324551 | -1.907115575 | 0.466640297 | -4.086907172 | 4.37E-05 | 0.00109689 |
| AL035610.2 | 114.6516115 | 1.413215354 | 0.247795129 | 5.703160337 | 1.18E-08 | 2.11E-06 |
| C1orf110 | 127.8381921 | -2.641530064 | 0.545717452 | -4.840472036 | 1.30E-06 | 7.92E-05 |
| HSD3BP4 | 79.50059657 | 1.123042054 | 0.280367472 | 4.005607528 | 6.19E-05 | 0.001395928 |
| RP5-1011O1.3 | 11.73798569 | -1.510685889 | 0.429205323 | -3.519727754 | 0.00043199 | 0.005539444 |
| CYMP | 5.658392995 | -2.832683019 | 0.908357203 | -3.118468166 | 0.001817938 | 0.015512141 |
| IQCJ-SCHIP1-AS1 | 82.91024534 | 1.188061085 | 0.240331514 | 4.943426131 | 7.68E-07 | 5.35E-05 |
| MLXIPL | 1313.771704 | 1.184704404 | 0.286302857 | 4.137941262 | 3.50E-05 | 0.000923952 |
| ANKRD34B | 11.376341 | -1.144508745 | 0.405996884 | -2.819008692 | 0.004817221 | 0.0314284 |
| MLK7-AS1 | 341.3673722 | -1.40906999 | 0.245384586 | -5.742292192 | 9.34E-09 | 1.78E-06 |
| DNAJC27-AS1 | 138.2672879 | 1.005199638 | 0.189061301 | 5.31679214 | 1.06E-07 | 1.16E-05 |
| ECHDC3 | 670.2310509 | 1.430035862 | 0.272287509 | 5.251933403 | 1.51E-07 | 1.54E-05 |
| AL359955.1 | 24.42824745 | -1.460466916 | 0.372535727 | -3.920340547 | 8.84E-05 | 0.001812876 |
| MT1X | 7924.31574 | 1.025886578 | 0.299429235 | 3.426140328 | 0.000612224 | 0.007154233 |
| RP11-15J10.3 | 18.27479306 | -1.446893946 | 0.376880228 | -3.839134655 | 0.000123469 | 0.002324801 |
| RNU1-59P | 5.042124059 | 1.208379282 | 0.297747418 | 4.058403897 | 4.94E-05 | 0.001198951 |
| RP11-615I2.3 | 32.84439921 | -1.411375027 | 0.371046202 | -3.803771663 | 0.00014251 | 0.002563266 |
| NGFR | 3340.281765 | 1.492718588 | 0.266520235 | 5.600770183 | 2.13E-08 | 3.50E-06 |
| NT5C1A | 70.66236519 | 1.869336656 | 0.368938011 | 5.066804186 | 4.05E-07 | 3.30E-05 |
| TCN1 | 3211.445928 | -1.611917826 | 0.428232716 | -3.764116484 | 0.000167139 | 0.002852132 |
| CTNNA3 | 211.195896 | 1.422869404 | 0.292040771 | 4.872160135 | 1.10E-06 | 6.94E-05 |
| C10orf62 | 56.95838721 | 1.027551756 | 0.376921946 | 2.7261659 | 0.006407478 | 0.038326632 |
| SORBS1 | 9917.842103 | 1.186193227 | 0.241403231 | 4.913742142 | 8.94E-07 | 5.95E-05 |
| RP11-1029J19.5 | 12.72168887 | -1.334550446 | 0.454530825 | -2.93610548 | 0.003323613 | 0.024143868 |
| AC019117.2 | 457.8686499 | -1.281698724 | 0.346472629 | -3.699278432 | 0.000216213 | 0.003414839 |
| GJB4 | 428.3127744 | -1.24550407 | 0.380169895 | -3.276177534 | 0.001052224 | 0.010558794 |
| SNORA4 | 3.858766816 | -1.199267879 | 0.341306763 | -3.513753632 | 0.000441822 | 0.005630779 |
| RPL31P47 | 6.794746553 | 2.05579145 | 0.640441575 | 3.209959396 | 0.001327537 | 0.012408273 |
| TRBV5-3 | 28.43191214 | 2.309761625 | 0.575090145 | 4.016347078 | 5.91E-05 | 0.001357984 |
| AL096864.1 | 8.752173838 | -1.325568431 | 0.479949077 | -2.761893905 | 0.005746715 | 0.035502717 |
| AC096633.1 | 20.74521488 | 1.122970489 | 0.361025628 | 3.110500756 | 0.001867704 | 0.015798694 |
| YBX2 | 58.40520923 | 1.443279685 | 0.289331214 | 4.988330376 | 6.09E-07 | 4.54E-05 |
| RP11-6C14.1 | 8.275438807 | 1.723541125 | 0.61283351 | 2.812413318 | 0.004917127 | 0.031861646 |
| TFAP2A-AS1 | 28.34516683 | -1.251397246 | 0.321389783 | -3.893705751 | 9.87E-05 | 0.001975248 |
| SLC2A4 | 220.1420827 | 2.17737352 | 0.287163033 | 7.582360091 | 3.39E-14 | 6.16E-11 |
| RP1-18D14.7 | 121.608976 | 1.197657678 | 0.202774199 | 5.906361272 | 3.50E-09 | 8.27E-07 |
| LPAR3 | 313.6322714 | 1.214361432 | 0.402381063 | 3.017938822 | 0.002545003 | 0.019832611 |
| SLC2A1-AS1 | 122.1201918 | -1.07494394 | 0.242328861 | -4.435889055 | 9.17E-06 | 0.000339238 |
| HBA2 | 4599.066155 | 3.06221295 | 0.380585538 | 8.046057037 | 8.55E-16 | 2.37E-12 |
| AQP7P3 | 112.7535655 | 1.299042337 | 0.327790001 | 3.96303223 | 7.40E-05 | 0.001584721 |
| RP11-576D8.4 | 8.555074003 | -1.706337571 | 0.470251351 | -3.628564952 | 0.000285001 | 0.0041394 |
| RP11-436I9.5 | 212.2611498 | 1.12956897 | 0.30977283 | 3.646443013 | 0.000265895 | 0.003935221 |
| RP11-279F6.3 | 19.2818956 | 1.087280715 | 0.370439099 | 2.935113271 | 0.003334259 | 0.024189665 |
| RP11-366L20.3 | 27.09611974 | -1.489756083 | 0.461266404 | -3.229708626 | 0.001239164 | 0.011837711 |
| AC004460.1 | 2.86007897 | 1.562826617 | 0.479824381 | 3.257080463 | 0.001125645 | 0.011092882 |
| RP11-350N15.3 | 13.11063377 | 1.255197171 | 0.302592211 | 4.148147652 | 3.35E-05 | 0.000896661 |
| OR56B2P | 24.46740919 | 1.803361187 | 0.484087435 | 3.72527989 | 0.000195099 | 0.003162041 |
| U3 | 8.181326178 | 1.128100628 | 0.301801416 | 3.737890439 | 0.000185571 | 0.003060157 |
| TRBV4-2 | 49.24761623 | 1.419778772 | 0.401489448 | 3.53627917 | 0.000405806 | 0.005305363 |
| RAX2 | 8.256633549 | 1.311933983 | 0.470341075 | 2.789324713 | 0.005281808 | 0.03344659 |
| RP11-856M7.6 | 10.24248967 | 1.644958795 | 0.603290696 | 2.726643732 | 0.006398208 | 0.03830183 |
| RP11-206P5.2 | 86.65152514 | 1.418386431 | 0.462972402 | 3.063652225 | 0.002186529 | 0.017798604 |
| RN7SL815P | 65.8081521 | -1.18169714 | 0.364399941 | -3.242857659 | 0.001183373 | 0.011480536 |
| CYP2A6 | 268.6761537 | 2.910632967 | 0.419158035 | 6.943998983 | 3.81E-12 | 2.91E-09 |
| XX-FW81066F1.2 | 10.62466129 | 1.471379657 | 0.364467905 | 4.037062347 | 5.41E-05 | 0.001278372 |
| ATP5G1P4 | 12.06563321 | -1.252934536 | 0.395507804 | -3.167913566 | 0.001535372 | 0.013814448 |
| CACNG6 | 55.7214017 | -3.027779775 | 0.716926416 | -4.223278298 | 2.41E-05 | 0.000691893 |
| CSF3 | 64.70870867 | 2.679202393 | 0.500110516 | 5.357220673 | 8.45E-08 | 9.75E-06 |
| CTD-2193G5.1 | 8.355074474 | -1.189948138 | 0.393904747 | -3.020903269 | 0.002520219 | 0.019698522 |
| MIR493 | 5.603386453 | 1.752298334 | 0.380843022 | 4.601103955 | 4.20E-06 | 0.000193254 |
| C10orf71 | 85.62284794 | 1.440282034 | 0.502864817 | 2.864153515 | 0.004181253 | 0.028335586 |
| PLA2G10 | 157.6228624 | -1.182752627 | 0.311647126 | -3.795166157 | 0.000147545 | 0.002617464 |
| SAA2 | 780.2585968 | 1.295687905 | 0.378205347 | 3.425884685 | 0.0006128 | 0.007155224 |
| RP11-494M8.4 | 36.78135106 | -1.561617715 | 0.447430061 | -3.490193999 | 0.00048267 | 0.006005653 |
| PSMD10P2 | 42.97984879 | -1.480510889 | 0.366039049 | -4.044680184 | 5.24E-05 | 0.00124978 |
| CELA3A | 375280.0933 | 1.731130353 | 0.607336344 | 2.850365156 | 0.004366906 | 0.029294256 |
| PDK4 | 35623.34935 | 1.015483034 | 0.321222972 | 3.161302654 | 0.001570652 | 0.014015464 |
| PALMD | 3113.40007 | 1.344582704 | 0.224254184 | 5.995797629 | 2.02E-09 | 5.37E-07 |
| MIR656 | 2.500556654 | 1.232206657 | 0.42200413 | 2.919892411 | 0.003501522 | 0.025018636 |
| F11 | 2242.76206 | 1.470183208 | 0.500837823 | 2.935447641 | 0.003330668 | 0.024172605 |
| COL11A1 | 19657.27215 | -1.243971863 | 0.354336101 | -3.510711609 | 0.000446909 | 0.005672921 |
| RP11-734K21.5 | 12.14576834 | -1.72878708 | 0.590993973 | -2.925219473 | 0.003442134 | 0.024719337 |
| ACADS | 1546.028251 | 1.200427134 | 0.21827973 | 5.49948974 | 3.81E-08 | 5.40E-06 |
| RP13-192B19.2 | 173.3284253 | -1.169419404 | 0.275230643 | -4.248870657 | 2.15E-05 | 0.000635634 |
| AC013410.1 | 56.36767145 | -1.948225158 | 0.590872912 | -3.297198293 | 0.000976545 | 0.009989608 |
| RP11-619A14.2 | 17.63455975 | 1.715057957 | 0.463431485 | 3.700779971 | 0.000214938 | 0.003405729 |
| AP000439.1 | 4.292012689 | 1.654858632 | 0.617089996 | 2.681713595 | 0.007324614 | 0.042108701 |
| MIR4757 | 2.330670143 | 2.85278805 | 0.874681758 | 3.26151543 | 0.001108184 | 0.010962363 |
| RP11-415D17.1 | 25.6945348 | 1.482614758 | 0.276219671 | 5.367520537 | 7.98E-08 | 9.32E-06 |
| ARHGEF15 | 1125.196673 | 1.210956578 | 0.18505686 | 6.543700029 | 6.00E-11 | 3.21E-08 |
| FFAR3 | 49.60721861 | 1.293603337 | 0.310301575 | 4.16885843 | 3.06E-05 | 0.000838308 |
| TRBV7-1 | 12.37822462 | 1.591821865 | 0.524567268 | 3.034542874 | 0.002409005 | 0.019085566 |
| RP6-149D17.1 | 83.8159713 | 4.07898683 | 1.545745899 | 2.638846936 | 0.008318853 | 0.046098292 |
| AKR1C2 | 1369.321034 | 1.308539561 | 0.29101693 | 4.496437923 | 6.91E-06 | 0.000276654 |
| RP11-1110J8.1 | 34.59025655 | 1.631976297 | 0.301119707 | 5.419692759 | 5.97E-08 | 7.46E-06 |
| FA2H | 1228.949435 | -1.035525955 | 0.333619512 | -3.103913038 | 0.001909795 | 0.016051072 |
| HSPB2-C11orf52 | 7.394621133 | 2.360188708 | 0.657558874 | 3.589319224 | 0.000331543 | 0.004604169 |
| PLA2R1 | 4512.560911 | -1.115736866 | 0.218979457 | -5.095166831 | 3.48E-07 | 2.92E-05 |
| TNS4 | 2694.879294 | -1.396694 | 0.402420892 | -3.47072935 | 0.000519047 | 0.006322398 |
| KRT27 | 7.601949019 | 1.371299454 | 0.512453173 | 2.675950754 | 0.007451759 | 0.042600991 |
| RP11-321G12.1 | 61.72573295 | 1.726840809 | 0.588646347 | 2.933579421 | 0.003350779 | 0.024259865 |
| snR65 | 2.599165446 | 1.390128337 | 0.493519027 | 2.816767461 | 0.004850963 | 0.031600955 |
| TMC5 | 7608.399959 | -1.308968052 | 0.3431669 | -3.814377349 | 0.000136527 | 0.002491277 |
| PI16 | 2136.618004 | 3.034147199 | 0.453157713 | 6.69556561 | 2.15E-11 | 1.33E-08 |
| ITGA8 | 1542.311846 | 1.059491066 | 0.267905743 | 3.954715769 | 7.66E-05 | 0.001627461 |
| RPL9P28 | 48.5982496 | -2.816764707 | 0.579008916 | -4.864803684 | 1.15E-06 | 7.14E-05 |
| IGF1 | 12890.95741 | 1.428593056 | 0.316589371 | 4.512447948 | 6.41E-06 | 0.00026277 |
| LINC00707 | 111.1448505 | -1.007551802 | 0.380661484 | -2.646844624 | 0.008124667 | 0.045360873 |
| IFNWP19 | 52.92913952 | -1.806078723 | 0.516323243 | -3.497961299 | 0.000468829 | 0.005880345 |
| DGAT2 | 1491.443836 | 1.981787876 | 0.324547238 | 6.106315632 | 1.02E-09 | 3.13E-07 |
| CTC-232P5.4 | 37.76617689 | 1.281276178 | 0.246724435 | 5.193146675 | 2.07E-07 | 2.01E-05 |
| ATF3 | 3757.173347 | 1.150146045 | 0.215234114 | 5.343697719 | 9.11E-08 | 1.03E-05 |
| RN7SL857P | 4.903862671 | 1.099031999 | 0.417403582 | 2.633020047 | 0.008462935 | 0.046675328 |
| SLITRK6 | 495.7828601 | -1.069265944 | 0.339326728 | -3.151139762 | 0.001626346 | 0.014341787 |
| KRT223P | 6.620573916 | 2.420881367 | 0.77189956 | 3.136264731 | 0.001711147 | 0.014890909 |
| ANXA8L2 | 297.0454845 | -1.240961304 | 0.475431162 | -2.610180827 | 0.009049437 | 0.048989562 |
| RP1-28O10.1 | 24.33674264 | 1.20204125 | 0.388726425 | 3.092255047 | 0.001986421 | 0.016523141 |
| OSR1 | 1235.309061 | 2.005681252 | 0.33215294 | 6.038426918 | 1.56E-09 | 4.30E-07 |
| CD34 | 4547.275659 | 1.040208449 | 0.196772204 | 5.286358682 | 1.25E-07 | 1.31E-05 |
| CACNA1S | 8.914206149 | 1.460492106 | 0.538556864 | 2.711862391 | 0.006690636 | 0.039596503 |
| RN7SL76P | 8.273778098 | 1.334126659 | 0.39096353 | 3.412406926 | 0.000643919 | 0.007404785 |
| RP3-523K23.2 | 480.8347065 | -1.205528847 | 0.359483873 | -3.353499109 | 0.000797967 | 0.008621976 |
| SNRPGP12 | 3.975455455 | 1.151231003 | 0.431953535 | 2.665173245 | 0.007694867 | 0.043664339 |
| SPDEF | 544.2646251 | -1.551433904 | 0.450231755 | -3.445856239 | 0.000569253 | 0.006763845 |
| RP13-1016M1.2 | 19.57803446 | 1.007203078 | 0.245209559 | 4.107519631 | 4.00E-05 | 0.001021541 |
| FABP6 | 46.31125907 | -2.211758367 | 0.635500981 | -3.480338239 | 0.000500781 | 0.00616024 |
| AC093323.1 | 7.007136196 | -1.150046383 | 0.380735827 | -3.020588823 | 0.002522837 | 0.019715036 |
| TRBV7-6 | 80.84654202 | 1.480169886 | 0.466841585 | 3.170604192 | 0.001521223 | 0.0137315 |
| NDUFAF4P2 | 5.292902052 | -1.76910555 | 0.623915196 | -2.835490401 | 0.004575539 | 0.030236086 |
| RPS20P21 | 6.023065441 | 1.121938186 | 0.244891922 | 4.581360533 | 4.62E-06 | 0.000207536 |
| CTA-363E6.2 | 104.4469124 | -1.610771427 | 0.397249578 | -4.054809661 | 5.02E-05 | 0.001213759 |
| ALDH1L1 | 2121.68301 | 1.373069195 | 0.321816053 | 4.266627416 | 1.98E-05 | 0.00060171 |
| MYRIP | 966.4624287 | 1.249385656 | 0.238289351 | 5.243145142 | 1.58E-07 | 1.61E-05 |
| RP11-449O16.2 | 10.40950549 | 1.585374831 | 0.50906382 | 3.114294845 | 0.001843851 | 0.015651063 |
| OFCC1 | 26.8251153 | -2.62648876 | 0.755485309 | -3.476558349 | 0.000507894 | 0.006223936 |
| AC119403.1 | 2.882179479 | 2.185409969 | 0.795309988 | 2.747871903 | 0.005998344 | 0.036547722 |
| RP11-1094H24.4 | 69.38533049 | -1.386756189 | 0.318366334 | -4.355850602 | 1.33E-05 | 0.000443296 |
| MMP1 | 3205.274424 | -1.391889286 | 0.407849383 | -3.412753196 | 0.000643102 | 0.007401932 |
| EPS8L3 | 1510.400272 | -1.223119337 | 0.382369607 | -3.198788069 | 0.001380066 | 0.012752358 |
| FASN | 15758.63277 | 1.019020162 | 0.224319257 | 4.542722612 | 5.55E-06 | 0.000237965 |
| ABCA8 | 6201.531469 | 1.445474846 | 0.243272712 | 5.941787865 | 2.82E-09 | 7.00E-07 |
| HOXB6 | 882.3202721 | -1.439898072 | 0.264980218 | -5.433983264 | 5.51E-08 | 7.08E-06 |
| RNU4-1 | 12.8540539 | 1.415673405 | 0.276129102 | 5.126853327 | 2.95E-07 | 2.56E-05 |
| ANPEP | 33994.55767 | 1.697148445 | 0.404697026 | 4.193627178 | 2.75E-05 | 0.000770163 |
| FGFR1 | 9415.54404 | 1.048568741 | 0.223403698 | 4.693605116 | 2.68E-06 | 0.000136531 |
| APOA5 | 11.7449688 | 1.558844015 | 0.575556236 | 2.708413039 | 0.006760583 | 0.039889381 |
| MIR494 | 3.286493338 | 1.106835737 | 0.381280461 | 2.902943765 | 0.00369673 | 0.026024789 |
| SOX11 | 291.9133039 | -1.651647996 | 0.357697075 | -4.617448987 | 3.88E-06 | 0.00018208 |
| AP000695.4 | 72.60565774 | -1.450297378 | 0.263956643 | -5.494453026 | 3.92E-08 | 5.48E-06 |
| HAS1 | 651.9488761 | 2.723810051 | 0.37516152 | 7.260366293 | 3.86E-13 | 4.18E-10 |
| PVRL4 | 1600.239398 | -1.180179092 | 0.354429902 | -3.329795498 | 0.000869098 | 0.009201866 |
| HMGN2P47 | 13.91909461 | 1.679256384 | 0.552002745 | 3.042116004 | 0.002349213 | 0.01872217 |
| ADRB1 | 148.6305635 | 1.304071543 | 0.253225604 | 5.149840774 | 2.61E-07 | 2.35E-05 |
| C5orf46 | 217.5360613 | -1.287110624 | 0.384693315 | -3.345809697 | 0.000820427 | 0.008784334 |
| SLC7A9 | 78.11076454 | 1.154286652 | 0.323112263 | 3.572401255 | 0.000353723 | 0.00482625 |
| RP11-408H20.1 | 37.42951515 | -1.352817022 | 0.28388134 | -4.765431295 | 1.88E-06 | 0.000104255 |
| UGT3A1 | 8.705908952 | 2.511797295 | 0.850611222 | 2.952932233 | 0.003147711 | 0.023216375 |
| RP11-496I2.2 | 28.9536401 | -1.258504318 | 0.334917391 | -3.757655924 | 0.000171512 | 0.002910413 |
| TRBV26 | 21.41981829 | 1.245843353 | 0.474072483 | 2.627959641 | 0.008589871 | 0.047104768 |
| HEPACAM | 196.4716868 | 3.603583411 | 0.571537063 | 6.305073884 | 2.88E-10 | 1.07E-07 |
| GBP7 | 8.737193401 | -1.284299664 | 0.375016567 | -3.424647804 | 0.000615597 | 0.007172766 |
| MIR370 | 6.969934992 | 1.282350948 | 0.363621513 | 3.526609126 | 0.000420918 | 0.005436795 |
| SULT1B1 | 2757.770251 | -1.165415655 | 0.392846853 | -2.966590278 | 0.003011219 | 0.022481812 |
| RP4-694A7.2 | 13.9813281 | -1.311289485 | 0.491964018 | -2.665417465 | 0.00768928 | 0.04364534 |
| WFDC3 | 70.19945683 | -1.628169769 | 0.295043573 | -5.518404455 | 3.42E-08 | 4.96E-06 |
| CTD-3096M3.2 | 10.61794856 | 1.321310793 | 0.36088058 | 3.661351886 | 0.000250888 | 0.003789068 |
| AC131097.4 | 166.8765698 | 1.171830005 | 0.379926252 | 3.084361764 | 0.002039894 | 0.016892262 |
| RPL39P29 | 8.148407929 | 1.235363831 | 0.300495597 | 4.111087967 | 3.94E-05 | 0.001010941 |
| RP11-368J22.2 | 74.97980751 | 1.257716084 | 0.25746675 | 4.884965078 | 1.03E-06 | 6.65E-05 |
| AC016912.3 | 6.119347437 | 2.325299574 | 0.735813165 | 3.160176637 | 0.001576735 | 0.014060093 |
| TRBV6-8 | 78.21103582 | 1.826391607 | 0.495707589 | 3.684413246 | 0.00022923 | 0.003555619 |
| KRT16 | 1305.959567 | -1.57081811 | 0.478190973 | -3.28491795 | 0.001020121 | 0.010327001 |
| TRBV6-9 | 74.51031964 | 2.10357571 | 0.496438225 | 4.237336299 | 2.26E-05 | 0.000658713 |
| SLC24A2 | 279.3390662 | -1.300131678 | 0.327496881 | -3.969905529 | 7.19E-05 | 0.001551624 |
| RSPO3 | 990.7869415 | 1.665382317 | 0.291872887 | 5.705847966 | 1.16E-08 | 2.09E-06 |
| IZUMO1 | 66.14108475 | 1.166308477 | 0.328069041 | 3.555070216 | 0.000377878 | 0.005056829 |
| AC005550.4 | 23.0979046 | 1.462058434 | 0.46453718 | 3.147344276 | 0.001647608 | 0.014466001 |
| MIR125B1 | 10.53550472 | 1.660083034 | 0.34518731 | 4.809223814 | 1.52E-06 | 8.97E-05 |
| C10orf82 | 28.97989171 | 1.312190758 | 0.383834644 | 3.41863555 | 0.00062936 | 0.007280296 |
| RP11-690D19.1 | 18.34564538 | -2.091006791 | 0.551879308 | -3.788884198 | 0.000151325 | 0.002662233 |
| CTD-2385L22.1 | 36.6194897 | -1.479186649 | 0.488683798 | -3.026878844 | 0.00247093 | 0.01941756 |
| TAL1 | 445.4502187 | 1.074126035 | 0.178217466 | 6.027052568 | 1.67E-09 | 4.59E-07 |
| HIST1H2APS3 | 10.43420488 | -1.269636549 | 0.340631036 | -3.727307304 | 0.000193536 | 0.003148499 |
| VWF | 21582.06215 | 1.359670566 | 0.214467928 | 6.339738438 | 2.30E-10 | 8.89E-08 |
| RP11-344F13.1 | 8.850350196 | 1.267956708 | 0.435096242 | 2.914198254 | 0.003566034 | 0.0253477 |
| RP4-575N6.5 | 17.68076332 | 1.238560758 | 0.316934722 | 3.907936461 | 9.31E-05 | 0.001881782 |
| RP11-15J10.9 | 7.914571324 | -1.769390869 | 0.404942409 | -4.36948769 | 1.25E-05 | 0.000422661 |
| ID4 | 2776.171732 | 1.15682705 | 0.244978085 | 4.72216546 | 2.33E-06 | 0.000123131 |
| CTB-50L17.14 | 462.1260746 | 1.766844816 | 0.388751556 | 4.544920243 | 5.50E-06 | 0.00023643 |
| RP11-1090M7.1 | 18.88246926 | 1.57162254 | 0.391607026 | 4.013264407 | 5.99E-05 | 0.001368018 |
| SLC16A10 | 2930.858202 | 1.381637488 | 0.30329675 | 4.555398267 | 5.23E-06 | 0.000228577 |
| AC064836.3 | 22.71929238 | 1.05030101 | 0.274970581 | 3.819685016 | 0.000133622 | 0.002454356 |
| CEA | 361.2813293 | -2.064940303 | 0.666631301 | -3.097574777 | 0.001951112 | 0.016299351 |
| CGB5 | 93.34388289 | -2.017431027 | 0.583110466 | -3.459775023 | 0.000540627 | 0.006518783 |
| CALML6 | 37.33957822 | 1.034606662 | 0.267954804 | 3.861123768 | 0.000112867 | 0.002183153 |
| PRAP1 | 343.7380154 | 1.271446942 | 0.398371787 | 3.191608902 | 0.001414828 | 0.012972303 |
| RP11-44F21.5 | 39.73041302 | -1.496777604 | 0.461953364 | -3.240105432 | 0.001194855 | 0.011544444 |
| RP11-350G8.7 | 69.78795946 | 1.095000822 | 0.203467727 | 5.381692908 | 7.38E-08 | 8.67E-06 |
| PCK1 | 588.1780225 | 2.55516562 | 0.381350427 | 6.700308796 | 2.08E-11 | 1.31E-08 |
| C8orf46 | 97.4107324 | 1.058055428 | 0.261221802 | 4.050410115 | 5.11E-05 | 0.001228425 |
| SARDH | 1689.589326 | 1.596795871 | 0.316906785 | 5.038692592 | 4.69E-07 | 3.69E-05 |
| RP11-1070N10.3 | 38.35320129 | 1.226410634 | 0.285414581 | 4.296944564 | 1.73E-05 | 0.000541082 |
| RP11-443B9.1 | 2.304162129 | 3.419831995 | 1.069712517 | 3.196963616 | 0.001388824 | 0.012804317 |
| AC055876.2 | 4.444735974 | 1.114695575 | 0.32209509 | 3.460765503 | 0.000538642 | 0.006504789 |
| RP11-536O18.1 | 123.317991 | 2.45594375 | 0.459130031 | 5.349124609 | 8.84E-08 | 1.01E-05 |
| HTR2A | 211.6170451 | 1.323595281 | 0.322241332 | 4.107465894 | 4.00E-05 | 0.001021541 |
| CIDEA | 321.2382977 | 4.258574372 | 0.650424095 | 6.547381012 | 5.86E-11 | 3.21E-08 |
| PXMP2 | 853.9833311 | 1.031028771 | 0.228817722 | 4.505895615 | 6.61E-06 | 0.000268471 |
| ITGA2 | 12594.52572 | -1.421123051 | 0.274343169 | -5.180092718 | 2.22E-07 | 2.10E-05 |
| SLC25A34 | 299.1840286 | 1.121168245 | 0.211284495 | 5.306438805 | 1.12E-07 | 1.22E-05 |
| AGAP11 | 96.42620819 | 1.30135424 | 0.370407815 | 3.513301254 | 0.000442575 | 0.00563626 |
| EBF2 | 1747.469451 | 1.650609532 | 0.301164306 | 5.480760827 | 4.24E-08 | 5.74E-06 |
| CDH5 | 5000.378019 | 1.023344022 | 0.169317262 | 6.043943846 | 1.50E-09 | 4.19E-07 |
| C1QTNF9 | 45.24391909 | 2.72424405 | 0.453450222 | 6.007812811 | 1.88E-09 | 5.06E-07 |
| APOB | 1973.868204 | 2.551665704 | 0.498019437 | 5.123626737 | 3.00E-07 | 2.60E-05 |
| CTD-2538A21.1 | 4.785029331 | -1.426103534 | 0.483237108 | -2.951146572 | 0.003165966 | 0.02332571 |
| MAT1A | 884.9406726 | 1.594263032 | 0.482590526 | 3.303552281 | 0.000954682 | 0.009846023 |
| CTD-2526A2.2 | 29.98104085 | 1.135455318 | 0.264200633 | 4.297700983 | 1.73E-05 | 0.000539672 |
| SLC4A1 | 22.81184503 | 1.863256562 | 0.434447029 | 4.288800332 | 1.80E-05 | 0.000556844 |
| AC027119.1 | 18.86398365 | -2.075443036 | 0.63720717 | -3.257093034 | 0.001125595 | 0.011092882 |
| CTC-218H9.1 | 12.82909415 | 1.005550362 | 0.296610156 | 3.390141376 | 0.000698566 | 0.007839015 |
| RP11-309N17.4 | 18.88774643 | 1.361433529 | 0.301025603 | 4.52265028 | 6.11E-06 | 0.000253884 |
| TATDN2P3 | 8.610422752 | 1.997895209 | 0.605784788 | 3.298028025 | 0.000973664 | 0.009973216 |
| MSLN | 3687.900331 | -1.167603691 | 0.421440653 | -2.770505604 | 0.005596934 | 0.034831346 |
| MYEOV | 930.1149313 | -1.234534305 | 0.323894043 | -3.811537543 | 0.000138105 | 0.002509509 |
| RN7SL672P | 13.04339109 | 1.053958314 | 0.333178425 | 3.163345026 | 0.001559674 | 0.013960399 |
| DCLK1 | 2951.762295 | 1.181581771 | 0.246437485 | 4.794651152 | 1.63E-06 | 9.39E-05 |
| AC002075.4 | 6.960831495 | -2.583096774 | 0.699826196 | -3.691054704 | 0.000223326 | 0.003487626 |
| ONECUT3 | 980.9651119 | -1.666193647 | 0.502531632 | -3.31559954 | 0.000914468 | 0.009538259 |
| RP11-451F20.1 | 3.630322889 | -2.112721736 | 0.742571896 | -2.845140985 | 0.004439177 | 0.029616032 |
| RP11-384P7.6 | 27.8826897 | 2.092568752 | 0.756491331 | 2.766150337 | 0.005672238 | 0.035187546 |
| CDCA7 | 945.7260386 | -1.013024476 | 0.236882824 | -4.276479228 | 1.90E-05 | 0.000583065 |
| SLC10A6 | 182.3153017 | 1.213616082 | 0.30841664 | 3.934988995 | 8.32E-05 | 0.001733122 |
| AC051649.6 | 18.91900008 | 1.012016571 | 0.389222951 | 2.600094798 | 0.009319801 | 0.049996649 |
| RP5-1029K10.2 | 20.17254404 | 1.01272588 | 0.328988456 | 3.078302177 | 0.002081837 | 0.017134072 |
| LINC00314 | 12.69330404 | 1.452625628 | 0.430087044 | 3.377515429 | 0.000731438 | 0.008091474 |
| MIR889 | 3.527980875 | 1.141393394 | 0.364794404 | 3.128867605 | 0.001754814 | 0.015145404 |
| MIR5008 | 8.834004646 | 1.218390699 | 0.354435529 | 3.437552385 | 0.000586997 | 0.006942661 |
| CPHL1P | 88.63334315 | -2.281766184 | 0.481265708 | -4.741177575 | 2.12E-06 | 0.000115058 |
| DUOXA1 | 148.0356314 | -1.27894726 | 0.322189547 | -3.969549198 | 7.20E-05 | 0.001552359 |
| ENPEP | 2205.421675 | 1.041647275 | 0.210153259 | 4.956607764 | 7.17E-07 | 5.10E-05 |
| FBLN2 | 11541.60862 | 1.0568107 | 0.242240096 | 4.362658038 | 1.28E-05 | 0.000431571 |
| NAV2-AS4 | 45.41734546 | 1.413802565 | 0.276545317 | 5.1123721 | 3.18E-07 | 2.73E-05 |
| UBE2C | 571.33724 | -1.056739281 | 0.252425338 | -4.186343923 | 2.83E-05 | 0.000786234 |
| EPHA8 | 62.39628053 | 2.078638692 | 0.485561827 | 4.280893962 | 1.86E-05 | 0.000574263 |
| SLPI | 3904.32157 | -1.364717396 | 0.302688741 | -4.508649346 | 6.52E-06 | 0.000265928 |
| GPRC5A | 22552.99648 | -1.246965315 | 0.321052595 | -3.883990767 | 0.000102756 | 0.002033407 |
| FOXD3 | 55.75864579 | 1.005800435 | 0.354116584 | 2.840308756 | 0.004506989 | 0.02994038 |
| RP11-763B22.6 | 2.488216282 | 2.047069075 | 0.679804915 | 3.011259597 | 0.002601663 | 0.020153329 |
| RNF223 | 71.7974644 | -1.093997886 | 0.381689738 | -2.866196751 | 0.00415436 | 0.028208124 |
| SI | 72.44357081 | 2.762979643 | 0.95283946 | 2.899732599 | 0.003734811 | 0.026184393 |
| NOTUM | 74.76790034 | -1.106709596 | 0.303150234 | -3.650696818 | 0.00026153 | 0.003894743 |
| KANK3 | 635.8508386 | 1.325390875 | 0.205067197 | 6.463202774 | 1.03E-10 | 4.76E-08 |
| MIR132 | 5.881581358 | 2.438852112 | 0.382106138 | 6.382656201 | 1.74E-10 | 7.38E-08 |
| RP11-475O23.2 | 20.34262595 | 2.28873503 | 0.352078113 | 6.500645581 | 8.00E-11 | 4.00E-08 |
| AQP7 | 529.1228642 | 2.783026329 | 0.326119756 | 8.533755704 | 1.42E-17 | 1.10E-13 |
| GFRA2 | 239.9302463 | 1.313233392 | 0.277172622 | 4.737962141 | 2.16E-06 | 0.000116358 |
| TRBV5-6 | 90.02886216 | 1.487742834 | 0.406262203 | 3.662026204 | 0.000250228 | 0.003782035 |
| RPS10P28 | 4.004848501 | 2.012128525 | 0.721791503 | 2.787686632 | 0.005308587 | 0.033523617 |
| CHRNA2 | 24.77909007 | 1.6222101 | 0.389412725 | 4.165786053 | 3.10E-05 | 0.000847272 |
| RP1-167F1.2 | 46.53742443 | 1.380959794 | 0.264874297 | 5.21364212 | 1.85E-07 | 1.83E-05 |
| CST4 | 88.27503632 | -1.931078945 | 0.665962962 | -2.899679192 | 0.003735448 | 0.026184393 |
| RPL34P26 | 10.92498792 | -1.306260816 | 0.419150928 | -3.116445002 | 0.001830458 | 0.01557807 |
| OR52N5 | 28.85286858 | 1.943392644 | 0.466349189 | 4.16724783 | 3.08E-05 | 0.000843067 |
| CADM2 | 207.3096677 | 1.434792881 | 0.355598522 | 4.034867396 | 5.46E-05 | 0.001284933 |
| POSTN | 36860.28566 | -1.086095623 | 0.246367809 | -4.408431563 | 1.04E-05 | 0.00037264 |
| RP11-626H12.1 | 97.2297722 | -1.133718751 | 0.339420742 | -3.340157544 | 0.000837309 | 0.008933203 |
| MIR337 | 3.865566484 | 1.395636708 | 0.398036315 | 3.506304963 | 0.000454374 | 0.005739658 |
| CRNDE | 281.1221337 | -1.391487831 | 0.270309539 | -5.147757042 | 2.64E-07 | 2.36E-05 |
| LY6D | 585.5237974 | -1.710754355 | 0.543462339 | -3.147880235 | 0.001644591 | 0.014462634 |
| GPHA2 | 586.1148726 | 1.875134771 | 0.521077114 | 3.598574412 | 0.000319966 | 0.004486547 |
| PEBP4 | 60.88591231 | 1.515995309 | 0.394241012 | 3.845351608 | 0.00012038 | 0.002286511 |
| RP11-680G10.1 | 32.49248906 | 1.181557992 | 0.218899387 | 5.397721789 | 6.75E-08 | 8.07E-06 |
| ZBTB16 | 7117.054117 | 1.125121251 | 0.350465255 | 3.210364611 | 0.001325667 | 0.012402684 |
| RP1-302G2.5 | 73.95575882 | 1.540485538 | 0.435059983 | 3.540857809 | 0.000398828 | 0.005243849 |
| DBH | 184.3840558 | 1.394986572 | 0.290969938 | 4.794263557 | 1.63E-06 | 9.39E-05 |
| LIPH | 3982.408805 | -1.163691671 | 0.311258388 | -3.738667669 | 0.000184998 | 0.003058076 |
| CXXC11 | 25.94965253 | 1.325279388 | 0.407377976 | 3.253193509 | 0.001141158 | 0.011205817 |
| AQPEP | 222.2393277 | 1.818702789 | 0.394020874 | 4.615752382 | 3.92E-06 | 0.000183353 |
| PPAPDC1A | 227.6130117 | -1.093267991 | 0.312202289 | -3.501793647 | 0.000462137 | 0.005818872 |
| TIMD4 | 239.3277939 | 1.40771978 | 0.392683926 | 3.58486734 | 0.00033725 | 0.004658536 |
| NIPA2P2 | 7.195848432 | -3.682716601 | 0.962625282 | -3.825701101 | 0.0001304 | 0.002414513 |
| TRBV7-8 | 109.003685 | 1.303540906 | 0.355186632 | 3.6700168 | 0.000242535 | 0.003701618 |
| CHDC2 | 19.5455299 | -1.636165759 | 0.564532084 | -2.898268858 | 0.003752288 | 0.026267905 |
| CES1P1 | 247.3526196 | 2.062394618 | 0.556663876 | 3.704919082 | 0.000211458 | 0.003364716 |
| C3 | 224771.4605 | 1.05748948 | 0.243405854 | 4.344552376 | 1.40E-05 | 0.00045964 |
| PTPRR | 616.3699415 | -1.462218369 | 0.390732672 | -3.742247506 | 0.000182382 | 0.003028079 |
| GAD1 | 220.1446732 | -1.065817804 | 0.241320087 | -4.416614527 | 1.00E-05 | 0.000363038 |
| RASD1 | 3557.678279 | 1.49078421 | 0.281929582 | 5.287789248 | 1.24E-07 | 1.31E-05 |
| ADAMTS18 | 230.1108939 | 1.80001728 | 0.34068862 | 5.283467584 | 1.27E-07 | 1.33E-05 |
| PTGER3 | 2215.678946 | 1.122363293 | 0.232280771 | 4.831925128 | 1.35E-06 | 8.21E-05 |
| RP11-286B14.1 | 12.59853571 | 3.837056618 | 1.237067045 | 3.101736993 | 0.001923888 | 0.016140709 |
| MIR3143 | 2.706151486 | -1.194298656 | 0.37468567 | -3.187468197 | 0.001435242 | 0.013125172 |
| CTD-2062F14.3 | 14.33661656 | 1.270123008 | 0.408152027 | 3.111887049 | 0.001858956 | 0.015755269 |
| TEX26-AS1 | 230.1606327 | 1.899456391 | 0.351243853 | 5.407799667 | 6.38E-08 | 7.81E-06 |
| UGT2B4 | 47.94453373 | 1.978676822 | 0.718783611 | 2.752812935 | 0.005908565 | 0.036182418 |
| FXYD3 | 3662.679044 | -1.416802186 | 0.367207652 | -3.858313356 | 0.000114172 | 0.00219859 |
| ART1 | 9.95746854 | 1.11160327 | 0.355491681 | 3.126945945 | 0.001766324 | 0.015201191 |
| RP5-965F6.2 | 29.57942783 | 1.074613681 | 0.287010456 | 3.744162132 | 0.000180997 | 0.003018815 |
| MYOCD | 499.8696027 | 1.8078693 | 0.302755698 | 5.971379938 | 2.35E-09 | 6.08E-07 |
| PTF1A | 955.8200212 | 1.940438531 | 0.687398033 | 2.822874721 | 0.004759517 | 0.031150948 |
| RP11-37O16.8 | 4.706745628 | -1.17728052 | 0.312193852 | -3.770992002 | 0.0001626 | 0.002791004 |
| FGFBP2 | 39.07047909 | 1.097711734 | 0.293665968 | 3.737960314 | 0.000185519 | 0.003060157 |
| RP11-57A1.1 | 55.12409768 | -1.289769865 | 0.321583553 | -4.010683541 | 6.05E-05 | 0.001377411 |
| SDPR | 3855.695083 | 1.554282758 | 0.251487016 | 6.180369803 | 6.40E-10 | 2.10E-07 |
| GRIA1 | 54.3649242 | 1.311178632 | 0.280406061 | 4.675999615 | 2.93E-06 | 0.000145498 |
| SLC43A1 | 3646.099505 | 1.323459637 | 0.310229799 | 4.266062252 | 1.99E-05 | 0.000601876 |
| RP11-354M1.3 | 6.40139608 | -1.860346258 | 0.431809669 | -4.308255216 | 1.65E-05 | 0.000520822 |
| RP11-1008C21.1 | 44.15589501 | -1.225181217 | 0.29610005 | -4.13772715 | 3.51E-05 | 0.00092419 |
| MMP11 | 6007.454234 | -1.074026559 | 0.309346529 | -3.471920511 | 0.000516749 | 0.006301013 |
| CBLN4 | 438.9713169 | 1.321893573 | 0.343390965 | 3.849529272 | 0.000118345 | 0.00226108 |
| ABCB4 | 565.1695648 | 1.069984946 | 0.321837484 | 3.324612573 | 0.000885415 | 0.009316446 |
| RP5-894D12.5 | 72.56278147 | 2.09358899 | 0.547799683 | 3.821814901 | 0.000132473 | 0.002441299 |
| RP11-497G19.1 | 10.14915944 | 1.613245105 | 0.472336619 | 3.415456349 | 0.000636752 | 0.0073462 |
| ANLN | 2697.981581 | -1.030955701 | 0.271872577 | -3.792054759 | 0.000149406 | 0.002641505 |
| SLC1A2 | 2417.964199 | 1.129039803 | 0.376096388 | 3.001995867 | 0.002682158 | 0.020625273 |
| FABP4 | 7727.423796 | 3.676084115 | 0.452441918 | 8.124985699 | 4.47E-16 | 1.59E-12 |
| ANKRD18B | 59.7866966 | -1.653172414 | 0.407858037 | -4.053303507 | 5.05E-05 | 0.001219259 |
| MIRLET7C | 3.682919054 | 1.606729654 | 0.40219725 | 3.994879764 | 6.47E-05 | 0.001434925 |
| AC114812.5 | 23.19267129 | -2.505462957 | 0.530096345 | -4.726429412 | 2.29E-06 | 0.000121396 |
| NPM1P11 | 43.18733037 | 1.129074173 | 0.38975086 | 2.896912592 | 0.003768547 | 0.026354825 |
| RPL3L | 49.49398485 | 2.467242335 | 0.720327293 | 3.425168475 | 0.000614418 | 0.007166988 |
| RP5-884C9.2 | 6.853034055 | 1.005571593 | 0.373941972 | 2.689111332 | 0.007164252 | 0.041506681 |
| HHATL-AS1 | 3.790531645 | 2.048740761 | 0.659166036 | 3.108079983 | 0.001883071 | 0.015873401 |
| RPTN | 45.72404933 | -2.968117174 | 0.739015294 | -4.016313596 | 5.91E-05 | 0.001357984 |
| CTB-49A3.2 | 4.163742821 | -1.265431185 | 0.454249219 | -2.785764139 | 0.005340171 | 0.033646789 |
| RPSAP63 | 3.997308709 | 1.665143339 | 0.562203898 | 2.961813935 | 0.003058325 | 0.022729064 |
| RHBDL2 | 594.7139927 | -1.068682583 | 0.217310089 | -4.917777128 | 8.75E-07 | 5.85E-05 |
| PRSS1 | 1111269.711 | 1.568395667 | 0.556778453 | 2.81691157 | 0.004848787 | 0.031592058 |
| U91328.2 | 15.9094265 | -1.11055544 | 0.315954957 | -3.514916973 | 0.000439891 | 0.005613079 |
| RP11-211G23.2 | 56.41870909 | -1.621791336 | 0.44373136 | -3.654894562 | 0.000257288 | 0.003854373 |
| EEF1A1P31 | 11.54938891 | 1.693101465 | 0.441935783 | 3.83110291 | 0.00012757 | 0.002373395 |
| PRSS33 | 49.24960718 | -2.139436027 | 0.525789097 | -4.069000363 | 4.72E-05 | 0.001159575 |
| CCL20 | 789.0647612 | -1.411537312 | 0.377148635 | -3.742655228 | 0.000182086 | 0.003027139 |
| MIR411 | 4.146827625 | 1.480289195 | 0.373869313 | 3.959376029 | 7.51E-05 | 0.001603015 |
| CACNG4 | 410.5917931 | -1.093150777 | 0.317161137 | -3.446673158 | 0.000567535 | 0.006753438 |
| C19orf33 | 3067.743502 | -1.707250664 | 0.355138381 | -4.807282888 | 1.53E-06 | 8.99E-05 |
| OTOP3 | 25.99394453 | 2.322188424 | 0.642022606 | 3.616988564 | 0.00029805 | 0.004279263 |
| KCNIP3 | 180.5762309 | 1.015647472 | 0.238564697 | 4.257325095 | 2.07E-05 | 0.000620583 |
| TENM1 | 798.5958391 | 1.483658939 | 0.313877438 | 4.726873488 | 2.28E-06 | 0.000121396 |
| RP11-461M2.2 | 38.37284813 | -2.019735137 | 0.55919204 | -3.611881059 | 0.000303984 | 0.004334134 |
| RP11-1252I4.2 | 15.72354332 | 1.053141787 | 0.338447295 | 3.111686225 | 0.001860221 | 0.015762564 |
| CNN2P8 | 2.658203428 | 2.062421388 | 0.734589537 | 2.807583395 | 0.004991475 | 0.032156382 |
| WNT7B | 849.200913 | -1.312830011 | 0.395363832 | -3.320561729 | 0.000898365 | 0.009409546 |
| TNNI2 | 474.0630474 | -2.24222456 | 0.363890596 | -6.161809586 | 7.19E-10 | 2.34E-07 |
| RP1-34M23.5 | 3.706290803 | 1.285409408 | 0.385244087 | 3.336610346 | 0.000848068 | 0.009018379 |
| TRHDE | 1748.732651 | 1.181161848 | 0.367791403 | 3.211499336 | 0.001320443 | 0.012371616 |
| OR5P1P | 6.80754165 | -1.784315918 | 0.672420537 | -2.653571418 | 0.007964491 | 0.044738597 |
| AL121657.4 | 3.141056587 | -2.560841435 | 0.736212653 | -3.478399108 | 0.000504418 | 0.006193303 |
| AMY2B | 147007.1927 | 1.632464619 | 0.553156545 | 2.951180158 | 0.003165622 | 0.02332571 |
| CA9 | 1098.040233 | -2.480937592 | 0.456893791 | -5.430009422 | 5.64E-08 | 7.13E-06 |
| RP11-435B5.5 | 90.83096686 | 1.416134635 | 0.339573481 | 4.170333419 | 3.04E-05 | 0.000833714 |
| TRBV6-6 | 79.90118954 | 1.522363389 | 0.408261921 | 3.728889002 | 0.000192326 | 0.003134035 |
| RP11-37O16.4 | 60.51026842 | -1.144975653 | 0.259725427 | -4.408408009 | 1.04E-05 | 0.00037264 |
| BMX | 303.5020839 | 1.121590678 | 0.235670567 | 4.759146177 | 1.94E-06 | 0.000106927 |
| RP11-460N11.2 | 1913.042404 | -1.736353175 | 0.356226489 | -4.874295513 | 1.09E-06 | 6.92E-05 |
| FAM71A | 22.40998806 | 2.070407058 | 0.507512709 | 4.079517659 | 4.51E-05 | 0.001123764 |
| AC110771.1 | 31.39025424 | 1.242047617 | 0.229819558 | 5.404446985 | 6.50E-08 | 7.88E-06 |
| OR13H1 | 8.960726114 | -3.498129396 | 1.225538584 | -2.854360884 | 0.004312351 | 0.029008805 |
| RN7SL229P | 6.287090918 | 1.048466801 | 0.400956363 | 2.614914983 | 0.008924967 | 0.048431545 |
| CXCL12 | 11553.38469 | 1.358861485 | 0.226710093 | 5.993828815 | 2.05E-09 | 5.40E-07 |
| MIR320A | 4.479241801 | 1.077462942 | 0.280911184 | 3.835600015 | 0.000125258 | 0.002348289 |
| CD163L1 | 1884.727357 | 1.032157038 | 0.229816609 | 4.491220376 | 7.08E-06 | 0.00028121 |
| PLAC9 | 685.3763237 | 1.162355305 | 0.21661596 | 5.365972599 | 8.05E-08 | 9.37E-06 |
| AP000695.6 | 76.84460513 | -1.187472534 | 0.270911037 | -4.383256384 | 1.17E-05 | 0.000404546 |
| MT1M | 698.8313813 | 1.409419227 | 0.285631091 | 4.9344041 | 8.04E-07 | 5.53E-05 |
| ANKFN1 | 200.8105054 | -1.056133629 | 0.315858326 | -3.343694128 | 0.000826708 | 0.008844305 |
| CASC8 | 37.08296005 | -1.036840969 | 0.249872928 | -4.149473001 | 3.33E-05 | 0.000892497 |
| CELA3B | 243744.291 | 2.203058736 | 0.647779662 | 3.400938411 | 0.00067155 | 0.007631067 |
| PCDHB9 | 461.3087039 | -1.039597512 | 0.224057804 | -4.639862995 | 3.49E-06 | 0.000166608 |
| TRBV8-1 | 18.62175915 | 1.972204361 | 0.668656122 | 2.949504678 | 0.003182838 | 0.023395806 |
| HRASLS5 | 369.4820975 | 1.441844488 | 0.325056575 | 4.435672432 | 9.18E-06 | 0.000339258 |
| GABRP | 2857.852843 | -1.533481283 | 0.441483552 | -3.473473196 | 0.000513769 | 0.006271962 |
| LINC00840 | 23.39168807 | 1.116938585 | 0.350700098 | 3.184882445 | 0.001448128 | 0.013215483 |
| SELE | 780.7726169 | 1.581506676 | 0.349462743 | 4.525537292 | 6.02E-06 | 0.000251473 |
| EPHX2 | 2464.63133 | 1.517537279 | 0.273733837 | 5.543842495 | 2.96E-08 | 4.44E-06 |
| RP13-279N23.2 | 9.86526947 | 1.990587053 | 0.677374165 | 2.938681687 | 0.003296114 | 0.023993 |
| S100A2 | 434.1491805 | -2.180772299 | 0.408093561 | -5.34380473 | 9.10E-08 | 1.03E-05 |
| RP11-400N13.3 | 106.3877264 | -1.518865265 | 0.415955888 | -3.651505625 | 0.000260707 | 0.003886194 |
| LEMD1 | 192.1284303 | -1.882054589 | 0.412064217 | -4.567381761 | 4.94E-06 | 0.000218343 |
| RP11-396J6.1 | 8.334839396 | -2.673004829 | 0.668062146 | -4.001131999 | 6.30E-05 | 0.0014127 |
| RP1-63M2.5 | 3.149682873 | -1.37849535 | 0.458205372 | -3.008466146 | 0.002625701 | 0.020291266 |
| RP11-196G18.21 | 6.440110609 | -1.373235771 | 0.276360694 | -4.968998118 | 6.73E-07 | 4.87E-05 |
| SLC4A11 | 432.8380825 | -1.215925858 | 0.301259558 | -4.03614035 | 5.43E-05 | 0.001280302 |
| ELN | 8857.212249 | 1.14114144 | 0.225984812 | 5.049637756 | 4.43E-07 | 3.52E-05 |
| DNAH3 | 138.6743595 | -1.003199731 | 0.251557065 | -3.987960874 | 6.66E-05 | 0.001463929 |
| NXNL1 | 6.856664964 | 2.932378097 | 0.593623627 | 4.939793442 | 7.82E-07 | 5.44E-05 |
| PDIA2 | 16363.67884 | 2.188052256 | 0.573951345 | 3.812260874 | 0.000137701 | 0.002506847 |
| AC006159.5 | 42.31777266 | 1.507155301 | 0.303426201 | 4.967123123 | 6.80E-07 | 4.91E-05 |
| FHL5 | 1008.173098 | 1.133531068 | 0.281710245 | 4.023748112 | 5.73E-05 | 0.001328734 |
| HNRNPA1P3 | 26.91454456 | -1.960569986 | 0.477086139 | -4.109467503 | 3.97E-05 | 0.001017391 |
| GALNT15 | 1069.707948 | 1.323911561 | 0.305726972 | 4.330372129 | 1.49E-05 | 0.00048292 |
| HK2 | 7552.292499 | -1.073114853 | 0.235256787 | -4.561461831 | 5.08E-06 | 0.000223676 |
| GCKR | 200.6970858 | -1.019021465 | 0.319991074 | -3.184530905 | 0.001449888 | 0.013219171 |
| CEL | 726161.4169 | 1.898094993 | 0.648027635 | 2.929034029 | 0.003400172 | 0.024490156 |
| BCMO1 | 186.9483138 | -1.237237527 | 0.336927196 | -3.672121278 | 0.000240545 | 0.003681346 |
| ADAMTS4 | 5888.130231 | 1.024793664 | 0.236846456 | 4.326827093 | 1.51E-05 | 0.000488189 |
| NPTX1 | 1167.746473 | -1.313998253 | 0.308182825 | -4.263697213 | 2.01E-05 | 0.000606873 |
| MATN3 | 2488.251821 | -1.12344377 | 0.335596648 | -3.347601282 | 0.000815142 | 0.00873651 |
| APOA2 | 55.8553436 | 1.142912577 | 0.436342504 | 2.619301503 | 0.008811003 | 0.047980042 |
| snoU13 | 3.153207325 | -1.136552972 | 0.368642328 | -3.083077783 | 0.002048716 | 0.016947323 |
| KRT14 | 160.7196619 | -2.270303855 | 0.482471254 | -4.705573306 | 2.53E-06 | 0.000130443 |
| ALDH3B2 | 104.6598404 | -1.824976945 | 0.490088963 | -3.723766672 | 0.000196272 | 0.003175784 |
| RP11-702B10.2 | 28.38743964 | -2.100213378 | 0.537388922 | -3.908181374 | 9.30E-05 | 0.001880851 |
| MTHFD2P1 | 14.38175309 | 1.780179453 | 0.443711952 | 4.012016002 | 6.02E-05 | 0.001371258 |
| CTA-134P22.2 | 156.2223462 | 1.204316184 | 0.321547502 | 3.745375653 | 0.000180124 | 0.003014565 |
| PHGDH | 2705.093203 | 1.283798069 | 0.259526285 | 4.946697663 | 7.55E-07 | 5.29E-05 |
| ZNF695 | 65.58514684 | -1.046423787 | 0.365880478 | -2.860015361 | 0.004236205 | 0.028614379 |
| NEU4 | 48.77347376 | 1.630277434 | 0.415427504 | 3.924336787 | 8.70E-05 | 0.001789634 |
| CTD-2311M21.2 | 11.5324558 | 1.020152901 | 0.359124237 | 2.840668481 | 0.004501909 | 0.029911727 |
| CTD-2377D24.2 | 9.29836918 | -2.678939919 | 0.670072769 | -3.997983568 | 6.39E-05 | 0.001425155 |
| AC009133.20 | 15.08048455 | -1.035491659 | 0.35869192 | -2.886855269 | 0.003891132 | 0.026947438 |
| CTC-558O2.2 | 20.70537243 | 1.317979701 | 0.384458803 | 3.428142865 | 0.000607726 | 0.007122765 |
| PF4 | 44.66029722 | 1.112650186 | 0.366964666 | 3.032036293 | 0.0024291 | 0.019186298 |
| THRSP | 545.2957037 | 3.657154107 | 0.498492198 | 7.336431991 | 2.19E-13 | 3.06E-10 |
| KCNA1 | 86.32142955 | 2.198084477 | 0.422174024 | 5.206583895 | 1.92E-07 | 1.89E-05 |
| hsa-mir-7162 | 16.62952071 | 1.729253061 | 0.447348886 | 3.865557994 | 0.000110836 | 0.002149195 |
| RP11-175B12.2 | 57.91932926 | -1.170476526 | 0.268728971 | -4.355602305 | 1.33E-05 | 0.000443419 |
| FOS | 20237.71025 | 1.089195216 | 0.241874713 | 4.50313802 | 6.70E-06 | 0.000271006 |
| TMPRSS4-AS1 | 3.067086198 | -1.267554388 | 0.466509351 | -2.717103925 | 0.006585593 | 0.039099449 |
| RP11-375I20.6 | 51.82891851 | 1.054820841 | 0.224057279 | 4.707817779 | 2.50E-06 | 0.000129664 |
| LYZ | 38871.97945 | -1.102973143 | 0.303978885 | -3.628453147 | 0.000285124 | 0.0041394 |
| PIP | 8.396659272 | -1.576388878 | 0.483983172 | -3.257115057 | 0.001125508 | 0.011092882 |
| HSPA8P5 | 113.0619443 | 1.544319602 | 0.391309825 | 3.946539292 | 7.93E-05 | 0.001671282 |
| AQP2 | 52.89544385 | -1.741412326 | 0.407534109 | -4.27304681 | 1.93E-05 | 0.000589727 |
| LBP | 261.1179558 | 2.009312796 | 0.471096061 | 4.265187006 | 2.00E-05 | 0.000603772 |
| HOXB5 | 409.3828207 | -1.225201367 | 0.224352621 | -5.461052156 | 4.73E-08 | 6.24E-06 |
| CDC20P1 | 44.20868079 | 1.254309491 | 0.272438281 | 4.604013383 | 4.14E-06 | 0.000191249 |
| GPR112 | 61.05882783 | 2.485226924 | 0.528236963 | 4.704757712 | 2.54E-06 | 0.000130755 |
| DUOX2 | 9695.175969 | -1.200124623 | 0.418521227 | -2.867535847 | 0.004136819 | 0.028143097 |
| CTA-363E6.5 | 14.29308194 | -1.333946202 | 0.404275665 | -3.299595589 | 0.000968242 | 0.009938567 |
| RN7SL347P | 7.560931614 | 1.017616439 | 0.275603219 | 3.692324219 | 0.000222214 | 0.00347722 |
| RP11-281P23.1 | 81.7904612 | -1.125448123 | 0.274592791 | -4.09860768 | 4.16E-05 | 0.001052467 |
| RP11-863P13.4 | 137.563752 | 1.91345748 | 0.294196878 | 6.504003352 | 7.82E-11 | 3.96E-08 |
| FERMT1 | 2860.615301 | -1.383754003 | 0.269802184 | -5.128772425 | 2.92E-07 | 2.54E-05 |
| BDKRB1 | 258.5319753 | 1.595750161 | 0.237968799 | 6.70571172 | 2.00E-11 | 1.28E-08 |
| GUCY1B2 | 118.8980166 | -1.662624116 | 0.336141211 | -4.946207307 | 7.57E-07 | 5.30E-05 |
| RP11-103H7.5 | 8.492821431 | -1.454357948 | 0.440578481 | -3.301019023 | 0.000963344 | 0.009901313 |
| RP11-167N4.2 | 26.60346928 | -1.853951283 | 0.503921703 | -3.679046311 | 0.000234108 | 0.003608313 |
| CEBPA | 2647.68655 | 1.298051806 | 0.250775393 | 5.17615301 | 2.27E-07 | 2.12E-05 |
| RP11-17A4.3 | 27.57752877 | 1.932678356 | 0.413101867 | 4.678454658 | 2.89E-06 | 0.000144319 |
| CXorf22 | 43.13136051 | -1.571588043 | 0.468558453 | -3.354091754 | 0.00079626 | 0.008619622 |
| KCNN4 | 1420.079826 | -1.386194887 | 0.249826002 | -5.548641364 | 2.88E-08 | 4.39E-06 |
| MIA | 421.9139666 | -1.804500653 | 0.334186059 | -5.399688605 | 6.68E-08 | 8.06E-06 |
| NMNAT1P1 | 23.45352427 | 1.344101439 | 0.335897298 | 4.001525014 | 6.29E-05 | 0.001412066 |
| TRBV26OR9-2 | 11.7417346 | 1.522500965 | 0.347304096 | 4.383769101 | 1.17E-05 | 0.000404337 |
| RP1-13D10.2 | 8.785443465 | 1.033012016 | 0.312885174 | 3.301569081 | 0.000961457 | 0.009889738 |
| CDH3 | 2982.771439 | -1.413366935 | 0.283109287 | -4.992301566 | 5.97E-07 | 4.48E-05 |
| RP11-725K16.2 | 36.88826036 | -1.831884044 | 0.543037781 | -3.373400729 | 0.000742458 | 0.008188243 |
| MIR665 | 2.479428554 | 1.590704657 | 0.414911603 | 3.833839895 | 0.000126158 | 0.002356134 |
| RP11-619J20.1 | 12.18937879 | -1.347883884 | 0.308898129 | -4.363522339 | 1.28E-05 | 0.000431117 |
| MT1A | 142.9608054 | 2.277156588 | 0.415357302 | 5.482404126 | 4.20E-08 | 5.72E-06 |
| MIR149 | 4.65349977 | 1.208815939 | 0.372806877 | 3.242472212 | 0.001184975 | 0.011483127 |
| RP4-681L3.2 | 4.715762851 | -2.150184461 | 0.623385927 | -3.449202764 | 0.000562244 | 0.006702757 |
| KLK8 | 202.2238039 | -1.545735772 | 0.552912588 | -2.795624129 | 0.005179961 | 0.033010715 |
| RP3-340N1.2 | 182.6427539 | -1.067402776 | 0.369098695 | -2.891916959 | 0.003828991 | 0.026653252 |
| HAVCR1 | 320.5927876 | -1.79069783 | 0.393051514 | -4.555885843 | 5.22E-06 | 0.000228304 |
| ADH1B | 17326.4357 | 2.705299593 | 0.357096995 | 7.575811691 | 3.57E-14 | 6.16E-11 |
| RP3-443C4.2 | 5.307163307 | 1.069121506 | 0.399796555 | 2.674163881 | 0.007491583 | 0.042791019 |
| AGTR1 | 566.614093 | 1.535626153 | 0.321710406 | 4.773318245 | 1.81E-06 | 0.000101239 |
| ADRA1A | 276.5378601 | 2.899618264 | 0.455948097 | 6.359535839 | 2.02E-10 | 8.05E-08 |
| DGCR5 | 455.9153549 | 1.483234111 | 0.349131711 | 4.248351162 | 2.15E-05 | 0.000635634 |
| MIR299 | 5.630486581 | 1.373399212 | 0.36565996 | 3.755946403 | 0.000172688 | 0.002920189 |
| RAB27B | 3971.443561 | -1.130389465 | 0.235142438 | -4.807254168 | 1.53E-06 | 8.99E-05 |
| HOXB8 | 76.34909938 | -1.486015171 | 0.404797084 | -3.671012534 | 0.000241591 | 0.003694455 |
| RP11-284F21.9 | 159.4443191 | -1.406415639 | 0.366487081 | -3.837558573 | 0.000124264 | 0.002334903 |
| OR2W3 | 8.021254085 | 1.183959422 | 0.387244704 | 3.057393449 | 0.00223271 | 0.018079421 |
| RNVU1-19 | 33.58521037 | 1.643279743 | 0.526658695 | 3.120198637 | 0.001807291 | 0.015444953 |
| RP11-10D7.5 | 4.711078952 | 2.423302389 | 0.615957582 | 3.934203361 | 8.35E-05 | 0.001737871 |
| CTD-2227E11.1 | 266.6620067 | 1.158021144 | 0.325903758 | 3.553261093 | 0.000380487 | 0.00508119 |
| RNU6-403P | 7.732945766 | -1.472998753 | 0.455410379 | -3.23444265 | 0.001218804 | 0.011700458 |
| LRP1B | 479.0546762 | 1.048857152 | 0.277578396 | 3.778597927 | 0.000157714 | 0.002737898 |
| RN7SL653P | 39.16799159 | 1.037728025 | 0.218836127 | 4.742032481 | 2.12E-06 | 0.000114913 |
| AC114808.3 | 5.501860724 | 1.340472819 | 0.454573066 | 2.948861072 | 0.003189473 | 0.023431331 |
| MIR329-1 | 4.214431654 | 1.389003662 | 0.410382476 | 3.384656367 | 0.000712674 | 0.007956309 |
| RP11-350J20.12 | 85.08286779 | -1.329886701 | 0.369254105 | -3.601548864 | 0.000316327 | 0.004452983 |
| AC005394.1 | 5.105555046 | 1.764878549 | 0.643221708 | 2.743810611 | 0.006073056 | 0.036876181 |
| SNORD3D | 824.8725961 | 1.250672895 | 0.226837825 | 5.513511238 | 3.52E-08 | 5.06E-06 |
| RP11-547C13.1 | 3.756877811 | 1.559623321 | 0.46623562 | 3.34513978 | 0.000822411 | 0.008803163 |
| SPOCD1 | 318.5966226 | -1.004747246 | 0.283428269 | -3.544978944 | 0.000392644 | 0.005190792 |
| RP11-384F7.2 | 61.21421138 | 1.580767318 | 0.363426599 | 4.349619217 | 1.36E-05 | 0.000451435 |
| FAM166A | 22.48938941 | 1.050365454 | 0.288190061 | 3.644697015 | 0.000267707 | 0.003951227 |
| C17orf107 | 592.6496004 | 1.171503564 | 0.179243566 | 6.535819323 | 6.33E-11 | 3.33E-08 |
| CEACAM5 | 15276.12345 | -1.409407152 | 0.523874614 | -2.69035207 | 0.007137667 | 0.041405786 |
| DMBX1 | 33.82404421 | -1.727984612 | 0.535925431 | -3.224300456 | 0.001262808 | 0.012013257 |
| RP11-125O18.1 | 10.97155621 | 1.280973955 | 0.452241861 | 2.832497531 | 0.004618592 | 0.030423597 |
| RPL17P11 | 19.20847597 | -2.415697138 | 0.562576899 | -4.293985663 | 1.75E-05 | 0.000546937 |
| CLEC3B | 1001.249276 | 2.124309753 | 0.295483619 | 7.189264025 | 6.51E-13 | 6.35E-10 |
| BX470102.3 | 248.0694151 | -1.432737749 | 0.267947614 | -5.347081575 | 8.94E-08 | 1.02E-05 |
| AC019117.1 | 106.572636 | -1.093544527 | 0.348473536 | -3.138099208 | 0.001700473 | 0.014807939 |
| RP11-553N19.1 | 5.188782559 | 1.34192844 | 0.476313471 | 2.81732204 | 0.004842594 | 0.031562254 |
| PDZRN4 | 236.3158194 | 1.103832915 | 0.353711537 | 3.120715047 | 0.001804125 | 0.015424657 |
| LINC00511 | 830.013964 | -1.195125965 | 0.299691487 | -3.98785424 | 6.67E-05 | 0.001463929 |
| RP11-511I11.2 | 6.197895095 | -2.020887819 | 0.761051354 | -2.655389559 | 0.007921687 | 0.044594511 |
| MIR3138 | 15.224043 | 1.340844132 | 0.241322972 | 5.556222514 | 2.76E-08 | 4.30E-06 |
| FAM83H-AS1 | 1085.086203 | -1.323169653 | 0.312134104 | -4.239106325 | 2.24E-05 | 0.000655164 |
| APOA1 | 186.8506861 | 2.519554193 | 0.560018277 | 4.499057082 | 6.83E-06 | 0.000273548 |
| SHH | 722.0778677 | -1.273427012 | 0.377504969 | -3.373272183 | 0.000742805 | 0.008188243 |
| CYP4B1 | 148.4856784 | 1.053889692 | 0.383877838 | 2.74537779 | 0.006044127 | 0.036757759 |
| EGFL7 | 2256.430893 | 1.0762739 | 0.182658986 | 5.892258163 | 3.81E-09 | 8.80E-07 |
| HJURP | 512.5380925 | -1.011265026 | 0.248911276 | -4.062752977 | 4.85E-05 | 0.00118271 |
| NPR1 | 1642.094072 | 1.755972546 | 0.241022148 | 7.285523601 | 3.20E-13 | 4.03E-10 |
| VIPR2 | 610.2003361 | 1.568171202 | 0.354297825 | 4.426138384 | 9.59E-06 | 0.000350608 |
| RP11-318K12.1 | 365.3707179 | -2.154431349 | 0.465439655 | -4.628809178 | 3.68E-06 | 0.000173414 |
| LRP2 | 117.4012918 | 2.081982464 | 0.552793998 | 3.766289925 | 0.000165691 | 0.002833831 |
| EFNA5 | 2366.271752 | -1.053811965 | 0.231395286 | -4.554163493 | 5.26E-06 | 0.000229409 |
| AOX1 | 8543.942432 | 1.274213049 | 0.314374442 | 4.053169975 | 5.05E-05 | 0.001219259 |
| HIST1H1PS1 | 18.47048733 | -1.051550428 | 0.290231705 | -3.623141136 | 0.000291047 | 0.004208148 |
| EPS15P1 | 28.57378023 | -3.466354355 | 0.768835095 | -4.508579768 | 6.53E-06 | 0.000265928 |
| RP11-893F2.9 | 52.68406523 | -1.134607201 | 0.2727811 | -4.159405484 | 3.19E-05 | 0.000864657 |
| MEDAG | 2491.34861 | 1.614695081 | 0.327636902 | 4.928306526 | 8.29E-07 | 5.66E-05 |
| 1-Mar | 905.194539 | 1.332025434 | 0.216514082 | 6.152142246 | 7.64E-10 | 2.44E-07 |
| SEMA3G | 1708.833173 | 1.983315018 | 0.262354026 | 7.559689649 | 4.04E-14 | 6.30E-11 |
| SLC22A31 | 294.4176522 | 2.031360112 | 0.588259468 | 3.453170279 | 0.000554039 | 0.006627226 |
| TCF15 | 36.450849 | 1.12704776 | 0.301723687 | 3.735363868 | 0.000187444 | 0.003082825 |
| HMGN1P9 | 2.944342215 | 2.177022993 | 0.594122845 | 3.664264069 | 0.000248051 | 0.003761967 |
| RP11-488L4.1 | 14.66645527 | -1.314012869 | 0.470117408 | -2.795073839 | 0.005188786 | 0.033045357 |
| MIR154 | 3.382447117 | 1.508812367 | 0.392439838 | 3.844697252 | 0.000120701 | 0.002289275 |
| GRP | 92.75073483 | -1.362159021 | 0.420671062 | -3.238062093 | 0.001203446 | 0.011607317 |
| RP11-474I16.8 | 5.304165224 | 1.434820624 | 0.473930184 | 3.027493654 | 0.002465909 | 0.01939845 |
| AL132709.3 | 5.399242096 | 1.009384021 | 0.289708443 | 3.4841374 | 0.000493726 | 0.006094601 |
| ADAMTS9 | 10052.86119 | 1.341420271 | 0.216316901 | 6.201181077 | 5.60E-10 | 1.85E-07 |
| AC000036.4 | 15.98822491 | 1.217093745 | 0.378071354 | 3.219217045 | 0.001285412 | 0.012148478 |
| Y_RNA | 52.01810715 | 1.535340812 | 0.338161014 | 4.540265583 | 5.62E-06 | 0.000239702 |
| RP11-161H23.5 | 4.357645805 | -1.353959416 | 0.500562188 | -2.704877531 | 0.006832958 | 0.040206908 |
